# Supplementary material for: Cognitive outcomes of the at-home brain balance program
Source: Front Child Adolesc Psychiatry. 2024 Dec 2;3:1450695. doi: 10.3389/frcha.2024.1450695 (PMC11731937; doi:10.3389/frcha.2024.1450695)
Supplement: Supplementary file 1 [file Datasheet1.pdf]

```
library(emmeans)
library(ggplot2)
library(psych)
library(rstatix)
library(lme4)
library(phia)
library(tidyverse)
library(CGPfunctions)
library(ggpubr)
library(dplyr)

setwd("/Users/rye/Documents/Documents - Mac/BrainBalance/data")
options(contrasts = c("contr.sum", "contr.poly"), scipen=40)

# run RM anova separately on the at-home and in-center programs ----
df <- read.table("df_rmANOVA_virtual.csv", sep=",", header=T) %>%
  gather(time_point, score, pre_score:post_score) %>%
  mutate(trt_grp = dplyr::recode(trt_grp, ">=90 days apart"= "BB", "<=30
days apart"="CTRL")) %>%
  mutate(test_name = dplyr::recode(test_name, "DT_final_score"="DT",
"FM_final_score"="FM", "ML_max_score"="ML",
                                   "RT_final_score"="RT",
"TS_max_score"="TS", "SS_max_score"="SS")) %>%
  mutate(time_point = dplyr::recode(time_point, "post_score"="post test",
"pre_score"="pre test")) %>%
  convert_as_factor(trt_grp, time_point, test_name)

## transform test scores into z scores ----
df_z <- df %>%
  group_by(test_name) %>%
  mutate(z_score = (score - mean(score, na.rm=TRUE)) / sd(score,
na.rm=TRUE)) %>%
  ungroup()

## Levene's test ----
df_z %>%
   group_by(test_name, time_point) %>%
   levene_test(z_score ~ trt_grp)

## build anova table ----
res.virtual <- df_z %>%
  anova_test(dv=z_score, wid = user_id, within = c(time_point,
test_name), between=trt_grp, effect.size = "pes")
get_anova_table(res.virtual, correction="GG")

## post-hoc test for the interaction between trt_grp and time_point ----
trt.effect <- df_z %>%
  group_by(time_point) %>% drop_na() %>%
  anova_test(dv = z_score, wid = user_id, between = trt_grp, effect.size
= "pes", type=2)
trt.effect

## plot 2-way interaction ----
df_z$time_point <- as.character(df_z$time_point)
```

```
df2plot <- df_z %>% dplyr::rename(treatment = "trt_grp") %>%
  mutate(time_point = dplyr::recode(time_point, `pre test`="0 pre-test",
`post test`="1 post-test"))
Plot2WayANOVA(z_score ~ time_point * treatment, df2plot, plottype =
"line", xlab = "", ylab='z score',
              ggplot.component = theme(
                axis.text.x = element_text(size=14),
                legend.title = element_text(color = "white"),
                legend.text = element_text(size=14),
                axis.title.y = element_text(size=14),
                axis.text.y = element_text(size=14)
                )
              )

Plot2WayANOVA(z_score ~ time_point * test_name, df2plot, plottype =
"line", xlab = "", ylab='z score',
                    offset.style = "wide",ci.line.size = 0.5, mean.size =
2, interact.line.size = 1,
              ggplot.component = theme(axis.text.x =
element_text(size=14),
                                       legend.title = element_text(color
= "white"),
                                       legend.text =
element_text(size=14),
                                       axis.title.y =
element_text(size=14),
                                       axis.text.y =
element_text(size=14)
              )

)


df_z$time_point <- as.factor(df_z$time_point)

# compare pre to post scores for BB and CTRL, respectively
trt_2.effect <- df_z %>%
  group_by(test_name, time_point) %>%
  anova_test(dv = z_score, wid = user_id, between = trt_grp, effect.size
= "pes")
trt_2.effect

#### get Standard error for CIs


length2 <- function(x, na.rm = FALSE) {
  # New version of length which can handle NA's: if na.rm==T, don't count
them
  if (na.rm) sum(!is.na(x))
  else       length(x)
}

summarySE <- function(data = NULL, measurevar, groupvars = NULL, na.rm =
FALSE,
```

```
                      conf.interval = .95, .drop = TRUE) {

  groupvars  <- rlang::syms(groupvars)
  measurevar <- rlang::sym(measurevar)

  datac <- data %>%
    dplyr::group_by(!!!groupvars) %>%
    dplyr::summarise(
      N             = length2(!!measurevar, na.rm = na.rm),
      sd            = sd     (!!measurevar, na.rm = na.rm),
      !!measurevar := mean   (!!measurevar, na.rm = na.rm),
      se            = sd / sqrt(N),
      # Confidence interval multiplier for standard error
      # Calculate t-statistic for confidence interval:
      # e.g., if conf.interval is .95, use .975 (above/below), and use
df=N-1
      ci            = se * qt(conf.interval/2 + .5, N - 1)
    ) %>%
    dplyr::ungroup() %>%
    # Rearrange the columns so that sd, se, ci are last
    dplyr::select(seq_len(ncol(.) - 4), ncol(.) - 2, sd, se, ci)

  datac
}

## prepare a tibble dataframe for plotting the two-way interaction at
post-test
df_virtual_post <- df_z %>%
  filter(time_point == "post test") %>%
  summarySE("z_score", c("trt_grp", "test_name", "time_point"),
          conf.interval = .95, na.rm = TRUE, .drop = FALSE) %>%
  mutate(trt_grp = dplyr::recode(trt_grp, ">=90 days apart"= "BB", "<=30
days apart"="CTRL")) %>%
  mutate(test_name = dplyr::recode(test_name, "DT"="Double Trouble",
"FM"="Feature Match", "ML"="Monkey Ladder",
                                   "RT"="Rotations", "TS"="Token Search",
"SS"="Spatial Span")) %>%
  rename(treatment = "trt_grp")

#### at-home, facet plots for every cognitive task, comparing post-test
BB to CTRL
ggplot(df_virtual_post, aes(x = treatment, y = z_score, colour =
treatment)) + geom_point(stat = "identity") +
  facet_wrap(~test_name, ) + geom_errorbar(stat = "identity", aes(ymin =
z_score-ci, ymax = z_score+ci), width=0.2, size=1.5) +
  labs(x="", y="z score") + theme(axis.title.y = element_text(size=14),
axis.text.x = element_text(size=14),  strip.text.x = element_text(size =
14, color = 'steelblue'), legend.title = element_text(color =
"white"),legend.text = element_text(size=14),)

## facet_wrap does not have scales = "free_y",

# in-center analysis -----
## load and preprocess in-center data ----
```

```
df_init <- read.table("df_rmANOVA_init.csv", sep=",", header=T) %>%
  gather(time_point, score, pre_score:post_score) %>%
  mutate(trt_grp = dplyr::recode(trt_grp, ">=90 days apart"= "BB", "<=30
days apart"="CTRL")) %>%
  mutate(test_name = dplyr::recode(test_name, "DT_final_score"="DT",
"FM_final_score"="FM", "ML_max_score"="ML",
                                   "RT_final_score"="RT",
"TS_max_score"="TS", "SS_max_score"="SS")) %>%
  mutate(time_point = dplyr::recode(time_point, "post_score"="post test",
"pre_score"="pre test")) %>%
  convert_as_factor(trt_grp, test_name, time_point)


# z score of the in-center test scores
df_init_z <- df_init %>%
  group_by(test_name) %>%
  mutate(z_score = (score - mean(score, na.rm=TRUE)) / sd(score,
na.rm=TRUE)) %>%
  ungroup()

## Levene's test ----
df_init_z %>%
  group_by(time_point, test_name) %>%
  levene_test(z_score ~  trt_grp)

## RM ANOVA on in-center data ----
res.init <- df_init_z %>% drop_na() %>%
  anova_test(dv= z_score, wid = user_id, within = c(time_point,
test_name), between=trt_grp, effect.size = "pes", type=3)
get_anova_table(res.init, correction="GG")

## two-way interaction at each time levels ----
two.way <- df_init_z %>%
  group_by(time_point) %>%
  drop_na() %>%
  anova_test(dv = z_score, wid = user_id, between =  trt_grp, effect.size
= "pes", type=3)
get_anova_table(two.way)

df_init_z$time_point <- as.character(df_init_z$time_point)
init2plot <- df_init_z %>% dplyr::rename(treatment = "trt_grp") %>%
  mutate(time_point = dplyr::recode(time_point, `pre test`="0 pre-test",
`post test`="1 post-test"))
Plot2WayANOVA(z_score ~ time_point * treatment, init2plot, plottype =
"line", xlab = "", ylab='z score',
              ggplot.component = theme(axis.text.x =
element_text(size=14),
                                       legend.title = element_text(color
= "white"),
                                       legend.text =
element_text(size=14),
                                       axis.title.y =
element_text(size=14),
```

```
                                     axis.text.y =
element_text(size=14)
                                     )
             )

Plot2WayANOVA(z_score ~ time_point * test_name, df2plot, plottype =
"line", xlab = "", ylab='z score',
             offset.style = "wide",ci.line.size = 0.5, mean.size = 2,
interact.line.size = 1,
             ggplot.component = theme(axis.text.x =
element_text(size=14),
                                     legend.title = element_text(color
= "white"),
                                     legend.text =
element_text(size=14),
                                     axis.title.y =
element_text(size=14),
                                     axis.text.y =
element_text(size=14)
             )

)

df_init_z$time_point <- as.factor(df_init_z$time_point)
## isolate the treatment effect for each cognitive task ----
by_task <- df_init_z %>%
  group_by(test_name, time_point) %>%
  drop_na() %>%
  anova_test(dv = z_score, wid = user_id, between =  trt_grp, effect.size
= "pes", type=3)
get_anova_table(by_task)

## Plot each task on its own panel, showing the trt_grp by test_name
simple interaction ----
df_init_post <- df_init_z %>%
  filter(time_point == "post test") %>%
  summarySE("z_score", c("trt_grp", "test_name", "time_point"),
          conf.interval = .95, na.rm = TRUE, .drop = FALSE) %>%
  mutate(trt_grp = dplyr::recode(trt_grp, ">=90 days apart"= "BB", "<=30
days apart"="CTRL")) %>%
  mutate(test_name = dplyr::recode(test_name, "DT"="Double Trouble",
"FM"="Feature Match", "ML"="Monkey Ladder",
                                "RT"="Rotations", "TS"="Token Search",
"SS"="Spatial Span")) %>%
  rename(treatment = "trt_grp")

ggplot(df_init_post, aes(x = treatment, y = z_score, colour = treatment))
+ geom_point(stat = "identity") +
  facet_wrap(~test_name, ) + geom_errorbar(stat = "identity", aes(ymin =
z_score-ci, ymax = z_score+ci), width=0.2, size=1.5) +
  labs(x="", y="z score") + theme(axis.title.y = element_text(size=14),
axis.text.x = element_text(size=14),  strip.text.x = element_text(size =
14, color = 'steelblue'))
```

```
#  compare raw scores between at-home BB and in-center BB ----
df_2prog <- read.table("2programs_rmANOVA.csv", sep=",", header=T)
df_2prog <- df_2prog %>% convert_as_factor(trt_grp, test_name,
time_point)

df_2prog %>%
  group_by(test_name, time_point) %>%
  levene_test(score ~ trt_grp)

res.2prog <- df_2prog %>%
  group_by(test_name) %>%
  mutate(z_score = (score - mean(score, na.rm=TRUE)) / sd(score,
na.rm=TRUE)) %>%
  ungroup() %>%
  anova_test(dv = z_score, wid = user_id, within = c(test_name,
time_point), between=trt_grp, effect.size = "pes")
get_anova_table(res.2prog, correction="GG")
```
