# Supplementary material for: Cognitive outcomes of the at-home brain balance program
Source: Front Child Adolesc Psychiatry. 2024 Dec 2;3:1450695. doi: 10.3389/frcha.2024.1450695 (PMC11731937; doi:10.3389/frcha.2024.1450695)
Supplement: Supplementary file 2 [file Datasheet2.pdf]

```
{
 "cells": [
  {
   "cell_type": "code",
   "execution_count": 3,
   "metadata": {
    "metadata": {}
   },
   "outputs": [],
   "source": [
    "import pandas as pd\n",
    "import os \n",
    "import numpy as np\n",
    "from numpy import mean\n",
    "from numpy import var\n",
    "from math import sqrt\n",
    "from scipy import stats, linalg\n",
    "import warnings\n",
    "import statsmodels.api as sm\n",
    "from statsmodels.stats.power import TTestIndPower\n",
    "from statsmodels.stats.multitest import multipletests, 
fdrcorrection\n",
    "from statsmodels.formula.api import ols\n",
    "import matplotlib.pyplot as plt\n",
    "import seaborn as sns\n",
    "import plotly.express as px\n",
    "\n",
    "# import pingouin as pg\n",
    "\n",
    "%matplotlib inline\n",
    "\n",
    "\n",
    "# Suppress FutureWarning messages\n",
    "warnings.simplefilter(action='ignore', category=FutureWarning)\n",
    "warnings.simplefilter(action='ignore', 
category=DeprecationWarning)\n",
    "\n",
    "idx = pd.IndexSlice\n",
    "\n",
    "mydir = '/Users/rye/Documents/Documents - Mac/BrainBalance'\n",
    "myfile = 'Creyos-BrainBalance-2023-11-30.csv'\n",
    "\n",
    "crm_file = 'Adult Pre Vs Post Paperwork_November 2023_De-
identified.xlsx'\n",
    "\n",
    "nonenroll_file = 'Non_Enrollment 
Type_Multiple_Tests_Identifier_Only_2.xlsx'\n",
    "creyos_file_new = 'Creyos-BrainBalance- Enrollment Type-2023-12-
05.xlsx'\n",
    "#creyos_file_new = 'SCI-92-Creyos-BrainBalance-2023-12-20.xlsx'\n",
    "\n",
    "mycols = ['user_id', 'client_id', 'report', 'test_date', 
'batch_name',\n",
    "        'trial_name', 'birthdate',  'enroll type','gender',\n",
```

```
    "       'age_at_test', 'SS_avg_score', 'DT_final_score',
'ML_max_score', 'RT_final_score', 'FM_final_score', 'TS_max_score']"
   ]
  },
  {
   "cell_type": "code",
   "execution_count": 4,
   "metadata": {
    "metadata": {}
   },
   "outputs": [
    {
     "name": "stdout",
     "output_type": "stream",
     "text": [
      "<class 'pandas.core.frame.DataFrame'>\n",
      "Int64Index: 2389 entries, 0 to 2390\n",
      "Data columns (total 9 columns):\n",
      " #   Column                        Non-Null Count  Dtype
\n",
      "---  ------                        --------------  -----
\n",
      " 0   user_id                       2389 non-null   string
\n",
      " 1   client_id                     2389 non-null   object
\n",
      " 2   email                         2389 non-null   object
\n",
      " 3   birthdate                     2388 non-null
datetime64[ns]\n",
      " 4   Status                        2048 non-null   object
\n",
      " 5   Program Fit, No Intervention  159 non-null    object
\n",
      " 6   Hours                         170 non-null    object
\n",
      " 7   Center                        1523 non-null   object
\n",
      " 8   Unnamed: 8                    2 non-null      object
\n",
      "dtypes: datetime64[ns](1), object(7), string(1)\n",
      "memory usage: 186.6+ KB\n"
     ]
    }
   ],
   "source": [
    "#df_crm = pd.read_excel(os.path.join(mydir, crm_file))\n",
    "\n",
    "# RJ notes indicate hours completed differ from sessions completed.
\n",
    "# In all cases, sessions completed shows 0 whereas hours are greater
than zero.\n",
    "df_NoEnroll = pd.read_excel(os.path.join(mydir, nonenroll_file),
skiprows=11)\n",
```

"df_NoEnroll['user_id'] = df_NoEnroll['user_id'].astype('string')\n",
"df_NoEnroll['Status'] =
df_NoEnroll['Status'].str.strip().str.capitalize()\n",
"df_NoEnroll['Status'] = df_NoEnroll['Status'].replace({'Could not
identify':'Can not identify' ,'Unable to identify':'Can not identify',
'Vitual':'Virtual', 'Hyrbid':'Hybrid', 'In center - control':'In center
did not enroll'})\n",
"df_NoEnroll['Status'] =
df_NoEnroll['Status'].replace({'Virtual':'Virtual New'})\n",
"df_NoEnroll = df_NoEnroll[(df_NoEnroll['Program Fit, No
Intervention']!='Not a program fit') | (df_NoEnroll['Status']=='Can not
identify')]\n",
"##########\n",
"#df_NoEnroll = df_NoEnroll[~df_NoEnroll['Status'].isin(['In center
did not enroll', 'Virtual did not enroll', 'Adult did not enroll', 'Did
not enroll'])]\n",
"############\n",
"\n",
"\n",
"## load new Creyos data with SS max score\n",
"df_SSmax = pd.read_excel(os.path.join(mydir, 'SCI-92-Creyos-
BrainBalance-2024-01-31.xlsx'))\n",
"df_SSmax['user_id'] = df_SSmax['user_id'].astype('string')\n",
"\n",
"###########\n",
"# load old Creyos data with enroll type\n",
"###########\n",
"#df_SSmax['test_date'] = pd.to_datetime(df_SSmax['test_date'])\n",
"df = pd.read_excel(os.path.join(mydir, creyos_file_new))\n",
"df = df[mycols]\n",
"df['user_id'] = df['user_id'].astype('string')\n",
"#df['birthdate'] = pd.to_datetime(df['birthdate'],
errors='coerce')\n",
"#df['test_date'] = pd.to_datetime(df['test_date'])\n",
"df = df.sort_values(by=['user_id',
'test_date']).reset_index().drop(columns='index')\n",
"\n",
"##########\n",
"df_NoEnroll.info()"
]
},
{
"cell_type": "code",
"execution_count": 5,
"metadata": {},
"outputs": [
{
"name": "stdout",
"output_type": "stream",
"text": [
"<class 'pandas.core.frame.DataFrame'>\n",
"Int64Index: 2389 entries, 0 to 2390\n",
"Data columns (total 9 columns):\n",

```
      " #   Column                          Non-Null Count  Dtype         
\n",
      "---  ------                          --------------  -----         
\n",
      " 0   user_id                         2389 non-null   string        
\n",
      " 1   client_id                       2389 non-null   object        
\n",
      " 2   email                           2389 non-null   object        
\n",
      " 3   birthdate                       2388 non-null   
datetime64[ns]\n",
      " 4   Status                          2048 non-null   object        
\n",
      " 5   Program Fit, No Intervention  159 non-null    object        
\n",
      " 6   Hours                           170 non-null    object        
\n",
      " 7   Center                          1523 non-null   object        
\n",
      " 8   Unnamed: 8                      2 non-null      object        
\n",
      "dtypes: datetime64[ns](1), object(7), string(1)\n",
      "memory usage: 186.6+ KB\n"
     ]
    },
    {
     "data": {
      "text/html": [
       "<div>\n",
       "<style scoped>\n",
       "    .dataframe tbody tr th:only-of-type {\n",
       "        vertical-align: middle;\n",
       "    }\n",
       "\n",
       "    .dataframe tbody tr th {\n",
       "        vertical-align: top;\n",
       "    }\n",
       "\n",
       "    .dataframe thead th {\n",
       "        text-align: right;\n",
       "    }\n",
       "</style>\n",
       "<table border=\"1\" class=\"dataframe\">\n",
       "  <thead>\n",
       "    <tr style=\"text-align: right;\">\n",
       "      <th></th>\n",
       "      <th>user_id</th>\n",
       "      <th>client_id</th>\n",
       "      <th>report</th>\n",
       "      <th>test_date</th>\n",
       "      <th>batch_name</th>\n",
       "      <th>trial_name</th>\n",
       "      <th>birthdate</th>\n",
```

```
"      <th>enroll type</th>\n",
"      <th>gender</th>\n",
"      <th>age_at_test</th>\n",
"      <th>SS_avg_score</th>\n",
"      <th>DT_final_score</th>\n",
"      <th>ML_max_score</th>\n",
"      <th>RT_final_score</th>\n",
"      <th>FM_final_score</th>\n",
"      <th>TS_max_score</th>\n",
"    </tr>\n",
"  </thead>\n",
"  <tbody>\n",
"    <tr>\n",
"      <th>0</th>\n",
"      <td>118799</td>\n",
"      <td>NaN</td>\n",
"      <td>152164334</td>\n",
"      <td>2023-02-17</td>\n",
"      <td>bbassessment71</td>\n",
"      <td>brain balance foxvalley</td>\n",
"      <td>NaN</td>\n",
"      <td>NaN</td>\n",
"      <td>Male</td>\n",
"      <td>NaN</td>\n",
"      <td>8.0</td>\n",
"      <td>NaN</td>\n",
"      <td>NaN</td>\n",
"      <td>NaN</td>\n",
"      <td>10.0</td>\n",
"      <td>NaN</td>\n",
"    </tr>\n",
"    <tr>\n",
"      <th>1</th>\n",
"      <td>165444</td>\n",
"      <td>petereggers</td>\n",
"      <td>23530730</td>\n",
"      <td>2019-03-12</td>\n",
"      <td>assessment3</td>\n",
"      <td>brain balance of overland park</td>\n",
"      <td>2011-05-06 00:00:00</td>\n",
"      <td>NaN</td>\n",
"      <td>Male</td>\n",
"      <td>7.0</td>\n",
"      <td>4.5</td>\n",
"      <td>16.0</td>\n",
"      <td>6.0</td>\n",
"      <td>30.0</td>\n",
"      <td>64.0</td>\n",
"      <td>7.0</td>\n",
"    </tr>\n",
"    <tr>\n",
"      <th>2</th>\n",
"      <td>165446</td>\n",
"      <td>sallysample</td>\n",
```

```
"      <td>23528820</td>\n",
"      <td>2019-03-12</td>\n",
"      <td>assessment3</td>\n",
"      <td>brain balance of overland park</td>\n",
"      <td>2006-03-08 00:00:00</td>\n",
"      <td>NaN</td>\n",
"      <td>Female</td>\n",
"      <td>13.0</td>\n",
"      <td>4.4</td>\n",
"      <td>NaN</td>\n",
"      <td>7.0</td>\n",
"      <td>NaN</td>\n",
"      <td>NaN</td>\n",
"      <td>NaN</td>\n",
"    </tr>\n",
"    <tr>\n",
"      <th>3</th>\n",
"      <td>165446</td>\n",
"      <td>sallysample</td>\n",
"      <td>23539134</td>\n",
"      <td>2019-03-12</td>\n",
"      <td>my bbassessment91 4336</td>\n",
"      <td>brain balance of overland park</td>\n",
"      <td>2006-03-08 00:00:00</td>\n",
"      <td>NaN</td>\n",
"      <td>Female</td>\n",
"      <td>13.0</td>\n",
"      <td>NaN</td>\n",
"      <td>NaN</td>\n",
"      <td>NaN</td>\n",
"      <td>NaN</td>\n",
"      <td>NaN</td>\n",
"      <td>NaN</td>\n",
"    </tr>\n",
"    <tr>\n",
"      <th>4</th>\n",
"      <td>165446</td>\n",
"      <td>sallysample</td>\n",
"      <td>23583737</td>\n",
"      <td>2019-03-13</td>\n",
"      <td>my bbassessment91 4336</td>\n",
"      <td>brain balance of overland park</td>\n",
"      <td>2006-03-08 00:00:00</td>\n",
"      <td>NaN</td>\n",
"      <td>Female</td>\n",
"      <td>13.0</td>\n",
"      <td>NaN</td>\n",
"      <td>NaN</td>\n",
"      <td>8.0</td>\n",
"      <td>12.0</td>\n",
"      <td>132.0</td>\n",
"      <td>NaN</td>\n",
"    </tr>\n",
"  </tbody>\n",
```

"</table>\n",
"</div>"
],
"text/plain": [
" user_id   client_id   report test_date
batch_name \\\n",
"0 118799   NaN 152164334 2023-02-17
bbassessment71   \n",
"1 165444 petereggers   23530730 2019-03-12
assessment3   \n",
"2 165446 sallysample   23528820 2019-03-12
assessment3   \n",
"3 165446 sallysample   23539134 2019-03-12 my bbassessment91
4336   \n",
"4 165446 sallysample   23583737 2019-03-13 my bbassessment91
4336   \n",
"\n",
"   trial_name   birthdate enroll
type gender \\\n",
"0   brain balance foxvalley   NaN
NaN   Male   \n",
"1 brain balance of overland park 2011-05-06 00:00:00
NaN   Male   \n",
"2 brain balance of overland park 2006-03-08 00:00:00
NaN Female   \n",
"3 brain balance of overland park 2006-03-08 00:00:00
NaN Female   \n",
"4 brain balance of overland park 2006-03-08 00:00:00
NaN Female   \n",
"\n",
"   age_at_test SS_avg_score DT_final_score ML_max_score
RT_final_score \\\n",
"0   NaN   8.0   NaN   NaN
NaN   \n",
"1   7.0   4.5   16.0   6.0
30.0   \n",
"2   13.0   4.4   NaN   7.0
NaN   \n",
"3   13.0   NaN   NaN   NaN
NaN   \n",
"4   13.0   NaN   NaN   8.0
12.0   \n",
"\n",
"   FM_final_score TS_max_score \n",
"0   10.0   NaN \n",
"1   64.0   7.0 \n",
"2   NaN   NaN \n",
"3   NaN   NaN \n",
"4   132.0   NaN "
]
},
"execution_count": 5,
"metadata": {},
"output_type": "execute_result"

```
    }
   ],
   "source": [
    "df_NoEnroll.info()\n",
    "df_SSmax.head()\n",
    "df.head()"
   ]
  },
  {
   "cell_type": "code",
   "execution_count": 6,
   "metadata": {
    "metadata": {}
   },
   "outputs": [
    {
     "data": {
      "text/plain": [
       "(63646, 18)"
      ]
     },
     "execution_count": 6,
     "metadata": {},
     "output_type": "execute_result"
    }
   ],
   "source": [
    "# df1 = df_SSmax[['user_id', 'test_date', 'gender',
'report','SS_max_score', 'DT_final_score', 'ML_max_score',
'RT_final_score',\n",
    "#        'FM_final_score',
'TS_max_score']].drop_duplicates().dropna(subset=['SS_max_score',
'DT_final_score', 'ML_max_score', 'RT_final_score',\n",
    "#        'FM_final_score', 'TS_max_score'])\n",
    "\n",
    "# df2 = df.drop_duplicates().dropna(subset = ['SS_avg_score',
'DT_final_score', 'ML_max_score', 'RT_final_score',\n",
    "#        'FM_final_score', 'TS_max_score'])\n",
    "\n",
    "df1 = df_SSmax[['user_id', 'test_date', 'gender', 'report',
'SS_max_score', 'DT_final_score', 'ML_max_score', 'RT_final_score',\n",
    "       'FM_final_score', 'TS_max_score']].drop_duplicates()\n",
    "\n",
    "df2 = df.drop_duplicates()\n",
    "\n",
    "df_new = pd.merge(df1, df2, how='inner', left_on=['user_id',
'test_date', 'gender', 'report','DT_final_score', 'ML_max_score',
'RT_final_score',\n",
    "       'FM_final_score', 'TS_max_score'], right_on=['user_id',
'test_date', 'gender', 'report', 'DT_final_score', 'ML_max_score',
'RT_final_score',\n",
    "       'FM_final_score', 'TS_max_score'], indicator=True)\n",
    "\n",
    "df_new.shape"
```

```
   ]
  },
  {
   "cell_type": "code",
   "execution_count": 7,
   "metadata": {
    "metadata": {}
   },
   "outputs": [
    {
     "data": {
      "text/plain": [
       "enroll type\n",
       "Adult                   347\n",
       "Adult CTRL                9\n",
       "Can not identify          8\n",
       "Child CTRL              124\n",
       "Hybrid                  860\n",
       "Initial               15499\n",
       "Test                      8\n",
       "Virtual                 640\n",
       "Virtual New             768\n",
       "Name: user_id, dtype: int64"
      ]
     },
     "execution_count": 7,
     "metadata": {},
     "output_type": "execute_result"
    }
   ],
   "source": [
    "df_comb = pd.merge(df_new.drop(columns='_merge'),
df_NoEnroll[['user_id', 'client_id', 'Status', 'Hours', 'Center']],
how='left', left_on=['user_id', 'client_id'], right_on=['user_id',
'client_id'])\n",
    "df_comb['enroll type'] = df_comb['enroll type'].mask(df_comb['enroll
type'].isna(), df_comb['Status'])\n",
    "df_comb['enroll type'].replace({'In center':'Initial', 'In center
did not enroll':'Child CTRL', 'Virtual did not enroll':'Child CTRL',
'Adult did not enroll':'Adult CTRL'}, inplace=True)\n",
    "df_comb.groupby('enroll type')['user_id'].nunique()"
   ]
  },
  {
   "cell_type": "code",
   "execution_count": 8,
   "metadata": {
    "metadata": {}
   },
   "outputs": [],
   "source": [
    "# get df_comb for adults\n",
```

```
    "adult_wrong_age = df_comb.loc[(df_comb['age_at_test']<18 ) &
df_comb['enroll type'].isin(['Adult', 'Adult CTRL']),
'user_id'].values\n",
    "df_comb = df_comb[~df_comb['user_id'].isin(adult_wrong_age)]"
   ]
  },
  {
   "cell_type": "code",
   "execution_count": 9,
   "metadata": {
    "metadata": {}
   },
   "outputs": [],
   "source": [
    "adult_wrong_age = df_comb.loc[(df_comb['age_at_test']<18 ) &
(df_comb['enroll type']=='Adult'), 'user_id'].values\n",
    "df_comb = df_comb[~df_comb['user_id'].isin(adult_wrong_age)]\n",
    "\n",
    "children_adult_age = df_comb.loc[(df_comb['age_at_test']>=18 ) &
(df_comb['enroll type']!='Adult'), 'user_id'].values\n",
    "df_comb = df_comb[~df_comb['user_id'].isin(children_adult_age)]\n",
    "\n",
    "children_wrong_age = df_comb.loc[(df_comb['age_at_test']<4 ) &
(df_comb['enroll type']!='Adult'), 'user_id'].values\n",
    "df_comb = df_comb[~df_comb['user_id'].isin(children_wrong_age)]"
   ]
  },
  {
   "cell_type": "code",
   "execution_count": 10,
   "metadata": {},
   "outputs": [
    {
     "data": {
      "text/plain": [
       "enroll type\n",
       "Adult                 331\n",
       "Can not identify        5\n",
       "Child CTRL            118\n",
       "Hybrid                834\n",
       "Initial             14976\n",
       "Test                    3\n",
       "Virtual               609\n",
       "Virtual New           745\n",
       "Name: user_id, dtype: int64"
      ]
     },
     "execution_count": 10,
     "metadata": {},
     "output_type": "execute_result"
    }
   ],
   "source": [
    "df_comb.groupby('enroll type')['user_id'].nunique()"
```

```
   ]
  },
  {
   "cell_type": "code",
   "execution_count": 11,
   "metadata": {},
   "outputs": [
    {
     "data": {
      "text/plain": [
       "count    25441.000000\n",
       "mean         9.639794\n",
       "std          3.183889\n",
       "min          4.000000\n",
       "25%          7.000000\n",
       "50%          9.000000\n",
       "75%         12.000000\n",
       "max         17.000000\n",
       "Name: age_at_test, dtype: float64"
      ]
     },
     "execution_count": 11,
     "metadata": {},
     "output_type": "execute_result"
    }
   ],
   "source": [
    "df_comb.loc[df_comb['enroll type'].isin(['Initial', 'Virtual New',
'Virtual']),'age_at_test'].describe()"
   ]
  },
  {
   "cell_type": "code",
   "execution_count": 12,
   "metadata": {},
   "outputs": [
    {
     "data": {
      "text/plain": [
       "gender\n",
       "Female     5250\n",
       "Male      11071\n",
       "Other         9\n",
       "Name: user_id, dtype: int64"
      ]
     },
     "execution_count": 12,
     "metadata": {},
     "output_type": "execute_result"
    }
   ],
   "source": [
    "df_comb[df_comb['enroll type'].isin(['Initial', 'Virtual New',
'Virtual'])].groupby('gender')['user_id'].nunique()"
```

   ]
  },
  {
   "cell_type": "code",
   "execution_count": 14,
   "metadata": {},
   "outputs": [
    {
     "name": "stderr",
     "output_type": "stream",
     "text": [
"/var/folders/qn/v0jt4xsj1y92jrmwls18h_600000gn/T/ipykernel_4753/40801938
60.py:2: SettingWithCopyWarning: \n",
      "A value is trying to be set on a copy of a slice from a
DataFrame.\n",
      "Try using .loc[row_indexer,col_indexer] = value instead\n",
      "\n",
      "See the caveats in the documentation:
https://pandas.pydata.org/pandas-
docs/stable/user_guide/indexing.html#returning-a-view-versus-a-copy\n",
      " demo_df['enroll type'] = demo_df['enroll
type'].replace({'Virtual New':'Virtual'})\n"
     ]
    }
   ],
   "source": [
    "demo_df = df_comb.loc[df_comb['enroll type'].isin(['Initial',
'Virtual New', 'Virtual', 'Child CTRL']),:]\n",
    "demo_df['enroll type'] = demo_df['enroll type'].replace({'Virtual
New':'Virtual'})"
   ]
  },
  {
   "cell_type": "code",
   "execution_count": 15,
   "metadata": {
    "metadata": {}
   },
   "outputs": [],
   "source": [
    "def get_interval(df):\n",
    "   df_sorted = df.sort_values(['user_id',
'test_date']).copy(deep=True)\n",
    "   df_sorted['interval'] = df_sorted['test_date'] -
df_sorted['test_date'].shift(1) \n",
    "
df_sorted.loc[df_sorted.groupby('user_id')['test_date'].idxmin().values,'
interval'] = pd.Timedelta(days=0)\n",
    "   df_sorted['interval'] =
df_sorted['interval'].fillna(pd.Timedelta(days=0))\n",
    "   df_sorted['interval_int'] = df_sorted['interval'].dt.days\n",
    "   cum_interval =
df_sorted.groupby('user_id')['interval_int'].cumsum()\n",

```
    "    df_interval = pd.merge(left=df_sorted, right=cum_interval,
how='left', left_index=True, right_index=True).rename(columns =
{'interval_int_x':'interval_int',
'interval_int_y':'cum_interval_int'})\n",
    "    df_interval['cum_interval_int'] =
df_interval['cum_interval_int'].astype('Int64')\n",
    "    df_interval['rank_interval'] =
df_interval.groupby('user_id')['cum_interval_int'].transform('rank',
method='first')\n",
    "    trt_idx =
df_interval.groupby('user_id')['cum_interval_int'].max()\n",
    "    \n",
    "    return df_interval\n",
    "\n",
    "####################\n",
    "\n",
    "my_ctrl = ['<=30 days apart', '<=60 days apart', '61-89 days apart',
'>=90 days']\n",
    "\n",
    "####################\n",
    "\n",
    "# add an argument for choosing whether to use the max() or min() on
cum_interval_int\n",
    "def get_grps(df):\n",
    "\n",
    "    test_dates = get_interval(df)\n",
    "    test_idx =
test_dates[test_dates['cum_interval_int']>0].groupby('user_id')['cum_inte
rval_int'].min()\n",
    "    test_dates['trt_grp'] = 'only once'\n",
    "
test_dates.loc[test_dates['user_id'].isin(test_idx[test_idx>=90].index),
'trt_grp'] = '>=90 days apart'\n",
    "
test_dates.loc[test_dates['user_id'].isin(test_idx[test_idx<90].index),
'trt_grp'] = '61-89 days apart'\n",
    "\n",
    "\n",
    "
test_dates.loc[test_dates['user_id'].isin(test_idx[test_idx<=60].index),
'trt_grp'] = '<=60 days apart'\n",
    "
test_dates.loc[test_dates['user_id'].isin(test_idx[test_idx<=30].index),
'trt_grp'] = '<=30 days apart'\n",
    "\n",
    "\n",
    "    test_dates['trt_grp'] = pd.Categorical(test_dates['trt_grp'],
\n",
    "                        categories=['only once', '<=30 days apart',
'<=60 days apart', '61-89 days apart', '>=90 days apart'],\n",
    "                        ordered=True)\n",
    "\n",
    "    return test_dates\n",
    "\n",
```

```
    "# problem here is that one user_id can belong to multiple program
(enroll) types\n",
    "\n",
    "def get_one_enroll_type(df, program_type, my_trt_dur):\n",
    "    df_grps = get_grps(df)\n",
    "    my_sub = df_grps.loc[df_grps['trt_grp'].isin(my_trt_dur) &
(df_grps['enroll type'].isin(program_type)),'user_id']\n",
    "    my_enroll_data =
df_grps[df_grps['user_id'].isin(my_sub)].sort_values(['user_id',
'test_date'])\n",
    "    \n",
    "    return my_enroll_data\n",
    "\n",
    "def get_one_enroll_type_all_interval(df, program_type):\n",
    "    my_sub = df.loc[df['enroll type'] == program_type,'user_id']\n",
    "    my_enroll_data =
df[df['user_id'].isin(my_sub)].sort_values(['user_id', 'test_date'])\n",
    "    df_type = get_interval(my_enroll_data)\n",
    "    return df_type\n",
    "\n",
    "def get_2_time_points(df, interval_length):\n",
    "    df_2_time_points = df.copy(deep=True)\n",
    "    df_2_time_points.loc[:,'rank_interval'] =
df_2_time_points.groupby('user_id')['cum_interval_int'].transform('rank',
method='first')\n",
    "    df_2_time_points.loc[:,'rank_interval'] =
df_2_time_points['rank_interval'].where(df_2_time_points['cum_interval_in
t']>= interval_length, pd.NA)\n",
    "    df_2_time_points =
df_2_time_points[df_2_time_points['rank_interval'].isna() |
df_2_time_points.index.isin(df_2_time_points[df_2_time_points['rank_inter
val'].notnull()].groupby('user_id')['rank_interval'].idxmin().values)
]\n",
    "    df_2_time_points.loc[:,'rank_interval'] =
df_2_time_points.groupby('user_id')['cum_interval_int'].transform('rank',
method='first')\n",
    "    df_2_time_points.loc[:,'rank_interval'] =
df_2_time_points['rank_interval'].where(df_2_time_points['cum_interval_in
t']< interval_length, pd.NA)\n",
    "    df_2_time_points =
df_2_time_points[df_2_time_points['rank_interval'].isna() |
df_2_time_points.index.isin(df_2_time_points[df_2_time_points['rank_inter
val'].notnull()].groupby('user_id')['rank_interval'].idxmin().values)
]\n",
    "\n",
    "    return df_2_time_points\n",
    "\n",
    "def make_df_2_time_points(df, enroll_type1, enroll_type2, interval1,
interval2):\n",
    "\n",
    "    df_grp1_2_time_points = get_2_time_points(df[df['enroll
type'].isin(enroll_type1)], interval1)\n",
    "\n",
```

```
    "    df_grp2_2_time_points = get_2_time_points(df[df['enroll
type'].isin(enroll_type2) &
(~df['user_id'].isin(df_grp1_2_time_points['user_id'].unique()))],
interval2)\n",
    "    df_2 = pd.concat([df_grp1_2_time_points,
df_grp2_2_time_points])\n",
    "    df_2['time_point'] =
df_2['cum_interval_int'].mask(df_2['cum_interval_int'] != 0, 1)\n",
    "    df_2 = df_2.drop(columns=['interval', 'interval_int',
'rank_interval', 'batch_name', 'report','client_id', 'trial_name',
'age_at_test'])\n",
    "    return df_2\n",
    "\n",
    "def make_jitterplot(df, program_type):\n",
    "    zscore = lambda x: (x - np.nanmean(x)) / np.nanstd(x)\n",
    "    df_sns = df.copy(deep=True)\n",
    "    df_sns[['RT_final_score', 'SS_avg_score',
'DT_final_score','FM_final_score', 'TS_max_score', 'ML_max_score']] =
df_sns[['RT_final_score', 'SS_avg_score',
'DT_final_score','FM_final_score', 'TS_max_score',
'ML_max_score']].apply(zscore)\n",
    "    df_sns = df_sns.drop(columns = ['birthdate', 'gender',
'opp_num', 'cum_interval_int']).melt(id_vars =['user_id', 'time_point',
'trt_grp'],var_name='test_name', value_name='score')\n",
    "    df_sns['time_point'] =
df_sns['time_point'].astype('string').replace({'0':'pre','1':'post'})\n",
    "    g = sns.catplot(data=df_sns, x='time_point', y='score',
hue=\"trt_grp\", col=\"test_name\", aspect=.5, alpha=.20)\n",
    "    g.set_titles(\"{col_name}\")\n",
    "    g.set_axis_labels('', '')\n",
    "    num_ctrl = df_sns.loc[df_sns['trt_grp']== '30-90 days',
'user_id'].nunique()\n",
    "    num_BB = df_sns.loc[df_sns['trt_grp']== '90 days or more',
'user_id'].nunique()\n",
    "    g.fig.suptitle(f'{program_type} Program, {num_ctrl} in CTRL,
{num_BB} in BB', y=1.06)\n",
    "    g.legend.remove()\n",
    "    g.fig.legend(handles=g.legend.legendHandles, loc=7)\n",
    "\n",
    "# remove outliers that are 4 standard deviations and beyond for the
first time point \n",
    "def outlier_removal(df, my_score_names):\n",
    "  \n",
    "    my_stats = df[my_score_names].describe()\n",
    "    outliers = (\n",
    "        abs(df[my_score_names] - my_stats.loc[\"mean\", :]) > 4 *
my_stats.loc[\"std\", :]\n",
    "    )\n",
    "    return df[my_score_names].mask(outliers,np.nan)\n",
    "\n",
    "def get_test_df(df, score_name):\n",
    "    df_one_test = df[['user_id', score_name, 'trt_grp',
'time_point', 'cum_interval_int']].copy(deep=True)\n",
```

```
    "    df_one_test = df_one_test.pivot(index=['user_id',  'trt_grp'],
columns=['time_point'], values=['cum_interval_int', score_name])
#.dropna()\n",
    "    df_one_test = df_one_test.astype('float')\n",
    "    df_one_test['diff_score'] =
df_one_test.xs((score_name,1),axis=1) -
df_one_test.xs((score_name,0),axis=1)\n",
    "    df_one_test['baseline_score'] =
df_one_test.xs((score_name,0),axis=1)\n",
    "    df_one_test = df_one_test.reset_index()\n",
    "    #drop(columns=['cum_interval_int', score_name, 'user_id'])\n",
    "    return df_one_test\n",
    "\n",
    "zscore = lambda x: (x - np.nanmean(x)) / np.nanstd(x)\n",
    "test_score_names = ['SS_max_score', 'SS_avg_score','DT_final_score',
'ML_max_score', 'RT_final_score', 'FM_final_score', 'TS_max_score']\n"
   ]
  },
  {
   "cell_type": "code",
   "execution_count": 8,
   "metadata": {
    "metadata": {}
   },
   "outputs": [],
   "source": [
    "def get_test_df(df, score_name):\n",
    "    df_one_test = df[['user_id', score_name, 'trt_grp',
'time_point', 'cum_interval_int']].copy(deep=True)\n",
    "    df_one_test = df_one_test.pivot(index=['user_id',  'trt_grp'],
columns=['time_point'], values=['cum_interval_int', score_name])
#.dropna()\n",
    "    df_one_test = df_one_test.astype('float')\n",
    "    df_one_test['diff_score'] =
df_one_test.xs((score_name,1),axis=1) -
df_one_test.xs((score_name,0),axis=1)\n",
    "    df_one_test['baseline_score'] =
df_one_test.xs((score_name,0),axis=1)\n",
    "    df_one_test = df_one_test.reset_index()\n",
    "    #drop(columns=['cum_interval_int', score_name, 'user_id'])\n",
    "    return df_one_test\n",
    "\n",
    "def run_ancova(test_df):\n",
    "    model = ols('diff_score ~ trt_grp + baseline_score',
data=test_df).fit()\n",
    "    return model\n",
    "\n",
    "def run_ind_ttest(df_ttest, score_name, ctrl_grp, trt_grp):\n",
    "    res = stats.ttest_ind(\n",
    "        df_ttest.loc[df_ttest['trt_grp'] == ctrl_grp, score_name],
df_ttest.loc[df_ttest['trt_grp']==trt_grp, score_name], equal_var=False,
nan_policy='omit'\n",
    "    )\n",
    "    return res\n",
```

```
    "\n",
    "def run_ind_ttest_two_tail(df_ttest, score_name, ctrl_grp,
trt_grp):\n",
    "    res = stats.ttest_ind(\n",
    "        df_ttest.loc[df_ttest['trt_grp'] == ctrl_grp, score_name],
df_ttest.loc[df_ttest['trt_grp']==trt_grp, score_name], equal_var=False,
nan_policy='omit', alternative='two-sided'\n",
    "    )\n",
    "    return res\n",
    "    \n",
    "def run_paired_ttest(df_ttest, score_name, ctrl_grp, trt_grp):\n",
    "    res_trt = stats.ttest_rel(\n",
    "                df_ttest.loc[df_ttest['trt_grp'] ==trt_grp,
(score_name,0)], df_ttest.loc[df_ttest['trt_grp'] ==trt_grp,
(score_name,1)], nan_policy=\"omit\", alternative='less'\n",
    "            )\n",
    "    res_ctrl = stats.ttest_rel(\n",
    "                df_ttest.loc[df_ttest['trt_grp'] == ctrl_grp,
(score_name,0)], df_ttest.loc[df_ttest['trt_grp'] == ctrl_grp,
(score_name,1)], nan_policy=\"omit\", alternative='less'\n",
    "            )\n",
    "    return res_ctrl, res_trt\n",
    "\n",
    "def cohend(d1, d2):\n",
    " # calculate the size of samples\n",
    " n1, n2 = len(d1), len(d2)\n",
    " # calculate the variance of the samples\n",
    " s1, s2 = var(d1, ddof=1), var(d2, ddof=1)\n",
    " # calculate the pooled standard deviation\n",
    " s = sqrt(((n1 - 1) * s1 + (n2 - 1) * s2) / (n1 + n2 - 2))\n",
    " # calculate the means of the samples\n",
    " u1, u2 = mean(d1), mean(d2)\n",
    " # calculate the effect size\n",
    " return (u1 - u2) / s"
   ]
  },
  {
   "cell_type": "code",
   "execution_count": 16,
   "metadata": {
    "metadata": {}
   },
   "outputs": [],
   "source": [
    "df_comb[test_score_names] =
outlier_removal(df_comb[test_score_names], test_score_names)"
   ]
  },
  {
   "cell_type": "code",
   "execution_count": 17,
   "metadata": {
    "metadata": {}
   },
```

```
   "outputs": [
    {
     "data": {
      "text/plain": [
       "enroll type  trt_grp           time_point\n",
       "Child CTRL   only once         0               0\n",
       "                                1               0\n",
       "             <=30 days apart  0             118\n",
       "                                1             118\n",
       "             >=90 days apart  0               0\n",
       "                                1               0\n",
       "Virtual      only once         0               0\n",
       "                                1               0\n",
       "             <=30 days apart  0              10\n",
       "                                1              10\n",
       "             >=90 days apart  0             141\n",
       "                                1             141\n",
       "Virtual New  only once         0               0\n",
       "                                1               0\n",
       "             <=30 days apart  0              65\n",
       "                                1              65\n",
       "             >=90 days apart  0             169\n",
       "                                1             169\n",
       "Name: user_id, dtype: int64"
      ]
     },
     "execution_count": 17,
     "metadata": {},
     "output_type": "execute_result"
    }
   ],
   "source": [
    "my_ctrl = ['<=30 days apart', '<=60 days apart', '61-89 days apart',
'>=90 days apart']\n",
    "bb_virtual = get_one_enroll_type(df_comb, ['Virtual New',
'Virtual'], ['<=30 days apart', '>=90 days apart'])\n",
    "ctrl_virtual = get_one_enroll_type(df_comb, ['Child CTRL'],
my_ctrl)\n",
    "df_virtual = pd.concat([bb_virtual, ctrl_virtual])\n",
    "df_virtual_2_time_points = make_df_2_time_points(df_virtual,
['Virtual New', 'Child CTRL', 'Virtual'], ['Virtual New', 'Virtual'], 1,
90).drop_duplicates()\n",
    "#df_virtual_2_time_points.groupby(['enroll type', 'trt_grp',
'time_point'])['user_id'].nunique()\n",
    "df_virtual_2_time_points['trt_grp'].replace({'<=60 days apart':'<=30
days apart', '61-89 days apart':'<=30 days apart'}, inplace=True)\n",
    "df_virtual_2_time_points.loc[(df_virtual_2_time_points['enroll
type']=='Child CTRL') & (df_virtual_2_time_points['trt_grp']=='>=90 days
apart'), 'trt_grp'] = '<=30 days apart'\n",
    "df_virtual_2_time_points.groupby(['enroll type', 'trt_grp',
'time_point'])['user_id'].nunique()"
   ]
  },
  {
```

```
    "cell_type": "code",
    "execution_count": 18,
    "metadata": {
     "metadata": {}
    },
    "outputs": [
     {
      "data": {
       "text/plain": [
        "TtestResult(statistic=-0.6521801779063109,
pvalue=0.514653166038064, df=406.91554534658695)"
       ]
      },
      "execution_count": 18,
      "metadata": {},
      "output_type": "execute_result"
     }
    ],
    "source": [
     "def demo_ttest(df_ttest, score_name, ctrl_grp, trt_grp):\n",
     "\n",
     "    res = stats.ttest_ind(\n",
     "                df_ttest.loc[df_ttest['trt_grp'] == ctrl_grp,
score_name], df_ttest.loc[df_ttest['trt_grp'] == trt_grp, score_name],
nan_policy=\"omit\", equal_var=False,\n",
     "            )\n",
     "    return res\n",
     "\n",
     "\n",
     "df_virtual_2_time_points['age_at'] =
pd.to_datetime(df_virtual_2_time_points['test_date']) -
pd.to_datetime(df_virtual_2_time_points['birthdate'])\n",
     "df_virtual_2_time_points['age_at'] =
df_virtual_2_time_points['age_at'].dt.days\n",
     "\n",
     "demo_virtual =
df_virtual_2_time_points[df_virtual_2_time_points['time_point']==0]\n",
     "\n",
     "demo_ttest(demo_virtual, 'age_at', '<=30 days apart', '>=90 days
apart')"
    ]
   },
   {
    "cell_type": "code",
    "execution_count": 19,
    "metadata": {
     "metadata": {}
    },
    "outputs": [
     {
      "data": {
       "text/plain": [
        "Text(0.5, 1.06, 'At-home program')"
       ]
```

},
"execution_count": 19,
"metadata": {},
"output_type": "execute_result"
},
{
"data": {
"image/png":
"iVBORw0KGgoAAAANSUhEUgAAAjMAAAAJ+CAYAAABGlIFwAAAAOXRFWHRTb2Z0d2FyZQBNYXRw
bG90bGliIHZlcnNpb24zLjQuMywgaHR0cHM6Ly9tYXRwbG90bGliLm9yZy/MnkTPAAAACXBIW
XMAAA9hAAAPYQGoP6dpAABo7ElEQVR4nO3deVxVdeL/8fddBERFkQDTLAlDcyE3irRxN9NyJq
Kmcfspo41lbpNGljVJpdZUpmVmCTbWVJppln4Ptrn9bJaBQtzS2z1EpHMUQJZYl7z+8P4+ZNVM6
9l+XA6/l4+FDOOZ/lfLgffHNWm2EYhgAAACzKXtUdAAAA8AdhBgAAWBphBgAAWBphBgAAWBphBgAAWBph
BgAAWBphBgAAWBphBgAAWBphBgAAWBphBgAAWBphBjiL0aNHq1WrVrVrrttvOuE1+fr5eeeWVc
tX3448/qlWrVho7dmyguggAtR5hBjiDw4cP67PPPPlNoaKg++eQTZWdnl7ndoNddcoyVLllRy7w
AApQgzwBmsWrVKLpdLo0aNUklJiV5//fUytzt8+HAl9wwAcCrCDHAGK1euVr19
fr732mngvKwBUP4QZoAw7d+7U7+7U119/rcTERNWtWtW1e9e9e/fW999/r//+97+ebf773/+qVatWnu1b
tWqlp556tqtxtfPzxx/rzn/+s+Ph4XXnllZo6dapycnO2+6LL77Qrbs6cbfeqoSEBLVv316DBg3So
kWLVFJS4rVd7969l96NXLkSG3fvl0jR45Ux44ddcUVN9gf//iH//ijE4Ol0OPN9/66LL77Qrb
feqoSEBLVv316DBg3SokWLVLmDBg3S9okWU5VFJS4rVd79639NXLkSG3fvl0jR45Ux44ddcUVN9gf//iH
lTpujIkSOntZeZmamUlBR17TxYN
nzho5cqQ2btxY5rbr1q3TDTfcoHbt2ql///46fvy4JGn37t36+9//riuvvNKzbs6cOTpx4sRp
7W7btk1jxozRFVdcoc6dO+vvf/+Dh06pDZt2mjq1kMe7YYPH67evXvXrgw8+UI8ePx
sSJnvWvv/66hg8froSEBLVr105XXXVJk+erO+//75CxhpAYBBmdKsXLlSkjRw4EnXXXS
dJWrZsmWebZs2aady4cWP6d3qdZ+ka3ogpGZaaalx+m4u97+zYn8hgBvK3ZPdNGHBSafg
o0aO9jv689dZGjp0qDIzM9W9e3e3fdfPPNKikp0SOPPKKxY8fK5J51fvjjz9q9q
/qLIyEgtXbpUd911llwYPHqwDBw7oz3/+sy66KCKtWrVK9913n1f5Zcu1mUlRbt27dLAgQ
7/8RTk5OZo4aIWLFhQ7vG75557tGrV1/bV4NRa+/99Xr+/KmbDsVm1j1OIxd8ssNu7TRbZxrrrrr
d77vPgAhqlevnp555MVWQBWNntRyxstJTJ4ooAMuvCMtF2BpWzXtt+5nRG9OzLK9f2SsofwL
hqoqCitXr1aw4YNU2FhYDHGkAJGQC8lJJAQJcZJxN8aE5BTiP5sfnbt8LMUU9lCQNnkuhKjeGFkYnDdjG+ZvG3xt+F
ejHdBWj3MQ00r+5dm9Ou3QZ9tg+bFhtyWXnpg3uRfSDGNaBhcZZQ21oeEL+D0ubLYz4ufaI+Dh3I2vzMBb
rbvbQQkbrxdJ69O2C1m7w12tZnxxgYwNP8tIzuJxXNfFaPxWlCmdafB0Z8RxgX1QGfsfJLFvKcAsXWv
YMDNUwMJXPxkmrk+WL0Mp5M6ep8dtV7QGqHUZg2sXmI2rK9mS+NUyr+aljg4qQ5VGmF3ErmsJUB7+kABvLK
dmvKCDQGbzpjiNhC4LPVO0B9Ln8GvnwxG2U7F2WePyZr4s8tJm4D7dTXVGP/Ysyd5b8sDLaXWQOW9lB
tSDO8NzswTUmpdB0cxZPMJHlgT+8zYqHxN1VdfqSWa8Kc6KOp2Lsy6eWp/Ww35M8DB/d3mdEUT7qahV6
ZbTFoMzYgXhj48gnTKWDAXUSV0J84GPJRXSk4YgT3uyGNpz6ljNw6ppj5ojp5B5OcGJ13bjdaLfoLrIjk
BTJ0C4GSJbNfbf9Gt/fzbKVTpoWMoNBUdu1+PJPAD1d1L4PYqvFmTeiWJnsWG5GSBN9gN6VHVN6p3ha2r
5a/S+vPyb0wbxEy2Ji7AJGjq87qWzLAcv2Awv2e2wI/MHRUkW5ZOGtxoODhhkd4oRwoOuyeaDHa1xJk
kKa5KW9Y/yW3BJA5HmbPeLYvwZPTvNhK2MBvf3kAHrC3yESDgOZ9tSHe8WyAMMlYe3Iw4wwxh4FbJx
eIwX82kJpP8bgbMu1SWX6pXMfg3AOS1mbYNwehFPBN3KLY5MPV2p2KJ3R+uo2RR1QOfQnD5dsZYzz/Zs
Bq5uXHk/ymAt9xZ5SOnNBp4mUvVcuNTBPeEh5jNDOwWBBqyKYr7ndPlVzpERXXSfNZuUXYEQ8KBdgF
eUHtIvzWVfYjMGjnK6e7oLI7dahI3BQ4ZTddKXO7u/yB0M9xqPtd5Zez+0tLRPffTxH4dqwQ7lNj1e
WmFBuUOVyxm0CHGFvbXNM5ouLN67BFvj/q6cWdb4YJlxiGS87Zs2lQHNJCP8YqDp2IFG+19NvY6L8
o
PHxS64nXdFeb6FSBK8kCMoiXGGxz4YG48ZxEaxGFMGJh7dW1ZzUEIjX9QrlyGCB0bPKt3c
mbl792rp0qVKSEhQ7969z1nXli1b1K5dO69lX3zhSdMQSWOnW/R4wY4bWuuLhYW7ZsUUREh

MLDwz3XomzZskU33njjaX0or9WrV8vtdmv69Onq2bOn17rdu3dLEs8YAqoxLgAGfrV//35t2L
BBzZs3LzPISCcv3E1ISNDx48f19ttvSzp5gefvn/kSCH369FH9+vX1yiuveF2rUlxcrAcfffFA
ul0vXX399wNr74x//KIfDoTlz5uinn37yLC8pKdGDDz6oRYsWlftZKYsWLfI6srJ582a98cYb
at269TkDWKdOndS8eXOtWbPG64iI2+3WP//5Tx09elR//OMfZbfb1bFjR1188cV68803tW3bN
s+2eXl5mjt3bnl33XN069T9lk4+c2fVqlWSVCHfYwCBwZEZ4FdvvPGGDMM47cLf30tOTb69e
u1bNkyJScnKzo6Wt99953S0tL0hz/8oVxHLsqjQYMGeuihhzR58mTdfPPN6tu3r8PDw7Vu3Tp
99913t69u4YMGRKQtqSTFyWnpqZq1qxZuvbaa9WnTx+FhYXpk08+0bfffqvu3bt3YF1Nj//
/LOSkpLUr18/5efna82aNQoODtZDDz1zrIOh0MPP/ywRo8erb/97W/q3bu3mjVrpg0bNmjbt
m1q06aNJkyYIEmy2Wx64EH1JKSoiFDhujqq69WgwYN9OgyH9qqoECSvO6UOpOBAwfq+eefV1
pamjZs2KDIyEh9/fXX+vTTTxUeHq6cnBwdPXq0XPs/PPsOoPJxZAb4Vekt3PQ/ekt14e9e9evX
0xRdfaPfu3frHP/6hZs2aadymyYKDgzVt2jQrrr/9V7u84I2t8o
WLDgtNuI/TVy5Eg999xzat26tdasWa0lS5fK4XDorrvu0rx581SnTp1y1fOPf/xDffv21f/93
//pP//5j3r16qWlS5eqffv25SrfpUsXLVu2TFdffc8svq6CgQOPHj9crr7zidXovS/FI999FxzateundauXatXX31/VP/30kyZ
MmKA33nhDdrtdH3/8cbn6DqDy2QxOBAMIkKeeeeeekrz5s1Mlh8VcnVWH3aoOoraKhbMKhIhw8f1vnnnn39a9/
FBL7zwgjp06KC1a9fqjTfeUOfOnT1HZurWrXvO9i699/FI999xzateundauXatXX31VP/30kyZ
MmKA33nhDdrtdH3/8cbn6DqDy2QxOBAMIkKeeekrz5s3T008/fdp1PhWhqKhIw8f1vnnnn39a
sPv88881YsQITZkyRbfcckuF9wVA1eHIDADLON78uPr06ZQaOAW9thjj6
ljx45auKli5dDADD+jPf/5zFfcOQOQXgmhkAAGBpXDMDAAAsjTADAAAsjTADAAAsjTADAAAsjTADAAAsjTADAAAsjTADAAAsjTADAAAsjTADAAAs
jTADAAAsjTADAAAsjTADAAAsjTADAAAsjTADAAAsjTADAAAsjTADAAAsjTADAAAsjTADAAAsj
TADAAAsjTADAAAsjTADAAAsjTADAAAsjTADAAAsjTADAAAsjTADAAAsjTADAAAsjTADAAAsjT
ADAAAsjTADAAAszVnVHYA1fPjhh1qxYoWbdum7Oxs1atXT9e3bt9eW3Uu3dvz3ZPPfWU5s2s2
bV646x40bp/Hjx2vq1Kl6/fXT1tvtsv9lUv359NWvWTP3799fo0aMuX9V++8zXBHuCCtx79e77//
vi644AL/dxKz8qaxazdbT2bNlSrVq00fPhw9evXz7OudP6eyuFwKXwsTPHHx8brllluUkJBQK
X2H9RFmcFb5+mGw8VL+m6evFa/dwtt956edi/f6neH0dSE9Vz6++GuPP3d7fZUUGw+XuDuY79GdwvhHkdbvvb
SCNb1+8fcq/+9XZdXB1Dw+s77V7Vhr+DFl4UOgHb5Bz2rqioAc2o7tf4
1a5/X/8/t26RTNiBx54QA6Hw4rkNFc2bK+Q8tJR9hbcxb+X6Q1ARtJE8P6WRW+ii0x2XW8YS13zedPdnMSb9/u3e
vhlprfh0nSgrrKpLQvXaLCvq5//yKeQE1Qe0+2tB4LY9h2v7v+nY0i+G/YEBbGP4+edR2Y3p+YMAvFr9i+vGOHoHi2Jr2Vy2Gt65
33Y1h/D8s9ChASCdGP5Xs1yjoH9/D7xr8ttOsbgRtmB5DbOKHf8kt8b9c8cTw89cuXNOxIq+Wyr
X2H9RFmcFb5+fm65557tGbNGrVt21Y33HCfDoqKidPDgQa1cuVK33Xab/vrXv+quu+6SJAUFBenRRx/VqVOn9Oqrr9r3Cx+8++bz
8XXnihVx2zZs1Sbm6u/vnPf3otb9WqldfXt956qy6++GGH//LiOMNzZ07V99++60Grr+8+60
ef/zxCtpTAIFw6juKSnRkSNH9Pbbb2vcuHHuOaOkpvba/++++FR4eLkkbqLi7Wz/9pNdf
f10jRoxQenq6unbtWun7Awygsyg+LOYOHGiERcxdnvPrqq2esplevX
kZcXNwZ1991111GXFyc8fnbnn5e5e+vrCw0BgwYIARYhMkYCBqAhm8cnTp
wwunfvbnTt2tVwu92GYZx9mZ90m9gy/mZnZxvx9mZnZxvxvfvr1GjRp2
2PigoSDNnzpTD4dALyxZK7xQYf0IDg7Wn/70l8JhyL0
RkaqVatW2rVrVyX0rlDVyLK9CQ0NrdD3SAVHtZr0VPKTt9
Y0aNVKjRo3Oua3L5dL8/e3bXWbxZ9Yyu8L2vXrpUktW
vXrsLbAuAuC7n3/+2XP0Pz6Hzc3Bzx54QA6Hw4rkNFc2bK+Q8tJ
To4ceeqjS+w9rIszgzgjLKzs9WoWoHPHQL7/8sj799FOlpKSo9/r3ZH4JDS+qRfdmtMUOaOg
APDN7bffXubyfv36eU4XXRFo35Pz+RFmNBfz1VRxe3bBmv+5h8elsq/9WkP4XTrHwKNzQmo5nfEN
aNGacKECZXWFuCuUt9W6dWtJe6SgHGwH8fQOZXlaePGjj6dfVJ9r3NuOjhTQWbRfUy+BhHprbK
6TdPLIzNGjR7Vu3Tq99NJLLL2ryf4IUMAVNHfsQAKKhj9qwL2v1YVX9Kg2+
owIEDWrRokbKzszVzt2jT98Y9/rPD9sXPDffAndUiuDCQnTwHdiuCS7a2O3yKBcrxCOBQQGDj
XadOnU57TtSgQYYYUMH6uRYsWJGdnqOt2aW69HGbbBIhNyWzYtMGPW2o1Jh0Fq2
5Sdne1Xe23btlXXrl3VXXp+Vs33XTVq2bJmaNWmMO++8UYS1fdQWn6zu3wJ5fdLE5W7LDm
zbo3YjzOCMrrvuOknSyy+/+/fMZt/ve//+nNN9/VnTvVmKtTVP2fVNvY7tlMvTJhn7WfJ
BrR9A5XG73ZJ02gXAgoetRhBmeWkJCgf36v6ac2a0Xr+wyZY1iAnU4uHwJdmjkT
ZGyNSFF16o++67z/t6y6KuiakMFbfWqd1UdbBCpq+8NtWVTXbIfV0JBH4i
atW6eqrr1bjxo21pxo5Iu56SbQI6CIU6mRlt3uhxkLRgVfdcXmcSpDxrxZEHI27NA5ZG9lx
Xq1TutNN26dXX33XdXWL8AnOz7zzTfzzJ8TWdZsJjG22bmjf/ey2dnSpSaBvbxQl87tBenoDiE4tYNvxOVTEyxGV9pe
FBQX6mDe4kHhy7ufGqmrGaoEBD9XjAhrMgrbPRN0Nwd0ElF26+EI9nmDGlD1LmcC8MNNXe+y+XTX+N9zdprO6VEEBdXlKy4
oUOHqnv37hXEjweEcCbNm3SsmXLdXetsqUmnLWpDRlaFwqwqBAhtPDFl9SsuKUmR+cXm5aM6ZcH+kWqazbRDk+dKyj9TRzYnHR
rSphg0bpltuucUrugEgn391WymazqW7durrwwgs1duxRo1qtIeDQFrsxmGYVR1JwAAAHzFNT
MAAMDSCDMAAMDSCDMAAMDSCDMAAMDSCDMAAMDSCDMAAMDSCDMAAMDSCDMAAMDSqv1D81wut44cqdhH2Nv
tNjVuXE9xHjhyX281jd8qLcTOvtoxZZGSDgNTD/K+eGDPf1JZxC9T8N4MjMzr5AbPZbLLbXd
FUth3MxjzKofvifmMWa+YdwqDmEGAABYGmGAABYGmGAABYGmGAABYGmGAABYGmGAABYGmGAABYGmGAABYGmGAABYGmG
mEGAABYGmEGAABYGmEGAABYGmEGAABYWrV/nQEAAMDSCDMAAMDSCDMAAMDSCDMAAMDSCDMAAMDSCDMAAMDSCDMAAMDSCDMAAMD
Dd5XbraO4JAg0sjzADABZlt9vksNv9js7lH3khKmyUY1DNeSaS2W32wgzsDzCDABYbRE9p

/OL+quwFUGS4ABgAAlkaYAQAAlkaYAQAAlkaYAQAAlkaYAQAAluZTmFm5cqUGDhyo9u3b69pr
r9Xbb7/tWbdjxw4NGzZMHTp0UM+ePZWRkRGwzgIAAPye6TDzxhtv6J577tHNN9+s1atXa+DAg
brjjjv0xRdfKDc3VykpKWrRooWWL1+u8ePHa+7cuVq+fHlF9B0AAMDcc2YMw9DcuXM1YsQIjR
gxQpJ0++23a9OmTVq/fr3Wr1+voKAgTZ8+XU6nU7U7Gxsdq3b58WLlyo5OTkCtkBAABQu5k6MvP
dd99p//79GjRokNfyjIwMjRkzRllZWUpISJDT+VtGSkxM1J49e5STkxOYHgMAAJzCVJjZu3ev
JOnEiRMaNWqsrrzySt1000364IMPJEkHDx5UkyZNvMpMpERUVJkg4cOBCA7gIAAHgzdZopP//k4
7LvuusujRs3TlOmTNGaNWs0duxYPf/88yosLFRQUJBXmeDgYElSUVGR7510VuxNVw6H3etvlA
/jZh5jZh7z/8xK+2yz2WSzmXtzdun2vuy3lcesKjFuFcdUmKlTp44kadSoUUpXKSpIkXXrppdq
+fbuef/55hYSEqLi42KtMaYgJDQ31qYN2u03h4fx4fV8KmtWWFjdSmmnpmHczGPMyof5Xz4Oh11O
p8N0Gcm//bbymFUlxi3wTIWZ0lNICXFxXstbtbtmypjz76SM2aNVN2drbXutKvGzdu7FMH3W5D
8/c22DNcjjsCgurq7y8Arlc7gptqyZh3MyrLWMWqADC/D+70r67XG6VlLhMlS3dV1/228pjVp
Vqy7hV1i8gpzIVZtq0aaN69epp8+bN6tKli2f5rl27dOGFF6pTp05asmSJXC6XHIzcz/pTp05asmSJXC6XHI6TvyVkZmY
qJiZGERERPneypKRyvuknfyDU3YRWHczGPMyo/5f26GYcgwDNNlJP/228pjVpUYt8AzdeIu
JCREo0eP1tNPP63Vq1fr+++/1zPPPPKN169YpJSVFycJys/P17Rp07tWLFCixcv1pgxY
yqq/wAAoJYzdWRGksaOHau6devqSee0KFDhxQbG6unnpKV1xxhSQpPT1dM2bMUFJSkiIjI5
Wamuq5vgYAACDQTIcZSUpJSVFKSkqZ6+Lj47Vs6qu7V06VK/OgUAAFBe3B8GAAAsjTADAAAAs
sjTADAAAsjTADAAAsjTADAAAsjTADAAAsjTADAAAsjTADAAAsjTADAAAsjTADAAAsjTADAAAs
jTADAAAsjTADAAAsjTADAAAsjTADAAAsjTADAAAsjTADAAAsjTADAAAsjTADAAAsjTADAAAsj
TADAAAsjTADAAAszXSY2b9/v1q1anlanXanXan2XLlkmSduzYoWHDhqlDhw7q7q2bOnMpj
6zBb7++msFBwdr7dq1stlsnuUNGjRQbm6uUlJS1LdvX6XX6X6WlpeaLL79UWlqaGjVqpOTk5IB2HAA
AQPIhzOzatSUsxMTGKioo6bd3ixYtlYsVFBSk6dOny+l0KjY2Vvr24dPChqsJMwAAoEKPYs309ddf
q2XllmWuy8jIUEUyKdyk6dOny+l0KjY2LpPEy27a+2vNhJNJRQ96uUlfc0ZZHyN0mYNVXrZn/Dx+
3aaUP4VvAvIdHsUzCCBSMYkNBr
3zx3M82gphrFo0KOZAAEeZLnhX5RJW2VUqUhf2WzBZhZYpPXbCfn2Ma/9V2t/tfTnbCk1kCUs
/T/uVRur0v+11apk4dh8/j5nYbMgzDp7JWZeXPWnVnKswUFxdr7969Yb/pyMTUxBuZWbzLYsZK8KR
Tt9xyi55//nkVFhYqKCjIq0sWOaWJDvUq7JcsSXWXiFhdSu1nZqGcTOPMSSf5n/5OBx1zOz6ZZm+
OBx2OZ0Uk3fb3ITqvYLy9vt5gJYT
WHlzdlp1ZSrMBBAUFacOGDXI6I6nZ7Q0q5d03377
bfKyMhQSEiIouLvcqUhpjQ0FUzO0CfJyKc5SWcm3G3c+dOdezdy
aoAML8P7vSvrtcbpWUuEyVLd1XX/a7Th2H6tcP0Strdir7iPnvT1TjUA3u39qSY+4PK3/WzKi
sX0BOZfo0U1mhJC4uTp9++qaNGmi7Oxsr+3W1X0dHR/vYRamkpHK+6SdH6sd/INTcD1hFYdzMY8zK
j/l/boZh/pRN6fa+7HfpUZ3sIyf0Y/bPpsr623ZNUFv3uyKZOnl4YLb3dq
pYtWyohIUEbN26Uy/UqcmH6ZAUaEzmZmKqVgoHn8XX0abbbzVr1i
x9+eWXuvXWZ5WcnKz8oVK7R48WKNGavPADBqQVOnmex2uxYsWKDHHlnt
MkyZNUl5entgaaPnn39erVrayRxkKUmRkpFTU5WUlQhnQcAANZRXFysgoIC
NWzYUJL0ww8/aOnPmJiois5s2bm67X9DUzjRs31syZM8+4Pj4+4Pj4+8uEEtZQQAAU
HPt3LlTI0aMv333qv+fvroYce0vL/dP311+vBBx/XMzRMwMuIMwAAAEAI1MA0
q0Uffu3fXEXo1apVmjq6++mot1ATvvvPPKP0z2crIiJCx4fCCXfdPLkHAABUum2
bN2vEiBFq2LChVq1apXHjjz/nA8CGKjo5WnTp1FB0FdPQMRJjChAlatWvWqvboMwAAoMIVFBR4
Hu+Sn5+v+v1xL+1l69v8zx5GHHPYJNL/LndtzbvYVhs33m9yXH6tS6//JHTd
XPNDAAAqHCDBw/WrFmzVGfWWEmw3W+YxfAm+KjR4qhRde0LFjxT788Hb0wFyxz1lPTs7ARFTTq
QZAABQ4YYMGaR4/qhRdeoLFjxyoo+YMGlSeVN9WHaHE3ZUTkEzLS0MPTtp06daquv+6HNwC9nDH
2rPLy8nTixInWxzn8EQKEapd2C5n/tHeqaNTnTF4uQkljRHjQGmFghsd4Ruaf8rzQxa5tiEVH/t3fdV/UzRpoOmvsxqLbDP//MWVq5hQTFDtVxBaP
e/Zsbd++XYZhG63Kz4+XPmnT1ZCQqOkpuribCQAAVKrPP/9/cY8aMUWRkpEaOHCmbabro0erO
KBAKSkpp73Q+lwIMwAAoFI9/fTuuaaa7RgwyFPu3J/ty5puojzA
AAgEq1detWzRo0ZpYMfIMvqpPVdffHfWWqPsIMAACoVHXCXQxpJHL5Spz3dGjRxUcGHGyqPsIMAACoVG3
bttWiRYvmL2AVFVkTkbvbt26eee7e26e5c+eqV69e6epojzAAAgEo1Ycl3Gcxw4saXPIUh25qQ9J4r
DlS9evWUmppSkvpDpSYRvuFF7Vt2zjPkAF9DtsCY2ZT
wAAKl18fLzi4+LzuLZFVVPbvp3Nb6bsTVoIrxkl2yjqPWmbDuh1kEakCW5dTg4b9WrvHcq
F15HYbCgurW9VdQS10zz33lLncMAzZbDZ7E2cDAKg7EKCnbLbbXplzU4dyjjluuhwT
jy4ohuMtO+fc4w0QSKBL/3bBkHJ
xmypDmAEAVRJ0oOALvxL2Fs6KRHDsgr00rLrTiLXTNpw7F8uYs
ysDmeQQSaU3czTZnYzyyytzjaU3q1+a0uG6c+E9ni0+jjXFZXriDucyMOsTR5ZSQVnmwz
YWjJR7o8vjH8B8HEa8+a9bXFn5oh5VkD2Ki0ZnVhAZRgb
pp1+TQfVaJ5Khu5N0Pfx9zkqvrk9sFcFTqt+AHnOkjSdqUFXM3VFlUmDqEmawxRGQGX6yuSAUAG
1ynWjKKFSBEhk1KsEKPb+d6ASNY/TtN8Ws0vTy8hGzmKgV+4UMl9ze7oKJLx5pwFwSdcnq+a1M
C+xbS1bUR0j2EkCAWbE5/HFUNtnHJ/oyN3Y2NP/I6sJ+UlhYEl5Su+LZZVF3uKkhmBEaI5GcOSM
pBQWMqvoVhx4adoXxwQPE+mWuo5xz8BLPVaE9xGvTnSkcNUfATqjdTkBBmK1X+Y2J5NhEs5HdvcJTEfWWD
3sc6+A0ZBmvvr6OC/GHtmjLmrcXNtOpVZ2c8ljX1ZpW0m3spWAFKAkFP6Bjm9Cbev
kjNySxAXx1TUrxjWfB3c1Vz4tsRR8VAyZ4C+S6GbhBzeCgHFRW0kdMfxf0/XlP2zK9W2y0a
Yt7qgz+IMNVEVF3Ivnz/1yVWMlRFVx1YSEnqPcM2hcxEK2m2nXbCAF6+tNSPMFjv8+4+T
WC5zJdzB7ldPgq4X6SUVYYQWQg7yTvtvV/72wqszSnhL7g/mfIJyoGEnthjlsZHuE+A1bc+g3ZpnsKP7q3Zr58hmDDwQdHfDHt1XrD6bNYI3JyAAFbrvjqIS+LPSVfLhjaUGYhGoQhCsiTe+9VQzOivPZ8ARUdPIHjFM/d/e/MgLjeHKmg8v+78kH/vZsuxnzyhlP+FnvKQj2T/sLgrRz07lDt8HEdr5azl9C1p9L6bJ+Lsjmqy82+TeEN9rtHknsG+5A8mTr2rZXKrGTmQsLnPTr/bYzRyGqfxN3BYOmH9W/3HvA3sKe5HzQjrAFgI4cvQGQZMtqs0WYhvXDtUvI5OHp6lz+nFm31mcfDpvhNVZLKIR4+QdmJG+3KZAM3HIvB7CPQgrtXH5rxyrTjdtkIyTmUeOqh5G3a6PtWXtMAHTYMeh8i5gl7o9vG3RxdHsfdKIMo8HUKFvQDDC2ChSpklkZ9RjTKEC3+gWgoxaDiyEbahYu3K9J9HEXo9AyKaEGAtj8f9MIRhNx2Qpd6ZvBrcLcSkwJvppRNFTXKFLb58HYz95pHXIsLj5W4q39/+fdSNrT8LzmaX3IK/SiUXKxAzMZKLkEzWTTrT1mLOvlGVb1FXCxOxHM1z8gbtoVG3yeHlwQGs7F4NXcLnXfOyjrWPd0afuM9JbSUStXBTDXWwXmjRSbBrL73SUO0L/71t/oIxRjlLVz2SHrN/GZCfOmTd7ZUkiwkjJ9TF4cs4x5Ma1Wr/qFTlaNQ5W6Msdq61jjM+K5f7qxTp43LRtrs+Vgi2OcLhvFwJ

mNjtW/fPi1cuJAwAwAAPGbOnBnQ+kyHmQ0bNmjp0qVauXKlevbs6VmelZWlhIQEOZ2/VZmYmK
hnn31WOTk5ps9/AQCAmi0zM1Off/65cnNzFRYWpi5duqhHjx6y2czd928qzOTl5Sk1NVX33nu
vzj//fK91Bw8eVFxcnNeyqKgoSdKBAwf8CjNOZ8W+D7P09kBfbxOsrRg38xgz85j/Z1baZ5vN
ZvqHv2d7m0yXPfX5MqbL+tl26fZ16jj8urX796c4KoOVP2uBVlRUpNtvv13r1q2Tw+FQeHi4j
h49qvT0dLVv314ZGRkKCwsrd32mwsz06dPVoUMHDRo06LR1hYX70YKJjg42NNpX9ntNoWH1z
v3hgHAkzB9w7iZx5iVD/O/fBwOu5xOh7kydrvnb9Nlbb6X9bfthg2C5XYbq18/xHS7pdxuo0p
fyWDlz1qgPProo/rqq6/05JNPqm/fvp6Q+ju+u+/WrFmzNGvWLxHmzNGvWrHLXV+4ws3LlSmVlZWnV
qlVlrg8JCVFxcbHXstIQExrq+wOW3G5DeXknbnzeHxwOu8LC6iovr0Aul7tC26pjGDGDfzasuYB
SqAMP/PrrTvLpdbJSUuU2Vdbrfnb9NlDd/L+tt2kNPuua07+4j5z0ZU41AN7t+6Sr7fVv6smV
Ge+f/WW29p0qRJ6tevn9fy7t27a/LkyXrkkUdMtVnuMLN8+XXl5OR4XScjSGfS4/Zyyl7RfWVf
TZWdne60r/To6ldYOpUp36vpKRyvuknfyDU3A9YRWHczGPMyo/5f26GYf60iWd7Q+ZPuZyyuS+n
a/xpu3T77CMn9GP2zz63XZXfbyt/1gKjoj0cHxmsen6ezyYyazafdfRTT9AZxdvvOZ5aw4sen6yh1mHnvsMRUWFnotu/rqqzVhr
gQNHDhQ//d//6clS5I5XLJ4Th52DAzM1MxMTFc/FvB7Habz4dGEWw4AKCyDRw4AUC+++KK6du
2qOnXqeJYXBRo4cKFuummm0cc+KKuummm0zVV+4wc6ajKxereWrWrJmOdOmafTo0dqyYsWL27
stLQ0Ux2COXa7noM3iseAAgMoM3iseAAgMoWGhqqrKws9ze/fX926dVRL16+0+PBhfffh9eL16
wQUX6O6775Z08mjaw8mjaww8/fNb6AvY6g4iICKW/fE+p6+dlDcTfufMxc4OccUz1aivGyCK0
M4O0+ePeSw4AKAqrF27e7+cPYeBAk0RBEDHbRU1aNa1gHSTMUfXVkTbFlU0RL1i+PX5+gDuzYU0
iTrDs2NP2CzYNa5VdPUxF1q4HurVKYqcSVAUyr2rNFIj5cRgf/y4nbc2j/tHB69vGq/t2dkS8bx1
SqaxWKcLKrdp9nR71+pnwI3QFvEQnRa/RuTlRkOpLnNjszkl9o82g+pZsEnmH9fTZGM/VDo4
iP7kgCrQXpBdoJi5Ri82RhhW7JuFAmGpvS/xvVDQUftD9hmO9NkHVdEh2RXhO0bNGT55Zd0yk
PEmD7yV3aXEcjlCZn/DLp6+AuiOz2oPm5+SgMPEwUpQsHcBkg46nTjqZA7SWmUnFSmeglD4/AtE
PuCVn7wDlJr20KvnnmtSEbEMlDqAL1TO3DUBJXsNzx6jfDbA+IE3/cM9EOQr3k7G5UpUzVnI6m
LTk2TJWAH31tJiNmHjNeQOjR5ofIntkmxQDFakrEcaGRbL0P8UUd6Tsw/yo/5Ud3h7b6Ji5N5JwT
qqtebjLl0jQsQQyeVXLyowZDIEgZsy9hwFi3bwjj8ru+AD/WCxH7oZ/nsm2FLBTeaB/Z6p5gDvl
+0pn+Wed2GlmXWlJ4ZaXZBNP0Hp+Xs0u+6NVtnVkpdDuxadk3pBQKGmZA/7u/PW7unW9r+Z5i
vnHN/pUfrWH8YnmsUaAjXdGJCYnvaX1C1YuNfEqukPlcB7uM+Ckk+AD0bGzC8pNP/6/zxJl5
3qX7/r/D4sl2DyeW2OkeDb1xEPd3cqxqVKp8VWHG0eoiaxBRNeFQwWh/fXsuA+UeBEKcwJRIsZ6
C8h2p5PHXTtzTyzqqssQ2XXAQWNtcVp13LPVU+yWTAPeGSzs5j/Q7D+Gy+BlnOxgGEKnW9+7wD
sruU7gQRVKJIoQQDUKVrwOTZT3MPTxJaqNQm4LA0rZsXTMOnL8Ab4sPVE7vwMNjvtnn0Q1TYHwS
uHnV1/a9Gqsvs6YMvPk0f9F0ppmhp+6tb+pqVxbnOuzEJ5EWOzzNr0kIuWHUKbHg4UqJHTahp
U4ex1n3HEtiTxhnu4aYCHQbRDfe/Oie+Vdd8eBHLeAKR/xO1ByyKLMW0lA/zZM+YbVhPAM7/S
Et2cwqF1eLYPZr/GvqAfvmoAUNBfS/VnFv4ZbJAPDphn1MFvdDTFV4LLGL/ZQyUy7KOyHXPCW1O
9w4D5rrXWedm+4kXDtvYgNbXn8N8OchPfzu7aPT1UWgZmoNEfKOgfSgFYEe6+xeTpP2SqCRzKP0
YmBZk7+DY/NS3pKr/QBeGDLeR+XDvPnQyMv0/a6ZIt/xl/mXqo2e2+8YsgAotGNL72Aeq4tFG
97wlrsw7cwjr/CSmawn9VTVbS7r+zfFtJlqhH2Cm0Zm4b7Pqbap/FXc7b+FfddbX1NeaW6xWSvV
/tI3HmXg1vUl1QOyfp3P3ML2gjoSSXHFmWxe+sXq/y8CYDxMNJnlkLTYXfCbu+/SnRZ8zrW7mG
PDY0tWi8o9yMeW0xlnP0zsP8+A0MQdSgjqOp4qw5AS3iqhXXM28BH1ocjM+c+oTX+c9obVxq5n
5uG3BrQnKIY5kk4jAMV3tw3L6kswS4g+VJSr+oC7zYmhXDMvsr+CpxL9U/2ftLbmPr7ng7ms2m
Pu3kIpsWQb2gSBXL9z/pA2F9F3kDEZgvfGFvgEEs7+3jL/v/l8W7ffrYbmLjrtAYPfHXPP96X39/
Fhcu+VRGLXeh2BsP8LgkqTSzzBaP2CWgo58mfThK06krtgbVv8ZQvSXhwK6ECsnfTh9P8f0vf9i
SbJ1M0wN5WDjTsddBQJIVJTB2P3qsSoJbtJ2/8HBxufBGRXtC4d1+Eq2PFdDRcAqn7Xl0rT/7+z
8ePVRDBvyn9u1XHW2xXqFKvGA5OQ1g4nl+xs3HB2RSBiMyHjr3nbMpMuXAZvlEsPGt2Q8/bJ5cI
vLjtY8lCzUEnYo8X1K/ij3DKZuL+NDLyeHKGb1f2UMYnDGc2aTHKgzbpkVbcF7Bk6cV6gM7M2Pe/
r3gZrLlX9ZZ1wJDLQn96lo/YNHo0eL56x0/sQKWPfu/yIMxFPB+lYUfdpZVP/5x2ijV/fiFGJ7
Y0l3NHzmyAx4V3dRQfMnjqKnfXNqVbPhmD4I0Cz6dn3DNH5Z5XALmMEI9/D/t7HjL7NbiQH6lt9o
35+XBnoA3n+Rkq4VqH2XQAA2Bqz2HxeJHmc7KrpFaX+DqOT2HPq4m3+vmsbvN6mQmBbbEEeSGzL
rJLcvgfrKrjT0Qz2n/W7n5GhS1WAZFjbtb6Vky/VrP9OHFKw+cj2rXWUuvVSm7k5wS6r2DVs0
PNdd0xyHs5lmyaEnMVfGLpm53qO8bxEcCDXEQOAUQjCl0gVSKeHZlK+lRubXFmW+OeXAzgdtG4F
tVe96V8LeyoyN9QMaE+FnrmTMtDz6B88vmBoyCxCpHnWA2OFhDzG8L71ciTmaLyg5tAAAAzfRkx
0rwOdBaTf5oLvcmN8rpSm3XuAF+wfCnZdnGtD0DK8ek4n3O9Yw4dhs5pD1qt88v4AiUWXLiotjt

RRRdpwIABGjhwoC6++GJT9RFmAABApatfv76SkpKUlJSk3NxcvfPOO1q9erXmz5+vVq1aacCA
Abr22mvVrFmzc9bFLSEAAKBKhYeHa/DgwXrppZf0/vvv67rrrtPbb7+tPn366M9//vM5y3NkB
gAAVKpLL730nA/Ns9lsstls2rJlyznrI8wAAIBKNW7cOFOvMzgXwgwAAKhUt99+e0Dr45oZAA
BQ6V5//XWlpaVp1apVnmU7d+5UYWGh6bo4MgMAACrVvHnz9PTTT6tBgwZ65ZVX9NNPPyklJUU
PPfSQDhw4oBdffLFcdzGV4sgMAACoVMuXL9fIkSO1fv163XnnnXruedkGIYefvhhRUREaO7c
uabqI8wAAIBKlZub63kb9g033KCjR4/qxx9/1AUXXKBbb71VmZmZuojzAAAgEoVHR2tQ4cOS
Tr5jJmIiAjt27dPktSgQQPl5uaaqo8wAwAAKlXPnj21aNHEiHT9+8kXD7dq10zfffCNJ2rhxo8
LCwkzVxwXAAACgUrVs2VIvvviirrnmGnXu3FkHDx7UkiVLtH79en3yySe67rrrTNVHmAEAAJX
qH//4hxo2bKg6deroq6++++knTyib979+5VcnKy7rjjDlP1EWYAAEClWr16tWWvoNC1WH9fMAACA
ShXIICOZPDJz9OhRzZ49Wx999Jx999WrVvWrTZ48WV26dJEk7dixQzNmzNDWrVvVvqFEjDR8+X
KNGjQpohwEAgLX16dPH1LuZPjgg7OuNxVm7rjjDuXk5Gj27Nlq3Lixn75ZY0aNUorVqxQ48
aNlZKSor59+yotLU1ffvml0tLS1KhRIyUnJ5tpptax222y220+lXU4/D+45msdvva5JvB1zAL
x/QIAq7viiuq5kWT+/bt07p16/TKK6+oU6dOkqRp06bpk08+0erVqxUSEqKgoCBNnz5dTqdT
sbGx2rdvnxYuXEiYOQu73aZG4aFy2Cv/P7kGoXXXkdhsKC6tb6W1bVSDGzO02ZLPV3iAIADNnz
gxofeUOM+Hh4XruuefUrl07zzKbzSbDMDBts2DFt3bpVCQkJcjp/qzIxMVHPPuscnJyFBERd
CO1xR2u00Ou10vv7ND2UdOmC7fqkVjDega49N/jiHBTtntNr2yZqcO5Rw3Xb51i8a6pmuMVIv
+X/Z3zKIj6mlw/9a1+qgWAEiSYRj6+OOPtX79ev38889q2LChrrzySnXr1UOM2FhYerR
o4fXsrffflvff/+9rrqKj3xxBOKi4vzWh8VFSVJOnDggF9hxums2KMWpYf3MLd
OAn8/85RjUOPfkPm0wHmtLts3NP+NZ2ROhpdZlt25d++1s+EGV9HTPbryHGbrdV+Oe6pqju87
+0nM1mqzXz4NRfYCzV7wC17e9nhdPNUmFhoW655RZlZWWUpNDRUx48fl2fl91uV3p6uq666irNnz9
fQUFB5a7P51uzN27cqvHuvRR7969NWvrWrNMaDg4OliQVFRX52uqzxp/zQMqSn2nL12jjX33a1cdgj
4nDY5XQ6zJf79fSUw26+vD9lJclhq8q2q2i/A9Xv+vVDTJetjaw0/32Zw5adB37Mfb/brsox+
zWE+PtZ4dS+NHfuXO3du1fLLli2T3W7XfcoI0bN+rjjz/Wvffe/zeqzUaxWa
spU6bossssu0+zZsyVJISEhKi4u4u9tquNMSEhoaeVkd5ud2G8vLMn34xw+GwKyysrvLyCuRyuSu
0rTO17XK5VLiMl3e5XZ7/jZZb3p+ykuQ9QyqrDtKtrvQPU7P79Qv/xivrxVBCqAWGH++zOHLTsP
/Jj7frddlWP26+fD389KVfxfU5nKM//nfuDXDdhvyWuPbGmL4SDIR6X5BEt+NVqGGZUTUsmzHNqStSI
iYnh418AAOARHR2tZs17nuvffeU0xMjKn6yh1m9uzZo5s34aM2aMcnJwPfd5YRR0+f
Fg///yzkpOTlZ+fr2nTpmn37t1asWKFi9erDFjxpjqEAAAqNmuvfZazZs3TytWrPAse/fdd5
WamqoolS5bo1ltvNVVfuzrVmzRr/88vee+89vffee17rkpPKS9PDDDys9PV0zZsxQU1KSIiM
jlZqaqqSkJFMdAgAAANdvYsWP17bff6t6Wrvq1kt9v197//XVFRUXkrkcoc0OcO/BAU/WVO8z
euut50xxK8fHxWrp0qakOVCf+3C7ndtuwAQPXEE8Fx
BUVpbi4ONl9eIgsb83WyWcH+PtUV5fbra05Jwg0AFBD8QTwwGvUqJEkqXv37n7t
WprlGNQzXkmktlt9sIMwBQQ/EE8OqLMCNntP9wflV3AwBQjfn6fYJ
UAAD8Q5gJAN4+DQBA1SHMBIA/51H9ees1AAAgzFYRJ4+4YAAFgaYQYAAHYAF
YQYAAFgaYQYAAFgaYQYAAFgaYQYAAFgaYQYAAFgaYQYAAFgaYQYAAFgaYQYAAFgaYQYAAFgaY
QYAAFgaYQYAAFgab80GAEkOh2+/2laDkDgEGYA1Go2m01ut6GwsLpV3RUAPiKPqMAKjV7Hab7H
abXlmzU4dyjpsu36pFYw3oGiObzVYBvQNQHn6Fmfnz5yszM5MvvviiZ9ma/uKkBWDmTQ6Y8YMbd26
aNdLw4cM1atQovzsKABUp+8Sbl+PTbfC9xsc/dOHD26fqmC3aaJ/96vu4kJ4YsZbzPJJSVFLVq0
0PLlyzV+/HGi+5G1fftdztjR6fcFqDrYLpNbKAFL1PfxXOwp4UQL0OmdyRcsOkOZYWGTELRHHEKXi7ycckmMSTFe6
1599VUFBQVp+vTpcjqdHBW+gG2MadAFhhNy9A6crsNf1qUb5MLLN+4uwo8njAJ5Y9luSvVQVbGW1
9/vfpC0qLnu7FG0Dgx0zt5oNT44GnqsnJEOIQ9ueLyT1VpTzNDmMfuwFo1p5
6TUAAYBEzz0+fqsqMahGnLNpbLbbYSZMpgOM71791bv3r3LXfxw4EHFxcV5LPLYuKipTHJEdZ
w0cw43RW7K2PnkN3Nvl0EZ+njA/l/S1b1W3rlCK1Zb8DNWalh5lxbsz/ati2H3Pf37YtO2a/l
pOk7NwTOvCTuTBT2l6d0g6fHwfgdhsyjJoZhAJ6N1NhYaGCgoK8CsyFUoKfKrTbrcPL
ye330rD4fdLqfT4VM5X8v7U7bK27bVvv0OVL99Pcxc2zD/q2fb/sx9v9u9u26JhJ/o1bwwBfp2
ikk6GmZp63U1Aw0xISIiLiKi4u9lpW+Eh1R8CB5zYjkP164fI5XarpMRLurzL
7fb8bba8P2WrvG2j9u13oPqdn1+oX34+4xX+oAhVAmP/Vs21/5r7fbVt0zCT/xi3o1wuAX1mzU
9lHzM+JqMahGty/tfLyCuRyuU2XN6VUDDTJMmTZSdne21rPTr6r6Ohon+qz+n29l+HTuj
Pk0yE4TxkfyvtTtqrb1ilFast+B2rM3G6jwj/XNQXzvxq27cfc97dty47Zr+VOq8tk29lHTuj
H7J/NN/1reZfLXSN/9gT0ZHRCQoI2btwol+u3+u3xJmZmmYmBgu/gVOwa2ZABA4ATOyk5ycrPT0
dE2bNk2k2jR4/Wli1bQWpGcCyuDUTAAIvoGEmIiJC6enpFhZkKShQl1J

SUFshnAsrg1EwACz68w8/DDD5+2LD4+XkuXLvWnWqDGy8717dH5AIDT8aALAABgaYQZAABgaY
QZAABgaQG9ALiq2e02n25d5XZXAEBt4M+rEKrzjQc1JszY7TY1Cg/1PG4aAACcVPpYiLCwuj6
Vr+6PhahRYcZht+vld3aYftRz6xaNdU3XGK+XpwEAUFPU9MdC1JgwUyr7iPlbXqMa+/beKMBq
fD0VK1X/w8wAzs2X/yOtoMaFGQBl8/dUbHU/zAyg9iLMALWEP6dirXCYGUDtRZgBapmaepgZQ
O1FmAEsxtdbK30tZxU8mgGovQgzgEX4e2tlTcajGYDajTADWIQ/t1ZKUqsWjTWga4xstpp3JI
JHMwC1G2EGsBhfr3mJDK/5R3R4NANQO3FMFgAAWBphBgAAWBphBgAAWBphBgAAWBphBgAAWBp
hBgAAWBphBgAAWBphBgAAWBphBgAAWFrAw4zb7daTTz6pP/zhD7rsssv017/+Vfv27Qt0MwAA
AJIqIMzMnz9fS5Ys0UMPPaSlS5fKZrPplltuUXFxcaCbAgAACGyYKS4u1qJFizR+/Hj16NFDr
Vu31hNPPKFDhw7pvffeC2RTAAAAkgIcZnbu3Knjx48rMTHRsx48LqDK8ReffddjR8/XpsXtnnc
XbTbJbrcr/0SxXOX
Y/lR1nHaFhtTxqay/5WnbWm1btd/+lnfYbaofGiS3263y/MRwOALzexLzv3q2bdV+07a15r8Z
zkBWVlBQIEkKCgryWh4cHKxjx475VKfNZpPDYSv39vVDg869UQWpe3a1bZV++1vebu9cn9IM
f+rd9tW7Tdt+6ay578ZAe1Z6dGY31/sW1RUpLp16wayKQAAAEkBDjPnn3++JCk7O9ObtreXZ2tp
o0aRLIpgAAACQFOMy0bt1a9evX13//+1/+1/Psry8PG3fvl1dunQJZFMAAACSAnzNTFBQkIYYNG6b
HHntMjMjRs3VrNmzmzfTooo4+qSZMm6tevXXyCbAgAkBTgMCNJEyZMUEl69vHRX3Xh9l4VFhYqISFBGRkZ
p10UDAAAEAgBvTUbAACgslXf+6+wVXf+6wAAADKgTADAAAsjTADAAAsjTADAAAsjTADAAAsjTADAAAsjTADAAAsjTADAAAsjTADAAAsjTADAAAsjTADAAAsjTADAAAszacws3LTbUDdt27fXtuduzbse0OHRo2bJg6dO
Aszacws3LlSg0cOFDt27fXtddeq7ffftuzbseOHRo2bJg6dOignj17KiMjI2CdBQAA+D3TYea
NN97QPffco5tvvlmrV6/e6/efXtddeq7ffftuzbseOHRo2bJg6dOignj17KiMjI2CdBQAA+D3TYea
OwAAgJxmNjYMQ3PnztWIESM0YsQI0ffp0OZ1OxcbGat++f
Vq4cKGSk5MrZAcAAEDtZurIzHfffaf9+/dr0KBBBXsszMjI0ZswYZWVlKSEhQU7nbxkpMTFRe/
bsUU5OTmB6DAAAcApTYWbv3r2r2SpBMnTmjUqFG68sorddNN+mDDz6QJ0gDMHbFu3nbxkpMTFRe/
JOnDgQAC6CwAA4M3Uaab8/HxJ0l133aVx48bmcjObzAp7ZyJsq6QlWr6G68xwD0dtEKxn3aaJv3jiHamJmnUd6TYBwPLq7ZUcAKhvFPLmzKF+nHjFBtH3EJT8pC8u8Rb6hJRt7rE2pZ1urmnkE4AqU+0PGOakHdEDlB8eezmc8ifxXOjDxtXvHvnJbTOHdd52mRevMi5i3VB42Dxuf7SpDVH7Ok/hv+vYgFGeclTzgcN8sGWyuUp3sUqUYYN59YcC8K8UbmJ+7Sn9oURDfHSxD4D5P9H/0YpTXQT7KWwJ5YKdbRqZ1XBUxcdmDSbbMHHvnTs80mzb9F5DCoMqJzZDNAfoAvL6MOLbIeLYB2Fk0Vd3XUZ+xW8uhvkmO5tv3fFeT8QakP9PDjZsVKWHzBhw2J4WH3EZVOlf0OQMQIChYAM+xe5zhCNXh35bZC3W10tqDSFE+9kn63VOMLAuPXvyB3K83CAqDvZjIbqeGABpUc3l79gO0C6FeiHKXbvRrsoXf6b0BZXhlh6RqIedU2DPOZRQAqmtUIi2jh4LPPUiFL5uAkJQM8D2tmWdQsOdcLL1UsOpYF7uAFXrzTVRuawnJdGtJIxuEnYK6T72OtaIKgaWYjUUxFZD6fs2iTSvw1HE5bfQ1WOtFoAdm1N8FIU0szVeI0b60+NG5HJxSbFTY1y1SfRsT9cTakAddSxzq9Kz+0jEKlXf/ptmfGmN65Yr8/xbuUHV+G6m8cbmv32+rlMp+UAPL7Qqwl/G8OMPb09Mw6jdA5T0Yn3AuzDdOH1efbZm3LvYHXdWz7dWQnScYGwb9rfSkf8FWxEB+FCTljdFkEbOXDZ+NLn0TrbvVz1Et+qJ5P+rRSfM/OZk2nbTsnj2s1zKdGJLN0VCH4c3v1lwRmDgMFPrYT8+VG13cdMJvz6JZuQKKjfNjt8HaVgU9fg8Y7rrxiMwPhBsCEEw2cqPhzncFzSWBdVCxKOmmB9e8ihmC8nVxtUbCYmwn6CYzFrNE9sqCTU6mDhZe4HxZy/NTfBs3lGvVQFR0k+Si5GzTMhgwNllF+yfXZWzbXpIFtgu3kyR0k0IVbCCnFPkfSNeTE7nWm1PmDqeMvu71ZnSMDLHEq8UZEJ2GzqOnrLfwp1RXpkpzlPpkv8QuMDSnKWpVRr+XTZEMTHe31lbx5JHqnOYxRMXPWUQhKyZ8xbh5i2sYtOEcfRCG2ivVEmdM5kGf96XH/mB4hJP8XhZIBm+dF/yWGUmIABq8zOGlTbTm/BpkAhfTsJpNiHD/bBb29SDBq/QoC1B5hd3MjnlKYDoMQkWGaDdSwvCX9/86LCRJSxQBfr69NmK3DMvQ4gL5hVKCFsBmzXbMmZc/GJRxPmEJdv23GOpNMXvk6tIIJLhHbvhk8eTfH1T8CJpPnLbAjRvEV7wD4hwmlbI+G6/ygiRubKfyvDnz3rAz0hSVyDGNY4uXxCMlymP8FX2TQvHGzpX/8nJhA0VxTk/r+2MTXKBtCeOzfpcEhm+68oX/39ENBg8Q4OX7pP47SpT3wfhdDfuG+GUlhOLVwK8uHUMiMnzvIkKvSSKrf8DFDy6HhdbMYk/wpwY9XenEKbvFfPeMpdkGA0dRrnqqJpGzqKkZgAFXqa8W+cPSeRW3HVgx5wQOUrB0wL8S0EOm1SPHYx+UBBrBJzn/H3+YgYR0+7zG7CoH/9Eu4SjjBHOn+8Od+BFNcZPtvXDiE1ZIMm2TvSAg3b/cSjEXpTj6CSkNRUvYAAcSNpJPm99JX4H5E+RPhbpmzXXhfEOhbo+4DDLjWGUlvLcTAhMkqXSFZmfN4f8Xc5BMzHl/R7/3sp0JNn/AikRBG+yq8YoDi1oKKbWN4/4BHZVA0D7OA6hQ9MzsoYTlp9UBQb3+/tPYMtkIJH02v6lMWR3QWUVLlc/0ufpcgqVBIB5RAm+nfyMAZNt2VtrkHjXTxVkgKQK8NK1KjKZz3v8aqBKJoVqD0l3TMGSafSsTyO8CqL9Yx95iX17r+4PtVfJDzKVTV1oSO6z8CjqJlCrw3WaOGP9DT+WLGzsFpp/ZbDq4FkDTcT3ZGC6o1Q/HMg1Lq4WPE5/EtULLMg5rDSI1XhIUDyrQG9WdSRkjIxC5yy6rCYmuJxNGkmgJn6uKwwX2ZKDdGJVQNDHoJQpH3mgNZpPTsZOCEsc5Ag9IAMSOjJNklZ+BZVKnKqeYzOWtlZxj73QJOpgAYaEAZAkGHyBTfqE5zvgnhSDl8DCQsQX3UaRvTxvy5XVxQjHlwVi+IjqTjCFCxVQORdp9HP8ulS/8hmlSv3kk2gthCj1zvQ/EDmZwoP2D8URQVR7q5TMnwJpr+8HJSRa33RMvyklW9aXgb8UDS5s1v0UF/OlQe6p3oc0t/0wxZ9yNNTrW/ZflLlTHFR4LtCP7pB7Dn/Uczvg4g7l1qyL6uRx9wVeD/oe8A8y2+K0DdAdMXeYH84NCWqA2PH3n0gwzStL3tLZNS8/VTzgBRDyUpl1t6zlEHJOJM8O9a+xL5HKwWRbGKAH/4sZyQJFW0S3ZklxTUMmxS+NwMROmaI4N0PG3b9sC0DBK2sxZpMsvIL7QfUH10KmBlyJs0BBnPk/lrJAQ7XUTd9aHk4kHLZDWDcTlxTBFxsWVxjA/tAcbW/6gmHXmAAZHtpUxLtZwF3XTl3HmBfO6KC9K/+zuB4VZRYjWqQpqH6AMWK0yHgjmATeGqSU6VGuXHwkaaHZiI8vNfXVRxjqPJnB4H6r+vJmtiHlsxtlt9etkvTOLgvMkVkeN7ua07fLQsp8B8x9ko5o3uqbdYiKVNP9eI0/6Jv2wDpnp6S8b4J1lUWnE1cBbaWkd+WTSKUdKpeF+Pz4xvIMA2gTQY7XCmfzeaTMJcErqmW3uh2bhnxGTUvSxnpmk+rxu0LSMAltMwzwnEcT5j6dbTwsCGfhwZAnzsr5hOPuCVZQRSPRIpm92kEx9sTxxkWVXSt7K/4dT1KFeL4z3mbNvGDlrY7Pk6AlVhnRF9Zrp4Y9xcr28mDHPkXmy1PLbZxSj1rnbAoW5p2wHnBmQnB+JOfRzcjBbbJr//3/qsU8eCtMFNf5qhLbRTbJ6xYAb1lnVcYLfnmWRYqXzcFbxZ2Y6NfP5bwjPqn/bz6ds9CrJbf9OMtzycZfFwgE3L7JEOJQtT7B/0wSsFQlFgSv3X8JE5nR2AFXdBbAZwoxoWo+AAn3Gky+WL6oSG7JWVxnJ9NBdFjA8zOEg1xHcMbjnFyk7IH5Y2f5wNMHZz3Ttmh9oyFhT6ZHwgvwVahz8T6wY8eSV7WnbS5DUhT7rLbaf3EmQthPzq2i+vCIbhnCUwFNxKlU80W3C92cIJ9EX4WjUJl7+ZLmNiJLi+E4h5Z6nG3jTaCttwwRMoI/RHYdCN8tXPLLnmnJVt8NWfcgf3sIKsYHyJ6MPGG9e2uKWqv7jTArv7mVG4MmgiW5hsJXi7O9h4gAvLaXJ8OyOYV4eXSDrz9rzk2fT7g9sEYjA6rRAxjGpmHwB9pz2tJrYZT6NRB1VsXZHFkYyCBFv5skCM3XoS4MPLVLzGyLzZI2yEzYtNpYnhnBEqRuhCjJg8MgDvtDHkLkJmQvzZbNG/w0ujOFpR9Y4sB5Ao1QgUSRVy+Qz0JwwQijfvxcq/ehhbPDPVAwE8ljtaPIUiFAvTd7YsGtdiMKlYaMUu7UmXgkhY0ClZvSCh+/qd6FEm6ORP0QC4GvZWMRAQM3O4SQjqwLUlPn0D7JSH0GZeTMKVdGuh2AtsaGE0LjE41YeJ1qXxGpJ0D7mdEE2Jf5N4KUxFNLr7cdaX8BFIH6/ETe7iAdQP67sznpjF7SF/pIfbWNNdwBWT7sO1fnHMm+R6XIvEjCmRohRNXtfHqndSAoUVpN4WRm6WZTLjEo6n2FJrM6BMj7RT2G6+0ZMJHPK5vSoHY0DdjV6uZV7i0Lm8xsxxJQjPYbMfCj9A16mR2FK2UEs4kgRUq/4OL+ZbOO3/m0I7JNTxWBx3YlxTvUzRAS7hfy2vYJsyM/5T2kb3v8pG9qjkwptq3Y6qRjSnsLzPKBTp18ZuW97hNmBtX5LHRJJzmSgjAbITzNxcNR4iGOfnAA+Y6lUPSMB8rzWm3E8x9ERkt2SAZRmFhJ6B91QNwOqwQ9i7kQZG2Rv3p1wF0XHoR2DTMQ0Cr6JgLy9IyAoUBWBuMRGuL6Rkf5qTAIFB48ZnmOWKkhDJBoXmF6Q7qD2AWXuBG3b9Vy1KfsNyQNRSW3IvP+2Re5mqwt+RIeVn/mAAf2nwF6nwzS+2pZFgJAtfpXyXOqgMlLiwLp1TqYoOqH8sMPp9Pd9tRb57nG3xR/QvjRmlCvTtfGbxYSXZOF9iewsuB2qqM5XMi4cFhd03sd1Ohj6wn/+z5Y1uO4jufzP3tUwGxRyRhATrW/yIsi7TwmcO6Ds5aeE2OPb08KldpnkgmBNJSXFOVcpdmxx8Uq4BnRTJsSdPPyvBPsOrIfdn0WIbDM97P+wv0YfQvXlNP8SkGuaobHlk+4dOu3vnGBkUlfx/Svb2afvTD0HVSlHb1QwN7fdeNBU8vEDdJr5u/8C0I0H1hSVjg==

m4uLidMkllygtLU3333+/wsPD9eqrr+rLL7/Ua6+9pvPOO0/p6emaNm2aRo8erS1btmjx4sVK
S0sz0wwAAKiBxo4dq7FjxyovL08nTpw47bZsX5kKM3a7XQsWLNBjjz2mSZMmKS8vT23atNHzz
z+vVq1aSZLS09M1Y8YMJSUlKTIyUqmpqUpKSgpIZwEAgPWFhYUpLCxMknT8+HHl5+erYcOGCg
kJ8ak+09fMNG7cWDNnzjzj+vj4eC1dutSnzgAAgNph3bp1mj17trZv3y7DMGS32xUfH6/Jkyc
rISHBVF218/4wAABQZT7//HONGTNGkZGRGjlypGw2m0aPHq2CggKlpKQoKyvLVH2EGQAAUKme
fvppXXPNNVqwYIGuu+46GYahiRMn6o033lC3bt00d+5cU/URZgAAQKXaunWrBg0aVOa6jJVH2EGAABUqjp16sjlcpW57ujRo563B5QXYQYAAFSqtm3batGiRV6vOjIMQ/v27dPcuX
PVq1cvU/URZgAAQKWaMGGCvvrK02cOFGSZLPZlJSUpIEDB6pevXpKTU01VZ/pW7MBAAD80bF
jR7344ovatm2bQkJCFB8fr8jISA0ePFg33njjjaS+tPhfCDAAAqHTx8fGKj4+XJL+fT0eYAQAA
ler1118/5zZm3h5AmAEAAJXqnnvuKXO5YRiy2Wyy2+2EGQAAUH29//77dPNW/eP
M2ZM8dUfYQZAABQqZo2bVrm8tjYWJ04cUIzZ87UkiVLyl0ft2YDAIBq45JLLtH27dtNlSHMAA
CAaqG4uFiivvfaaIiIiTJXjNBMAAKhUffr0kWEYXssMw9qGDaa5aMw9CRI0dUVFSkKVOmmKqPMAMAACrVFVd
ccVqYsdvtqlu3rnr16qVu3bqZo8wAwAAKtXMmTMDWh9hBgAAVInMzExt3LhRBw4c0MXXXaN27dl
6aIePXrIZrOZqocwAwAAKlVRUZEee+wxvfPOO7LZbDIMQ4sWLdLixYuXP9WgAAVBkzu9Ua
TcTAACoVI8++qg+//xzPfjgg/rss8/0xRdf6OWXX1bv3r31+fPn+vrrrz3Xt9daPTgzG15
uTJk1Sv379vE4pde/eXYH3xqj7CDAAAqFRFRUVnfApw48aNFRQUaKo+wgwAAKhU48eP18SJ
E9/nT95S9/UfPmzfXII4MAUhIXVrZR2ahrGzTzGrHyY/9UbY+Ybxk1KT0/X4
MGDde+992rbtm0yDM2NwyDEM33XST/vKXv+iuu+7SM888vcrJyVHPnj29lt9///3efnuZVHPnj29lt9///3KyMhQ06
ZNlZ2d7bWu9Ovo6+j33G5hHxwOu8LC6iovr0Aul7/aPl7ej2ctz9bFdfYBcW9/+ziqm8L4fGnmOkx2Bml1okUSxQdSm1sBAZMgDBgxQZGSkunbtqh49epiuz
68wAwAA4ItmzZqpWbNNmkqTZs2f7VRdhgAAVKp58+adc9r7w4pYq9knPHKaHIiiLUrFkzJScnKz8/X9OmTQUu4RQo1Q/3bu1YsUKLV
6HmjZtSpgBAADV1/bt9duTIEe3atUvlbtbS1PNOe3tuTUPPmtYV8rGLTYiYIGERkMZ6ANUAJCwNMIMAACw
NMIMAACoMocPH9a8efP6ux5Z4QfCrCDAAAqDLDLZ2dmNeWJ/ea2Bk9MLZEm9JMNTFIPiKH
QgzAADA0ggzAADA0ggzAADA0ggzAACgStlstrN+fS6EGfSG1G5UGQ1gHHc5xjpU0ZUdNUV2KAFBIGjZsOuv97zazOtk5OjO+9
LUvHlzzZo1y97zdXh4uNfk1UGYAQAAlkaY
UYmKiOnbsqL/97W/avW/avXu3/2OHTs0w8dw//lKCF
CxfqtddeU0hIiEaOOHKmCggLl5uYqJSVV7F+/HjNyVcq00PLlyzV+/HjNnTxhbKmz0e4GNzwLfBOjBjfx8EYE5YNKeRGeENl/+NGCMvR4xS5tN+IPxGTANWd3UtV/Hn/f11eOiY38QP38E7yqSfi6wmRAqcpx2rLa7CxzTi5Uqwr5lO9uqTF1dMmSNvwGHtS1IL9Cj5MkXYL3QIfx+PwH8j9AwAwMP3EmMpC03Ur4PC2frpGE0e9LDj51a++O8IQ2dw0jP9COeNkBK0g47Gf+L0ndwKXEQYSQ9zfuLvrE4Um82tHejBCBeBAEWdC9uBy87rBBqQrxJaKCAvqbkmWLvLFYhOX4hFTZ/tdOVrxQcrWJaXHEf+dDgB0c1W1ihxVM/3YabvT9XkY7Xr5lhF8r3n0zkXM7iz6fKDiawc6FdxM5QsyNnxIh3GWZ/N/5dIikRvdJUMTaBZJ/XvC5xXBOH3cb/Wk9a5+UJYMZ/8bfbWTGNlsjgZt/JvW9lP+Crtt2yIX0W1dZbh6iI9a9e2tvG+38jcNm8YhI0wDFx2hIwfjo1NM0FPmBDcBrO3Q7IPvZ7WH+fPDBZaHVRdxOfbG1SAYkWz/LvXvjY3lPjm4zMXU3e9fSoNl0XHkOW5DDnB8iVuSfv1Ddx6OwnN4CzP+0s7XOw+8ULZfyx9dfFIyzrk5SqvWjOzCMLMRN3ezAkd6Z+KQUNAcfeOqm/0+V1t+JYwD4CxmEzp/5d4eF0RvuDYXiilanB+fRmwPDvvFSrMgThYBMxhk+YU9TKNnhPH9WNKGYa/9hbLr1bMVjTS+ha0Xr5Jz10YdmwDEcHZhSSq5Abk9OG7r3ETwtdhSd04n9KUTfvL8g6ibM5HFJjv1/sSlXbT0h2zZcgWd0DKVKqiJdXRDpdtK9IxkUDyKTPl+JU3jNT0UlR3yY6hewAhq6+jgIOf/ZkjHwuu9Zrd1sPcHzzbRn2BdbEP7ng21mkoXwsmpBVx3Nz4fAB0KwONlJRcGAUx5ni1y6dWvkHAp1bVZk/a0ofA9Tk5AB3KI0i57J9WuqJZRQaqn3Y96RaQ11knW+HMqgz/Tle85b1z3Q3Nj5OhF2VTNfvL3/YhxT/+Iqr8/jHNOWD7R4W1ov5/Ljs1m6ks0VTHrm9X9ec3N4Y9fyFTEE/M/yI1Xk/s6IeHf8z8pFYH6kx8m3Mbjf9ylCsk5PndjXSW0fK2hlQyCWWxcrBTrNvB8Cg8wWoUNDUJ5mO7RtU79xrZc2Wn+OMDD8pcLLXfAvxxcT3aE0MHuY4xx5HCNFc/D/+Qr4pWYl+fujqjAAAXZtiCYjhK9C0iJtaQ0xHvy/a4uZLrR3L4tYrJMOnexLyXbAlSU+I4MAEnVrN8+YxQANWmKhUqVQsfdyyH6AAHdxyxVd1RFL00DXTWybmRfs1VpZFoHjLDEahXrXE7hLQD7fyPnv8O/3NF7JkhJYyC2pXeTf2AVWw9DMk+g5v9Jh57nYvxy8dJwZ6Tj2xJf8mkjLZ5sHlNqIYc3J8kJQLsEcpnPXtbGPXnIKJjdgfjwZ4X7Jx/OtGO9f/2WKE+89P7QtTqGHT/QMFOL7//2wzPlh3bBvbCfyXSN1QZxEmd8YfPyUExf+3NjUSdbl/O06Bfzx9cWoygE7lt0Qsw8E0/2L6+X20mq44qB+eOOTAnhuxjr1Mcz7SMqM8GaXXLJUWV1XrmCNVLM9F+tLfOh+I66tF9M9Sgq8RNNQUUkpAWHkR0xKEb+kJMB7CdhQNcU9DF4QZ1UqQ/dMbQoVJO3VsSfwTpFIVyeOydjlZ8vX4BYCYm7V2+2RnJ7/s+Ak0CM5GbH6e88DoUxvNBoXyKpCsyRZbBqTtqXsXcPYXnK8qQcvVQB8IBCCrDILEm1IXS6JBRLfTvXqBbLG2q1J48Fq8i6LG+8kUfC2EZNYqShKJtR1wO1wpL+CKWpMAOONxIb+0+u/QdYW9qs2u7v4ZTrQHKt98k9MkI6XnTjqMO0OPN7RBpvLtEcDZd4SCgCM8rBkrtqbr6h5oxQbWFBqDnMSQQ3ZwCrzjkPoo8RRlpqKsITAE0dHo33kQ1yK8CaIKwK0OF2e3OQN3sCtHAQeWbjpz9hxp2l1f4KtbFQGfP9gWcbdK4jEMKWVh4rRjKyjdcxGNmMo9N3DZRAjYGUPC+H9NTmJNqyl4OQq/U7R/p3MGw+rc/oOTJ+Hnya9kTxfabqK9ukODDtKBIDTSOPf6QhGyXNcuBfQmT+Ev9KUHp+Wq0hE3NwlsVSzF+X0vLDNxFXUF1Hn8i5EPY0u+c0yYTvDNLSIxhTtvAv2ZYkqM/pdkeJXLpGv8tPm+8y4r/AUo5lYzRK1LBRqNlcG2jPNLgcpLDHqrqoXQ5HK6QHzbRG1QMB1ScQqvSVVT3zMNL0wfQxuJqOrzO0YBPzxQ4vwBxkFx2N3AYWgqWmeLRjvCh2/hODEl+mIAkBIXhlJ8wU6xvqN+KY4lFvPaPxN5Ml8mnVN3XpWyJKZV18ZmAqxvC1yDoV7b2+2vzN+Ln/qzu5MNtWmftbeAtVmbwPe3y6XwjVOBvx78n8Rc9wuZkZCezI5WPf8xnoQiM8pZIqnNZq3/6I0hzVkv5gWBcyLmXJz9ry/ODbbk6+QL2LDqHtM7o+rXOw5z/hqP7jFk5EL6AIsNwWZJdRc1TmK4DEsVTIhhTh3W+WXY2BUvZhKxJULgY1K3wV17sV/HjYOzrSrkEkuSqAq0XmqfqiV/c/qIqVmqfYlDgx6nbnJ7i7W+YQ9k1mhBNHtwYzj2ZZNAm8g/Sz0ZC/XbVAUGIsy5WSq7FDFS+t2Uju8ruCC7OPo+qEzQrwhnm3/aVX6PIZNlfUjIKtFujl4IVjrYnDnp7F4hrrqfo9nRy56zUJLDzg75Sd5WzY5ke0XqmL7UVCQeGSHjuj8rvzA0CzvDXrXdxrGSxP8cvZhGZjXjs/6NbBRr9E5r6BDBtZQMYvGpzBnRIzNaNZQsI8TGWlKdvQ8BRErOiKX8nhw/X/9RdUUrd2n2YPy9X+3m11FydxnNF5Rtn+8gk3YdnzVWbv3LNKUZtX8k8XTFl1Nw/xTmPY0Kcr8LjdWXx24AGBCHl3FyWiN8VMWsX4Mkhaa4lN4CwVkIT0AgL+yttT8KMHbKiHUm8SL5cf3oYDpiHk41d+z6mOwNzfHctr3Tq+1Z7D1EJz+2M2flMW5cb9xkcHpjUh0LpaDt0ql5rNZXWKq3PVsE/rq6VdM2Wy5uXbl2K/7oKmADpJpUdW9c

Px8TZs2Tbt379aKFSu0ePFijRkzpqL6DwAAagizb8suZSrM2Gw2zZkzR4mJiZo0aZJuuukmHT
t2TC+99JKaNm2qiIgIpaena8+ePUpKStK8efOUmpqqpKQknzoHAABqh+bNm+uf//yn5zKWUuU
JODbDnytuKoHL5daRI8crtA2n067w8HrKzT3OeUwTGDfzasuYRUY2CEg9zP+KU7rfc17eaPqa
mQuiGmji4E7KyytQUVFJBfWw5qktn7VAzX9JOnHihHbu3KlOnTqddTuf72YCAADwx9GjR7V58
2bl5eWpUaNG6tixo+rXr+9ZHxoaes4gIxFmAABAFZg7d67S09P1P1yy+/SDp5OikkJES33nqr6W
tteQwhAACoVK+88ooWLVqkv//975o9e7ZsNpuee+45JSUlac6cOVVq2bJmp+jgyAwAAKtXLL7+
sv/71r/rrX/+qbdu2yTAMde3aVV68WDfddFO56+PIDpJc4bwNzSdy5ZdXua6
Hj16aN++fabqI8wAAIBKFRYWpkOHDpW5bseOHTrvvPNM1cdNdFWdf+rvvS0+WJEcdjhcjOpbbt/YsO
3r0qD766CM9+eSTuuWWW0zVx5EZAABQf7+97/LbrfrkUrp3brp3nvv1aBBg3TbbbbbbeZqo8jPOm6wzMeh2A2CS
keZqo8jMwAAoFI1adJEK1ZfKKl7Ke9FJMVGfVoXXq7IfZ5
BCdVVFWiVUxGBSrpX6FfE5OB5oDF6NQ5j9K9ZxpgIx42vZ6XTIS7wgzIlPcXbsPVSkfErewsmQ3Q
kSVOmTPGrLsIMAMAndrtNTqdvVyu43Ybc7mr9Nh1UoLvvvvuc28yaNavc9RFmAACmNAitI7fb
UP36IT7X4XK7dTT3BIGmltqwYYN+/2rI/Px85eXlqV69eurQoYOp+ggzAABTQoKdstttemXNT
h3KMf8i0KjGoRpyzaWy222EmVpq7dq1ZS7fu3evJk6cqP79+5uqjzADAPBJdu4J02/cBs6mRY
sWmjRpkmbOnMkTgAEAgDUVFBQoOzvbVBmOzAAAgEo1b968MpcfPnxYb731ltq0aWOqPsIMAAC
oVPPnzz/tAuBSzZZs3N3Unk0SYAQAAlWz79uJu2nLSsqKtLmzZt13FQN3bqm4Gw4G5rk98+OaGQAA
UOWCg4N1+eWXa+LEiZo9e7apsoQ9zWVRkWQmcWnvuzOGAABQjtdfz3wwww+mynCaCQAAVKrXX3+9
zOVLGfZQGIAyGJXnnnvuOeOe6li1b6oCEHGjGBXYYVHgVH4EGAABUqvfff7M3P+kxdM06F
H94x//UPfu3dWpUycnqNFa+VPwPB9NtzK4bdrzHYWg5vjB8+FxYKhjgOzFzxBkeqD+eQ4M1tzrpmptsdl
l2v8+PHGaO3euli9f9f7lPHAAABAzfT1t11+rT58+qk2tYp9tJJc7vYUcppN+FmWAd31AV8yv5y4HJ4GGq8hOiMqpn
ruFAAAqLlmzJPghp90padOmKTg4WP/4xz8kSXXq1NGTkyOyU/WVO8yEhxX+EyMpHh4frUeeu7ePZjyU/
3a11x8rOfT2r6Fezn7tRLvGDHEyXwwNiXhlD1bbbbb+v997/1NV2VHGhmOzExKlcWzFjWa2Q9TCWm3mWmmio
LbDabDMPQsWPHlJWVpYSEBDdmvtl2Gk5iYqDD9z9gnJ8dUpwAAQM21bd2sP0ZT48WzOHD9fLmmTP1
qIkSQcOHFBEERISpnj1910lmxj8nXOoxef6M86GDfszGDPzmP8Vo3R/bTabbdDabucKnbG667Cllau
uY17b9LktQUJDCwsIkSZdeeqnq1KmjvXv3ql27durcubOeffFZZU/X5fDFTxo0bdc8996ShPnz7
q3bu3Zs2apaCgIK9tgoODJZ18RLGv7HabwsPr+VzejLCwupXSTk3DuJnHHj1iDJnHmJUP87/iOR
x2O20O
c2Vsv/6nbDdftrRNqfaOeW3d71O1bNlS//nPf5SYmCi73a6LL77YE2aOHj0qt9qt9ttqj6fwszat
Ws1ZcoUXXbZZZ5HDoeEhKi4uNhru9Ebn3e3gmfy5eHw2FXWFhd5eUVyOUyN4
C1GeNmXm0Zs0AFEOZ/xSndb5fLrZISl6myLuPkOLnc5stK8oxzbR3zmr7f5Zn/t956q2699VZ
FRESoR48eatWqlTIzM3XxRdr4cKFiomJDdWm6TDz73//WzNmzFC/fv302GOPPeY7GNGnSRNnZ
2V7bln4dHR1tthkvJyzubaRgP8jBqIHIRhC8kmnKNy6ImL7ejVvb+v6CRd23YBVqEt7klyf3x89xw
081c+ZMlZSU6Nn9H//Ur6/cn5kFhxGWr6qpH37VkVi5+xDQ5b+Nn+Ppr8nzecdeVfNYz0EpwaUDYTYy9c+gd4Kn
28X0JCgpYsWSKXyyyWH4+Rhx8zMTMXEXxh1vQwAAKey222y281fq1PK7TbkdpsPYQicuLg4DRk
yRHXr1vXKEkFBQerUqZOaNGliaV6uYJyLzFOZPkOw8jNpnMj9m/7E6YLvsr
afTo0dqyzYsWL16stLQ0Ux0CAOBM7HabkDRV6IknnghDOM2vWr
NEvv/yi9957T++9957XXuqSkJD388MMcW52u00ou1
0vv7ND2UfMX08V1ThUQ665VHa7jTBTc7osmyGIahG2644azblDvMlF6mmMQ9TmM2fiGe7vzbmJuYH3s5
EAMAn2UdOaP/h/KruhqX4c3ou0KfmzvaiyD8LaJgBAADW5O/puUCfmjvTiyZ9RZgBKCG8+f0
XEWcmmvatKnn38ePH1d+fr5CQ0M0orB7n99YxrT9xeRX/mcx9XGZp9Uv3wsMHMmnFFxs02r9
HhNnjxZCQkJpurimCoAgpx8v7wKoCa4fPPPPD5v7wKoCa4fPPPR1UO9fYeMWMPuGmpkSNHymazafSo
qysrJM1cenAgBQqRqE1pHbbSgsrK6fG6/ToZhaOLEiXr
jjTfUrVs3zZ0711R9gnYCAFSqkGC7b9ajCn26pFyW3Zr5lYW2Q1YFMBGn3ILbo+Ohxq2ZTHbkYB2BCZzuzeX4hdNdPnp8LZi4xuaR6
c92QIUM0ceJEU/URZmmA6nS7HQCUly/6rLJzBtp6Wk9WFXk3Xwt
jsAAM6lbdu2rRokbp16MmSqZzzhiPMWFx1u90OAIBzmTHgUk8EZBqdbd4BjMAYL1/lt4a6OnO2PwK02
dq/PjxjxtlssSkpKUiLL75Yox4elHNLu0QwAAzqzjx4568qcUxsbWhWsNmlkoH0
RkZEaPHiwbrzxgUFBZmqjzADAAAq1fz5M82rNQfGnGC5vPhtaMk7CsXdG5gt7yqVjKxXqSlbqDpvalOCDTyPJcDK8gurC0UPZYfDGkWOl
dG1z6m3hvuA6w+qJMAMAFuXPNXO19RZz4vZRUF9ZbnGHsOZDY
S3HKa7AI8xUMQ4TA4A1+HuKShnuCoIYaaK+XOYWKq9h4oBoLL5c4pKPlH9kLQ3aKxrusZwiqsCEG
aqCV8Pedb2Q8UAUNl8/Xkd1TJ0ZRIQZAPATrxGxEgluhgrXxBfl7AgktAetrxQBqhZhBgD8d
qEWbg922GAKr2Vl/UHnxWykaYqcUCdZshd1IBvgnEHETtwGfl7AgztZi/txlGR9TT4P6tfb7w
Eajt/J2DPJqh9uCzcnZ+hZn58+crMzNTL774omfZjNJ07NGPGDG3dulWNGjXS87NGjXS8OHDNWrUKL87i
orj6+HxmjjopgMrGoxXQXnyWyubzSbR/etfevLJJdDy5cs1fvzXL4aGdoCOH5lZlcrmiLQiH6e4M8
uXL/e7oy0B1Ybfb5HTaffpT089BYHQ5o2bZ3omjyvttMNs36bNWuZmVbyaV6i0toUX/WvvqqgoKCPCH36dDm

dTsXGxmrfvn1auHChkpOTA9ZpoKoE4vUTXGcEAIFlOsxs27ZNDRs21Jtvvqmnn35a+/fv96zL
yspSQkKCnM7fqk1MTNSzzz6rnJwcRUREBKbXQBXx9/UTXGcEAIFnOsz07t1bvXv3LnPdwYMHF
RcX57UsKipKknTgwAGfw4zTWbGH5v19k2kg2rbZbD79tu4pYzN/DYs/ZUvLSb+ddjFd3Ob7U1
Olk0c4DMO3Z3P42nZpmcO5BTrwk/mL8Gy/lvd1zGqj6j7//ZnD/s7BKpv/p2xuqX5Xcdv+jFt
12O/qfJo8oHczFRYWKigoyGtZcHCwJKmoqMinOu12m8LD6/ndt/KoylveHA67nE6H+XK/nu5w
2M2X96fsqeXr1w8xXVY6GUb8DTP+PELen7b9/X75Oma1jZXmvy+fiUDNwcqe/w6bNftd5W37M
W5Vut+/hpjqfFt4QMNMSEiIiouLvZaVhpjQ0FCf6nS7DeXlmT+cb4bDYVdYWF3l5RXI5XJXaF
tnatvlcqukxGW6vMvt9vxttrw/ZU8tn59fqF9+MVe+dL9fWbPTp9M1UY1DNbh/a5++Z/603eq
icF3TNaZKxsxKAhVArDD//ZnDgZqDlT3/XYY1+13lbfsxblW637/Oi/LOkcr6BeRUAQ0zTZo0
UXZ2ttey0q+jo6N9rrekpHICxskfRpUbZkoZhm+nTDxlDJku70/Z0nLSyf9wfB23QznHfbrNs
LS//nzPfGn7vEa/HlGpwjGrbawy/32Zw/7OwSqb/6dsbql+V3Hb/oxbddjvqvw/8lwCegIsIS
FBGzdulMv1W+rLzMxUTEwMF//WYL7cqlydz70CAKwloEdmkpOTlZ6ermnTpmn06NHasmWLFi9
erLS0tEA2g2qi9PHaXP8BAKhKAQ0zERERSk9P14wZM5SUlKTIyEilpqYqKSkpkM2gmvDn8do1
/dHaAIDK41eYefjhh09bFh8fL/pTLSwmO9f847Vr+qO1AQCVhwsXAACApRFmAgAACApRFmAACApRFmA
ACApQX0AuDazG737fH43KLsH1/GjzFHWfx9nQGAqkOYCYBAvEkZ5pTeFl6dH68Na7DZbHyWAI
sjzASAP29S5hZl33BbOAKl9KiqL58lic8TUB0QZk7h72Hm7CPcolzZGHMEii+fJYnPE1AdEGb
EYWYAAKyMMCMOMwMAYGWEmVNwmBkAAOvh9sAAGBphkAAGBphkAAGBphkAAGBphkAAGBphkAAGBp
hBkAAGBphBkAAGBphBkAAGBphBkAAGBphBkAAGBpvM4AqAJ2u01Op7V7V+l3DbndRlRlV3AwBOQ
5gBKlGD0Dpyuw3Vrx/iU3m325Dd7vsLTf0p73K7dTT3BIEGQLVDmAEquiw0+c3tJe+nd3ft7
v7Uj6qcaiGXHOp7HYbYQZAtRPwMON2uzVv3fjW7ZMeXl56ty5s+V+6+65o+6//35ddNFFFgW4KsKzsXPN
vaC99O7u/b3f3tTwAVFcBP2k/f/58LVmyRA899DJWCuR0CgkrF8/Mf6ZP1u/qdOvVJFD9a1B9dcb2mw5N5RkRkVkRzXUFmgreAqdGkGu
9/N4bPC/SxqFc5NW6xBb5/xzUa4u3NPqx7PqqlXuDp9s0QyEdFuKBpPRGeeymG2nnPvd12LkeVCArmVxKmNE8x3DKhOtFnrOM0ruRoWk
2ctNGbDj0DMtJXyTxrxkf1wpe2tbTw+S3CPdIUdHxfmGuQd1d3tSOWdgXAvTLf6BwsUnOu/m0t
H+dGcW9oD0/4HG4oHDxkM1aNGWG4MX6K3atbdDd5z7Ue/6TcZe+f9+dbxaVlYPS59pi/BeBBvq
BNAQAASApwmDn//PMlSdnZ2V7Ls7Oz1aRJk0A2BQAIC0AYaZ169aqX7++++/vvf/3qW5eXlafv
27erSpUgmwIAAJAU4GtmgoKCgoKCNGzYMD3322GNq3Lix:xmjVrpkcffVRNmjRRv379AtkUAACApAp4
AvCECRNUUlKie++9V4WFhUpISFBGRspZFwUDAAAEQkBvzQYAAKhs1fc+KwAAgHIgzAAAAEsjzAAAAEsjzAAAAEsjzAAAAEsjzAAAAEsjzAAAAEur9WFm/20ZAA++ugTW1W1/li1bVtVdq5bOz5+v5uuv
OHHg0bpg4dnqz57KyMoot57t57KyMioot5VT2WN2d13333aZ6579+5MPai/lvDvPfPOZ/5Qj4Q/Os5uu
vv1ZwcLDWrl0rm+23t/M2aNCgNCSGHwM+Oy3NxcpaSkMJrzEqEVBMTkcGwtVcWVvSUJ2w0t/qqlSV
tLQ0NWrUSMnJzyJyVXY2+qhrDGTTn7ubr31Vg0bNsyyzzOOFwVHb3aj3mf/kx/81j/leeWh9mdu3ap
ZiYGEVFRVV1V6tQ4cOadqR4caNiYmK81r366qsOAgrS9OnT5Xbq3379mnhqsFRsbq3379mnhwoW1
fZ2cbM5XJp9+7dGjt2rCIjI6uoh5CY/+XB/DeP+V+V5av1ppq+//DDhg3
15ptv6rLLLLvIrfYs6vtrLLLvNal5WVpYSEBGDmdv+XixMRE7dZd3+Wrjm0xr96+ZzbrTTW+OFRcZPtlVdZZqMzR2zZ6Lxk2jd
/3Nj/pvH/K98HJnZtUuRkZEaMmu7dq4suukkXvEi7wh6ruWrXRu3dv9e7du8x1Bw8eV
FxcnNey0t9yDxw4oFKaBGaHBEBaKsUnVmeyDenwUs+ZEhVPmFlt/4
b8N4/5X/lq9ZGZ4uJxCcWAKze+45tW/fXrl6/mBFcrorooo4o0DzbqWTW8rJqFhqZNpaLlkRoIxgyW
JRUVFVdGlau+bb76RJA0fN2QhGe8FqX5rxk4TMfXvf3vl7+fOn06dOnTp0iQDU+d7fMxeo3/Fq3
ZoKCgrRhwyY5nU7zRn9l4jQkGlNnYb03sX+d1N/fH11ec0Dis50d1Scq8wNaemQjGw18hJJ8YP
14jR45UWFiYJCkuLk55eaAc1sLk6RkZG3eab9dVxx512WWwBovg/nvP+a/aH/12i+yZ4Jyfc73+ziIuLH6
FDh6qoR9bSpEkTrS9Cm1EkHtjWDnea0r/To6oquiIYdMqeaaWbWnn6g8ePFgVdXaq1mP/ehn/yn+l/Bx/vzUL
6zOzcuVMdO3a3ZWUaW1++vuHgiMbtMqoQWtsdl0ptBtBUtqr+8fGxCbjpmfrhX0PTp87UpdxI
r776SpL43SFx1amPRZlUQnRppeq+Ydhm4rqUnNvtyZhY7Cdh9IlyKkb7kgc5rHxeTMFLM2syX
UtITk5WFfn6XpbXx2zu/K1Zv5gHv00ZCnJfrLxFgh6bp29bvFTqgYlb4w4sPtaY+qEeffyeg6
tO3XXXcYDJWL++6z1I0bRmHsYDtqYr3l+frpRyFb2V8bWq6sSZgFwWa6gbwWGGXXEkIxq5Pnr
ZOkREhNLT0JPjbzVjKapmpSkpKuqubGssnp5lkkdnbCrJSkq5gkduPVfXY1SWiVBEESAkjRB
pKruWq3C/Pcf89885n9VI3bmGYSKVZ7oPrPYzGHAQFP/AAQ6yPMMAAASyPMAAAASyPMAAAASyPMA
AAASyPMAAAASyPMAAAASyPMAAAASyPMAAAASyPMAAAASyPMAAAASyPMIGAKCwv1+OOP6+qr1
a7du3UqVMnpaSkjUOHZ5tXn/9dQ0cOFCcOFt27fXr5bwn0HRvjjvu0OW
XX67LLrtMI0aaM0kvn0X0cbe6t0wvDFVFWkymg4BJTU3a6dqldunfvf//+8/t9ztvf/o7kW7U29
DMPQypUrNXzVq1HXq1D1nz57555/9npX5ZdX5+tk5X//8evfc4vd1/3Nxg3+X5KLN0V+3dth27ZN9Q4nN5//HG53W4NH

```
TpU3377bRXuHYCzYf6jShlAABQVFRl//etfjf/7v//zWr5o0SIjLi7OOHTokNGzZ09jzJgxXu
ufffZZIy4uzli+fLlhGIYxe/Zso3379saPP/7oVXefPn2M8ePHV/yOADCN+Y+qxpEZBERQUJA
yMjI0cOBAZWdna8OGDVq6dKk+/PBDSdLevXt14MABXXPNNV7lrr32Wq+vMzMzdemllyo6Olol
JSUqKSmR3W5X9+7d9dlnn1Xa/gAoP+Y/qpqzqjuAmuM///mPZs6cqe+++0716tVTq1atVK9eP
UlSnTp1JEkRERFeZSIjI72+Pnr0qPbt26e2bduW2UZBQYHq1q1bAb0H4A/mP6oSYQYB8f333+
v2229Xnz599Oyzz+rCCy+UJL300kv6z3/+4zkvnpOT41Xu9183aNl19+uVJTU8tsJygoqAJ
6D8AfzH9UNU4zISC2bt2qoqIijRkzxvODTDr525okRUVF6cILL9R7773nVW7NmjVeX19++eXa
s2ePYmJi1L59e8+fN998U8uWLZPD4aj4nQFgCvMfVY0wg4Bo27atnE6nHn30Ua1bt04ffvihx
o8fr48++kjSycPDEyZM0Nq1a3X//ffr008/VXp6uubOnStJsttPfhRHjhwpt9utkSNH6q233l
JmZqbuu+8+vfDCC7r44ouravcAnAXzH1XNZhiGUdWdQM3wzjvvvaN68efr+++/VsGFDdejQf/
v//0/DR8+XPfdd5+GDh2qpUuXKiMjQwcOHatq0aXrqqad09dVXSzp5yPrxxxx9X
ZmamioqK1KJFCw0fPlw33nhjFe8hgDNh/qMqEWZQaVavXq02bdp4/Yb10Ucfafz48frll19UU
nXrKuwdgIrE/EdFIsyg0vztb3/T+q0mTJun888/X3yt6+Tq+q0mTJun888/X3r179eSTT+qiiy7Siy++WNXdA1CBmP
+oSIQZVJrc3Fw9/vjj+uSTT3TkyBGdd9556t+/vyZMMmOC5hRNAzcT8R0UizAAAAEvjbiYAAGB
phBkAAGBphBkAAGBphBkAAGBphBkAAGBphBkAAGBphBkAAGBphBkAAGBphBkAAGBp/x+XaKFw
0n+TYgAAAABJRU5ErkJggg==",
      "text/plain": [
       "<Figure size 600x600 with 4 Axes>"
      ]
     },
     "metadata": {},
     "output_type": "display_data"
    }
   ],
   "source": [
    "df_virtual_2_time_points['trt_grp'] =
pd.Categorical(df_virtual_2_time_points['trt_grp'], \n",
    "                        categories=['<=30 days apart',  '>=90 days
apart'],\n",
    "                        ordered=True)\n",
    "df_virtual_2_time_points['age_years'] =
round(df_virtual_2_time_points['age_at']/365.25)\n",
    "\n",
    "\n",
    "sns.set_theme(style=\"darkgrid\")\n",
    "\n",
    "g = sns.displot(\n",
    "    df_virtual_2_time_points, x=\"age_years\", col=\"trt_grp\",
row=\"gender\",\n",
    "    binwidth=1, height=3, facet_kws=dict(margin_titles=True),\n",
    ")\n",
    "\n",
    "\n",
    "custom_colnames = [\"CTRL\", \"BB\"]\n",
    "for i, ax in enumerate(g.axes[0]):\n",
    "    ax.set_title(custom_colnames[i], fontsize=14)  # Adjust the
fontsize\n",
    "\n",
    "\n",
    "g.set(ylabel='')\n",
    "g.set(xlabel='age')\n",
    "g.fig.suptitle(f'At-home program', y=1.06)\n"
   ]
  },
```

```
  {
   "cell_type": "code",
   "execution_count": 20,
   "metadata": {
    "metadata": {}
   },
   "outputs": [],
   "source": [
    "my_ctrl = ['<=30 days apart', '<=60 days apart', '61-89 days apart',
'>=90 days apart']\n",
    "bb_init= get_one_enroll_type(df_comb, ['Initial', 'Initial New'],
['<=30 days apart', '>=90 days apart'])\n",
    "ctrl_init = get_one_enroll_type(df_comb, ['Child CTRL'],
my_ctrl)\n",
    "df_init= pd.concat([bb_init, ctrl_init])\n",
    "df_init_2_time_points = make_df_2_time_points(df_init, ['Initial',
'Initial New', 'Child CTRL'], ['Initial', 'Initial New'], 1, 90)\n",
    "\n",
    "df_init_2_time_points['trt_grp'].replace({'<=60 days apart':'<=30
days apart', '61-89 days apart':'<=30 days apart'}, inplace=True)\n",
    "df_init_2_time_points.loc[(df_init_2_time_points['enroll
type']=='Child CTRL') & (df_init_2_time_points['trt_grp']=='>=90 days
apart'), 'trt_grp'] = '<=30 days apart'\n",
    "df_init_2_time_points.groupby(['enroll type', 'trt_grp',
'time_point'])['user_id'].nunique()\n",
    "\n",
    "df_init_2_time_points['age_at'] =
pd.to_datetime(df_init_2_time_points['test_date'], errors='coerce') -
pd.to_datetime(df_init_2_time_points['birthdate'], errors='coerce')\n",
    "df_init_2_time_points['age_at'] =
df_init_2_time_points['age_at'].dt.days"
   ]
  },
  {
   "cell_type": "code",
   "execution_count": 21,
   "metadata": {
    "metadata": {}
   },
   "outputs": [
    {
     "name": "stdout",
     "output_type": "stream",
     "text": [
      "Chi2ContingencyResult(statistic=1.8408452811848117,
pvalue=0.17485190743884685, dof=1, expected_freq=array([[ 61.3916501,
98.6083499],\n",
      "       [131.6083499, 211.3916501]])) \n",
      " Chi2ContingencyResult(statistic=0.29060083476050125,
pvalue=0.589835758308618, dof=1, expected_freq=array([[ 118.13996033,
254.86003967],\n",
      "       [1318.86003967, 2845.13996033]]))\n"
     ]
    }
```

```
   ],
   "source": [
    "print( stats.chi2_contingency([[54,106], [139, 204]])\n",
    "      , '\\n'\n",
    "      , stats.chi2_contingency([[113,260], [1324, 2840]])\n",
    ")"
   ]
  },
  {
   "cell_type": "code",
   "execution_count": 22,
   "metadata": {
    "metadata": {}
   },
   "outputs": [
    {
     "data": {
      "text/plain": [
       "gender  trt_grp        \n",
       "Female  <=30 days apart     54\n",
       "        >=90 days apart    106\n",
       "Male    <=30 days apart    139\n",
       "        >=90 days apart    204\n",
       "Name: user_id, dtype: int64"
      ]
     },
     "execution_count": 22,
     "metadata": {},
     "output_type": "execute_result"
    }
   ],
   "source": [
    "df_virtual_2_time_points.groupby(['gender',
'trt_grp'])['user_id'].nunique()"
   ]
  },
  {
   "cell_type": "code",
   "execution_count": 23,
   "metadata": {
    "metadata": {}
   },
   "outputs": [
    {
     "data": {
      "text/plain": [
       "gender  trt_grp        \n",
       "Female  only once              0\n",
       "        <=30 days apart     113\n",
       "        >=90 days apart    1324\n",
       "Male    only once              0\n",
       "        <=30 days apart     260\n",
       "        >=90 days apart    2840\n",
       "Other   only once              0\n",
```

"   <=30 days apart   0\n",
"   >=90 days apart   2\n",
"Name: user_id, dtype: int64"
]
},
"execution_count": 23,
"metadata": {},
"output_type": "execute_result"
}
],
"source": [
"df_init_2_time_points.groupby(['gender',
'trt_grp'])['user_id'].nunique()"
]
},
{
"cell_type": "code",
"execution_count": 24,
"metadata": {
"metadata": {}
},
"outputs": [
{
"data": {
"text/plain": [
"Text(0.5, 1.06, 'In-center program')"
]
},
"execution_count": 24,
"metadata": {},
"output_type": "execute_result"
},
{
"data": {
"image/png":
"iVBORw0KGgoAAAANSUhEUgAAAjMAAAJ+CAYAAABGlIFwAAAAOXRFWHRTb2Z0d2FyZQBNYXRw
bG90bGliIHZlcnNpb24zLjQuMywgaHR0cHM6Ly9tYXRwbG90bGliLm9yZy/MnkTPAAAACXBIW
XMAAA9hAAAPYQGoP6dpAABfXklEQVR4nO3deXQUVcLG4be7swOBhAlBEWTRELawDCCbCKyiW
NER8EwgsCAKItKEAU/QVlUEAQRRQmKjAIiiCOyCIqOgyDbDCKLuAAqW5AkBMhm0vX9waSHNgF
S3Uk3RX7POR7pqrq3bt/uSt7culVlMwzDEAAAgEXZ/d0AAAAAbxBmAACApRFmAACApRFmAACA
pRFmAACApRFmAACApRFmAACApRFmAACApRFmUO79+uuvql+/vvr06ePvppSJvLw8v8v
fXWW/5uBgCUGcIMcJnr06ePZs2a5e9mAECZIcwAl7nj/x4/7uwkAUKYIMwAAwNIIM0AxCufRvP
zyy/r0090911113KS4uTjfeeKPGjh2rtLS0EteVn5+vN998U7N998U7fffruaNWumdu3a3aKTk5Wb/88ov
bdoZhaOHChCUpMTFReKjzi4+M1ePBg7d692227r7/+WvXr/+vGIHEq1pFIIuSUWqiMcBw+o1UhaNIIOCRmj9oi7n47ggNt3J/tpVfNJ2b/bPaPt5+YQq+HoYQ4BmwoTl3nftoeh23ioDPn1VAkoS7H5ffk/2ewWoz5U2fFuTRW6/k4/z7Kv6+f/3v9lAzq2zZQv/ZPKXb8gVz1IdXdjiNq0t4TrHGXT5SvXYWPfHvkFnSg9xq8P0pvrj1Ttftc/eLn1LkN3jwqSn5SmHD9Pz1RLMv+TnvLezXL7Ijzk4m/PnmtdGr0fnuxNqeBkqj6zRoa6U8zzdfPnTQNXbZLc3ep3xpFx5v+5yp5DsJhrx0g8a60vtt4/K/KUd4lL8z9ucFEIXt7yvmEkFQqNOGvrn6UOLs0DfmwUZW/hRB8QL4mnpZZwZ6T7Vz+OOjS6TVoGaqOBh++0Hte+5MrrHVyb6fwGGjw5X2+qoCFqb75O6wgHlgJtvdoItJUD+GS9fNUG3Pq3XI9CMUf6PK3QF1eKCJqcQ4KJHEuImOIk4ByXHHsU5RDpRHwWFYQBE1sgBXBqHMPMqpeoF3vf+zX+tjP8fGwgDwdgzKyw1vb/xw5NuQ5e1ezSjiIgT11KFyxAODsdE9Zvcbb6wEeKu8pXl/B8Xiu39i77CnIwUiqNRFXAl9kiiE+RkB/cWTAKxd/vSRQs5UA/IhOPwWhsJUefV3xAPiNf0GVlEe3+bGwTn+fyi89dEJ1eSYK6Xh5Jk9UtqNN8yNTaSQnJ91/qFs85lrzLSXENN29/aQ18ZE2+gpvkszOYbDAHrpvPj1LMwY4JQuyNiVTtEXfqEhVBM0wbFkGdC5W/OFG/aBRC+H6ae8s0nNqFqsNZO71b
NzVo0ECSdOrUKfXu3Vv79u1Tq1at1LlzZ/3yyyyy9asmSJvvzySy1atEjVqlVz29+DDz6opk2bq
m3btgoLC5PNZrvge58+fbp++++OEH9ejRQw6HQ2vnMNGDDz6oiRMnvWrNGX0iRMnqmfPnm7bTpgwTNTpQ0HR0frX/

+qX3/9VTVr1tRPP/2k3r17Kz09XTfddJOuvfZa7dq1SwsWLNBnn33m1sYDBw6oV69eysjIUMe
OHVWzZk2tX79evXv3ltPpVPXq1Yu0r7j3s2DBAk2YMEG1atXSbbfdpsDAQO3cuVOfffaZNm3a
pNWrV7s+L+lsIOzdu7euuOIK3XPPPdq2bZtWr16ttLQ0ZWVlKTc3V4mJifr++++1fv16paam6
v3335fdzt+RQKkygHLul19+MWJiYoykpKQiy2JiYoyVK1e6lufl5Rndu3c3YmJijIMHD1607v
fee8+IiYkxRowYYeTl5bmWL1++3IiJiTEmTJhgGIZhrFy50oiJiTFGjhxp5Ofnu7WjZcuWJdRtu
2bV3lN23aZMTExBgNGjQwtm/f7to2MzPTSEhIMOLi4owzZ864lnfo0MG47rrr3No1btw4IyYm
xli4cKHb8s8++8yIiYkxhg8f7lr2+OOPGzExMcbDDz980fdrGIaxdOlSV/v+/e9/u5b//b//PPPR
suWLY3rr7/eOHnypNu2bdu2NbKystzqSUpKMmJiYoylS5e6LZ8zZ06R9gwcONCIiYkxPv74Y9
ey3Nxco1evXkU+2/O9n9zcXKNFixbGrbfe6tZ/hmEY48ePL9JfhE176KGHDKfTaRiGYfz+++9
G+/btjZiYGOOee+4xcnNzi2z//fffl6gfAZQcfx4AF1CzZk117drV9TowMNA0qT2yzzw5qqp66Svfee6+OHj2qD
Rs2uNUdHx+v5s2bu15XuEaBzdejQS1atNAnn3yi06v58ZNtdnn52v58uWuEaBzdejQQS1atNAnn3yi06
dPu6279dZbL/p+z9WWzc1a9bM9bpmzZrq06ePFJTev4eOrecsXOxXLeNuXn52v58uWuEaBzdejQQS1atNAnn3yi06
Lli115513um07YMAA1alTR2vXrlVGRobS0tL05dfqkWLFurWrZtru6CgII0cOlICoOfK87fvj+yko
KNCzzz6riRMnKiwszG1dQKkKCJBV7euuvf/2ra5QqICBATZo0kXT2NFRQUJBru6ZNm0qSDh06d
N42AfAMp5mAC6hdu3aRZZUqqR8VZ3Ug+tF/Pdd9/piiuucDs1IUk2m02PPgKR6cAE93M/wyFwwUHIb5ZjV8z8wKgInZPIqY
55p0gd+/fvlyTt2bNH7du3L1Hbfv/99/O2f/vvlJZb3kb79OZIXd8MwvGo2/vC0dUU5NQ6Yw3Fu7R1zzd2+5
dd51r+VVXXXXeOovTsmXLIssKf9Hv3btXt99+++3nr3rt3ryS57b+Q3W5X8+bNtX/u3bt0+5
ublyOp1uwalQ06ZNIFRBQ/I+5P+4zNDTUFYb279/DTUFYb279+vH3/8UT///LP/27dunr7/+WtZ04Z/dPXVV
7u9LgxCf6w/ODhY0oU/GwCeIcwAF9AF3DuX9aF/jhXXZNmyZUmyZUX+2m7QoIFuueWm6k9u/+tNF93
Pq1CnL5+df8H4wJ0+eLHHbhPNMzpWkzMxMSdJPMSdJPP/1kan8hISHn8hHSHn3bY4fwxkhQVFSVJRUZ9Cn/
RFypcX7FixWLrLpwrk52d7d7Wpncf3scDgUGRlZbB3FvZ8tW7Zo8uTJrsm+ISEhql/vho3bqzD
hw8X26/njjiidq7jPB0DZIMwAXvgggw+0efNmt2WJm+wJmfw6rV7VFBYWpjW7RbLisry/Vfr
CpUqFDk9EtZqa3Ok7qe7e+tTy1VtYy209OTk6RZadOnZz2Pa7PrCABMREa
GCggJJRQNSofP1/x8dOnRIAwcnwHaKVVwAMoJPTOHoLSOVgdu3jTJtr9/1Rn2N1JzJoe2Wh9dVAzJc
WLFhw3nUxMTHatm2bjh8/7hqVKNSmkrrVVFqQPHpOH3Ih2N1ZYtW/Tbb78VGWFYv369bvp7
166KjY3lur1169ZVUFCQdu/leLcMwiow0vfXXW8rKylKvKv5xROH3Hh8xX6++eZYbde7c2v/9b
0lSXFzcBcsWfs/t27cXu3r1q0kDxmUOZdFnpm+++abdlj/88/0ZbhzatWnKMaC
TkInOJfvjhBOkxHvEC4D9MAAbKO0ZP3y7DMDR16lXTX/+L+qtrMnFiYqIMw9CczzZs7
rNhcnNTVV48aN05w5c857OuNiAgIClJ+f73odf2f8fbF5Pdtv3660661wgsv6P33
31flypUh9+h9957Tz/++KPr9cGDSBzV//nxFRUWdNYdWdeeWFyxbo0YNtWzUsiDtSSJUyc
Oj7779XhwfAB4erujqhoaIVu3VpfffWVvvjiC9d2eX1mjJlSonbnwbW3ja6bfffnNbvnfvXlc/nd
uPAC4djMwAZeiuu+7SSJXhIuvp7Lxl+uk7777TDTfcoPGfGjjmnNmjWQUaOGGxXJwYXHWXeo1KhR2r59uyZzP
fffedbrrpJuXn52vVqlXKyMwQiBEjikw0Lano6GgdZ/HdPHhQo0aNAmNWXcnmLtePRncWWaRqv
nqx169apSZMMmOnbsmD755BM5HA5NnDjR63uh12/eUvf1GXLl1kGIY++eQT5eTkaNasWZFkR
s8884x69+6w6HfStsWPHavvXaXq1a77zGzevFk1zctTQ2LFjXduOh9ZzEHdcsstio6r1o1r/+9S
+lp6e72nTxHTp0O4snaPg5+7uUq1atxTw4EGtX7/YPRB6IsKPDs84UKKYYYmQHKkHMPh0Ku
vvqoRI0YoJydH77zztz2q2jTq2Pe79dCxcudlI1+2Gw2zZw5U2PGjFISFIsSIiWLFmivatW6ZzwrrtHL
L7+sBx9980OM2JCcn69prr9XXklSv14YcSpIiYWI3nvvv6YEHHtDRo0oe1YMEcbd26VR06dix
YuL3KDPE4MHD1afPn20of6fv16rVmzk2bNtXf///53xuuLqROnOnTpaunsSpevbsqe+++/05/
jwYT3wwANatmyZ2wTjunXrauHRrp2+RaLLVmyRFdffrVrRKUk4Sk6Olpk6e1j88mvvvsEz3
atEnvvPOO9u/frz59urWqWqUqWKvqvzyS041AZcgm8GRCaAULVu2TE888888Ykcs3WeeOIJ9e3bt8z3
53Q69csvv+jKK690u5e5PJP3yyy+65ZbyI8oKt40bN67N6M2wLAPxiZAWBpNptNd9957957r0u
j8pKSmSpBtuuuMEftTQPgI8yZAWBpNptN9957r+bNm6fbb79dbdu2ucPh0Pbt2/Wf//2xHN9107
p06eLvZGIoQ4QZAJaXnJysunXrasmsXrasmSJfrrggw+Un5+vq666699et30YdiArA25swAAAB
LY84MAACwNMIMAACwNMIMAACwNMIMAACwNMIMAACwNMIMAACwNMIMAACwNMIMAACwNMIMAACw
NMIMAACwNMIMAACwNMIMAACwNMIMAACwNMIMAACwNMIMAACwNMIMAACwNMIMAACwNMIMAACwN
MIMAACwNMIMAACwNMIMAACwNMIMAACwNMIMAACwNMIMAACwNMIMAACwNMIMAACwNMIMAACwNM
IMAACwNMIMAACwNMIMAACwNMIMAACwNMIMAACwtAB/NwDWtn79ei1btky7du1SamqqqKlSooCZ
Nmqh37966+eabXdu9/PLLmjVrVrVonqfPjhhzV06FCNHj1aH3zwQZH1NptNftSWVI0aNdS5c2cN
GDBAQUFBrvV9+vTR6mnn+qqq67y/k0CMKW4Y9dms6ly5cqqX7+zu+vTpo06dOrnWFR6z5
3I4HAoPD1dcXJwGDhyo+Ph4n7Qd1kSYgYUdUdnd0z6tJ598UvpRmvWrFGjRo02Ro105513qlq1
+uBx98UA888IAef/xxSVKnTp1mp1Uq1UtYttzomT56s9PR0vfDCC27e7L69ev7/Z68ODBBqlu3ruu1Yg
6cuSIPvzwQ82YMUMXNOJ//vijXnXnzxxTJpxY2pt6JMPd9sNKakaM8TTTGN+wS27bDE2srpt/8QT

TygiIkKSlJeXp99++00ffPCB7r//fs2dO1etWrXy+XuARRiAB4YPH27ExMQYc+fOLbIuNzfXS
EpKMmJiYoz33nvvvHV06NDBiImJOe/6xx9/3IiJiTE2bdpU7PqcnByja9euRkxMjLFnzx7X8s
J9//LLLybeEYDScqFjNysry2jbtq3RqlUrw+l0GoZx4WM2NTXViIuLM+66664ybzesizkzMO1
f//qXVq1apc6dO6t///5F1gcFBWnSpElyOBx6++23y6wdwB/yxJ2rJlS5ntB0DpCQ0N
VYsWLfTbb78pLS3totttHRUWpfv362rdvnw9aB6viNBNMW758uaz5nPp2bNmvroo4909dVXl
2lbwsLCyrR+AKXv119/VZUqVVSlSpWLbltQUKAjR46oTp06Zd8wWBZhBqZ98803CggIUNOmTS
+4Xb169cq8LevWrZMkNW7cuMz3bcCcCU6dOuUZfnE6n0tPT9f777+ubb77RM888I4fD4bZ9ma
ma/uCggL99ttvmjt3rk6cOKEJEyb4vP2DwQb4vP2wDsIMTEtNTVWKlXcriAqS+f+f+QJTOTiQ8evSo3n33
XW3atEm33HKLmjdv7pO2AAC5hx56qNjlcXXtQTBTzVvSQjxlVvZtnT0/c9fPzxvHOuUZnzx3
CwiP99n+zvfD8QqVaqof//+GjZsmM/ajQDkHn/8cWk2aqmFAPi2ha7MB+Ai0clw+2Ry9pglf
58RUZGurafMmWK/vSnP0k6OzKTkZGhDRs26J133tHmRXrs2gTmJ8Mz05rwo2tSAH+9kaaCn+Pbr3A2R9ftwZ4tG2+6Z7Mz+KBWsjw5kzhXJKZz9lTHCxABu24HAziRHWRwVFt5C1kcfM08yrIoiIbWAJfLMeMfqnm1m9nDrNG3xWrlJpUExJU/N+cEm0Tv2Ehxq4xnj82yVodZA0PxoTjSz
pDHA9edTi6e6zbQWbk85z1SCXXkbn5lfY8lyBy3cd7ofhLwUDc5F2pfjmmqsXb5bPwm2E1lIXx1YAE1KeUhIb4LVG4a/WLw4Sa+Gk36Ix+B4Z6F/E/Z6bdXz2lVm2tS7a1AEBWtm1QQoojDHyOsmYjtDq4k3oQjRsOLpAtkpyTsqSLvSm3
8Ij5LsS1bjMSc/E+zaedh12GMxYnpmUjwaXI+ASBnfT1fn65LaO2IDdX5S0yoHLXeFD/ELh9Xpxq3t2TgkD7aVp5zKC4i8V5p8cDpWr3Ou5uNxMPb7XIyz7G54/ZbNNDDpg4B8w2o8xVwJ9Jj
Ek6efKknnvuOX300Ue69dZbFRkZqQMHDuiDDz5QWlqa7rvvvPt1333l1obExEStW7dO69at0+
TJk4tcujl9+nRVqFChSLnQ0FA98cQTZdYAGd99dVXOnr0qOt1Xl6eNm3apFWrVrVsnKK68sctP
NdevWuR5nIEnZ2dlav369Pt/8WzIIBnoiZ0f4hRL2+NvkMYL2DOeXSP1NoezKnLubsvlsjtQqkqxu0psPgvZ5WNAYR+bO/1u9
WLMjz69uJLh74SzpNyFFdxbnYR81fsh1s+yo+J8pAsvycdMCuJaYvqqDKkvjv+ocRbFDZDMrW2bXU+GQ52s/iAmrZYjDLR4JYk0gNQX4jgrHQmQSE
26vQ0NDdeWVVyopKUkDBw50Cy7S2ee1FbLZAoNDVWtWrU0ZMgQ9e/f32e3g4D12AzDMPzdCA
AAAE8xZwYAAFgaYQYAAFgaYQYAAFgaYQYAAFgaYQYAAFgaYQYAAFgaYQYAAFgaYQYAAFjaZXHTvIICp9L
Syu529na7TZGRFZSWdkZOJ7fluRj6y5zy0F9RUZVKpZ6yPtal8vF5lCb6y5zy0F+ldbybwchM
CdjtNtlsNtntNn83xRor0sLn4c59Jc59FfZIMwAAABLI8wAAABLI8wAAABLI8wAAABLI
8wAAABLI8wAAABLI8wAAABLI8wAAABLI8wAAABLI8wAAABLI8wAAABLI8wAAABLI8wAAABLI8
wAAABLI8wAAABLI8wAAABLI8wAAABLI8wAAABLI8wAAABLI8wAAABLI8wAAABLI8wAAABLI8w
AAABLI8wAAABLI8wAAABLI8wAAABLI8wAAABLI8wAAABLI8wAAABLI8wAAABLI8wAAABLI8wA
AABLI8wAAABLI8wAAABLI8wAAABLI8wAAABLI8wAAABLI8wAAABLI8wAAABLI8wAAABLI8wAA
ABLI8wAAABLI8wAAABLI8wAAABLI8wAAABL8yjMLF++XMaWvrVq1UqtWrVj2hrHVwOMj5bc8uPtgw8/Lh92jNn3uuyrzsOynbWc0p+rHJWKKJFnvMcStrqzRk6
65557tGLFCnXr1vvvfqo+u+kUF3pEBvTzpVPVJdRpUg0dQ9lQzZsZZsZQ0qVLXeVnz56tRYsW
acKECVq8g8eLFsNsYDhyovLy8ovUn9vTCkhg5L9ACtgfAgws7FhGJoxY4uuv/9+3X+3QX/fYGhxEb8VZN/erVy6Sku9+HPxvl7Mf+G1Ilkaq5cm56Mn1H
Zu1efNmBQUFAdy4cQoICFC9evV08OBBBvfHGG+rZs6fy8vIu0I9b948JJ3cnq127dpk2dpnDQnd2bNl
q7dq26d+9e+u8QAABc1kyNzPzZ00086dOiQevTo4bY8JSVFgwYwN0tatWxUfH6+AgP9PLpEHe3ISEBO3
fv18nTpzQ3r17debMGMSUkJLjWh4eHq2HqzHDHtqyZYuXbUU5HHvHHJUAAJRHpsLMgQMx37zezq+/+/bR2rxz
Rt199367LPPJElXHxjxx5V9erV3cpUUq1ZNkZGRknR2ZkJNvUhUmaLh4AtmsMIozbIkG2Gp2G1On7ZYxg
Pr0aUnS448/rocfcfnvCsf/lgjR47Umj/jvVrNTGTIELX355pvKKzcrrKczsyxWp2G1Onj
zp8ZuQpICAsrswy+Gwu/0fF0Z/mUN/mVOWx7rE52EW/WUO/VU2TIWZwMBASVL//v2VmJgoSWr
QoIF2796tN998U6bhy9sIFCVm9KRkIUMm8ubm5kqSwLSCFBISIknKnKy8tz/btwm9DQUI/fhN1uU0REBY/Ll1R4
uOdtLI/oL3Por4vz1bEu8XmYRX+ZQ3+VLlNhpvAUSZ/Uky7UMjNvya665Rp9//rlq1Kih1RUt3WFr
6Ojo5Wfn+9aVqtWLbdtYmNbf+v5xO5mZWR6XvxxiHw67w8FBlZmaroMBZZvu5XNBf5pSH/
qtAFLWx7pUPj6P0kR/mVMe+stsXf3Dzi1SYadiwoSpSp2opKUkpKCmpqqKAdO3bdv+vbmeVtMj
RokUqKCiQw+GQrC/GQJG3cuFF21tRR1apVbRHuaoEaWvqtWmJMmj
oMDpk/1cLugvc+ivkvFVH/F5mEN/mvVMe+ysvL0Z2dmqXYMUVERChI
UE1a9b0qG5TYzeR9PZb7BjG7KCGlNZbb+maa67R3JMZXyS4mNm+XAVuTRp9Ni6RBNKNDDFu
DffPON5s+fr/Hjx0s6lcmKSlJU6dOVWGfdHPTKjbtFTmkK6j/R4L/zoL7p6cDq2vrK0tp7UD6k8/22YmMOl99NQcSqKm1YWqAuKkEyTQbP0WyFE/hjL4cV5n5SU1oTSK6qyxFbVG+CMnI
F1l15hJn95Wk/Kc/tQ5yvHDBGAmbmTpTTZaNU4rCmUFK/YpPb63vWkDqUxDkAEZaDepSUEOJJ9J7rJ3e3efqxkYV3Blj20/VW4+yyOWRxB2p5+bk1zqRqTQH5GTTiZFlEtTaKKPYIOW71yytHoBcEXmdQRpAlSE3rlJp0mwaS1T43mJoETNKE9Uu32Ax
dDKwnYZNEYpNV9OYhymUGFjWHFSF+Ty/WtyL6UfppC0Y3+Ijh0kT9a7I+XM4yLhFSXo9RxLr0xVoEO0yu5uqIqwj0J1vH0EzWbMm+z5GDBTPyfVDD9Ihaab+uUmxlfFH4aCNOHbHGt6E/X4vT+kp9/uCGUzgJyXo
SRH+ZUx76q7jj/cYbb9TkyZPVvn173XXTTTerXr5/69fZLuUlBS9/fbb+uKLL0ztk2vDAABA
mcrOzlZYWJiks7d5F7GxxD2f5Od9FP7GxscrIyDBd2P2EGAACUqZo1a2rbtm2Szi1nPPl2uxrVqhly
5am6zc9ZwYAAMCMXr16afbs2dqxY4fo2elzfszAo0ZfMRHX2zZfiBBZSxDMkFqqcx7OeaEeHqw

YZ0/UTZgAAQJnq3bu3MjIy9Pbbb7vu+L9+/XqtX7++yLajR4/WHXfcYap+wgwAAChzQ4YM0ZA
hQ5SZmamsrKwil2V7gzADAAB8Jjw8XOHh4ZKkM2fO6PTp06pcubLbY47MIswAAACf2rBhg6ZN
m6bdu3fLMAzZ7XbFxcXpscceU3x8vOn6uJoJAAD4zKZNmzRo0CBFRUWpb9++stlsGjBggLKzs
9WvXz9t3brVdJ2EGQAA4DOvvPKKunTpotdee0233XabDMPQ8HD9eGHH6p169aaMWOG6ToJMw
AAwGe+/fZb9ejRo9h1vXv31s6dO03XSZgBAAA+ExgYqIKCmLXZWRkKDg42HSdhBkAAOAzjRo
10rx585585Sbm+taZhiGDh48qBkzZqhDhw6m6yTMAAAAnxk2bJh27typp4cOHS5Js7hRSFsrVr
VahQQaNGjTJdJ5dmAwAAn2nevLkWLFiigXbt2KSQkrHIFxcYqKilKvXr101113KSgoyHSdhBkAA
OBTcXFxiouLkyQtXrzY6/oIMwAAwGc++OOCi26TmJhoqgHeJMwAAACfmTRpUnxXS
ZgBAAA+t3HjRm3atEnp6ekKDw/X9ddfr3bt2slms5muizADAAB8Jjc3Vw899JBwaN0Ngh8OhiI
gIZWRkaO7cuWrSpIlSUlIUHh5uqk6uZgIAAD4zZCoU7dy5UzNnztTOv15ZdfaufOnXr99dd
1+PBhTZ482XSdhBkAAOAzK1eu1IgRI9SpUye3U0pt27bVgq1ajr+ss8++8x0nYQZAADgM7m5uY
C3BkZKRHdRJmAAAC3Tr1k0Lk0LFizQ77//7rY8O//rY8Ozbb7zzxhu6++27TdTI+ExYWJi2bt2qz
p07q3xr1qSpSpYqOHz+uL774/L774QqdOndJVV12lJ554QtLZm+k999qpwe0aw+vFRqsU4XqG6WZA
XVV67lYWFhrqBT6I83lzsfwgwAAPCZTz/9NTrJMwAAACfczqd+umnn5SZmakwVaqobt26Htd
FmAEAAD61ZMkSvfrhxx4oRrWfXq1fXXvfxPx+pRv/bjmFgpr+rhx49SlSxeNHj1a
NptN48aNU/369Di+Bgn8HxOdKb+ox4ReUVNGnWAI+J9vVWamA4+6lzbBhw5S
fn6+xY8cqJydH8fHxSklJKTIpGAAAoKS8CjPfffed2+u+u4uDgtXrrz4vVNs7Ha4lAA4c1ybcrOTnZm90C
AIDLhM1mu+DrkuBCdwAA4BdXXXz5kV0+/7+e7HVb+AbW6/+gYKi2Ce1Ht9Ca9R4ubUt/CjatirzAf
hUaxWSzCheQ7NNBeFBVdexmDwnCi21UNAEYFiOIgfRNYOFBOPuCoKWjJFaUFChVUqeLmvZS8h2zTVkT2i
gU5R5pDWwlk5tKYERK0rkyHVTIu8RW3hNmqWGUOZJAMDeBkV/2GuiS7JsDR5ZJfVG0wXPdanPy2RIG3FQFcKVWk0c
s59szurHubJbTAtsAOvE/hcedGCzj+OjMhk7OKkTBJzBXQcMFCF9X9GwBsVt1evJgGd0cK7Itq2Bb0MT
qSnrfM4VgWpi3CrtCLTjDwAV+8Okpc+o/WSWABRdcRRBMMnqNZ1x80f9bLrKJZ9Bq9sPJMDp2Vri6qyHV
4TQXi8nAJ+vTaj+Aih3/b7lOEqazYcpjm+cYVYkNCaD/n8KB1ETaHcuGo1Jqbq0RJ+/16wCzrsx8rRwhj/N
EXzI8FcwsTlQuXbh7kCA/ehmTk0zkYuCgjyF0GjKjSU6eVbWjUlBfpOOXsXlfZaX4Q28mmnCAKbiB7q1
+8hOAhQ9Y3OQtxTXIuuP72A7GhZSJnv2Aj7o6pH0UEYSSQf90F5AobBg6FmlrOfQjeVwZe08OCA9uj
cWDfqrCNhZ3r1htXkIMjZMOcaRW0ALo59VRzGd2lccLFHyLuaeecJ0vzEfIXpvkCl09IzX0qpH9IuU3
AAFwgYqmIOzEO2AXyfU4xJsKQeFazDrvrgNyXCGlwp2qYAdOs2/YNm7YdfifYzfNudN7Knd/9oT0n
iR6/c8Nz+MdAgSIfc0dObh7ksXLwAjAQCQpiGxY0+U+tl0B6TsywKB/Mm5HjRcsE7f3HN/5ZuZHQg4
T4/c8HxH2oeSGhrNBa8G3GVX+qs3hXF3HqKPABXOMzBwWh/CdqsF1JTzI1jIv84wRsgtMoD/SRjb4
ZZKj+Pi4/72qFsS3HIaOAygyjJIYsiQM9Ln+tjrizP9VBGHZ2AdB/xR9I0RWf5ZUdSsI7DT2UgzdaIO
yhrtptYzTgOq7YFbYlYxbj1fQVtgRO2xYTIiTxUbiAEq7rvFmS/74RkS/yDbzwaS0+2+rjYGZ5E6dWcdA
jRFI9Wd5Z/FTR1eLTqwCFrN1OTeYYX8/PQNwpD+GQ4IxHxa2U+MlzQmzBzYNCQwIx9vCf/9qzO+Rv7aC
vH28hYKCuUuO0MOiwe2w8T+jo9u1MyhO7O0YPyr8OwRzL1w9KLNxKM3DFk0Myxr9nCq1NmC0zX5WwMNk
gKvMqR5ovy7TPSzW8F/IXbSOwSGRPXS9i3tKBXdl71EDuyWC7HMl09J9u0x5YKYOBwPS/wmDcPgKGA6wG
6fpNC3gKJYYnBIEXbRGxg5jmW8DQHh1ilRGgrM8ai1zWHFbgC9PhLz+b7NnlpBm5iuTxRVpHGQIgiVmc
oeYn6T1AtP0+Bim7xr6S/Yxlo0K8LMZc6lS0o9eLSQr05GQbExGbOfI8w2dwAwAlLMnwOdmD1ZXBwMpl
GsmCO+60LJ6O6hNAgkuhV4B9oWl4u6NG+OKlxcszAPVwDqSL19s2lLbDcTjGgTdcM2P5OXNDm55EEQVj
ZmFdk39H/Pzcy+ZZ22YS1OUgHRTXrZaVrPcLGFPe63oPGrPxcAMvfx85T5LW4TfrsBPXxd6JMp7JjSHr
BI/sbZbWz+2BjnWyw8jpY3npz5PXnTFzRbp/ry1EJ2XdDo9zFSxMyxMjdmCdQM2ll0kJ1EeHHfQpg2TKf
Qn3NJRPcYJZNVuBCYwo8ccMG+ptFpzyWbxPzOUA1qp7MX8g5OEwAU2vzp2OcvESwvLCs5Ob0+lwRM/F9
L7hDLOW4rI9WApP+K/ufqrnmSAA/PdhWWemwn5KVLCS0ApbS+cPCpZ2Il4IK1AR9WI9nBjnyRaVEnvGvy
XMzRyuHR2v8ajvqG1zYkMmhjrOL9z/WA7hz9FRNXP9z+KvUSOKBJ7WFFuhmMUu0JmQY2F6sC2XgqwfK
oDaZjh9IjkHS9R6bS1Ygv7V8NULc7H67GKyahu6ndk1nXTGV+Wm1h49pqxWFS2bwi2Xy+HVfM4ZEoTpf
u4Ko8R1yIJ2uDHTVMVVJG7fo2MfyzVJDPDF3DlhJYSHQ5zGLSwxQS/eLHyOWWl8cmVtRxXWXoiO73b1

cuRI2Ww2tW7dWjabTT179tSDDz5ouk7CDACUcyHBAR7Pe4mtHakurepIDNCghKpXr67ly5dr7
969+tOf/qT+/furWrVqat26terVq+dRnYQZAIAkz0Z1qkWGlVFrcDmrWLGirr/+eknSyJEjva
6PMAMAAHzmiSeeuOg2kydPNlUnYQYAAPjMli1bZBjuV82dPn1amZmZqlChgpo1a2a6TsIMAAD
wmXXr1hW7/MCBAxo+fLg6d+5suk6emg0AAPyudu3aGjFihF5//XXTZQkzAADgkpCdna3U1FTT
5TjNBAAAfGbWrFnFLj9+/LhWrlyphg0bmq6TMAMA8CueuF2+zJ49u8gE4EI1a9Y0fSWTRJgBg
EuGNw+K9DQQ+JO3dx7midvWtHv37iLLcnNztWPHj311FPav3+/ateubapOwgwAXAK8fVCkFX
lz52GeuH15CQ4OVsuWLTV8+HBNmzZNHTp0MFWeMAMAlwBvHhQpSfVrR6prqzqy2az3XAGeuI1
Cdrtdv/zyi+lyhBkAuIR4+os9KoHHRMIaPvjgjg2KXZ2RkKCUlRkKCUlRTExMabrJMwAAACfefLJJ8+7
7pprrrtEzzzzxjuk7CDAAA8JlPP/202OOXh4eGqWLGiR3USZgAAgM9ceeWVkqS0tDRFRESUyjyjv8
jNtHgAA+N13332njh07qlWrVrVuratat+/vlnSdLzzz+vd955x6x6M6M6CTMAAMBnJk6cqICAI0ZM0
bBwcH6v//7P0lSYGCgJk6cqCVL1piuk9NMAADAZ3bt2qXXJkyyfr1ltvvVVsWLXTXXfp9OnTevT
RRxUWFqZFixbp7rvvvNlUnIzMAAMBngoKCFB4eLklzkSBCAgMDDdeDAUnSddddp59++++sl0nYQZ
AADgM9dcc42cc42+/PJLSWdvkle3a+/kle3bl1XmMnIyJDT6TRdJ6eZAdOHWA4JEnGZcJ9WSiaXv
18bN25U3bp19cbbnsSw9v1htVSMOT94gMXFHHBgb4iIQbgdKxJg5zTJDPcXjOGbxnnxngE5
2EGQCAZXn6tHCn0+ABlX4SAxSoj2Kr1hH7KzQ0VPZZHqwaFBSkFi1aqHr16qbBjrhJMwAACynUligEE5
D4eGePZOqwOlURnoWgcYPpk+fXup1mgozGRkZmjZtj7//HOdPn9a7g9ezXvaa2r9aevX12OPPPabbr79
zx5NnDhR3377rapUqI+ffqof//+rvJOp1OzZs3SkiVLVNJmZqeuuu5u05PP/20OGBDNM0P/2O3rr766tJ9VwCAy
1pIcIDsdpsWrtmrYYfOmCpbLTJMvbs0U0NkNnkcwzB0O513XnHHm0HSNn0sc
Rt2jRFRkbq3XffVf/+uWDW/+/bVs2TJFRkaqX79+/uWWZR+/Hj95z//0fjx2XN1PlTkjR79mw
tWrRIkydGPVnRDPVEdxqp/A44Q7ufC0XV5XMWc1Fs9vGgY1Nz42pmwG11eu/wWh5XbDjSPnwO19t
tWjRQpI0ZswY/fOf/9/SKFSsUEhKioKKgRs3TgEBAgjsRs3TgEBAapXr54OHjyoN954
ORktWvXTTLZoaY2bdpo7dq6ne4nfHAAAsKbzPWjyUOMxTdMzDZN5gEzEpxmDuArb9mdzBb
Ty5El9++++A2iOo+PV0DA/6pMSEjQnDq6FbdpCMyQRx2kKCa314eLgaNmyoLVu2Ecaa
HCh80KUlnzpzR6dOnvDnYZWEhHcZ4nDTnHh4u06tEpdCqVav0888/2KGvaedkDLDvd0Ve0xcaMbf2z
tWqSpMOHD+vo0aOSpCuKuLINkeOHPGo8ecKCCi7+/8Vzpb3dNZ8Sir7rj1zTnuLLtijth3Omzqdhe
H82m82jpwi7ythkurw3ZXXO5r7cb2mU9ccxx/F+1oYNGzrRt2jTt3r1bhmHIbrcrLi5Ojz32mO
Lj403X5/HVTNu2be6a6a6ha9ay6ZOLzHsJDg6WJOxm5io7O1uSKmTnjLdaDkmS
32xQRUcGrOkrCO1nz5RX9ZQ79dxXG+OtY1/3eDoddAQE08+X+e4mrw26+vFdlbX7arzd1/xsk
/HnMlefjdfdOmTRo0aJBuuukm9e3bV2+99ZYGDBigL774Qv369qWdPk+vWdPwDWKM+vWdPIk
SPVtGlTTZs2TZzIEhKivLw8+1t+1yc3MLSWFhYa7ho7y8PLehpNzcXOKrjQh
wOu8LDQ5WZma2CAvO3WS5v6C9zykN/lVYAKetjXfLf51G434ICp/LzC0yXL/jvLeALnObLe1X
W8NN+vSn738/VH8ccx7v0yiuvqEuLpo6dap27qN99nU0HD9ejjjgYMGacGacMGVqwYIGp
fZoOM3//+981+9ceJEderUSRs3btTIkSMVEBCgl19+WevWrfPp4KhR+2xG7bZ4Qq7Lo2Hq5q24wi8
vPL/kt6xofnlKwv0lzn0V8n4qo/+89Xkzv+UPLSiosHYhwjHRMH+5sKuo6P7ZEs1IYFHCKdAy05Wr0P4ZMs
x/++23GjBgQLHrevxfu8vSHHrevfuVfPPvus7r7X300UdpinckynUR/xFRs8aLjAeD
v3LhRderUUdWqVRUbG6uKFSvq66+//dq3PzMzU7U7t27TQ8nAAQAAawoMDhRh
Zv/+/Zo0kaZI6leqkqQYMG6cSJEzuffz5YWtGxHEr3QmmQHfMeVedpqmWfj49DuPDqvhTjccuWL1P
NAgSWfnyiQlJWnq1Kn6WNNPPtXfvXj3yyCOq3yqCOqXr26OnrXhaAD4i91uuU0CA3fX3X1iryQ1at
RI8+bNc0eGMjgOFlr08eAzZsxQQ4wdTNdZ4as0ai+//PmzY//HPPK8FKSUnhxbkJ+fkNnhknHhAAJ/xnkAJ/
cuZo4caISEVFRLJUaNGRxkzVHQFKTEFx0bXdsGHdlJ+frfOSNkXmCunnkAJAj7gzXFKUZhqKHzOn5ZzZAQS
Cg9xTUI2y+k0PLpi7XIxbNgw9e3eZV8OHD9fQUnmUmJion376xkOpKUcyRv1RJbjJM6NnfVMCIdmFvW2x
O4LiYuL0+LFi8+73uhwcvxbxHuE
PmzbVgwQLt2rVLISEhiovZhpj1TIRV1ZjVqnOi3wqL8q0UHr16u8KjAhOvrzAdSSDEAmQPYTXzRx
HKZTnEZhzxQFKS4uTpIMAhSUoQzAEC55OlzaIiyu89Yi5VzEQCAACWRpgBAACWxkmAChF
drvNo8mdXLILeI4wAwClxG63Tw736JLdwqEUp4xpCZ6dp6Yp5Whbf2rMaMuPbdEcMIq9MzaNRvNZoFMI8wAwClz
JOrZLhCBvAcY6EAAMDSCDMAADSCDMAAMDSCDMAAMDSCDMAAMDSCDMAAMDSCDMAAMDSCDMAAMDSuM8MAA
A+ZrfbFBBgfjzB6TTkkqZQqlgxxKPyBU6nMtCDR/QJgBAMBHQoIDlHPoI
DZLfbtHDNXh07YqmrmgxIMPH07cZU2WqRYerdpZmhsjsiHb9xnV6G+kAqTn1o3BswTkKQAuQvw9F
AFAMhP833qelAHgPcIMAJzDZjs7uKbIGwr5TZ3tFQKGWZyXLL8z8NBscsxPhYmP3tDqnFYkAKmD8xD9p4
YDiEGYAoBipaeavNDFCeOuKCLdBL72U5jZ6nNmYCNA6dSozR4Pht
Bc6kGIMAMAgAV4c/fg2leG6/a216hKlTCP9n2pPOaBMAMAgIV4Op/rcn6MA
c4QkAAFgaYQYAAFgaYQYAAFgaYQYAAFgaYQYAAFgaYQYAAFgaYQYAAFgaYQYAAFgaYQYAAFgaYQYAAFga
YQYAAFgaYQYAAFgaYQYAAFgaYQYAAFgaYQYAAFgaT822ALvdJrvd1d5lFZp9O4ZB/ZDgBAaSDMXOLsdpuqR
ITJYfdsEK3A6VGepZfAo3D4VmbCWAAADMIM5c4uw90h92ud1fvUWpalmy1yTLdoqtmy1SLD1LtLtLA9ntNp

+GA5vt7P7Cw0M9Ku/PAAYAsB7CjEWkpmXp0PHTPt2np6e3AgLsstttWrhmr46dOGOqrL8CWCF
O6QGA9RBmygFPTvfYbDZVCg/x+PSW5J8A5o3SOKV3KjNHhmEu0Hh6Og4X5mkw9TTMAvAfwsxl
rFJYoFeneyR5dHortnakurSqI1nsd4I3p/RqXxmu29teoypVwjzat9NpKCCAOUalxdtgCsBa/
BJmnE6nZs2apSVLligzM1PXXXednn76aV199dX+aM5lKyQ4wOPTPfVrR6prqzo6np5tenSlWq
Rnv9DP5Y/RisJ9ejKiFBUR6nFf16lRWT3a1FOlSswxKi3eBFOrhnGgPPNLmJk9e7YWLVqkyZM
nKzo6WlOmTNHAgQO1YsUKBQUF+aNJlzVPfzn7Q2mMJjmdht9OFXjS19Uiw7yeYxQY6FBBgdNU
2dJwqY8Kefp5ALAWn4eZvLw8zZs3T8nJyWrXrp0kafr06Wx7Zs3T8nJyWrXrp0kafr06WrTpo3Wrl2r7t27+7pJJebNaMGl/
kP/UuHNaJL0vxElb0ajbDY/BaF08794vQ1/3gY/RoUAXAp8Hmb27t2rM2fOKCEhwbUsPDxcR
s21JYtW8o0zHh+dY7D69ECJoea4+nk4cIRJSuNRnmjNE4lehoc/X3lGQAUshlmf7t66ZNPPtH
QoUO1Y8cOhYSEuJYPHz5cOTk5mjNnjuk6DaNkox52u82rv7qzc35XgQfd5bDbFRLk8Grfp7Py
VGDyF0ZggF1hIYHlpqw/913eykqSw25TxbAgOZ1OXeywKK1QXtJj3WaT7Ha7pfqU7741yvpz3
/4qa+ZYl/zzR7jPR2ays7MlqcjcmODgYJ08dkKjOm02mxyOsj81EBoSWOb7OJ+KYZ7PJSpvZf
257/JWVjobGnzF7LFuxT7lu2+Nsv7ct7/K+vJYN8vnLSscjcnLy3Nbnpubq9BQ6w3zAwAA//J
5mLniiiskSampqW7LU1NTVb16dV83BwAAWJzPw0xsbKwqVqyor7/+2rUsMzNTu3fv1v1vXXX+/r
5gAAAIvz+ZyZoKAgJSUlaerUqYqMjFSNGjU0ZcoUVa9eXZ06dfJ1cwAAgMX55aZ5w4YNU35+v
saOHaucnBzFx8crJSWFG+YBAADTfH5pNgAAQGm6dK+zAS4r5hDK7lDp2nAt6pq2Q4Xa89NzauA6k
TCDAAAsDTCDAAAsDTCDAAAsDTCDAAAsDTCDAAAsDTCDAAAsDTCDAAAsDTCDAAAsDTCDAAAsDTCDAAAsDTCDAAAsDT
CDAAAsDTCDAAAsDTCDAAAsDTCDAAAsDTCDAAAsDTCDAAAsDTCDAAAsDTCDAAAsLQAfzegNBQUOJWWdqbM
6rfbbYqMrKC0tDNyOo0y28/lgv4ypzz0V1RUpVKpp6yPdal8fB6lif4ypzz0V2kd72YwMlMCd
rtNNptNdrvN302xBPrLHPrr0sLnYQ79ZQ79VTYIMwAAwNIIMwAAwNIIMwAAwNIIMwAAwNIIMw
AAwNIIMwAAwNIIMwAAwNIIMwAAwNIuizsAAwC8Y7d7diM3h4O/ieF/hBkAKOfsdpuqRITJYfc
smDidhmw27mgL/yHMAEA5Z7fb5LDb9e7qPUpNyeFXqHMvt+eFXhBkAgCQpNS1Lh46f
NlWGERlcCjjZCQAALI0wAwAALI0wAwAALI0wAwAALI0wAwAALI0wAwAALI0wAwAALI0wAwAAL
I0wAwAALI0wAwAALI0wAwAALI1nMwHAZcJut3n0wEeHw/2XQu4VZwtYZxOlO/IWh+J9ZQJwZ
ADAJcBu92mKhFhcth9N0Qc7R2D+TaT7nR2nOh20ZPmAWrPJ7HGTtzRXmlRqhVU9LM0/OubkTW
KMjKzEQ0+zBNDbXzhgHhpTT/pu5WTqVsoW2DXCJycBmCpVYdqd1O4Wyf2vH8z6k2d6yNLvz1uvU7TG0wZHz02Ml0
bapMVESo9/tNN79foLQwARgAAFgaYQYAAFgaYQYAAFgaYQYAAFgaYQYAAFgaYQYAAFgaYQYAAFgaYQYAA
FgaYQYAAFgaN80DgEuEp89Wkkrn+UqAVRFmAKCMlfTZSoZkwc0gr1AmAGAMlbSZysZksdKCrd1
AlxJNnK0ml83wlwKoYzwQAAJZGmAEAAJZGmAEAAJZGmAEAAJbmUZnxXnpiZ
Nmqh79+5atWqVa92ePXuUlJ9kSkZs2aqX379kpVp00ZNmzbVAw88oIMHD3r3
LgAAQLllOsx8+OGHevHhFfdoxV6+OGHevLJJ3XNPatWtWpjy6ytatWv4++ftuyu+Ly9a2Ty9
0efO
UnJysdu3aSnT5+uNwaa03aSZKmT5+uNm3aaO3aterevXuvXvpv0MAAHBZKMAHBZKMAHBZKMAHBZKM
GbMmKGlS5e6wm3ySse6ys+ePVuvsrPd6VmpyrKG2pX6Yqi/K0fY6Dt
t1//33+S5Ieeughbd+b++XZs3W9bmZSkcPGKSkOPVVaPK2VRWwWVPdDuGbs3bb9bmzZsVFBSkSkcPGKSkOPVV
UnJysdu3aSZKmT5+uNm3aaO3aterevXvpv0MAAHBZKMAHBZMzUy89NPP+nQaOaOH2/KULjR
W7duVx8vAIC/peREhIStD//fp04cUL69euuTs3blRy9uyaWbZXV4uMDh36F8KR0tdxXO+qqpbzvXp
jMHDhyQJGVlZbTXMzCDmUPRT0QZgjm8FFkzfp+3eEVmeKOFOTQOsxvLGYkpapjcdpFFbp7Ooi4/YG
Q3DHWe6OIhIbh3HnRTKKjq1vH64z2HkHqx
FkmyNHjnj0BgAAAGXWXDJTH4ZZmiIqaamSMUHFQkKeR21+KpEk0gcm8Cl/hdLgICxLjtCfz5J+KW3
4OFiSlJubHvTMS/Cz/Z72xLBmxC6jzYwwgjxn0jqaJ1s+S1gmxVrvCK3X+EAsLKnwm4WUtLKT0jvtFL2o2b0Kk
...

nZ2tsLAwSdLp06cVGxtb7HaxsbHKyMgwXT9hBgAAlKmaNWtq27ZtkqSEhAR98sknxW63YsUKt
WzZ0nT9zJkBAABlqlevXpo8ebIyMzPVuHFjzZ49W8ePH1fnzp1VtWpV/fbbb1qxYoU2b96sZ5
55xnT9hBkAgF85HJ6dJHA6DTmdRim3BmWhd+/eysjI0Ntvv62TJ09KktavX6/169cX2Xb06NG
64447TNVPmAEA+EWlsEA5nYbCw0M9Kl/gdCojPYtAYxFDhgzRkCFDlJmZqaysrCKXZXuDMAMA
8IuQ4ADZ7TYtXLNXx066cMVW2WmSYendpILvdRpixmPDwcIWHh0uSzpw5o9OnT6ty5coKCQnxu
E7CDADAr1LTsnTo+Gl/NwM+tGHDBk2bNk27d++WYRiy2+2Ki4vTY489zvpvvAADwmU
2bNmnQoEGKiopS3759ZbPZNGDAAAGVnZ6tfv37aunWr6ToJMwAAwGdeeUVdenSRa+99ppuu+0
2GYah4cOH68MPP1Tr1Y8YM03USZgAAgM98++236tGjR7HJUwAAwGdeeeUVdenSRa+99ppuu+0
UAUFBcWuy8jIUHBwsOk6CTMAAMBnGjVqHnz5ik3N9e1zDAMHTx4UDNmzFCHDh1M10mYAQAP
jNs2DDt3LlTw4cPlyTZbDYlJiaqW7duqlChgkaNGW6Ti7NBgAAPtO8eXMtWLBAu3btUkhIiO
Li4hQVFaVevXrprvuUlBQkOk6CTMAUIrsdpvdpvdpvpcp7e0h+wori4OMXFxUmSFi9e7HV9hBk
AKCV2u01VIsLksBNMgPP54IMPmxu2mxx2u95dvUepaVmAHamurayvHamurerI
ZjM/qgNYyZNPPlnscsMwZLPZZLfbCTMA4G+e3J4/KsKzhy0CVvPpp58WWZadna2tW7dzq0dO3Zo1qxZe
uml10zXSZgBAAA+c+WVVxa7vF69esrKytKkSZO0aNEiU3VyYhcAAFwSrr32Wu3u3evdt0OcIMAA
Dwu7y8PL3//vuqWrWWq6bKcZgIAAD7TsWNHGYbhtswwDKWnpysxt0tTRvebYA+w3N1cjR440XSdhgAA+MwNN9xQZAEUaqo
QJMzY7XaFhoaqQ4cEat26k6tek0Emp0UPkh2aVOp1EmYAADIP
q127dh7da4kwAAAfCY3N1cPFfSQNmzYIIfCGVkZCGhnGYdoqSJiwCGVkZGju3LlrbqpOrmYCA
AA+M2XKFO3uWcuVM2Z87Uzp16Fe9uWXX2rnzp1uWXdnzp1MkpsBDaLC
cSmGBcjoNhYd79kyrAqdTGelZBBo/6NatmxYsWKBWrVopmDDQtTw7O1tvvvPGG7r77btN1EmYA
AJYTEhwgu92mhwVu92mhWv26tiJM6bKVosMU+8uDWS32wgzfhAWFqatW7eqc+fOat5WDaAdMN
y++0KlTp3TTVVfpiSeekHT2ZnrPffcB97f95fzy+ldtnjVl+CRhSTgPd2iSBOVyUbDBv/M1AA
X+eHO98yHMAAAAn/n0009Lv7U07CDAAA8Dmn06mfmZZqcdqmg9BwydV16zurMPbzZZQdc4
LL72kEydOuJZv3p5pdamrHr06xX6B1f43Zg6Pu4zAwAAfGbj16bDUk5E9E0n6eUGVd4DWbZmb85
kpOTtW7dOtNEmYAAIDPpZJ07V7169dJjg0cvd8vrq7gGbRmtUJV38oDaN
ACf+f7773XzzSmTcXu65Hjx76/vvvTDdJmAEAAD4kaw2hdPFrvx0KFDph8ZU3/6mzZ
tr2bJlrmV79xqLr8rUkSSmjVrpvbt2yslJcWtjNp1MyzM9WmTRs1bdUgaA4ePOhpEwAAgMV
cd911evXVVX3X8+HHXHXXmFwtGnTJk2duaPbdk2u8YXAdHYeb333/XqfVXVnufqxAGQMV5VqWgtkBWbDKh0Nz
2lq6dKmGDGh2qGbdMoaOnSpa5tZs+erUFmnChAlavxHixbdDbBg4cqXy8PE6d
uyYRo0aJUmy2WxKSEH37591ahRIz366KOm6KmOm6/To0uyX35FSpUcFv23nvvKSgsOPGjVNA
Dq1aungwcPP6o033lDPnj2Vl5enefmKTk5We3atZKtZ8XW3atZMkTZ8+XW3atNHtatWvVxt3
T5oCAAAs5NprO2ZcYu0a9cuhYeHq2vXroqKilKrVq1c+cAs02Fmy5YtWrx4x4sZYvX6727du7dd/l
CPhflQkJCCZozZ45OnDihQ4sc6cyZM0pISHCtDw8cyZM0pISHCtDw8PV8OGDbVlyxbCDAA5USGNjVUo0YNSdK0a
dO8rs9UmMnMzNSYcqYwXXHFFFuFFW7rjh49apWVlm6kiSzVdQDLaWpvdpUDbVxqjVYGLdl1apVkyQ
6YbjgAALCeWbNmXXXSbhx9+FSdpsLMnHzMzK3K3Kzs2KxZs2pVq5a68yGr3O3L6YVZpdW5zdnS1K
x25xvVrMZAQFld2GWw2F3+z8ujP4yh/4ypyyPdcm7z6OwjM1mkM1XWtb1NpstH6W96rfZ+z
uS/3Wxpl/XHMcbyfnT97vgdIOhwOXXnllWUZUXZpYvX66tW7fqo48+KnZ9ks5eh
hUSEiJJysvLc/27LFc/27cJvQ0FBTjf4ju92miIgKF9/QS+Hh3rWzvKG/zKG/Ls5Xx7rk3efhcNgVEO
AwV8Zud/3fbFlvy3tV1uan/XpT9r9Bwp/HXHk+3nfv3l1kWVpamvkWvbt26eq39xi+k6Sxx
mli5dqhMnTrjNk5Gkp59f+WikpKbryiuVmprqtq7wYnk7be4mWqvrgwdZTq+a
lJmZdfENPeRw2BUEDjLbD+XC/rLnPLQX6UQMr6WVmGrJ6Gaqtyyf6rbIHT6
fq/2bLelveqrOGn/XpT9r+fqz+OOY734kVGRiohRiEBEj9NBDD5kqX+S
gtu/XWWzVs2DB169ZNqSCgI5HGdT8saNG1WnTh1VpVpVUsWKFX1X111+7wkx
mZqZ2796tpKQU4uTn5+2X8pzv6Aujy/fGWB/jKH/ioZX/WRN5+HYRjnHUa/UJmz/5DpstGW
92rf52zuy/2WRll/HnMc78VLT09X5X5ezVqVVo0YN9N9Rd5ZNMoAC6AGHj9u6otzJNbJG1Ia
MAAffPN5o/f77Gjx8x8v6v6excmaSkJE2dOVq07ChYc6v4xcAs6cG6mGw4AAC4v3U1q
vWrU2X8+g+M8WpWrWq5s6dq4kTJyFUFRUVEaNaqUEhMTZZe6U3NMWqUpVVtq4bYa
UlJQik4IBAED5VKYjM8X57rvv3F3x7e3ZEVh3x+fqacq+vGBkAAGCkVJqcEtDADApfxeGAAC4L
hBkAAGBphBkAAOAXx48xXs1xbc1hDrSkxmBO8dqxxEdwj+I8IMAADwi9TUVM2O2kzGa/bqbiTe
cIMAACwNMIMAACwNMIMAACwNMIMAACwNMIMAACwNMIMAACwtFJ7nAcEsMDLEZhvU1+4zTcs+Mk
MAgHKlUigEdE5D4eGhHtdR4FR
MHgsLWi3YZe4ddsUgp7Edu87i4UhNllvggjJJaXtoSuQaXLiuMa/pzSqnV9jsC8/zAWKsl/FjyAAAGEpp
HyG63aeGavTp24ozp8tUiw9S7SwPfizPCUP2l8q5t/NXLVaW7XodV9R4BXSR3zmidaVV8zcsOjaI3ewkIPX/dirWBnkKBxIWDEIhb5HR0lG
7e9moBQwARgAAFgaYQAAFgaYQAAFgaYQAAFgaE4ABAIDPdOzYUYbnsjSNqWvdYZU8tuQ0gBA
AA+c8MN9o0zPz666Pf1c0r666Ml1wAAApjMpEilWQnYQAAPjMpEbilWQnYQAAAMsfqvAPvvsS4uQSqtq4KWMrdLwJUmVk1LmZ8rDKUbGGy8x6uNoj08Ta2GXf3Al8TH0

jQoUR1EmYAAIBfpKamavXq1Vq5cqV27Nih0NBQdejQQYMGDVLbtm0VFBRUonoIMwAAwGfS0tK
0evVqrVq1Stu3b1dwcLDat2+v/v37q127diUOMOcizAAAAJ9p27atJKlz586aPn262rVrp+Dg
YK/q5NJsAADgMx06dJDD4dC3336rXbt2af/+/V7XycgMAADwmZdfflmnT5/W2rVrtWLFCqWkp
Ojqq69W165d1a1bN9WtW9d0nYQZAADgUxUrVlRiYqISExOVnp6u1atXa8WKFZo9e7bq16+vrl
27qnv37qpRo0aJ6uM0EwAA8JuIiAj16tVL77zzjj799799PddttWrVq1Tp27Ki//OUvJaqDkRk
AAOAzDRo0uOhN82w2m2m7755psS1UmYAQAAPvPwww+bepxBSRBmAACAzzz00OlXidXgAA
Dzgc5qedelLmcvTBBx/om2++UYsWLdSjRw9J0t69e1W7dm2FhISYro8wAwCACZXCAuV0GgoPD
/WovNNpyGazlXKrrGPWrFl65ZVXVKlSJS1cuFC//fab4nNasGBBBia9iKkSYAQ
DAhJDgANntNi1cs1fHTpwxVTa6agX16hwru738hpmlS5eqb9++evzxxx5WSkqLXX39dffv21XP
PPadHHnlEM2bM0AsvvGCqTsIMAAAeSE3L0qHjp02VKc8jMoXS09ND0NdT8O+8847NXXqVP3666+q
WbOmBg8erHHjxpmuk5N3AADAZ6Kjo3Xs2DFJZ+8xwUqnz0vYrkWQM
n379po3b57On7t6iq5x48b6/vvvvJUnbtm1TeHi46To5zQQAAHzmmmuu0Y0On
r0qBYtWqTNmzfrn//8p267Tb7TdRJmAACAz/zf//t2fKleurMDAQO3cuVPS2bl4BEw4cUM+ePfX
oo4+arpMwAwAAfGbFihWqV69eqdZJmAGAYnBDNKBslHaQkkGxgzAODGZrN5dUMUNABfWsWNHU89m
+uyzzy66DWEGAM5ht9hs8viFa/dqR6tqqDvcSAS7ghhtu4EGTAOALntwQLSqCSQqC0RyUjN1uU0CA+
dOSTqchp7N0g4CvTZo0qdTrJMAAOAjhc91qljR/MMUJanA6VE5lbAHEpbB1A41hGPriiy+0BeQ
p1SpUrV9aNN96o1q1be1QfYAAB/x5rlO5SLzFFTt0eZycHA0cO0HFBbt25VwW
5I7vdrrlz5+qmm27S7NmzFRQUKZpOwgwAAD6Wmm27S7NmzFRQUKZpKpOwgwAAD6Wmm7+NOblYsaMGTpw4
bdMXX3yhsPWKoUaNMSa2/qsw
q6TMAMAAHzmxIkkT573TXtL169XXtKkdq3q1Klj6+w0TnlVHvv9nRzTvwSrbuqszXkE7ZRRRr5Rb9O
P/93//p7Zt26pFixbq1auq/Z88eSlqVmzZmfvr1SUlLcyjudTs2cOVNt2rRR06Z
N9cADD7ge+w0AAC5/3bt316xZs/m9lHCA1Ss8k8Wt/Zeg3d5YpHd1yNIbN2GN
06bp/fffVFV6NGjdS/f3/9+OVS7dm0tXbjDyovL8nA6cO1YwZM7R3r17t
GGCFi9eLJvNpoEDyovNpoEDyovL890wwEAgPUMGTbUtzTeMqZJVJaqX0CjvQ7yD6ckyUjKxHt
k6S3w108GDB7VhwLQVq0kCSNGTNsa8gGkrRTRDqmZogOGQ/c//SdFHX88mBNTJp2vaYZQT3Fvrgx
qjTfeUM+ePZWXl58+YdO1dq1Xk8rLcQoecAANYSFBSkl19+ucjyCQkJ8PKBaFPIZEcQDotwgv6xR
GQoICNBrr72mbWqKMYmRna7Z7NfSlwqIiJCr7/+uho3buxKaLNpTMpaSBh8bGo9clqk6ZwIsbmbtdp8c
f/LRwkJCdq/f79On/8NLq1Ss+P11010003afr06YqJiXFbx3J1M
eVL+rkijJXtIRcVL+NbfXMK66fr02wf9TsS0pudD8W9vSDFf3u2Bk71tyLfkqHAsPKxMbMkbu2mMmnQ8gH
eTcaDWWjQgAKKtb5KSkmBwaG1t1cgsVWxixQb4IlDj1d8V3TB
cvcMaZ9mPzTzLq8ku0uHzJdX3JLAhLQ4cqsYKx/iq2mQ8E5BcHgvzJ
eTxxZW9zYKCZoXpKIG9ePDadgDwxhO3r0dW/3OHzTJHXvYvVl0kVVEngW/Mgu8/aL
eLmL//CyuHB/+99dXpUBGyRmYhWRAoFtANiFRRjjDb1xyX9gA

K4SZEvD2WUPS2XuQnMrMkWGYCzTeBChYiz8+a0I2gMsBYaYEvHnWkCTVvjJct7e9RlWqMLHwc
udJIAkIcHgVlp1Ow+N7o3gass/dN2EIgL8RZkzwZlKqp2GIy4WtoTQuKffm++FJ2dII2dz1GM
ClwC9hxul0atasWVqyZIkyMzN13XXX6emnn9bVV1/tj+b4THm5V0x5VBp3TE5N9/z74el3y5s
RR+56DOBS4ZcwM3v2bC1atEiTJ09WdHS0pkyZooEDB2rFihUKCgryR5OAUmHF+5pwx2MAVufz
GYd5eXmaN2+ehg4dqnbt2ik2NlbTp0/XsWPHtHbtWl83BwAAWJzPw8zevXt15swZJSQkuJaFh
4erYOG2rJlS5nu2263KSDAbvo/HjwHAMCly2Z4ehmDhz755BMHhz755BMNHTpUO3bsUEhIiGv58OHDlZ
OTozlz5piu0zBKdkWF3W7zaiLt6aw8FXgwNyAwwK6wkECPylPWHCu224plJclht6liWJCcTqc
u9lOktC47L+mxbrNJdrvdUn3Kd98aZf25b3+VNXOsS/65zYTP58xkZ2dLUpG5McHBwTp58qTp+my2kv0xLq
ddpsNjkcZT96UjHMu/k83pSnrDX2Xd7KSmdDg6+YPdat2Kd8961R1p/79ldZXx7rZvm8ZYWjM
Xl5eW7Lc3NzFRrKlTsAAMAcn4eZK664qjw1NVXq3f3dXMAAIDF+TzMxMbGbqmLFiv
r6669dyzIzM7V7925ddtllvt4cAACwOJ/PmQkKCtKIAQP0/PPPKyoqSjVq1NCkSZMUExOjXk7k3YnfkRpKmTp2qyMhI1ahRQ1uPmMTd0
SurhhHgAAMM3nl2YDAACUpkv3OisAAAIAS
IMwAAABLI8wAAABLI8wAAABLI8wAAABLI8wAAABLI8xcxKFDh1S/fv0i/y1ZssTbukzJ49W
3369HFbtmfPHiUlJalZs2Zq3769UlJS/NS6S09x1gXbz7o3769UlJS/NS6S09x/fXEE08U+Z61bdvWTy0sfzjWS47j3RyO97
Lnl5vmWcl3332n4OBgrVu3TTjbb/57WW6lSJT+26tLy1ltvaebMmYqPj3ctS09PV79+/XTLLbd
o/Pjx+s9//qPx48qSpUq6Uq6tmzpx9b63/F9Zd09rs2ePBgJSUluzZY5HA5fN6/c4szje/c1gvGY53czje
fYMwcxHH79u1TnTp1XLbrPxP4Vz6tvb5r333dHnQnOnFzg1W1rv3sfBQBKAyFeIG4lbvFb
Z3NxgLvE6bmCn09m3zY/Oo0bY6w7Ng7Hi0ukHiUW0mgotTvqrH8BWMyC9AQ3/8oEbIj78rjBdSPZ8W5DK/Zs79LFH6cYSBzPB6PkNfkw2ZEGJ3W3gBM2n3sQRPqxjBNPB/Ns6cTc1DzBU6FqlUEsHbpYe+bQKkh9FQT88SfIvu6Ow8XK/4enJRk8Q4+lMD4Sl71ZtZcx5O4yoxdH4Y5V9v2HdqhbDVgYB4XVyeqDwQ6e8+Zo8/dDnyvxK/Ps0ptn7WFtUqlvRjLYkNtGOk7YujMGVNbpyqa/yEIk3r33Ff1Zhpe0Yn2LtxQlC5wNV9MFwBkPh3RqbHt4sDxiSAwFu/PS66K2+BppVyuuM4kvf8m28g7rjfr3eHuuJzuxDcMc/jLQLWs8r8P9UyuM/iHNiN5XQcIWwtzZJ3dcNl2YcmWDrCFqytzVu/5Bu6irfHyHptX/3Ti9OG2jDcEfF6W+hphtM3BYSkVHgAhP9GM6fu6ALtyRW2Mmzr2hzqWDv0Bv+yr7oZrfxZoWGvZ4FZrH7xTtu5WcOvuQh/mWMzxkBvzsmhDzX/TQKvxZnwzGbLdHMEcRJAw8v3pgJfuxWbFJL0xuwVr/hd2n+wr2f+1zlyFMAsqgPZEA/MAPxxPD1oqaAKU8qZGpUPtbCXh1MuXPvDrwlj/8gUa5iRqwxEQfF2VvsFfBpnLM2Ah/bvO/vAHyYnwoHUYPWshHa81HUYqYgX+fGo3k3vsKG8W/69r6xJUX0FUF6/MIOt3u/pXEkfcnvxtkc/j2J2UfnLF0Op3HBBsA3SHIrafRbi5EsqvrPWY5AV/lNAsfhqcgUbS5T8fm8rPFDF36Jt/TM2NeFQszw5+Rxw7wDjOBl6xK0iO/lgfZaPSgL3vqxzepbqF/RhQFXdy0GbCmHHTC8Dq8TiJTR/wSj2nr2FEsdj69cYqW8NX9Uy6OWhh2hrH+CMZvBBeHGGtDgAm8ZEfhcnEtUh3MeuBKcJj9xSr0A9PnLdOCyIHPnXrWFx9wYO1/CShPymHM9FupWMSUCH9aHFNBhOFCkkj1AuR9AjYK1AX+EGzGf5BTa6YHdwdyaZV4qGbeCUdK9m3u3X3qvvBsbE7eyjhmnPTT7QAMOo+y29J+HjJYaS5EkHzkOPCw3X0Hq8PN7zGM2Hw1Nq6TdGUvTjE1cUvLZvEEkxqzhsUHc2NpGhHfT85mgbnZ3yMpr30q6ibcO6GxqpmBDxqTTZnV+VjpPb1G8zpVxYJUgTbxxPoyhiUmP1wwOfKl6jLBMbMmeHGMVZ32GNGGWfYx5FDG9RJuqSvZZ6Pd2nmG0wdvx2p+fRdIN3k8bSaSbf3bV8f+WnMHjC4jyNm3ixWxP5mDDSPN19fS+Le9MzqhBxxC4hQGhNWnxPN7PDQwkdgYwXpUmAMXFrP2jVsncH1d8sPrXvIdHqtDuP5JKQ/5/aDQJk8SvOnQBMwSnqzyqPFj3h8ByoTPpV0r5/AUEn5GGtMT7lpVj1AXrhMf1kM9PeHkgTh7yVI3mzKxdPW6OLgcV2k3ZlfxMQ2HGsxCKMHhQXkTNDmYMwNvuHxovEM83XgQB3Fx3UWhP4M7eN6Q6b7XG9pZmKdv6YmGk+YoQA+H0MD1oQ5FbX9lwTjD1xMWTOgHKvB0y7mLZvoY9fZMAvgU9+AHdyq3m+E8U4rq3d8qYXAxiUuQpBwANRuJWcnTyT/w85P3FbqxZ9f3EwIS2AfxEzHqBIdyp1dSbdRnKRIfY2Lx7d/IhYtBc4HtFt9Vw9iF3v8y/uezS1LzXVSg9kQKf/WHxAKVyKQ1/H+gRk3u7rcXDPhfvI8dbwdqxHKqc2+NAlm8HRAAAVDP3xEy4bvLivG28kCsSHXf9Uv8BdbkWv1U+7Tl4QJE6lr4J9vDj7IKOD+VOgTLqT0Xrg2HKiJgE8XU9nXSHGj+4jNbmVRiTHVDjcOXWfC19AVlhkehfiV37d9oXwPN0IAELyR1e7gBpyjG8FlvoK3bZmDRUoOaQO8p62wFCbOr+8yThbB2SKUadN8+Kc+Cbu9a8zAfQz9aKK1Dxi8JpiEVDk9E2m6/S8v8q3m3IpA8pPQoOsY1JmsDAQ76RtqOP9Dv1vWnePbwNAoKDy0K2QiB1rKxVlFQKz+qO6HHBcUgRZyQ1ZkMMcVNv1G1Aqe0NUBuXJWTJDzNtTJbl3rtlT3a0W+rZEdH3x9MfTG5d/Uj/CP2vbTaMLNpdfAJlRcNExCcGxO8OUm2HCnEH4bRbTqYRuU2K/G3xE+u1XnQC1y1BRi2Cp1Gq+2l7zAvG9wQeIgPtXe+nYbP1YsQXHm+2dO7ZyjbNO8dWVA0s+Ek8W9pHBfkEp8OfrMCkvTKLTiXGxH8d+FrTYRb9TlG8Q+cv5PpX0z2Q4qWwCvRkzwi9v48FRXUB8pEEzRiyfQXGAUd3e8ewEFR0y5ppVkw67HD6oSXyvVk6tdm+/IoB4BMSVF5sLySjfGE5lWhEXZFiTArpRbVjHSEAM1dqwdnBYHZL8ovqPEmMTwtLj8NZcpe5HfsfvkCSxzm45g8TLUYsCQ8VWCnEDyF81tAT+Bnzm3ISpMWYO87wJCEnrVYdfqrTR4Uns8pBx4ZL8Ihz3tx1Aeu2TkqBh48IxU6ENIDcAGdT3KzFTS3rbAC8x3l/X+0TNg5xqp2+z7MyaUlX2aB3O3WeWWBqFmDyVhSWKaLNcNsQhm8lIZEn6lxEeK+TRz5lNkHsLqDkjJ3/Okfq5jJ0j/1/YVi7UuA6JIH6DuBAV1+qZjRt5EUWqwBEHS+VqD4GBjKl3tGrW3M8u3GEbLQFP4WoZ4cq8LO4FxQG2gIAuYCJhkyVPI3Sp4GQNkHdmFJLvFqJ2tV2ROBKLEBCMQMEhpvMRhiImVjW3nzUpOjZvq6jz2K4txDj6jVv4Ld3N9fPOoScs8eGPpR0ux87MjoWR6Fek4Ob9S3yvHc+R/4vrDyffgY5U3K6AA1J/yPdRcxDzqO1PJpUKtLxubbRwkbq9wuvr9N78++Gd6xMabZ1/dh7flLa1TzLBHgUxqM8jPWDvy6EB8oHlLrsOMEd5AsmeOBkDfHVQmM/Ax7q4GBuX3bRefLiR/vGPMj4XfZz6W/SS1r49lMlIYvjawOhnrL6ka8Cw3XezX8sVSAw9Yg5r6Kd81wWaMMfKGmjaszyO+Gur1QJ99NTnRtzOFZp+7u38x0=

"text/plain": [
"<Figure size 600x600 with 4 Axes>"

```
      ]
     },
     "metadata": {},
     "output_type": "display_data"
    }
   ],
   "source": [
    "df_init_2_time_points['trt_grp'] = 
pd.Categorical(df_init_2_time_points['trt_grp'], \n",
    "                         categories=['<=30 days apart',  '>=90 days 
apart'],\n",
    "                         ordered=True)\n",
    "df_init_2_time_points['age_years'] = 
round(df_init_2_time_points['age_at']/365.25)\n",
    "df_init_2_time_points['gender'] = 
pd.Categorical(df_init_2_time_points['gender'], \n",
    "                         categories=['Female',  'Male'],\n",
    "                         ordered=False)\n",
    "sns.set_theme(style=\"darkgrid\")\n",
    "\n",
    "g = sns.displot(\n",
    "    df_init_2_time_points, x=\"age_years\", col=\"trt_grp\", 
row=\"gender\",\n",
    "    binwidth=1, height=3, facet_kws=dict(margin_titles=True),\n",
    ")\n",
    "custom_colnames = [\"CTRL\", \"BB\"]\n",
    "for i, ax in enumerate(g.axes[0]):\n",
    "    ax.set_title(custom_colnames[i], fontsize=14)  # Adjust the 
fontsize\n",
    "\n",
    "g.set(ylabel='')\n",
    "g.set(xlabel='age')\n",
    "g.fig.suptitle(f'In-center program', y=1.06)"
   ]
  },
  {
   "cell_type": "code",
   "execution_count": 25,
   "metadata": {
    "metadata": {}
   },
   "outputs": [
    {
     "data": {
      "text/plain": [
       "TtestResult(statistic=4.024969228864507, 
pvalue=6.724888995398134e-05, df=433.498231695994)"
      ]
     },
     "execution_count": 25,
     "metadata": {},
     "output_type": "execute_result"
    }
   ],
```

```
   "source": [
    "demo_init =
df_init_2_time_points[df_init_2_time_points['time_point']==0]\n",
    "demo_ttest(demo_init, 'age_at', '<=30 days apart', '>=90 days
apart')"
   ]
  },
  {
   "cell_type": "code",
   "execution_count": 26,
   "metadata": {
    "metadata": {}
   },
   "outputs": [
    {
     "name": "stdout",
     "output_type": "stream",
     "text": [
      "count    8326.000000\n",
      "mean        9.996774\n",
      "std         3.094034\n",
      "min         4.010951\n",
      "25%         7.600274\n",
      "50%         9.629021\n",
      "75%        12.119781\n",
      "max        17.987680\n",
      "Name: age_at, dtype: float64 \n",
      " count    746.000000\n",
      "mean      10.436555\n",
      "std        3.243900\n",
      "min        4.087611\n",
      "25%        7.911020\n",
      "50%       10.087611\n",
      "75%       12.800821\n",
      "max       17.911020\n",
      "Name: age_at, dtype: float64\n",
      "count    620.000000\n",
      "mean      10.327182\n",
      "std        3.147210\n",
      "min        4.030116\n",
      "25%        8.035592\n",
      "50%        9.980835\n",
      "75%       12.496235\n",
      "max       17.549624\n",
      "Name: age_at, dtype: float64 \n",
      " count    386.000000\n",
      "mean       9.991552\n",
      "std        3.120711\n",
      "min        4.191650\n",
      "25%        7.563313\n",
      "50%        9.824778\n",
      "75%       12.566735\n",
      "max       17.911020\n",
      "Name: age_at, dtype: float64\n"
```

```
     ]
    }
   ],
   "source": [
    "age_ctrl =
df_init_2_time_points.loc[df_init_2_time_points['trt_grp']=='<=30 days
apart', 'age_at']/365.25\n",
    "age_bb =
df_init_2_time_points.loc[df_init_2_time_points['trt_grp']=='>=90 days
apart', 'age_at']/365.25\n",
    "print(age_bb.describe(), \n",
    "      '\\n',\n",
    "    age_ctrl.describe()\n",
    ")\n",
    "\n",
    "age_ctrl =
df_virtual_2_time_points.loc[df_virtual_2_time_points['trt_grp']=='<=30
days apart', 'age_at']/365.25\n",
    "age_bb =
df_virtual_2_time_points.loc[df_virtual_2_time_points['trt_grp']=='>=90
days apart', 'age_at']/365.25\n",
    "print(age_bb.describe(), \n",
    "      '\\n',\n",
    "    age_ctrl.describe()\n",
    ")"
   ]
  },
  {
   "cell_type": "code",
   "execution_count": 27,
   "metadata": {
    "metadata": {}
   },
   "outputs": [
    {
     "data": {
      "text/plain": [
       "trt_grp\n",
       "Initial    4166\n",
       "Virtual     310\n",
       "Name: user_id, dtype: int64"
      ]
     },
     "execution_count": 27,
     "metadata": {},
     "output_type": "execute_result"
    }
   ],
   "source": [
    "# compare the virtual program to the in-center program (initial)\n",
    "# only keeping subjects who stayed for >= 90 days\n",
    "# note the difference between df_comb and df_comp\n",
    "\n",
    "cols2drop = ['birthdate', 'gender', 'Status', 'Hours', 'Center']\n",
```

```
    "df_1 = df_init_2_time_points[df_init_2_time_points['trt_grp'] ==
'>=90 days apart'].drop(columns=cols2drop)\n",
    "df_1['enroll type'] = 'Initial'\n",
    "df_2 = df_virtual_2_time_points[df_virtual_2_time_points['trt_grp']
== '>=90 days apart'].drop(columns=cols2drop)\n",
    "df_2['enroll type'] = 'Virtual'\n",
    "df_2programs = pd.concat([df_1,
df_2]).drop(columns='trt_grp').rename(columns={'enroll
type':'trt_grp'})\n",
    "df_2programs.groupby('trt_grp')['user_id'].nunique()"
   ]
  },
  {
   "cell_type": "code",
   "execution_count": 29,
   "metadata": {
    "metadata": {}
   },
   "outputs": [
    {
     "data": {
      "text/html": [
       "<div>\n",
       "<style scoped>\n",
       "    .dataframe tbody tr th:only-of-type {\n",
       "        vertical-align: middle;\n",
       "    }\n",
       "\n",
       "    .dataframe tbody tr th {\n",
       "        vertical-align: top;\n",
       "    }\n",
       "\n",
       "    .dataframe thead th {\n",
       "        text-align: right;\n",
       "    }\n",
       "</style>\n",
       "<table border=\"1\" class=\"dataframe\">\n",
       "  <thead>\n",
       "    <tr style=\"text-align: right;\">\n",
       "      <th></th>\n",
       "      <th>user_id</th>\n",
       "      <th>trt_grp</th>\n",
       "      <th>test_name</th>\n",
       "      <th>pre_score</th>\n",
       "      <th>post_score</th>\n",
       "    </tr>\n",
       "  </thead>\n",
       "  <tbody>\n",
       "    <tr>\n",
       "      <th>2462</th>\n",
       "      <td>708978</td>\n",
       "      <td>&gt;=90 days apart</td>\n",
       "      <td>ML_max_score</td>\n",
       "      <td>NaN</td>\n",
```

```
"      <td>NaN</td>\n",
"    </tr>\n",
"    <tr>\n",
"      <th>2069</th>\n",
"      <td>614364</td>\n",
"      <td>&lt;=30 days apart</td>\n",
"      <td>TS_max_score</td>\n",
"      <td>5.0</td>\n",
"      <td>7.0</td>\n",
"    </tr>\n",
"    <tr>\n",
"      <th>2108</th>\n",
"      <td>619539</td>\n",
"      <td>&lt;=30 days apart</td>\n",
"      <td>ML_max_score</td>\n",
"      <td>6.0</td>\n",
"      <td>8.0</td>\n",
"    </tr>\n",
"    <tr>\n",
"      <th>2646</th>\n",
"      <td>753667</td>\n",
"      <td>&lt;=30 days apart</td>\n",
"      <td>DT_final_score</td>\n",
"      <td>NaN</td>\n",
"      <td>NaN</td>\n",
"    </tr>\n",
"    <tr>\n",
"      <th>2686</th>\n",
"      <td>764189</td>\n",
"      <td>&lt;=30 days apart</td>\n",
"      <td>SS_max_score</td>\n",
"      <td>0.0</td>\n",
"      <td>2.0</td>\n",
"    </tr>\n",
"    <tr>\n",
"      <th>2977</th>\n",
"      <td>846125</td>\n",
"      <td>&gt;=90 days apart</td>\n",
"      <td>FM_final_score</td>\n",
"      <td>81.0</td>\n",
"      <td>64.0</td>\n",
"    </tr>\n",
"    <tr>\n",
"      <th>280</th>\n",
"      <td>261518</td>\n",
"      <td>&gt;=90 days apart</td>\n",
"      <td>SS_max_score</td>\n",
"      <td>3.0</td>\n",
"      <td>4.0</td>\n",
"    </tr>\n",
"    <tr>\n",
"      <th>2749</th>\n",
"      <td>773417</td>\n",
"      <td>&gt;=90 days apart</td>\n",
```

```
"      <td>FM_final_score</td>\n",
"      <td>132.0</td>\n",
"      <td>132.0</td>\n",
"    </tr>\n",
"    <tr>\n",
"      <th>1414</th>\n",
"      <td>491731</td>\n",
"      <td>&gt;=90 days apart</td>\n",
"      <td>SS_max_score</td>\n",
"      <td>6.0</td>\n",
"      <td>6.0</td>\n",
"    </tr>\n",
"    <tr>\n",
"      <th>2526</th>\n",
"      <td>720536</td>\n",
"      <td>&lt;=30 days apart</td>\n",
"      <td>DT_final_score</td>\n",
"      <td>0.0</td>\n",
"      <td>10.0</td>\n",
"    </tr>\n",
"  </tbody>\n",
"</table>\n",
"</div>"
],
"text/plain": [
"     user_id          trt_grp       test_name  pre_score
post_score\n",
"2462  708978  >=90 days apart    ML_max_score        NaN
NaN\n",
"2069  614364  <=30 days apart    TS_max_score        5.0
7.0\n",
"2108  619539  <=30 days apart    ML_max_score        6.0
8.0\n",
"2646  753667  <=30 days apart  DT_final_score        NaN
NaN\n",
"2686  764189  <=30 days apart    SS_max_score        0.0
2.0\n",
"2977  846125  >=90 days apart  FM_final_score       81.0
64.0\n",
"280   261518  >=90 days apart    SS_max_score        3.0
4.0\n",
"2749  773417  >=90 days apart  FM_final_score      132.0
132.0\n",
"1414  491731  >=90 days apart    SS_max_score        6.0
6.0\n",
"2526  720536  <=30 days apart  DT_final_score        0.0
10.0"
]
},
"execution_count": 29,
"metadata": {},
"output_type": "execute_result"
}
],
```

```
   "source": [
    "df_rmANOVA_virtual = 
df_virtual_2_time_points[['user_id','DT_final_score', 'ML_max_score', 
'RT_final_score', 'FM_final_score', 'TS_max_score', 
'SS_max_score','time_point', 'trt_grp']].melt(id_vars 
=['user_id','time_point', 'trt_grp'],var_name='test_name', 
value_name='score')\n",
    "df_rmANOVA_virtual = 
df_rmANOVA_virtual.pivot(index=['user_id','trt_grp', 
'test_name'],columns=['time_point'], values=['score']).reset_index()\n",
    "df_rmANOVA_virtual.columns = ['user_id', 
'trt_grp','test_name','pre_score', 'post_score']\n",
    "df_rmANOVA_virtual.to_csv('/Users/rye/Documents/Documents - 
Mac/BrainBalance/data/df_rmANOVA_virtual.csv', index=False) \n",
    "df_rmANOVA_virtual.sample(10)"
   ]
  },
  {
   "cell_type": "code",
   "execution_count": 31,
   "metadata": {},
   "outputs": [
    {
     "data": {
      "text/html": [
       "<div>\n",
       "<style scoped>\n",
       "    .dataframe tbody tr th:only-of-type {\n",
       "        vertical-align: middle;\n",
       "    }\n",
       "\n",
       "    .dataframe tbody tr th {\n",
       "        vertical-align: top;\n",
       "    }\n",
       "\n",
       "    .dataframe thead th {\n",
       "        text-align: right;\n",
       "    }\n",
       "</style>\n",
       "<table border=\"1\" class=\"dataframe\">\n",
       "  <thead>\n",
       "    <tr style=\"text-align: right;\">\n",
       "      <th></th>\n",
       "      <th>user_id</th>\n",
       "      <th>trt_grp</th>\n",
       "      <th>test_name</th>\n",
       "      <th>pre_score</th>\n",
       "      <th>post_score</th>\n",
       "    </tr>\n",
       "  </thead>\n",
       "  <tbody>\n",
       "    <tr>\n",
       "      <th>4070</th>\n",
       "      <td>209574</td>\n",
```

```
"      <td>&gt;=90 days apart</td>\n",
"      <td>ML_max_score</td>\n",
"      <td>5.0</td>\n",
"      <td>7.0</td>\n",
"    </tr>\n",
"    <tr>\n",
"      <th>7169</th>\n",
"      <td>249406</td>\n",
"      <td>&gt;=90 days apart</td>\n",
"      <td>TS_max_score</td>\n",
"      <td>4.0</td>\n",
"      <td>3.0</td>\n",
"    </tr>\n",
"    <tr>\n",
"      <th>15623</th>\n",
"      <td>418060</td>\n",
"      <td>&gt;=90 days apart</td>\n",
"      <td>TS_max_score</td>\n",
"      <td>4.0</td>\n",
"      <td>7.0</td>\n",
"    </tr>\n",
"    <tr>\n",
"      <th>20468</th>\n",
"      <td>555349</td>\n",
"      <td>&gt;=90 days apart</td>\n",
"      <td>ML_max_score</td>\n",
"      <td>NaN</td>\n",
"      <td>NaN</td>\n",
"    </tr>\n",
"    <tr>\n",
"      <th>3013</th>\n",
"      <td>197521</td>\n",
"      <td>&gt;=90 days apart</td>\n",
"      <td>FM_final_score</td>\n",
"      <td>51.0</td>\n",
"      <td>70.0</td>\n",
"    </tr>\n",
"    <tr>\n",
"      <th>17591</th>\n",
"      <td>474405</td>\n",
"      <td>&gt;=90 days apart</td>\n",
"      <td>TS_max_score</td>\n",
"      <td>NaN</td>\n",
"      <td>NaN</td>\n",
"    </tr>\n",
"    <tr>\n",
"      <th>304</th>\n",
"      <td>174070</td>\n",
"      <td>&lt;=30 days apart</td>\n",
"      <td>SS_max_score</td>\n",
"      <td>NaN</td>\n",
"      <td>4.0</td>\n",
"    </tr>\n",
"    <tr>\n",
```

```
        "      <th>16411</th>\n",
        "      <td>445744</td>\n",
        "      <td>&gt;=90 days apart</td>\n",
        "      <td>FM_final_score</td>\n",
        "      <td>100.0</td>\n",
        "      <td>90.0</td>\n",
        "    </tr>\n",
        "    <tr>\n",
        "      <th>22362</th>\n",
        "      <td>617945</td>\n",
        "      <td>&gt;=90 days apart</td>\n",
        "      <td>DT_final_score</td>\n",
        "      <td>4.0</td>\n",
        "      <td>6.0</td>\n",
        "    </tr>\n",
        "    <tr>\n",
        "      <th>1593</th>\n",
        "      <td>183211</td>\n",
        "      <td>&gt;=90 days apart</td>\n",
        "      <td>RT_final_score</td>\n",
        "      <td>NaN</td>\n",
        "      <td>86.0</td>\n",
        "    </tr>\n",
        "  </tbody>\n",
        "</table>\n",
        "</div>"
       ],
       "text/plain": [
        "       user_id          trt_grp       test_name  pre_score  
post_score\n",
        "4070   209574  >=90 days apart    ML_max_score        5.0         
7.0\n",
        "7169   249406  >=90 days apart    TS_max_score        4.0         
3.0\n",
        "15623  418060  >=90 days apart    TS_max_score        4.0         
7.0\n",
        "20468  555349  >=90 days apart    ML_max_score        NaN         
NaN\n",
        "3013   197521  >=90 days apart  FM_final_score       51.0        
70.0\n",
        "17591  474405  >=90 days apart    TS_max_score        NaN         
NaN\n",
        "304    174070  <=30 days apart    SS_max_score        NaN         
4.0\n",
        "16411  445744  >=90 days apart  FM_final_score      100.0        
90.0\n",
        "22362  617945  >=90 days apart  DT_final_score        4.0         
6.0\n",
        "1593   183211  >=90 days apart  RT_final_score        NaN        
86.0"
       ]
      },
      "execution_count": 31,
      "metadata": {},
```

```
     "output_type": "execute_result"
    }
   ],
   "source": [
    "df_rmANOVA_init = df_init_2_time_points[['user_id','DT_final_score',
'ML_max_score', 'RT_final_score', 'FM_final_score', 'TS_max_score',
'SS_max_score', 'time_point', 'trt_grp']].melt(id_vars
=['user_id','time_point', 'trt_grp'],var_name='test_name',
value_name='score')\n",
    "df_rmANOVA_init = df_rmANOVA_init.pivot(index=['user_id','trt_grp',
'test_name'],columns=['time_point'], values=['score']).reset_index()\n",
    "df_rmANOVA_init.columns = ['user_id',
'trt_grp','test_name','pre_score', 'post_score']\n",
    "df_rmANOVA_init.to_csv('/Users/rye/Documents/Documents -
Mac/BrainBalance/data/df_rmANOVA_init.csv', index=False)\n",
    "df_rmANOVA_init.sample(10)"
   ]
  },
  {
   "cell_type": "code",
   "execution_count": 34,
   "metadata": {},
   "outputs": [],
   "source": [
    "df_2programs_RM = df_2programs.drop(columns=['SS_avg_score',
'test_date', 'age_at', 'age_years',
'cum_interval_int']).set_index(['user_id', 'trt_grp',
'time_point']).stack().reset_index().rename(columns={'level_3':'test_name
', 0:'score'})\n",
    "df_2programs_RM.to_csv('2programs_rmANOVA.csv')"
   ]
  },
  {
   "cell_type": "code",
   "execution_count": 37,
   "metadata": {},
   "outputs": [],
   "source": [
    "n_times = 10_000\n",
    "change_score_dict = {}\n",
    "res_dict = {}\n",
    "i = 0\n",
    "creyos_tasks = {'SS':'SS_avg_score', 'DT':'DT_final_score',
'ML':'ML_max_score', 'RT':'RT_final_score', 'FM':'FM_final_score',
'TS':'TS_max_score'}\n",
    "for t, score_name in creyos_tasks.items():\n",
    "    diff_score_1test = get_test_df(df_2programs, score_name)\n",
    "    res_init =
stats.bootstrap((diff_score_1test.loc[(diff_score_1test['trt_grp']=='Init
ial'), 'diff_score'].dropna(), ), \n",
    "                 np.nanmean, confidence_level=0.95, \n",
    "                 n_resamples=n_times, method='BCa')\n",
    "    \n",
```

```
    "    res_virtual =
stats.bootstrap((diff_score_1test.loc[(diff_score_1test['trt_grp']=='Virt
ual'), 'diff_score'].dropna(), ), \n",
    "            np.nanmean, confidence_level=0.95, \n",
    "            n_resamples=n_times, method='BCa')\n",
    "\n",
    "\n",
    "    change_score_dict[t] =
res_init.bootstrap_distribution/res_virtual.bootstrap_distribution\n",
    "    res_dict[i] = {'task name':t, 'program type':'at-home',
'ci':'low', 'value':res_virtual.confidence_interval[0]}\n",
    "    res_dict[i+1] = {'task name':t, 'program type':'at-home',
'ci':'high', 'value':res_virtual.confidence_interval[1]}\n",
    "    res_dict[i+2] = {'task name':t, 'program type':'in-center',
'ci':'low', 'value':res_init.confidence_interval[0]}\n",
    "    res_dict[i+3] = {'task name':t, 'program type':'in-center',
'ci':'high', 'value':res_init.confidence_interval[1]}\n",
    "    i += 4"
   ]
  },
  {
   "cell_type": "code",
   "execution_count": 40,
   "metadata": {},
   "outputs": [],
   "source": [
    "## bootstrap for testing treatment effect of the at-home program and
the in-center program\n",
    "creyos_tasks = {'SS':'SS_max_score', 'DT':'DT_final_score',
'ML':'ML_max_score', 'RT':'RT_final_score', 'FM':'FM_final_score',
'TS':'TS_max_score'}\n",
    "res_dict = {}\n",
    "dist_dict = {}\n",
    "n_times = 10_000\n",
    "i = 0\n",
    "for t, score_name in creyos_tasks.items():\n",
    "    res_init_1 =
stats.bootstrap((df_init_2_time_points.loc[(df_init_2_time_points['time_p
oint']==1) & (df_init_2_time_points['trt_grp']=='>=90 days apart') ,
score_name], ), \n",
    "                np.nanmean, confidence_level=0.95, \n",
    "                n_resamples=n_times, method='BCa')\n",
    "    \n",
    "    res_virtual_1 =
stats.bootstrap((df_virtual_2_time_points.loc[(df_virtual_2_time_points['
time_point']==1) & (df_virtual_2_time_points['trt_grp']=='>=90 days
apart'), score_name], ), \n",
    "                np.nanmean, confidence_level=0.95, \n",
    "                n_resamples=n_times, method='BCa')\n",
    "    \n",
    "    res_init_0 =
stats.bootstrap((df_init_2_time_points.loc[(df_init_2_time_points['time_p
oint']==0) & (df_init_2_time_points['trt_grp']=='>=90 days apart'),
score_name], ), \n",
```

```
    "                 np.nanmean, confidence_level=0.95, \n",
    "                 n_resamples=n_times, method='BCa')\n",
    "    \n",
    "    res_virtual_0 = 
stats.bootstrap((df_virtual_2_time_points.loc[(df_virtual_2_time_points['
time_point']==0) & (df_virtual_2_time_points['trt_grp']=='>=90 days 
apart'), score_name], ), \n",
    "                 np.nanmean, confidence_level=0.95, \n",
    "                 n_resamples=n_times, method='BCa')\n",
    "    \n",
    "    dist_dict[t] = 
{'pre_test':res_init_0.bootstrap_distribution/res_virtual_0.bootstrap_dis
tribution, 
'post_test':res_init_1.bootstrap_distribution/res_virtual_1.bootstrap_dis
tribution}\n",
    "    #res_dict[t] = {'init_0':res_init_0.confidence_interval, 
'init_1':res_init_1.confidence_interval, 
'virtual_0':res_virtual_0.confidence_interval, 
'virtual_1':res_virtual_1.confidence_interval}\n",
    "    #res_dict[t] = {'init':{'pre':res_init_0.confidence_interval, 
'post':res_init_1.confidence_interval}, 
'virtual':{'pre':res_virtual_0.confidence_interval, 
'post':res_virtual_1.confidence_interval}}\n",
    "    res_dict[i] = {'task name':t, 'program type':'init', 'time 
point':'pre', 'ci_low':res_init_0.confidence_interval[0], 
'ci_high':res_init_0.confidence_interval[1]}\n",
    "    res_dict[i+1] = {'task name':t, 'program type':'init', 'time 
point':'post', 'ci_low':res_init_1.confidence_interval[0], 
'ci_high':res_init_1.confidence_interval[1]}\n",
    "    res_dict[i+2] = {'task name':t, 'program type':'virtual', 'time 
point':'pre', 'ci_low':res_virtual_0.confidence_interval[0], 
'ci_high':res_virtual_0.confidence_interval[1]}\n",
    "    res_dict[i+3] = {'task name':t, 'program type':'virtual', 'time 
point':'post', 'ci_low':res_virtual_1.confidence_interval[0], 
'ci_high':res_virtual_1.confidence_interval[1]}\n",
    "    i += 4"
   ]
  },
  {
   "cell_type": "code",
   "execution_count": 41,
   "metadata": {},
   "outputs": [
    {
     "name": "stdout",
     "output_type": "stream",
     "text": [
      "SS 0.935 0.9555\n",
      "DT 0.7745 0.5913\n",
      "ML 0.3312 0.372\n",
      "RT 0.8317 0.4935\n",
      "FM 0.8626 0.8282\n",
      "TS 0.7793 0.9023\n"
     ]
```

```
     }
    ],
    "source": [
     "def find_nearest(array, value):\n",
     "    array = np.asarray(array)\n",
     "    idx = (np.abs(array - value)).argmin()\n",
     "    return idx\n",
     "\n",
     "for t, score_name in creyos_tasks.items():\n",
     "    pre_hist = np.sort(dist_dict[t]['pre_test'])\n",
     "    post_hist = np.sort(dist_dict[t]['post_test'])\n",
     "    pre_idx = find_nearest(pre_hist, 1)/len(pre_hist)\n",
     "    post_idx = find_nearest(post_hist, 1)/len(post_hist)\n",
     "    print(t, pre_idx, post_idx)"
    ]
   },
   {
    "cell_type": "code",
    "execution_count": 42,
    "metadata": {},
    "outputs": [
     {
      "name": "stdout",
      "output_type": "stream",
      "text": [
       "SS 0.9119\n",
       "DT 0.201\n",
       "ML 0.365\n",
       "RT 0.042\n",
       "FM 0.2744\n",
       "TS 0.4721\n"
      ]
     }
    ],
    "source": [
     "for t, score_name in creyos_tasks.items():\n",
     "    hist = np.sort(change_score_dict[t])\n",
     "\n",
     "    idx = find_nearest(hist, 1)/n_times\n",
     "\n",
     "    print(t, idx)\n",
     "    "
    ]
   },
   {
    "cell_type": "code",
    "execution_count": 43,
    "metadata": {},
    "outputs": [],
    "source": [
     "df_ci = pd.DataFrame.from_dict(res_dict, orient='index')\n",
     "df_ci_plot = df_ci.set_index(['task name', 'program type', 'time 
point']).stack().reset_index().rename(columns={'level_3':'ci', 
0:'value'})\n",
```

"df_ci_plot['program type'].replace({'init':'in-center', 'virtual':'at-home'}, inplace=True)"
]
},
{
"cell_type": "code",
"execution_count": 44,
"metadata": {},
"outputs": [
{
"name": "stderr",
"output_type": "stream",
"text": [
"/Users/rye/opt/miniconda3/lib/python3.9/site-packages/seaborn/axisgrid.py:718: UserWarning: Using the pointplot function without specifying `order` is likely to produce an incorrect plot.\n",
" warnings.warn(warning)\n"
]
},
{
"data": {
"text/plain": [
"<seaborn.axisgrid.FacetGrid at 0x167ddc550>"
]
},
"execution_count": 44,
"metadata": {},
"output_type": "execute_result"
},
{
"data": {
"image/png":
"iVBORw0KGgoAAAANSUhEUgAAA8wAAAIzCAYAAADVm5AWAAAAOXRFWHRTb2Z0d2FyZQBNYXRw
bG90bGliIHZlcnNpb24zLjQuMywgaHR0cHM6Ly9tYXRwbG90bGliLm9yZy/MnkTPAAAACXBIW
XMAAA9hAAAPYQGoP6dpAADBPUlEQVR4nOzdeVxU1fsH8M/MsAwgICCbIqCgiAuKIagtopYmaq
UtlmZG4oZoZiQulVaalpop4k6imWXu5fLVn5pWhuK+byCoIALKvjMz9/cHODoy7DADw+f9evn
ycufcO8/IeGaee859jkgQBAFERERERERERErEpEKs7QCIiIiIiI6iMmzERERERERERqMGEmIiIi
IiIiUoMJMxERERERERZEaTJiJiIiIiI1GDCTERERERERKQGE2YiIiIiIiNZgwExERERERE
anBhLmREgRB2yEQUSPEvoeItIF9DxFVFxPmRujw4cMICQmp9fOOHDkSI0eOrPrXz6qIjR45g1K
hR8PLyQqdOnfDKK69g7ty5ePjwYam2Z86cwfjx4+Hj44OHTVC19cXM2bMwN27d7UQOVH1se/
RLjc3N5U/7du3h4+PD/z9/XHs2DGVtn369CnV/tk/dP19IrIaoa9j3a9bjP+OGHH9Q+rlAo
8OKLL8LNzQ07duwAAMTHx6v8TKRNetoGDQvIiJC2yE0ajt37sT06dMxbNgwfPjhhzAyMkJ0d
DTWrFmDv/76C9u3b0fTpk0BAJGRkQgICEDfn0xd+5cmJmZ4e7du/jpp5/w+vWrXB0dN
TuCyKqJPY92vfWW2/h7bffBgAUFRUhJSUF27Ztw9ixY/HFF1/g/fffBwAsX74chYWFyuOCgoL
Qvn17BAYGKvdZWlpqNniamLfo31isRj/+9//MHXq1FKPnTp1CsnJyJyVqIiqhymDATaVhYWBgG
DRqEr7/+Wrmve/fu8PLywveu/PLywuuuv45t27YhICAFBr1698MorrD9+fFBr1698Morr2D9+
vWYPXu2xl8DETVMdnZ26NKnC+AQMGYOLEiViwYAFF8fX3h4OCA9u3bq7x
qja9euOH36NK5cuYIOHTqoPLZ37164ukj3IjBw5ElFRUYiKioKbmxtOnjw
JALh+/TqCgoLQvXt3dOjQAS+++CLmzp2L/Px85bH//vsv/CvWtT1tOv/7TqhHgb9Uu2mQ=1
zz//oGPHjpgxY0aZ9w6NHDkSs2bNwpo1a+Dr64tOnTrh3XffxYULF1TaHTp0Cm0CMOHD4enpyc6d
uyIV199FZs2bVI+fvLkSbi5uSEyMhIjR46Eh4cHfH19sXXrViQnp7o1atXqSvN6e
np+PLLL9GzZ0906tQJ77zzDiIjI8v9d5w+fXq5UxUf/7uq8/DhQ7X/Hu33bWDQsNlbb
q2NjY4PPPP8fzzz9fbpxE9QX7Hu33PWURiUT49Nn3PUVRUhUHG3btlX5eKL6jH1P/
...

M+zfv19lv0wmw8GDBzFw4MByjyfSJpHAKgiNSnR0ND777DMAwOzZs+Hq6orc3FwMGDAAXbp0w
ciRI2FgYICjR49iw4YN+OSTTzB+/Hjcu3cPgwYNwptvvol+/fohIyMDS5YsUXZ0YrFYeR/Pzz
//jFOnTiEgIACvvvoq5s+fD7FY/bWZkSNH4tq1a3BxccGYMWMgCAK+++47FBUV4ciRI5BIJDh
69CjGjRuHDz74AH369EF+fj42bdqEf//9F7/++iu6du2KkydP4oMPPoClpSXGjh0LNzc3rFmz
BlFRUXB0dISfnx88PT3x66+/4vDhw9i6dSs8PDxQUFCAd955Bw8fPsSUKVNgY2OD7du34/Dhw
1i3bh169OihNu67d+8iNTW1zH9nV1dXNGnSRO1jU6ZMwf79+/Hyyy9jwIAB6NatG2xtbdW2Xb
hwIdatWwdvb2+8/vrr8PHxQcuWLct8XqL6in2P9vseNzc3BAUFYdKkSWof9/X1RcuWLfHzzz+
XeqxPnz7w9vbGggULynxuovqIfU/96XvS0tLw999999/49ChQ8rH/vnnH0yePBlbt27FwIEDMX/+
fAwdOhTx8fHo27ev8mcibeKU7Ebm6Q7t8dS68+fPw93dHUuXLlU+1rNnT0T0RGRuLUqVMYP348L
l68iPz8fIwbN06Z3Nnb2+Pw4cPIzc1V6SQvXryIcePGoV+/fuV+aDwmk88kQHh6uPEdOTg5CQk
Jw7do1dOzYEdHR0XjjjTcwa9Ys5TGenp7w8fHBqVOn0LVrV+X+N998E/7/gAAY2NjDBs2DB4
eHpg8eTIAoGPHjjh8+DDOnj0LDw8P7N69G9evX8fvv/+Ozp07AwBeeukljBw5EosWLcL27dvV
xuzo6Fjte4e/+eYbKBQKHDx4UPmh4ejoiD59+sDf3x92dnbKth9//DGysrKwfft2REVFAQBsb
W3h6+uLUaNGwcXFpVoxEGka+x7t9z0VadasWcY3Wogakvy99afv8fPzwy+//ILLly8rZ9Pt27
cPffv2hVQqrdG5ieoSE2bCCy+8gBdeeeAFFRUWIjY1FXFwcbty4gdTUVGXxqc6dO8PQ0BBvvfU
W/Pz80KtXL3h5ecHDw0PlXPfv31deMZ09e3aFHxpA6auSjz+Y8vLyAEB5P29f333aubi7u3r2L2NhY
XLp0CUBx0ZqneXp6KrebNWjumjP0xCwsLAEBWVhaA4qJa1tbW6NChA2QymbJd79698f333
wPm5ualYlYoFGAoFGW+JoFIpPYxU1NTLFu2DPHx8Th27BhOnjyJkydPYIiAr///jvCw8
OVH4YGBgb4+uuvMWnSJBw7dgwnTpzAyZMnsWXLFuzYsQOLFy9G//79y4yDqD5j36PZvqyanI
sUUPBvkc7fc9zzz0HW1tb7N+/Hx07dkRhYSEOHTqEhQsXlnscnckbYxYSYoFAr88MMP+OWXX5Cb
mwt7e3t4eHjA0NBQ2cbBwQGbNm3CmjVrjVr8PvvvvyMiIgJmZmYYPnw4Pv74Y+UHRHx8PF544
HkSoaGhmDFjRoXPb2RkpPLz43ONTnjuuecAlF5oGfP/7
T09HSkpKSUKkDxWEpKitoPjzZ2Lnzp1lnnfjxo3w8fEp83Gg+N90xIgRGDFiBBQKBQ4dOoQ
ZM2Zg7ty5pZZRsLa2xltvvYW33noLhYWFOLhnb6nLElJSWjT
pk21jiVqSNj3aKfvEYlEePXVV/G///0zzSEpKKvdYIm1iwkxYs2YNI
iIiMGfOHPTv3x+mpqYAoEzOHvPw8FAuNXLmzBls2bIFq1atgpubG/z8/AAAbdq0werVqxEaGo
q1a9di4MCBpa7GVlVwcDCBsjLy8PWrVtrdF6geLTX2dkZixYtUtu4g4O
D2v1BQUEYMWJEmedt3LixfMOhzA7Nmz8euvv6q0EYvF6NevH06dOoXff/8dAHDhwgVMmDAB
CxcuLFXcy8fHB6NHj8b8PQ+fORlpYGKyurcl8nUX3EvkdzfU9FYmJikJycjOHDh1freKKGhH2P9
voePz8/bNiwAZcuXcK+ffV+pYIm3hsFQj9Oxo5JkzZ+Dq6oq33npL+aGRSmz
dvKq92RkREoE+fPigsLISBgQF69Oj/BBbR77BgCQmJioPJeFhQX09PQwYcIENG/eHLNmzSo1fai
qzpw5g/79+6N79+4wMDAAAPz9998AAO7ooMrw9vZGYmIirKys0KlTJ+WfhdIb
Dg4OKu2f/VNW4Ys2bdogPT0dGzZwnZGXl4eNm7cqPZ1xsbGwtrammuhU
oPBvucJTfc9FVm2bBmkUimGDBlSk5dFVC+x73l3C231Ply5d0KJFC/z55584cuQIq2NTg8AR5k
bIzMwM586dQ2RkJNq3bw8PDw8PDw+sWLECa9asQZcuXXDnzh2sXr0ahYWFyM3NxVjQxdA6ns0B1ub
4//33IZFI8Ntvv8HAwAC9e/cu9RxSqRRhpe9Ehw4dYGn
h3PnzmH16tUQiUK+Kpr6NCh2LRpE/z9/TF+/HjY29jv//+w9q1a/H+++/X+1XP1q1bY+zYs
Vi9ejXu37+P1157DXZ2dnj06BF2796NyMhIrF+/HgBgbm6OkkJAQzJ49G8OHD8c777yDli1bI
srC//3f/+HnTt3YtGiRbznkBoM9j1PaLrveezBgwf4/48gOLCQ0lJSdi5cyf+/fdffP311
FB4l0BfueJ7TV9zzt1VdfxcaNG9G0aVN4e3uX2/b48e/48ePIzMxUew72PjfXHoDdDRFkdNQQn
M2YM5s+fj3HjxiEtLQ0bNzyEWF7O3t8frwZrdLLeUKhYUGjCpYfVwMoSsWqBSvRG+++Fn
M5gN0zMzN4eXlh27Zta2+LlR+eKr7+FoZw2tkwYMJRHzAS7nvN3YbdLi6s3u
+Xo2LEjfvrpJ7Ru3Vrt8/+fHypUr0b9//2pXdF6wYAG++eeYb5ZVdZ2dnfPXVV/jjjz
9w+vTpav87AMUVJX/55RCxcuRFZWFlq0aIFPP/UH33OUY3OXZapU6fC3d0dW7duxy
5c5GdnQ0zMzN4eXlh27ZtaNeunbLtu+++CncnJ2zcuBE//PADHa9yoS
aQP7nie00fcAwLZt25RrLevr68PGxgYdO3bEpk2b4OXlVfPS6RN7Hue0Fbf8zQ/Pz+Eh4djw
IABFdZg2bNnD/bsUEBSEX0KfArIJJjwJYOLDMrlp3fP2zqr/5ILn7N/cSfDVqE48a4yLRWz
yYiYiIiNRgwkxERERERSkBhNmIiIg3btWsX/Pz80KlTXy+RUREWL16
MF198EV26dMH777+Pa9euaTQbFyIiUxDREFwzZLHBhw7Bnzx74+f/lh6tSpHFu
nNr2c+bMwbZt2/DNN99g+/btaNq0Kd+aMGOYOsrCwNR05E1PhwWSkIiIiDREEAX379kX//RE
hKi3D969Gh4e3tj3LhxxKu3v3buHl19+AIDMzE2+88aQjJ16aDJ8IqJGR0
/bAdQHcrkCqak52gbdW2q7RBqHfsfooahtvqf27dvIyEhAYMYMHD1bZHx4errb9v//+zM
zM7z00kvKfWZmZjEiN4mDfQ9Qw6OJ3n4aGU7KJiIiINCQuLg4aKjubi9GjR6NHjx54+
y0yA4+Li0LJlSxw8eBBBDhw7F888/jzFjxiIiLSkOzsbABBASEgoKCE
BwcjAMHDiAwMBDr168vNcU6OzsbdC+/exYoVKzBt2jSYmZlh5cqKVbvVeXo+BD6

ETIqKKMGEmIiIi0hB9fX0AxfcsDxkyBADg7u6Oq1evqk2Y9fX1kZWVhSVLlsDFxQUAsGTJEvT
q1Qs7d+5EQEBAteIQi0WwsDCpwSshImocmDATERERaYidnR0AoG3btir7XV1dcfToUbXt9fT0
lMkyAEilUrRs2RLx8fHVjkOhEJCZmVvt44lIM3hhS/vqVcIcGxuLoUOH4osvvsDQoUPVtklJS
cH8+fNx/PhxAED37t0xY8YM5QcQERERUX3Vvn17mJiY4MKFC/Dy8lLuv3nzJhwdHUu19/Lygk
wmw6VLl9CpUycAQH5+Pu7du4eBAwfWKBaZTFGj44mIGoN6c/NKUVERgoODkZtb/tXOTz75BIm
JiVi/fj3Wr1+PBw8eIDAwUENREhEREVWfVCpFQEAAAwsLCsGfPHty9excrV67E8ePH4e/vD7lc
jpSUFOTn5wMoTph79uyJkJAQnD59Gt2bRokEglef/11Lb8aIiLdV28S5tDQUJiYlD/lI
DMzE6dOncKYMWPQvn17tG/fHmPHjsWVK1eQlpamoUiJiIiIqi8wMBCTJk3CkiVL4Ofnh//973
8IDQ2Fj48PEhMT8cILL2Dfvn3K9qGhofD29kZQUBDeeustZGdnY+PGjbC0tNTiqyAiahzqxZT
sU6dOYcuWLdi1axd8fX3LbGdoahAhjY2Ps2rUL3t7eAIDDu3fD2dkZ5ubmGoqWqH5QpCci/79f
AADSniMgbmqv5YiIqLFg/1Nz/v7+8Pf3L7XfwcEBN27cUNnXpEkTzJkzB3PmzNFQdET1F/sf0
jStJ8yZmZmYNm0aPv/8c9jbl/+GNzQ0xLx58/D111/Dy8sLIpEI1tbW2LRpE8Timg2Wc2kFam
iyTvwKefxlAEDBiV9hOihYyxERUWORH7lZ2f/kR/4K4wFTtRwRETUW7H9I07SeMM+ZMwddunT
B4MGDK2wrCAJu3LgBT09PBAQEQC6XY8mSJZg4cSJ+/fVXNGnSpFoxcGkFaoiyMhKf/JCRyPcw
EWmMIu3+U9sJWoyEiBob9j+kaVpNmHft2oXTp0/jzz//rFT7v7vXv3YvPmzfjrr7+UgyAyfGqVavQu
3dvbN++HaNGjapWHERiREpElaTZi3b9+OR48elbpvefbs2Q
gPD8fevXtV9p85cwatWrVSGUk2NzdHq1atcNTSCIKhs8z1MRERERFS7tJowL1q
0SLlswmP9+vXD5MmT4efnV6q9vb099u3u3bh4KCAhgaGgIA8vLyEB8fX6kp3URERERESVpdVK
V7a2tnByclL5AwEBWVlZo0aJFqbUI33t3dVy/fh2ffPIIJDAwMMHToUG29DCIiiTcP36dVy/fh2ffPIJDAwMMHToUG29DCIiI
iIiItJB9bo09LNrEdrY2GDz5s0QBAGjRo2Cv78/9PX18euvv8LMzEzL0RIREREREZEu0XqV7G
c9vfagurUIXVxcsGrVKk2HRURERERERI1MvR5hJiIiIiIiItIWJsxERERERETSY8W
KFRg5cmSZj3/++++efo06ePSPBiMiIiIiIk1jwkxE9E9IyIiIiIk1jwkxE9IyIiIiIiItKG
elf0i4hIW5KSkjBr1iycOXMMGrVq1UtsmOTkZX3zBBy9vzGQkKDhCImIiIhIkzjCTERERERE
zA3N8cff/yBzp07pcEARMnz4dr7/+Ory9vbEPnJmvZZQRQRERFpEkeYiYiYiIiIiCSHK9O
JVq1Zh9h9erVtfa8enkkNi0gkgvDUNtkgvDUNt/DRESkq5gwExERERERERERERERERFJmEO
iWFiY1Nr5iDQhSyyComSb72EiItJlTJiJiCpQUFCA4OBgBgTJgwAe3atavUcysUcysUAjIzc2v1nER1
TaEQVLbT0nK0GI1m8KIAEVHjxISZiKgCFy5cwK1bt7B8+XKEhYUBAIqKiiCTyeDp6Ymvvvo
732WrXPL5MpKm5EVI8IgqCyzfcwERHpKibMREQV8PDwwMGDB1X2/fzzzzzzh48CB+/vlnWFlZaS
kyIiIiIqpLTJiJiJiCoglUrh5OSkss8SLss/c3Bx6enql9hMRERGR7mBZSyIiIiIiIiIi1OMJMRKTGggU
Lyn180qRJmDRpkoaiISIiIiJt4AgzEzERERERERERERERERkYbt2rU
9efOnTsajJiIqHHiCDMRERGRBu3evRszZ85ESEgIfH19sWfPPHkydOhV2dnbw9PQs1f7Gj
vbGDz/8oLLf0tJSUyETETQBCxduhSjRo3CqFGjJAAATJ07E2bNnNnERUVpTZhvn
nzJtq1awdra2tNh9ugKdITkf/fLwAAc8REDe113JERNQQMWEmIiIi0pDbt28jISEEBgwcPVpV
fHh5e5jE3btxA//+M9LW7/CeMBULUdERA0RE2YiIiIi0DYmLiwTh1SN
0bh69SocHBwwYcIE9OnTRyvxZ2dnrPQlHcxHxfL+2dRAgHVVHQUWQLLAnotX8Ohq8C0jCcA
kJ64lPb93X+9TYWIpEIwlPb/L1SXWPCTERERKQh2dnZAIAhISEIDQ2FtLQ0hIWFoeXLl9o0FBYWGUw4cQGeINavX4
8ePXqotL958yJeCZiHMjB9erytHzvkdVWrFiB4OH488TNHKO3yoAUSP7dvWQUiVeQUsf7cbgzh
y3Rheb2PB3ytpGhNmIiIiIg3Rl9cHAIwePRpEpRpDhgwBALi7u2Pq1asrt1W3bsQu3b3eq1atqE+bu3bsjKioK5ubmyn1h
YWHo3bs3duzYgbFjx1YrDrOVCQGZmbjVfRcOgUAgq22lpOVqMhmpLY/u98oKA9jFhJiIiItQO
zs7AEDbtm1V9ru6uuLFiEllJyyhwhs4Lh7hyG1wDTNb8gjQ5qMSTfQdUVzqBALqj3mRjaVFVFKL
3jpH8iIiIiDWnfvj1MTExw4cIFlf0ibtwwcIFlf03b96Eo6Njqfabn2+Gj48P8vPzalFuys7MRFxcHV1fXoo+
XiKixY8JMREREMpCFSqRQBAQEICvwDnj17cPfuXWsP89Pyfn7XYrEwdDwzxwZ7ynnilAlslUBclEAgIQgC
pk2bhlu3uHSpUuYNGkCtl1T06iYio7jBhJiIiIiItKgfgwMBAYGBgIDKEF6UlJUuWLGC2ixGafnxIp
D4+PkhMTMQLL7yAffv2sjffp0AQDs7e4XybNy4EVKpVq9tSnTEVTMuvhIbI9/EeZi
IiIiIIN8/f3h7+/f6n9n9Dg4OuHHjsMRZiIiIiIiIiIi1mDATEERERERERRqcG
EmYiIiIh0iiAIgFz25OeCXMiToWJJCIiqgzew0ERERERpOkOemoD8Y+sg5GU82VmUh9zdcyG2
bgVprwBILFtoL0AialA4wkxEREREREROkGemoDcP+ZBkRKr9nFFSixy/5gHMjooaKCTMRE
RERNXiCICD/2DqgXh5LNRPJWlVSiIiIIGjxFckyZI8ul2qbEouh2qbEouh2FBR5mRAU8j
qOjIgaMt7DTEREREREqNnnuzOuSq1Lzi8EgWPfzAwhkjaBCLDJiV/mxT//Xjfsz9LmwB6hhCJRLX
+OoiofmHCTEREREQNnlCQUO/2DC3MhFOZCQHLljxHrPZVAmzxJtlUS7yaA9KnHDU0gEkuqHycR
aRwTZqIGKzlMsQ2LrzaTUREjZLI0ESzT6iQQchNh5cbXrXjyhrNLifx5mg2kfYwSZqYLhcB
hERUWl6Tp4oPL+30u0ljl0gEosh5GdDKMgu/js/BxDq+J5mjmYTNShMmIkakMfLZZRVAfTxch
nGr81i0kxERI2K2MYFYutWlSr8JbZuaBP+H5catRUEASjKV02ilcn04+2c4r8LcpT7UzRXy+
rWLVHs43KTK4b4mg2Z9iRNjBhJmogqrpchvEbX/LDg7ROkZ6I/P9+AQBIe46AuKm9liMil0l

Eokg7RVQ7oVlAICBMaS9AtR+RopEouIk08AIgHWln1tQyJ4k0s+OWD+7T6Oj2XkQCvMgZKVU/
piKRrMNTUpGsp+ZVl7Ho9mcYUfawoSZqIGo6nIZipTbkNi41HFUROXLj9wMefzlku1fYTxgqp
YjIiJdJrFsAePXZiH/2Dq1n5l1lViJxHoQGZsDxuaVPqbc0ezHo9fP/txIR7M5w460iQkzUQN
R1eUyZHFnmTCT1inS7j+1naDFSKi2cEok1XcSyxYwfuNL5Gya8mQ0Ut8IxgODIbZuXW/epxzN
Rhmj2SYqybZgaILCU9s4w460hgkzUQMgT0uA7N7lKh1To+U1iIjU4JRIaihEIhEgefI1V2Ror
DMXkWs0mv30Pdj5ZSTXDWE0u6zTcYYd1QEmzET1lCL7EWQxJ1EUfQKKR3erfLzGl9cgIp3GKZ
FEDZfKaLZpHYxmP5VkCwXZgKKOR7PLwRl2VNuYMBPVI0J+NopiT0MWHQl54k0AQrXPpefctfY
CI6JGTSGXI/+v1ZwSSdTINMTRbM6wo9rGhJlIywRZAWR3zqPoViTk8Zdq5aqs2LoVxNatayE6
ItIVT77E5hT/Kcwt/qJamFs8WlSYU/zltjDnSZuSPyis/BdZTokkatxqczQb+TkouvUf5InXK
//8nGFHtYwJM5EWCAoZ5PFXURQdCVncWUBWUG57sWVL6Ll2h6SZE/IOraj2chlE1PAJ8iLVZL
YgB0JBbsm0yKf/Lt2mzov+lOCUSCKqqrJGs8UWzZG7e26lz8MZdlTbmDATaYggCFAkRaMo+gR
kt6Mg5GeV217UxAr6rj2KE2VLB+V+bSyXQUS1S1AogMLcUiO5qn9yi+8RLBkBFkpGgCEv1Hb4
FeKUSCKqLWIbF4itW1VqaU3OsKO6wISZqI7JUxMgi45EUcwJCFkPy20rkppCr3U36Ln2gMTWV
e0ocUNZLoNI1ymnOKtMbS5rirPqCHBVpjhrjZ5B8dIuhsbFceekVvpQTokkotoiEokg7RVQbt
FBAJxhR3WmXiXMsbGxGDp0KL744gsMHTpUbZuioiIsW7YMu3btQlZWFjp27IhZs2bB3d1dw9E
SlU2R/QhF0Schi46EIvVe+Y31DKHn3BX6rj0gcWgPkbji/5a6vFwG6Y6Gsl6v6hTnklHdejbF
udpEkuI1TQ2MAWkTiAyMITI0UfsHyu2SNhJ95WnkSdGcElnLdu3ahTVr1uDevXtwdHREUFAQB
gwYUOFxf/75J4KDg3H48GE4ODhU2J5IF0gsW3CGHWlNvUmYi4qKEBwcjNzc8itwzpkzB0eOHM
H8+fPRsmVLLFmyBGPGjMH+/fthamqqoWiJShPys1F0Owqy6BOQP7hZfmORBJKWnaDv2h16Tp4
Q6RtqJkgiDdH0er26PsUZahNdY+UIMMpKgvUMa+XiBKdE1q7du3dj5syZCAkJga+vL/bs2YOp
U6fCzs4Onp6eZR6XkJCAr776dSoOREtUfnGFH2lJvEubQ0FCYmJQ/hevevXvYtm0bVq9eDV9fX
wDAt99+izfeeAOXL19Gjx49NBAp0RNCUQFkd86hKDoS8nuXKxoi7Qc+lO/Rbd4NI2kRDUR
JpVnXX663UFOenkt+GOcX5mYTWwEQ5Aix6PAL8zEgwDIwhEou1GjqnRNYeQRCwdOlSjBo1CqN
GjQIATJw4EWfPkVUVFSZCbNCocBnn32GDh064MSJE5oMmmaje4Aw70oZ6kTCfOnUKW7Zswa5d
u5SJsDr//vsvzMzM8NJLLyn3Zm4pT4dqqQZXHxLhcfiJtYaShKI
u0QBAH5x9ZVar3e3D/mQmRuD5FyZLiBTXEuY1S3MlOcGyJOiawdt2/fRkJCAgYPHqyyPzw8vN
zjVq1ahaKiIgQFBTFhriSxRXPIsx+VbPN9SUTVo/WEOTMzE9OmTcPnn38OWhZcu
WOHjwINasWYOkpCS0b98e06dPh4tLza4u6elhaJ/o4v91W+CoID8QTQKb0WiMLriCtdiM2sYtOkB
gzaqFa5rk0gkgvDUNt/DVB8okmMqNW0XAFCYByHltvJ9rFGlpjgbl5Hs1s0U54aKUyJrLi4uD
gCQm5uL0aNH4+rVq3BwcMGRP/30E7Zt24akpKRai0XXPzdMXngfuf/+DA
AwfmEEJDr+ehsLfv8hTdN6wjxnzhx06dKlSx3d0SQtMHA34cT4SbnbKs3H37l2sWdX3hdU7lrdGcmi
j3759sLKq3uidWCyChQUrel24UEsTnRQJEf+eyjFoL8PWhjd75iCl5B
IigePzffw1RPyO6c09yTVTTFuayktx5McW7IOCWyZrKzswEAISgLbnYKoPuD3SuhxBCbIrqbwTAAMcLIJAAWECsBJM4ea6/Nn2WMJIPDdaS3dbBqLi7O1GqaJ1MuHK3nH2gHcY6tKmTOZN6rNkgKW3pVh38Y8iZbkTu4zhmHMVf0yaJI9/M/ENB4ZQPDAV/
7++1O1lubm5CA4ORnBwMJydnWstYW4UnxsWrkBr3vOta/j9hzRNqwnzrl27
+vr4+srCwsWbJEOaK8ZMkS9OrVCzt37kRAQEJRj2KRQOHNE5BXVOF
aXwqDVs/BoG0P6Dl0gEgsQR6AvPS6fz8pFILKdlqa7q97yg/F+q/q6++Kiu/ZLWdUV3XU96lE
uIFPcabGSV+/+H07evRoDBkyBADg7u6Oq1evqk2Y586di8rdnZ7z77ru1Gge/+1BD1di+//C7j
/ZpNWHevn07Hj16VOq+5dmzzPzZZM8PBx79+5V2W9nZwc9PT2GdWwxX6bLyWrJYzn8jWYRWfU2YJ
Y9ngDGq3rpHcJ4zaMkUcp8cR0Vy1ZNE95xgR4GPFgfzHyb2eHCl5/rxfKi2tBcPw5QuwAtoFTz
YHAwqzN9pBFhAGfiPrLz/LqYZ9Z2+IqyTH8pJzm1dTVJW+kXzjPMiO8Q6HgHcKhBEWKUF2n2
36igaovrHzs4OANC2bVuV/a/6urjh69Gip9tu3b4eBgYGYGJcXnyf/6BBg/Daa6h66+/rnYs
/NyghojfffOjTtJowL1q0CPn5+fi+Sr7vXh8mTJ0pr0dAd1/8SmMfIHjPdNTjTsQjJM04xlIWHHzr
XsYOHCgRmIm3SEU5UMWdxZF0Scgj/tvKjIEG1z9GFa9fu0G/lxQrXRGXQc/JE4fm9FTcsod/quT
qMhqj+ad++PUxMTHDwgV4eXkp99FHs9mpKVzxtqzevV7Vx1x/hYiIKqb
VhNnW1lbtfisZGzg4wDjFS2x8n2/KdLhGIeqAvObyDbVwixoiv7pkAWbuk8fU/IeIPkU7/ZlHEq6bCRaLHu8lkm7B
f13D0VNDJChkkN+7XFzh+s5ZoKjaGkE4e+1ZsbMFC0W+e+ih2S4Fn2NKFFdcbt+E9QKLLD+E+7UvkgAQBuKGbj
Hh4YO/evTh+/DgiIJKffdxcnJSOf7BgwcAgObNm7dkgsREVe1ot+lScXMRF9+/bF/PnzMX
ToUADF6zUvWrQIQUFByM/PR9euXbFx40Ywp5F5wByEGRM4fv/DgIIZ/ZUsHl1KMNXRZydRpB3YPdxu95lhL5j+5ZRXbvu2m6iN4lHIrBD37u7KmCNNe4qtV/VQJNSS/9EaLbd6YNMYSAdE7QQQJ4TldERXWAacVoSKV2HBSWV+Mg0X3WTM+5fIjQ0zrhajrKvzLVY9UuSu+vLLQcmSY2IVLRPULgOjNXzkyQtChs5Sok/30N1BKEmE2HGhjvyZFmN7KEzzZ5eJ/e/zhzd92wCIjZW6Dgom0I/IKPyBP0J+nMDWGi5QUnaYHJLxGhrg6HD6H0tPeEiZNpa
QzsZB6ruQ9h87tmXYkG7kuM7ZnE0+bj2EyEjb6bcFZvl2Sm9yTc29DHtRv46TDzmUqK8xSo/PZt/OZO0evDd9JWmvMk9s90UjZ4JjpQ8Gw6e1eMkq7OhKLQ1wxZ3eVLnGERWXN3fd5wsmmdGoMF7HwxYd9OgkP4uuXWPkpZcIiczqpLMw+ufb98SX4
d8ByB4yMe/OXJ4kgw3DjGwHTn0GwzGf34kSU1IobAnwVLBrDq1F6zPjsyNAH6/rtGN7pzCvUONIlI6/OQTKCvKiNSNS7JbDQp5N1s+ZSiyqwx7ecvTHQOXJrpsswWWI0O8MHRP9gJ4l/9bSfuR6MgBNzGOzjI6ZeEfzJiU3nK1cbg9Ky+bRdhk4NSMOLJRwaIRKjaRPFYSKbGX
c5IWuV3Jr2O/Qw0WcmC3nuqcyIMHu/HGgcnl+7fYpYHnqwD4ebqNKaBuQSJzEG6eyU8bYYjPHlcnqb9YpuHqnhwMKIqHYtSRBbQFHUBr8Ks9UQWNSkzDRi1J72IwzZp1CmXzRWwpRE9nT2WR0TuV6f2tqsmHljTwiEXqMi3YDKMAUWCiDzf2VPVzAMjxYpJd7d7yZkxC2olG93p1Jz/ZN7C
SrPGJfA4Xbt9yo9wyJRJu6FTB2l8hTQnRvCgxrS+ZAFzxJ9AJDD+vBG+kbqBRz0kmIFdYO2/GtO+p8xN23W3CVq6FYf2c4MM9xBUjT32uBHcG21fA8z6PvDvsEWJJUxWTyK7SYIS08HTDK2MBeyZMmXQ/NmAI9R7B/IB1X1cbNy/ME1HCmlqo/CiFw+CWqEiFG4cYHK2zmyPVeuOSB0ndtw0oY/XA2gIr3MzrYeZGcJkcfhItZFRvuaXZn+YgKj2zK4M58TyC1/MUeAy6VIwdgQiLo2hfZB5gpFaGf3m4gUCtOhe0n0Vdgr9bmMgKfJkDOUfTH8Y0ObKFPCqAgg79HSzJ7+Fbc5QxOGXhI8LR1gEq8Aqv0v+sw1H2Yw2bIhUE2F8Jap7BiwO7PgjtC+KBovdEdQ0AmkH9NqzHBvVJGUpq3s96NKT2FujKAJSuXfsAqpdK4p9E8aBxWpyvO0KYpvm8yp9ZFb0NuqxZ3PcBMZWI5ORbxU4WvhKqFoQsvyIOFXKIdLvn0hdBTKehCZGxTOhA7FSD4c4r4Y5gU0dBONAvJEjP/r8aI8Il5C1YFPFCcxaJhp9WVcTJpDbw3sXCHKJVB5W5lK6eRUgi28f4JdMUAiBpyU57/OM1Tm9BaeGi5CbNrr3hQ8X1vX9AIgqlp1Kt5LKb8rE0cWkdFxsMHPVtDvH7rLH+U2OyAFX0XvKuHrZYQ9wkMCoZLRXuQwGe8l+b6K2s6AXvU2wMFFNLX6IDZwOVe+zXLXjbQfdgyh+yXMjKyF5I8qhkVqq0p3RqGaUIkvFEnmyLSnROUZrvj2xT6pFgR+eiN6X9WAA0WW+AXKPLGz2O1bhY53ebJbEsRPKFHDXxDzz9OFBTzwTqUK3yWumhtmr6eovgO3PgMEX1l1P2E/GFdePCVgW0+WrSQHfYsQMs+Ocg9MjOJj5yUnODqVT1WqfgwNUuVtvJuzb0PaEYXi0KW5yH+tRZtYGDQ93ZZwrdYCDNTaWALt8fKCZgKY7bbMwjpOwrF/UrEGmzrq1XhQJR41UOwGN26lNnNA6h2CKCjhNr5r06ZkCHVdOBHPDGtHRafPbCA9lEU4TNdL6RzqDkf+4FWqbKV4wEgSAXT5wNbY/3yFWHnyaBwS2FqAxF6l/N8UUmuQ/dS19rybx4uc2yXXMb4ifBO1P4AY8r5PR5vo1KiqCoEUb3K2lsrk4KDW4iwCpTq6fwmGDGdgCNdwIP+DsaI0I/TSRDzG9Z/KVPc9/YgVdQw1rj92y8/ol03kSrHyPMGBnj5bkWT0J72NV9EhRJCu9CuwQyQnOvWt5nUf0K2zG/6/Y0pUwpO7gPsk5/f4XqhhmuTiEXqglf92RMfzRNBYaQXNhHRVXOBJmw2T9RXkEYp2aX4ZLfF/KpKBcFR63bBkP8Adr8tbGe8UC/5lrf2BavzPn8YEeyXqUmqv2tfeAAhFTTi4Gr0bYYHpI2U4T0dTxoq/+YcGWA0HjnXzt9Bnw0nM9u0ITy7vlJwmD54kQ9lPnNA9WKYHq0wbMCa+sDDg0q1y6NvkfjWVeclvtCiiGFFnx9Y4J4BDTsdrNkSOxtYVRLzzHsT0YdAMu7CxqKV5Q/sWwYh1t7pqeH4uAbAEwESe4oGBGyHKXjvlsqLBHkWpkCq7r6FHTCEO/y8yOf6IfEyeZOGGECJH8dzIPQ7mUAxEc2YQGxeJp+UC7LTsU3NuJAv4rgRa+yWpHVlXNPRVQFVGqRjqoJ/XbvXp2pPFTmLSp1UR9Ixe8aX9xt4Bc9zbHW9WZXXQdA/KnKCvm5l+6gMz0JHKFRWtTvs6NX9DuLcHGxI0BzZ7VK8ZbDTwRq2u3yQxhpaPxmQBfPvVOCW0WDRVUpZyQ7WIfFaC7AeuWSHuS+7p2jmTN8wXz8c8bhbLjpq7YKcDTgV2ewpo/AG39B3f8OWW4smBY4a6D7jHKAf6zWnlnUk1L2cUzw3Y4Px2dFyMkvk4Gs4qj5lDYjR5QO5NAAO74DFxoFRfTRmtr3uNiEX9ZDfUu0HcWFqNDQyMjP6E1qqQtHwk/9z5P0AZmFFmhkTZfWC2EL0EJsB1LB/TW5KsTzYxwLPqJTOGE6k3Y3Qn0ApV2pM2GdvU3IucS1ugufFJRVwO3WQKQXc0Lq9uz6oYEjuEJdp5YdtxA1DDO8IKd1+9rbmy3aumTTyJHR+OL6QpeoDPF3QcSe7xUp1d2I19GUkn+h+1yBqJkYiw3dezUqo8hskVBYnZ1BKyfxPOhxJX8KsnMVVqq8e9cMw+ZkCHR1LlE4kz1LsE3cXf/5f/XpKQnwi0Fu2EF6Ti9CzdRVTMAW/XL9uXvYnCXj0HDKNLIjNRXlv0pIhtVl+CMcIvFHUTNY8k7kJNMU/EjZTqpZkzQoYbz4eGD6jcDTAUb0uSFTvOhHHi3GJ8tdaJPpSohUB+qJ0Kq7x1SY9gzlnGE5Yaw3CvvlQ3sYVyOwNVV7ad2AQ5iEsK6bg47x+DhUYsqWFq2XKAUfSIEHq5XXXk0YNnlpXDkY/nwq99qGlxYIg/aFJROlhM8r8r2jWW6n5JaCwXmk0/GkkjTbRbwyxTzq29Cj4dt4Ahz+z0mu8KB8gm4f1eSNqE9CrCm+ZPPp7qmNLlbrTMpTmkJ0PKiZQrNb8eNQHIEdLjiAN21OEz4oCd1kzqzE8J9u8XYrkh6KfECpAVoMJbFM6aFV6U3Myb1e7HJbZeVrf5cJkgwVjtCsBdrwv4hP0rBvdpdVnC3/CMDfvg5fRbhRxQaNCepWcIazNBSxKs0ScfUSXNmqX/6vMDfBLq9SyD3DxrjEsNdv3VTpaV8h4lhnVr7BcBVW5XRHXmENzQJRIuyM7ShWvqVlqR3+I2WrekjxINqZO0jBRuvK+UACwXqOqkNbcJdOG9bNcE5FG+pjFA8tmsXpvr2uz07/lBYrLWKQ+ab9KK4kcjtNF5Mvj1kPm7XKNaUhr1DJDYBxVD2bSsLyqCYMYpVmJFDYavkGxW8wpjXCRTEWx9ZBxG9/cyc1l0dnN7+TsWkYcIZ/O+5Q6vwWLgIVh8pVXbpl6Eclt8kJoBe5/8E5ahWdQCq8Gn8HhtU95Q/xqHfoR9Dev/Ur8tAfH33bvzL6JL2CyJdYzXg8SZyJ9kb6ZLH7bvbmG+YUQ1xN8AFQH1i5Vs/fn07LkECXb3cvBE8j1UA73FhAW1vycADtnuXBzG7f2ikZG9H4N2Vc9+cF4XgNaMvzFu2bK8VJqEeUMgvQOCaHj/W1F04R/uWzoDBDFvXvR+2kaRm0Prqo8ZE2kvjVTF1GYo2kI4IOP4NBt0u5jS0BA3iPAqBJ7WjTK5XTLBMrnlvctvvRozN42UdBP8x8ToVHdyOuqLzLzzvKVkGUNUvZR2QpHNeAwqGKUJ8QZZbnqRt5y7wHvBNJWmwnLpIk/OjFeMTTE86zWEDmGYxX2aPwHU5hY0Kjn8whp5X6RW2jqjQJIdqRhwuDq+Zsd9ZA1Q/1VR0IkCDhiiWdIL1LEuq80KI0hPPFEz0OtMzzUUB8kxX9BCPy1i5U2rs6yobi3x2TQx1tQwgMhKjDy+ZXMSUK3gJY8BFF19+3wrOkJg0Ba2PZXeSDeKHrP90NNCLdhkE+WEUl7SAK3b9dLn9gm6aLX6yI9VQbRNaD0S0e3v0S6wXQZW8HNV0bJyNCvczL1X8YfN5a8p+8dzPbJKgIEP3uxhxz8Pup4hxJA2WH6l2YAzg9bmiyzkIwc4u0QrvTHnLpqWkVC6eNfDGt7F1xr+3fNmtNGF/ru1+IwgvP3cNxmn97A7mITi9kJqI8a/A0jdbH4vbBDpXjAs5d2n5Tq0YbhrnQkt5gU6G3K3Q0cM2JVMsaeUOvrMk1a0ezUPOUfNoaYbz+Sn4tMPO0WF5H8y0NL1AxLyGEXOmLKYm34fLmCUmqYBhODRNEnDTUL4jKpgNpVyMz0a40Yq3PNgbZNZHvV1gKz0XeQ3tJ6Y4fX1xRNPbFpvAf4mqZmSfh+4/EJDgbDSFnvB5Upr7EjXQJ5h4PavNypGWrwvCU4fgp1tZuW1fg2ZEMMmVONZCnpXV0c6EQfAcGyqXOJvGzL3Zj3KNR8VXQZNfB3QxMmnmSE+SCjlU2pKtCdEzCCH4w3lU8xjCJ9J+NA/6fFMHQ79+y3FMBchFt3Mh5JKkRN0x7fsnDzajC/i+nOhOuIodWsr3kBtt2L6m7wE0yH7jj4Rlx8vN3fBpqKFyCcFXJkYE1pF9CEOKgOfNvpHp2kzZRuHdM2GIhrtBUIpDDqzHMsLZYqI9NawaexSBBvcg0ZmmLOnxeTK2Xm1t3U0VhYH+ILmXcpS9KG5+y+ugVf1YwEuXUngsXZSjTbPO2EKthCKu4VMKgUTqpf/TM7fUO6NDoOSoNYTCpBl7HGf3d5aHZwHYTkTWa8pjXK/8tgSaPmYlN2J1ihaDF2yPkwZ6MNOWY93m98QqTb82L7fnLKoOUwPcRDwB+TZzG1kMfnD8DMMDQbpgFxnHL31ywDg1a8oPhcbT36Y6uybMcR5jKr4EBxntOQaHYJ9Vz2yNCW7w9AZTD1MUXi3qJv7bUM4CC8XPrWgJUyUkRQj6EaiiK4lXTlXaBIlVijgRRwe1rG7x3zO5Tpa5e0glPEuGbAbW2H/Vs8Bq4i0XpEEFBNo3RtT3tNsoW5PnvV7VjKUWaIYL5zNwNG/9qTiX4qmMVx5SMTsEUbnOMDQl4GnjBNoOdnRVWVq2hRBJBcXB/j4HQoWW5smiMqjaXKMjyBT+nkSUGzfRo5HHXyM8tQ5LiuJTxYzX7RkBM9+fkCv7mD2zJSZyJDMM6Mbhqgy8cCw/g8YZbIupdcJqL5W3x5AwUmvuMg9WW8MaN6WIAg8nI0xhKsZFQqmETh+Qg4FBb6fxr6RYKybIhk3cmAOCqJABbyaODBqLXCUAG4M4J8FVaCI6l/fSJBOPsIqbt/6wAdkp9NuCH46WNxjhQ+pkRUqWObyNhoNS+s+x6m3qdTuFRDHGmHYLCcw6EExJHXVwTCQMl8VW3IEIpMJjNTz/Vy1avPgpW9pfSNdFkr/ztXQtwGuPkZtIfr/dHvsC9JJGDXd7XTPkLbK8Pf1iVPQrdgwGmUwo9fyp9a3K0upvsZONTwEKIQ26NfOpE4CRwsl5ZWxHtsi7sDDBlB5Q8vN5I4yDUJZ8jkWgwKYUOuxbnvOZYGnOa4PqbMmxI7T0TKq6xfGtPcx14s31K36QrPj58MNC6fxyzUF60hO5uJIsnLqzJrdB7wpAeUtLg0lsNeQqf0tyCFfhe+1Qw8ubyxRD1GjLRGnLNUjbhDyvMbFLk1DODXbMbc9hYWt6mQFiW9pJbiO6KYdDt5QI4Uxfx2FvV7m0ZxyajGPeEl1QHkbCQKQRfgpqD+rbm7N5RW0MD3Hmq4aETlBmRhCuC4jb1qwHKrAK3COm0F6P8NqCsuzf93AcWRCNFyFBhpIGUlQN/kdDK5Wk8T5XfwgFbw3+UJHWhfkEbBSpsH0XLHDl90UGlKyUJNkP5bt+DtLrnXoUjPUb54X3HaFOjkVg0YJlNu3GtHcF5oP7Ya3I0xCfAkBCmYVfdRNeZJQ7o8aVO7AJufpk9S8V7Nt5zjq8T2jYYLTL6q1Lp0ZzW7Dd+4Ykl1Us40OCfW1yp0+f0uC8a44aIYvpAA6IDf0z4LovNBDG56JCjQpqRmXXwCXFRcbkIRXvfKxsyq1XgpyH6XtCmHgA4twAqDc7mBvG6Vr3SUFzWpB44Bbdg2qU6fjNsb8DsoqzfxKPsXRv4Bq2z9vDCMN7/DeFrdwWpx6vnaCG90F5+vBUUDAiBIfmb+EqbRNeKD/EYxFfXk7cNJFbwIvxOjwUKDfpTF59m7zhO4EYvgxzLTIWeG8tdNRtFYtHDydZ/56/OPI9HQKu8H9G06zJzJsd2+Q9cDoXeP0qGT5j3yAlM8iGnbHfY7oeBWmDyb37tRXfhzbrjXcTJ9S57J2g7Crb2ewYvBV2IfGrhqV6vcVWnrs/WYmEPHQsbm/jb63PjlgCcHy1mgpGu2Wvi8qUHKQvD5dIF1Zb9zlKaTMkFYsUdzCAbqMr29PL6N5xmfEjL0xG9D6ip96bzMHrP0yJX1D3j4JE3JuyY8fFrD+7R9Yq04XQt5LjFxMqt7lP2g0PUw0r5JB63lDxwXLLLDApv1i9i+8mdV1RRMRyHwPxhC3KYhx9t6F4RR+ExKbP60HcvX3dP+r4tsYnKD+tBtxqu+KgUXTbTTsftv+ymrc8ZzGfnJpFXIpn9E4cXvPBvKFC3iAsbOWRDSBb2kjKZO6kmW7vOdZvfNTLHo3uFcbZK5FVcnDbFNc07VzHJhFTeyYkA3yMqm7W8jq0R+UL8Phlia9yAhn9lr7+lVHbJH+x7YQLgCxTLU5Vu8/WQvQKiF9+zRbPDGMTezLpNPY9rSGzX+T/yeJzkDJSmLlpjEQlCVS0P3Kgm/VNmuhPrnfzUX52RrH11cQT+nZ3XhXTvK1Vi3hVG4QIPDDEyi/gCcNy0yrR6JMNE8FDWsXBrxiGPUaT9I0v2eAyQ0ic/2gpsf+2eEGqgxY1xMzZ2H2YL6w+aZsyw3bwSPe73dPeN9MEq4FZ2v8vWnqmhDOTKtLDI7SlgSiyByzS9e06O5xvxg3pXQpgDOd3rYG1d3NcNhSSHvBs59p9sfGtsxMF4MwUivpoBOAvSTwp2TchNHfjYA0LwtjWyrLg0TKXXa0mSXKYJ0NDA9C5mAPD8g4qxbqJoebNuhmcpfkRzuZbqKLM7bYt4oC2S4d2IVBD8M1PcAp3L9dfM3tpxxK9I7MM46VOdCr2eUX8KjLpg2M3K7zhYO72WJvw4qhJpPSKLMOf2hFXdMjGGfwNeCNqZq7ykVOmtGX3a2TvuQpTaoHOIjM1c3qKg9kUo7jrYGEI6cEvfr6PlWSGvz4EcKdU6I2gM+QE2rN1PwDz4lDFvO6nJgM7ZLnPkjCLVp7pXK1yt3s+0XrQE5tUjtY2vMJoB97aRlOpaWUazQx3hp9BZyd5P/dAbVl1P2dH6/HlqJhfHbz1Y4lmCUnJULjjaN0jnWFl0x0c4ogUN+C86+LqVhjltDpBN9ErQ3oTBaQE/kBgFbiLCRNX8K3M4SKHo3AQCeQ/7mFsdz3K3ywLl0Fx1uMBoc8nNIh+8JJy1l8eZ8fk5hmV1bzOL7CI0oQ9I9z8Hfe8zINvK1yRhebWAkOlkFOPIGA1AwlH2C8y2ivz8yUbZWypajq3vF2qMGxJq5V4aWXexj5ugbv4ZcPaQaw36BKpkKixlK0h0KJlXUhAhkwyCSz6zSb+cxpkH8wnIbXmQp98CkEoC3dWb1qXgJdBsRd4DJsp4AiiJ4+r5PpUGpwTvDrs+LvB5wPMlf/g1u0LcMV4FCmwsK1lmTLlw6sU2B4s7JbGf6nc7D5jDDDHjCH2jhXz5fgPeQ0pAVbPe0tYYDXfc2zEoHKrqmKgkh4w66wYe3b+wGhYUr6vBdiEmX6ZnnRBf1fX26lgrpSnmJbxnK7EfUzRVdTe3Yhz4V2ZN9mftWEpkAhD7yrLW0yJIpq4r5Pk7ncHDjoKPXU1AZb8+6dHmpfXdoGgbb2ZDZ9jk6smP7z88FnE3rCVgjwNpnXo4dd2QSYTmBcfU8LTE7mZVkc1tr9NBzQfEqx5BEOGmDnN+eIDXfhJcqLtmzJXzaCTVQEOnt3GoPo7v4rI6a35MuP3k3+bnPVRU0oL+9W8hRpBm92xVLJIkgRhsTJOWcMvqXUIMcxOfAWa9KMN00tXRQgswPjBiEhVMpqhlvS6Aq7LaHbf9AIomGYGPdArKmaBtsjzcNEYL9O+QtyGLhqQt3M7DNVaQ4QOI4HRPQ0pS0qUr7k3fGN7I6EF0kQmEVsKW5k0UbnFyNuWJ3oJrRPgeKZ2Kw9I6b07eEwWb/2sXdOrlWQ7GcJL+gfGFhBpkeWtVaSPtovmJDKg4KqkLumPdRN5UUXL9qQk5Ovl69k1ytF6x3uTYjIfpA3Uwsc1EXvqtHDzQQvXpmSOY4JWq0DTFqAL7OZhtckdrQMEyaQu1NfLdFLDnPfH3T1bHuLSCwyAJtfm/PsZahQfD0m4LSq9pHtjuoRFQyAQmsJLPkp7wDOWBVLqEThwTT2ehmqvHEsTQ8Uh5sOHGk4vTaWafLPGcqMMHGb0Xl1vnkpkMn61qzEpwYszgjWO0snKnvbdqYKDLRzxgDODMGtrqmAyZLH6UHuOU7rqbnFGj7zq6nmYTAqxZnkffO8hlv7MkkyhhDiV1ow8xF7RPvd4ypKrb4h8hMlhf7Pfg8xD3GiMnAKNrV8Wk6aCI2MmI1hZlT0DG0Cdm8HOAI7egwVhGz2vACkX6HP1xCfyDBaAE+jCVe2WQHYjXgDnyQP4oLR1qElWpYcONvBJiVn6fHaSoM6MYzvdfWhoQkNoD0UsL3RD17m9AIyJcKCbnrTkS9x3pLZ4Rmr8ZuxIg3GoS5W1EaYgXPSVH/SW6dQqbTqjVfkzAsv6WejG3WFBhLIl3DJM4wC1+ilPhpgR5kodoR2Cd9qUyHMbw8BtqWKPrE4g/eNcgNE8T5gzRIrOoe5EaVCsuXNX8iuaUY+zDc0U5NoqgSfbY4AGzGH7K1U1uU7DxkFF5cYkvbGpH7d8eKbH01yr5ZbFnrpmnMd6TrR9ZCdu9ejqmIl6e2sM1sHfzaLN9sBmj7QwJbpd8zTsjXczOBN2ylZlOEzszYs8V5cM1SqO6RzNQkhGz5Oi5KbE/b7X0Aw7dr8j9PVhW3pyEWkJhmu7LbUnc6FWLWzBLmO7flPLG/xdSkNHL8i2A3Wr0ZO8gEvXhvgqjKmh7lTnRBEcq0dh2A3a9MwgE9J9/bfdB5fEEJ8VNyKgpFeBTpJg2tBJJtWwZhmjA3ElxoQqplNJfyaFDUmd3rffQS/vr0bXqq2SBZa4UdcY3OLYyIvh8RuQG5G4SKiiFVQHvzGeFWWhy0+Yo4U5uxNpH/H3dwtSLvXT8+IrXIUNVh+7B+FuT8iUJFDgCfKwyFPl8TwWt8YRlJLmbAwZLfSW0gOLTcmaUAxBqsZ4JvSw7Cna0V1u5g4/4KvIUp+D4KmE1FMCgMyYExMNQLZ4uAeNvmZPSNvlFPwMMV8H/ayQ1vwhGUXiAbg4ZtW3y7q9d3K4ChrxGI5wBMXbL/L2xkgWh2SgUEqkgc0dMgJBVFbZUb8+MmYvIbD1LRIeKbP2eu1SsumFXLmuSM17Cqk7oWPjsR4utvTnD3DRc+xeYxpoBCTMTmI6jZEH/yEBpzE5mi0KjgEt+9KoZiJcSQfG1CxvCJ0mt2JFN9tN1KWC1vGXd/LE2ISeQCNQEbb7Dk05uRCz3Wx/EMh5Nj5QLfJVbAd8IfoufTGOn7HUx0K69VhGoAQPLpdhNGsRVNHXOAVkxP8X62uf5zRNW5e0Bcv3oRzZhhO6RaRgaW5AdQzrAAk9Os3qLy6LNSV9oMIMrHdqxvkwGtmLKE5R5fRGC/5hCzOJrj3nV2c9uiRG1gJe8EkGlnKpC3Rte0b1p3PVmW02dXWmRmtDC2jPzyZ0k8n4V5RzOcSlSMI8Bt7bkn/N7nOBPXgRnOThMlZFhwc7PnyLg+HojCJrzRdMLeMq6pJvWeNbkFXf+RxN4Q8DUZGwRRK3I+6OaiBzjqUTJ3GAY7OVCSUtCqvxC6kkrFJoSyn2MeVsbGGHz8wE/DdZ2AaPQwg6TX8vAGTiTlNFUlMGkuaLGfOP1SWD24kmeYjm3mZGmtPQlUPl9Mbv23fhV0OJuLtv5Tc2SRxbnjl4R/4fQ+Vqvv/6RNXYvgGM8Wm3lTy9C0kn7Gs86TjyiKVOdD8TKLoTuqD7O1ofdfXl+m+vLnEQaw2cZX/+OWoBZQ0c5u4ahUdcjh0CvxhIx2ArPXtyxHC5Q8T9nmvc4Wf/A2nvTvJsw4MyrBqSxgIlKOOKlc+nIvk6uTRWQ6Dh8Dvl1PJaxWuK4Ra7y+eVXexZJsFVvMpoMYgiEVx6hrAUMjoMXv1Xfu6Pj0vv2Uun9jibArIjE2dxKDL0AxGBa8WzNQaaqAbJWJwHGSSn2tUqjSsy/pAnMILW6QEbQWdXWXd6LIqKPMAeEZ6Jxgk4LbQrSV3UgmwO+0xKRtq5c5njEJ5r/PY/XTKYJwlPhXcCQWGEqeq0v0mpPbHGAWbdLKSY0zd2nwgyRHCLC0b4UxPsSCVCzkvCxsQXFrPHBlaR7ocrThnMuRTsZMCcq3AeE1R1pW6jC4VpAQWdxE7IHIRaQR5KuQCdQvCaUEdfkrThHvpaTIVADMp9MpHswwNaTzeL7OKf6N9xU1lC/BJT+CjdYMW4HdmrlE/KQvSB7tpXZWGEKjtRoA2+AJ6OUcZWtYtgPd8C9bBp6F+Sr7QvH89jUbsTdjfc5Qq0udj5Ydfsd0ORD0iP3vVCsdJ5BN4sVy9IP/Xyhu0vGvU6uIn0e2XaHdwxrxt3BFWmuwP1IDe3SP+BzFKqWCxq6DRvoBqfhXr/s3GeX+ZCqvhfWPX3Wpx7/6sK2avMKt2wm6BZpJGO2xJjl3xrsgRE9tLjMaIzZtxmOxm3aqKDnTt7W3ETPvwsa6b9PEwsE9zw15t+Oq3kXdjbWrHTXFd6uyAgvK6P6r8d4tY+KYoTNTD9eokuaYdo3TJjPJhxd4BGeBYZ0xA98K4B3RgC2lnpPzsAyRgcT1dhqB8d2m3fmVCvPxJpyqBR7pz6n7cO0hiAjCqL4bl8QLvI0ObLm3XsLvOXJY0lg2GTDzy3hiQ/ehfUxsNqUoOk2U7yFmvDvaaLqqqaAdoG3vnPeCK+u0MzACHnlIqKM0A0GcM0L2QZLIt8FUgwl0y8EfOpPjw5yABaJ1dr6ySQ2pebZDiJEs0qWqXdwjzD4ar0KFbIgyRpVTI1SDDUHWWCyTz0K7vo+Eb1aTGGPkv7PkiL6J4vLB0zj8ypb6tR7jwlqb3AQpxXm6HKOMITMhgUIMg/QWA82C0R8q6FnFq2Xm/xhH1YMtoHxRn+0OHVlM7EE37FngYj/NjgmOHRA6ZtDEPBjLoSeITqbq7eqlOpX/7GJNVnmU1Fvv/VGR3EDK3Kn3fyBqgIHBb/yHlUTeXXZk11p0Ou1ETOGTF+PXlPRMK2s7kYu/QT6JHDxzy5dJTyRYjEMd8y12hWQHc6u+vzVTl0KB6ILXGjohqV+9V3nCL9S6JpvINRDoF19VDDlKwP4ZbLRVRYf+PnXoEnpLKAHvVCoC/qcbKPIUkMKlHYd10kDcFoDt/v0Ey0jXk9tPlcXUU0vM/kqaIoyzvoEWlMqWNHC6LCgIDjkLIS2cLB98jlzBpv6D4RcrK0+cwFdNpvqTIBVqJ5lJzGfyp6a0UTbUSNvl4s89ROhVfaGjHjPQEvOpI8lYfNYEIR1M76yPfx1x8Sf7FHj2dmmwRsl4KQNclLc4gN8z+Bb6D02DKEF5e8HB42P8UDmIy8uXSyX24pS48Dw7mKkAzMdbrb+D5EDiLTRINnZMCvHA0ZZbL/zMgjiAmx/IsMCR0FgmbEP97X7tNvyNpuJjSnGXzbRkDKvrLyvybx8dl8zDTxFLfjF7qxeSyMs7uUm6CQSQ0zRd7L4iDw9Hhxo/QQ8OMEmGVtBOYmv2UrH+Y8rS8L9xR52HlJYXfOaOx2hs3fX1R6sI8TskvEk+TjL9CFW6CI7lw8bJm7ug9SaK1NDH0ZzT7oGq8ZJyF45t3Jl7k/vC57Xo5eFSN7s4g8ezdR+uQF7fNrYIvgI3YfkvCoUsMsD2oYGhvyxqDHlZ0W0I/F2slQEpaW+LKbX9w6P3WfxBcAxYlnB8NNTWJXwbrOZMGNPG85ZSPtVUe3ibKS9ReiUpjfxiPCoaL7F6Uaj3JVjtxuE2Rrh6VAXA4gzzcP5kxl3ZnsYJa7zNd6FCR2B/WNAqe+Lkg9LuPm0Ny2ggjz5YEyH7vwmVzw+iFtvv1l9lNXfqJhw7dBTtO2XxfKqpHSdRxeDVRqMJiSuO3NfGL+oqPqEb5TN5x3ISIaP8kYhHAF+2aaiVXkXX5UZ1LMWpXHw7tTazpmEfkaNtHxA1d2e/KtsUOMpPt3T2OR0M9HhT1yKNbEJ4vOaj5U7GNgHI1sLqw9MF5NrsODJDSHn5Y+ea/PdsOQD9Y6Uqq4tRdJ2MJiYxbFxMW33JnPTxMdD4mxjP7q3M7CKK4iQPl9nkcRZrxlpbfwhCiNaWaQH+YoAeV5u6hzLOAG/1l0b3Wfd1ObS+a4WfAa3NJK3Q4KYH3A/NnCH4YkOIVmNrTJ5MbOcwJBDsS7W6f9FK1E7sMpBqLfnAycIbNqKa6OMCgEGcVDEEt9hXjdNPZR4kU8q0f2H6TyNnBbjL1JQbNP8g9W3/C8e5NMHwyLeyt8nv42Qk5Ptv3vsOo1M3v8K+CObq2vbIp8nFWPS3fhQe99F9tcsT31qDdl8FBGxuL16uKnbXjeNIBAKYYZ7yzm+sfs6U6OY5yITTwEMHiwLLUDHFtb6rEebkIMRu3UK2gmO0R4nNcgK8c18P9mXjgDIavNJuL4KBD42Yj3tfS2KmWbxQ6kW5/0BcVFFm6HO2fY6ikTqwXSvIUe2YSVy5jgLvp1y0Eu+VYcf63P6l31o0gHzbRoajuuvwnSPNqlSU1vINMjDuapmnIVcOJGTh7Cl0uMtu7DyhjozhBAJ0nu4/H6mbYnEDTQ3ltX7MeUtrFQhTeZR8rNoJNbvcmlzbaVJhD6K0+X+f92w/AOO8z08UK0h2slpnd1jzMpbfPsm8VzupH7E8jC5CQqcjr+1QJmBSnlzLH0iSS0cAqr36xgpYXOuM0R6WvH29KA1J8q6V/yNcnWCgWyRm6ktaHnJ5hrcQ2XtEyDIEPFPhnfMwX7THeGu7hk5VqPGUaB1aX9d1BnuT8bEEvlEUThhIWGn/TvD6ZBfCbGTPXK6jsgfGmM2XvrfXsM+ZUbGMcmBCYyaBRZ3SrN1MMRA5sK9jefLsDfu4CkJz2VWI9X8SvuV9I9NeFTWH5M7xrnrB/2iAbzyj9JRzgIgSUHSa6yezQDB3+zR+sgPNTvP2ZaNl7DuBFMmyEI2KFUHYsmTSbzuVp/a2CCA7I/9Hr0i6k/Ng2INUjWNRWjb8qoU/lfUXAXd4BL44xNKwW4GV7EBrJEYnRHVmsVeZwmBEVV5KMPkv4AJQqvzE6XfRVpmS4ApA7ZBlY3d11f8FQo72eoJHfqi1LeIkx7G4ekxMrQFHZW0BaXrq1OJY99W/OgAqxKmz5HGSrNYzc3nLGB/ZU8uk0d5emvcYJGZy9W5hxTJZ9NLoMiNHw9Pe8M1VnTQzUjNJp+3KCyrGSRIQiVkAgVwbSn+Yf5jP7nkmU9HQ58UhL+1Kkx1XaGIbXiySqj9GxHeQcLTuWsnW2Mc8jTeHEFIKBL0YRh8RS7vhQWA90C/CGBJdtkGtRPhtfAFMGMrmEm6jyUHE+HB8jzHW3d7u2fx1tPhUbCwr0NJMp3TfPRZb0G/k9ZrptcBq8n8gYIbPHWk/Q8dz0HrNqDf3pEz9Hr2dCq7NAZHkL5Q+AVGjXa/LakXj2KSL1VxePCbUpqHnXTjMeRoMZzw/mNbpIN6ZmAOFB9cEgZGAq0I+jjMfAa9B+8BhJzsMgzRKT7oLmD6ObMi26I8sjy3KbOcbsFf/UPAtKrRRXp0jkmS6gfg7EARpvX28SsiDIV5j/G4/fJRNETFkkLhpJAhTyxm8Ht1u/4PMnBTYYeaMJrlUxJRjwbDNx+0e07GYqqZpcf25Qmz7PnnVg1XoWA2mFVGNbpW5BwHOEg0QByqbGqQvn8ACQl/cEmKCR/FqOT9sEtcZEPvQ9ICaV6D8qBfGD28W3gOxMFO7ItxR5CUsxgCv8dFjCKKOqkGXYNa/vCHdZvrFyuXbsXQH9A1aJuYDktbxoI8ImXJp5j5qUqVH5pPDa6A3XpmJCMoe+SgupNGoxzu8KYx/AiHW4AdMsWOR3ZLvj+8tyfQgmdFXx6SsHtiUH6zBZBeK1c9HdN0b4aRfbxMeWgr3I5jhZs4jZSe16Vs11gZzMsDsIxmH6XzubsOAsHsRKpdtMIA82tqSF9oWVz1QchoJtkaEp6KvQQXptPd5HCkrO1z2dNK1qQQYjn2kRNw6GFSh8IeyLcrwd8mENFxsV8PWKqn4Ps8E8WnBzJJv5HnfXrNvg3c+fF5Jo7WW8efnMrgZLHQp8tNPLw6yWOAe7Knqx/LuVKu8IUCdmoAQMh95Bq4fJF5lYbIqK56ZKHZfPuqx9qVw6RC7XFcYOzLq/M6Pj6hr4YPl0Zp7lQ7nDkEDwKOQaNXiVvCqZrhlqAm59Mq9Jy7L6ub5VDP9EazbYHkkFKf6SWQ3P6oMvkh1p7F6BbWRdu09wvzv7vdYiCxvHSjy3dR8jnjNxyVHaCSTQUdUQq3A+TfcJk/bdKGMLBhr4GjafX8lD0OWwx/6QqHc3aADXHFLuDMPr7xCwZhVsmbwmDqV5BcBNbOR3C7JgKCKzEMTNDvMu7jWAR8NGvn/vL+3iNLhd0NEz6o9Yxm0hDBLfd2CeUp59rNQDdnmqWMD1K88PcE9JqPlP5YvpQWfjC9m4c4/5hpPzQOUdtfrsPWtJD5XPH3lCxuucuo4CLOOB6FYHTCMhjAhBMLW8KqUnmt2PtkHnNOMPhl9eMvbmcwRu5x6UaM8VSq6G2A/UjtIGl9Ud8yaFxFiW9ij6CPqP2+6Yt9Nwvwst4CSJfLEbM7d1kYRB4DCqYPmCMjCPIw3vsD4b7/6KMuGwCQ8OakxqyZVL3p0ZLRAR0U3kgsX3Pd3y3a0aXV7S3/WgqRAEXKqbJgsSmdu4cGrzHmAgIRzeDHfwCqgdIUaW8C0ydCWbmjs5ZeG9B5LpzDiQBf0u8V4JBEtFfzSYHG8Q9u4OjqFE2q6jKHK0w9cU0p4u3qUQ/U9sJPzldB1nrmlXyuJ5oCyiqGcC0FqhR10nY5cJY8QRHiTHT3gyZjpd4LbQmuEEDwz5aZrSzr/rAtjnEuR3YvC1vD7LKgL+bwEFL9WKAFV7hNkaOcfgxZQQ7FrrWk1iAvyuEqzscCpwi2x8Qo7fSLJXxvsS8b9tbZhpMOJWWaAC5Lq6Jbwtf6o3S1xhZnSeTnYj8uLL7fqukwHCbq2Ly+XbSvnsMXC6ngBvPDLyAdtfyjYVxb7Mr3cVUn4Hy0mV5S0MP4U/yaiYoBnKbDQ2xTaPqDcTvhAuebUEbtdhydXJV98Rx3Cjv6wemLwBc7w5XBh4MPKgfHGq8eHUbgtfWS3N5e1hWtAtEUD2ozO3DZ2jCB0rT4TDGoz6eQPNTRD+iMDQJN8RzjmQwA2aw3URQhL6S1u2R1xuZV7ajXDBRC4Lep6M3Z/YXW4O1TvpNHw/7tkXzzLB+Sq6Yz4MnJaJRZX2Lr+cvKfa4qkSzuORrrVpbALK9Hd9pvqaSbCyBtxi+UTtZqrrCX/KbrcPLApmEGNq9VGb3hScvwRIqtt5qMZpX7yZE0bEAGXvVgBOKWWLudWKN5sLEa0AsN8a95+3Al30fLNT5eSHKZiJHY3O3P48VGVnH7Z+vUMNCswIyMI8aSNhIGGtmiBo7eMt2dDY7pfnrXrzSV7C8BHrw6k5CUAZM3dDpSPXcjxExpfJW2U0rOFyoc7TE3bA0+AoarMrSIuwA/YxKhP8HvYB0fVMQL+EHmq8WHE8g+A8v1T9L0v0Te1JzpukSVCWFZfZeC4YfSnNexujK5Dlk9GWRwjsc/1DaL/yN9i+rRJ3g3jIdBGOHVZBQx3jFcxqqwMNvc3lGgUKTCTuvXJHfo1pLDOlRJJdzgVk9ScH8ODbTOIVDCOjZdwzLCiavpfFo05o1SeRhbbsh+9HDzjx8zYSr3rXnQd59wQ4KDR14o8MmSeaPu8xFVh/wjExYmf3kKh0d9MILyUGd9eJGuKyAR2Ixh8Q9RX1ej5Xiqu1zw6bU4uHYcc5MG9Ls60ZjkRGBLTbMuvA/LbubKc0G2GrKDC3B5qvT3QwslKJwLIWOgu95GPdZ8MfSUZbY2f7CnbaDunN2B7yd+v8CUuCaCYqH7bwDNEvT+pEBVVkvpkAHGUvxPsLsEv8X3oL0aAiyBG/ds5NSJ1zGhUfxEVwzmzjMCoqRnEOU19nXnxIljXsPEEqsyZHM9mxuybDPDNj5BtYgKcw4ESWHbEwqQA3bQqsTeOJsPvfVbe4ge6m8KFqGpXHVyVBgrKqZ1r5JM1Y9xv1Wwl6LpEqXbL9/n9g9+fWGg2aU3NnQc3pLxRMC1Obr2B1Cu66Oe9c2LYyeWfqfKuNjXxKz4BPa4j2RzCS0aq4nNHLaQJSPTKJsyUn7CiTEBeR/HrgOjNnEVZH9Iyz3DROfDyftLOMwr3s4Pt0SXTxkn1fLBBIdTUqL6SBf2FEyzNwVAXY+ZPvTkjaLp0Y0t3hPcw6PUHNB7YohrSe96HfcydLyFgbJDLPS/SCwEMBRXqfxodlZzgYRtHA+IQ7XkBaLz/zeVcjaEpX6cmTvzH0C/hzVBBE/fXaAA7+RgfQ8CMrKOCDc/CDfMCMbHHu93ffwAPLPvSGhIvN5m2xH4LxqPNCCeqAMT0s19ATIqiwP/LL2O5zoFv3/WQLGy1qPu4zWrrtzm3Ql6XEzCxZPZzBIbBKVJDdq7F7fC2D+QOTj2YfgBqM+d9+sD5NYI0Cw/OlGwOrX8ZkD6GldspZjpkz/qHnlLLzCwtxaycgyNBxv3uO7IJxPHsaO46ihm32AHyrmSwYMYLmNoxzu4PU8iMEV6UXDgvZjY2AjhOzbhgZ7tvMRPo1HRWQdIEhkCHbTOrM+40UM1F88v8lJkG+Rnp2FD9tBwbF5UtiuzSAakPzCE2lHzKq1jo73nvKQJ8R6hNyiaPwNhvi8aNbK1S3uxWsPH73d1Lm0XyZ5YPf+BG8SeMNBwAt/c9LHoxXE58ha2nHh/jEb5YxQ2XsWrxfCBz0wFqLH+RuMcIxwG9HpGPHyIAkyzcRYqSZdf/iyCknN2MnEkU9LWJ8wvcJ9s1dUaPV+U1PlwQzY9zpDT9rW8bS4uEHutb1bbVQdEEDRHCRCvjmvwdhf7NNB8oGdOrjKmRyp0rcMrSd9+jZ2gEbzcSJb2CNydeGl4rZmcfqGBcmP8sbnW2eqkHT3LUkINZkj4u6+3GpNQgZfe2ll8tvg1jRPwsG5iCnS/qUfcmfqMJozr2IeBxZjB8/PeU7X5yWXZ8bkAM8PbpNqvVxX8KyfN8VoXYwFHU9gD6Jmu5AyT5/k6q8Z3oP7Cnh6M7ANRyw6dtz64GBgBeOzY19qHTAYfJJVZ4qNyRsvy/qnkm1K7CNnfOSc2EUNs8MIbmFfbEcv30Oz6HqtqnbhvG0lNyvmshHV2D3M0k9/xzdOCsBcw0UeMNDtwvRmpcLxgZsvw/GH8sx3p6VIUjILIAvUL2mU+AswyDL3QHdZ9+BoSG6dCaTlWTlFr/bvGz1svlHQAXVb5Wd6/6nAFsC7Mz1Vnsux2E0jjBeWiGzWq0W5NsjQ0lnsubwbl8nNgdp1PaYaLIPXGEJctc/yMChVVp76ry2ccVsX0xyqxA0Bj0O6xvXlxr4Gyp0d4Wqx3gCaPdbnlKBgUY8OeLJ0qHF4OoAn6hSgpu6EJUMgZLgQm8LSjEbDI+Ek4RVe16GInGtJxm/HpBzTGSbRAYN3N3QapCGsRPtefMeQQJ8d8EiOm3ldm9Nqc+fo1+NVwk7G+Yi0IaoKZG/vI9H1bFOp4EfX5AGT1I2skb3oDwBPOEvYbjLWWbvAAKJjrHIYH0lV5EbSWdNiUk5K+kT9O9g4kHYeP8oWTaN6Vw3E3LHoeXI8hqN0KJtIokPGtBIkCvlvTewl7Ap7q+bu2sHQD7AWnZAxmSbbf8AHswFnAW/kJq8ZnX+Kp7xTNfnhWZnaZ4B2ooZMkEsFmcfrrYhsMqfn1BQzNiPkTiiVDJgDQjpspjw9VYnESMd6T9nCFhAkyiC8iUHjx6XfRJZ1GMtBMR/qfwPlVPn1pl4dq3ZJe2lVfldUU2vMlNZyN9sdLPq+FKNC3qFP4O0IJt/0oBgvSErYgsSgV0vVPV11Y5r7sFXIbTZGVT9B4YLmLffqYe6hxZ7fuYnPOwYRLvkCzcXoyj7GbmlMiaXv2gJGHiJHx9T1kOIFq4BFFXnCqQzZOrZEfnCr7nx7dLkXUvYlbKxunAl9+wFbbzExvKsKe3sIYOHzs4JGtBeLCpt7pEiPgKLrk1a1nOPlVkGwwOlEQ9ugvo0O7VmmSoMIPHl6Mh7eIA2PdQFYPsnHn6JA7Y3yQXWYtx9g5o+zCodqOxO3Ig08Gpmwtzh4JQBKGPlTnS4J7Zb4jWP6d0vN9vaNYXbVE4HEzA8oYHDKp+wRBcs4xA08K9vtQwOZTZ5SFxObn4YcDN8jnbP4jgNhXmOeBZzYOn11+jKQc8RsdeFPPENhqYeyBTBBmP7xk2k5Z0GUSHfQDBRmZvfH1IlQ0xBDMQVR77u4CQEUTLTPSedd4Q0pWJt3DeL92u6bUv6B5eI1zvZK1ac7P3tMLjK9+x/ZZXAF1sZcpt9BS17Pq78c7+c29bP6Z5jG2vq3J79WbMhtPsvNzKvV8LBO5ThLOjjLg3MV/0P6JMp0l3CH09Ci58ywu62kDFjmfAzW2o15YkgNS8y0KQMwVzu6O2bk0IPQZFkXaOPn5WYTk3CJO63+P6m62sdbKqPZSC3Zx2oH7L+3MJGjWh5YrqKnvQJQzHoJVVzX3HXl+NbBZs6YoCQv0EYgDdA6YuR+dOtlLdDxnGuibhE7m0Vri7azPYvYg0nm5S7v3CuOb11LXftjwr0RlLlMNvDMFmvFmajBcf0JSZe9yzpbtNqiDQNz5NK10JXvX5Me5V3UH2Ol1DKV1bRUrGW1w0hXBvr0XQ4UHPUcPoHU1K38IRB6vjaDNLlIK6vZTO1y0Bw0XdxOAcKCw6S2mHdkP5y3YZQyNZBQDKNjZUqLVZVwoUe0rpNZwGfYS2T3IV6cEYV8WY5CTJ5RD0TYq8p6RhF9JU9ro0JUsPDu6CUPVbbnExjHCXSzmIjfZG6UrRjYIYE8XiTqnxS8WRv7TjVP4htvo0tpCHrnQPdgzyw8R5R8gHWBUMt+iuc4ln4J91GkMQRbZz7htRa9NTlQ9HAqGK3Ecn0ZF1bpf8zk5gLSU6T6oHGSNmBoeQDvMDQT2UJwm6GbLq9VVE8N0ZsL7whyPYB42D0gyCOTVnxZSyVFvtX40hSGFx5mCtvvZAlBEkTFgbbBiVXmAeTBzUNg/vdqWU6aGPwMthF+HLwMKEOvOSSH12bXWIkoRASzhbaTsoGbQ9SrLGuO19hg9SB2rVNeWQh0x2Y5ngb3RYz21YVIz0E/wLXfq1s+W7pXOVYXeeWEKH2qQjLDMs2F1JoIE8YmXxvT4B4ZrBhQrLQtH8fP3xUAFEnHqrWAGF6NHcK+AxLh7+9KXNC0CjBOEPsVsXTI9jM2/S6SbuX0IOvxsSNnZWhPy+Z61/zRImAw7GNy/rAJyZ1RTPsOnTRzsQ5Z1E7F4nw8wc4EZnRtLvTy9JR+MHoVAQ7uuRdG3N3L2BMYjBU3HnUM+wahLNaAGbrgbbbtHvwS18vP5F80ScPm2KfBWGzN2hUDjvBtWfIgBbh4X8qH2rNzl4z4g+AYTF36zVw1+p0SqLITBGX2xl1YqdoaNjHNsGZhtOA8n4eQhRlpD7bGUKDb1Y1DJXCNO5SjlF5OJb5Gfyhv8k5vCU2YDbGsIi+eCAmx3lgxN13YA1W7hy8jNCkzqoiYGsj4h8/lSHPuBcCR4N21Xpa8nVKr5mCMGO0b/nkS5fGAAEZlbbIUjtqMsVjJXWwXQ3XV1u8xLOBEx/5fVD6cEMsR6mhLLgq/1XtEIUQGq7Fd6Y06KWdVCGtmzB+V2oMKYrgZ6qSl7V18vmL1Q8/NMqFA0dI7AucCAgA1rtDfEJpDtHdrk29Q4NXoQUcrdSV9J6TC5fJW9W7mjufhXr8FRd15Lh+rFO/3Co4I0uN9ZlCWqJbgwWBsYAr73z9SgKCUbuk6R0RuMzRZEhnqrFbhBpjGSsC9eT4gO0OFeCBCJzUgY31Je3XZ9XezpkAHsfTkOtSN/Xc0Qso2gohxMbP1S1BlRqFDUmb+A11P2hzs+CxQ41cH9lGzIe+6O9qAJqBZP8RZmfPJ76EPq9LJMW9VGoTm36DrxLjMMRoTR8Xr/ZeYqz7AMbwHa/gIMpl/f0wM0iH6nz4Ua/wl9PrjLXeaxr/QpnLkHHzETuB1r1iGxSRw7L2oFdlr8mE3jpj2KkK7yJZJIf6G/mkjzgXZrYbcGhNJkNhFlN3qeGrkhojlKe5cOOKZWbJ5XuZ3iJxG9hRHfhZhmcrL0Dr4tCm4WFJzdQkvqdO2ixTfnuV1pbRx5rJQf2qlApC5Gra5ntyGyXBFuWmgyxCKdG3KspGlObsOh0HYhNxWLOkVCmsfmlKT7S+jD00vOwuV1bvMXqoVD8m0HqbV0Cs9hcbUBTC9q2mYMG/2j8zhEUPIKgVqZ7vnwhm8wBW9rbbf5jHKnjW1QvBVuyKWcgCqiCjBxNeJCYnqx4dKxr2jVfbJJ6WnCQEF1q1tcn7f9GkBjAH+sR5qfO64K2bqMPf3HVn5HdGI5yKV5zy1q1XBwrJBmu7WR4Hf1aaDzvdCtCHgzaNV+V3w3wvSC3Fb8tA1hKQpUmvGlfPR5vVKF1gPL5Mt0FZtVXdS9hVODgA8EZWsgUu7Ol0aVbxa4dMy4n1+m4MKNaNe9wN9AOlzLcaVfSo0Y5S4T9d1mMfRyQx+bjnm8OvjP3fWhcOTIK+vYEhKNGEeVrRVAqR8OBUfmjhrdPgHZIJS+Ut0r0YwMeN2tGHWPO3XwsNKZMnY8kf9/V8oQT0FdRJaGj9n4Gnqb6rYiSrBRB8u5Vb/KGGsVCkeZb8VkI2KSpgkqkGXrWo8GUM8bqxhIhrsAKiyUaSg9j+eCUe0KCT1wnlQFBOZnQzwuD8ELqIiL3UvL/X4Z5k3Az2XqI0BT5r1kQyOs1o0q8hJLBqpvqtNtWDlvIFBuJ6qeF2mfw3CGmGx2W/bBrJmMkK7NO6C69wM4pNY9LoLCWwaUD3ZXbuUhL3AoUvfGMfU7wKvl6A9k/Nfwa5ofwP1sFZG1YYmJEvhBa3vuy5KTa6CVZvPz5H6JW/FVCmxBRhbLA4zxhDsZsyLVAM0D9AGSb1LtP3LAR2K5l5i8+dDfmU3bmwmNKH14fVxEw5ry3rBqsbuAgJ3H3F9wa7VNwJN0pQkC7pxjwl5ObZgUi7NxP6nfVQTPqP4O1oH6iCOVDoqzgXzGJ/m3D4dsjVy1VNg04m4uOkmSrP4pfmwvXrX06nyqLd3E7uPXNUKVo0zf3C8H6cdqjwHM5jFAtY1WNlZqD7WKMzRs64VBx0cuWbR6eq5AB+/8L9aBLqxudHlaoHNN0S0zt+s9tE38iMAbmKnnXWCxG4Fx8DkXZ9m4EBahXVR3avkj2hCugxoewYkgIX2WJrnIl72ir3BdLCxxqsXEY6l8fXiW+zZY0Ssn1H1XQ5cfDQI5nKOw7uzsnv0DMlXaH/PdgH6wD1dWtrC6w6o8gVAwE9Rl0iHsiLDfHPvXUkM+xKHUn9L5BcKBVeclNK3bIFZHO1aG5AZF5MIRPkRo8J5RBnR1x7K2yQKxkGLjOHu/SiZMaUFd/Ss2+k1lUGd7VTzcmJkAmKpfn3dDXjKNtDEYL5wpiBtrHc81PVnO01ZQ0dTIJlmytnTLCNAAeGTvzLLbkMK2Es/ZHkSO3FfsCuGVi1vuxcWdCJkI7OsMbqSJGuO3bWvtlOmFlTuk4R3M0csvrNPwbAiNM7jqNc2ra8dGPwsaD05Y63eQMCg7LGCIz1oaY4wS4sryKLS1uZi5F5mKCGaxGG7F8Bf6LxYSIhbWxE4JtzJ7jOqJP3bx0QqlULhlJ0tcFV3B1Mp3x3gLbNWz1ajEmcbJRFd5gpVUQX9D0rm0tzHFtYCwjJnpHGtP4ed7iJE6kEGh8OAQZ1pZJbMs9RklNQIpkd23Hj0t98o2O4BFRc1a2gxkl5L6EQRmAm4KyK3LYNSRuGB9MWoz8YZ7hv9BMqFY30NjOEAbD8kNCjwgq4IMMTL2dcd4qbSJh+EQZchtFLXQnGh9lDzIlK+n3OHIyCVKBWlKbVcCuQpHr2vl3eEXYrKzNj3a0OJyjH9uMfBYH3MOVX7cf8rE/Vw2r8ZHDcfWbBJ4lFFvPSE7X3cdbEI4YZ7yUXVVtN0nz7rf1GVa1nNNGMzNbI8G7Ta6LAjB9iFqQH5Y56n0Nin2jAYj8Ow+7rhS0+C4Y9+9bBVzC/Mk3o2IbRsMfAx0cZCbsXu7g2ogwCc3ooHXAr5XrHqYq1bJWhE6HdmNlGvAQ1Ts2c4Ea4IDZXyG0JD0qIh4hNPDxtmHtFHTVRmKDqP1xLaJ4SD8zXbVtyVYgjfnuZxKJUCHa2BtXKDmQ7tHxfnfcr0D7bF7iSz2Tr6aJC38pTfLz/PZL8DgSfxAjd3Pb5pGoUPy2sGaD6pfzJQ8C5XNDDYVc2OwzXMCWEsXzBeJF43E0qUv0tf7k/gAYlNxH9ZKhTwwJJyBnfUCPmcPJYh+a7DfqzVI4qXwOEmwJIiCUl/ZS0YCn0bH02Ffd4IXGSJT14GQHT56XZZxGT/YLk53grkUMY0pzpVPR45CbPbHGLlGnCxwS5kvrqk1kIQ8v0YoOm4pVGQgoQLcYdmmM7W5p3PLxTjMRaGjWrnKQOQZgGvENgLxFyHM6e0jF3kbBUuVQOuS23Y8ttoJt/yjaFWdO5a9qyfWRPQXDJqBvpA5nnxgAkyrDDXaxqlqGWF2qbfibvjMMLK8eb+xNL5sXGsCnmiV1Y9MTFBfQRzjzMQmS7bM2XEudXkS4m7YvaA63HSiobvnXHHIMBnXCFm01zlR+OY3SNDOA+x+vNONYyDVnJ7B9jh94FeYzU+2swcZX80tCPA8/iLZKEu5HG6wtMj4Xs4G2aZTGyjjtfcwpKF9p6ZYWE4YKRPyvDfFmkdp8ZfWhm5WS/pb8u0gOaUG+kYo9VL0dLbs0KrTyP6CnVDh9v8RbSIikPuHYzrUfSf2JuWRNTAJC0gdu70dUukcvqiEVIiN/bRpr8q+M3nw6gLaPzpn3J9aQcAfPPY9sgtdo76+oMXzFQ6ZvCb1NF8YIuTJVl4lIKdXIQGTO8QkJz3QmNrPDqJMp7KrvT/QC8YrOYiQYzmk3cGT4SBddJo8YHuVCFIV0I/bDpkEl61XSb6o3N9zmSHdtBK/F8zdSXdAb7pnfG3ByU1Z6KcNeoFVQw0oELKVHJpcfWthdQ6ES5M6mEMyyxKY3YnRh0tNzmGFoiEc5D1vjyANBjeRRjCk1+ArhDDnQKf6Z4b4LKkn5M0bzDnEPqWX4L6frOjMJ8qWOemtE1DgSB9WE8NBRd7O6wSXCxy8vYlffIevYsIJTjgR5sbXt+4ZQxiwYPJyuIKDNmIq0xq7mqovJOeemDVCn+fgKmTZ0FQakl7PnEqxMHTvE1Jc5aj6e+JMuWrgbjnU1YQN9k3+7HzGuFEMhB3nsQ+bK7C10DoJEzRlsKc+wOF1FHUKpmqOvAFG+jvnwbZgROvQE7H7kzXlKH23UdoP0c02J6CWBr7nUR8i6Ia0lrLqK9uOUZ0+gEUV40ZcaQs8DjD8crG3uU9hYmTxUkHvSTYtGfqimL0ZsDg0ADodpjjjDhmIZhhb5u2YFgyCYv0XGDUvSbf0KyWKBzhQHCtuyXwsb25ktVKeQvWyaJAgMdnavuhJXx7fASc5mwDNavCtqoNVh8PQeyMPl8eDmT09uUxkfX3b4u8V2M1NKY6lwdS7l6zmUw9BzDQUcuRl3W5bpc/DS6FzhYgCaqUqRvUdp92MsqtaaD61xN6KYhwrxz2JMtR7eotGjJ+aiVm4Xiob8BOCKBdH5rmGXv1IJGJ79hJCTAl4hdl+d94/GQ+PH1Pj0T7bFdjTqtV9pVJQBPJYCSpv5jYbWLXSLb0rPkdXBrj7wcUw1zhYExahmj4ETPcnhbr0vUS8U1Ht4J0BgAZ6VoyZCsszb7xKDgwHlEd7mz0kvUeQ9NhzeKPdVb7b8Fv0Uk2nQoRFGPvfqOd7bmfZDVHCkx36w12yhS8qNMPt2cKiAMEAsx3BEuxtVmTQ9x4Cv3MWAcJBn6oFxjxV7fT8RxeI1YImWzzFHN7gCNHzKGmdkx5iXVwc7RqiaZNb6MHRsMK9kfyT2gBfHKdCDQ8PKyEkMzxr2ApRdKKPEm3gS7SnxTbjXpBpxdBTDOnKWpjZ1sKhkpNUbqYnwXIPGGcMQMu/q3J7PvUeIUTTQFJmjAWNMVGe52jEEE2pHpTg2/2+YyXLKIJl5OBWwnr9ykB9GyeZ0uP08+kRA+yMqsgEtb9K1xOK4lTFDXXgHZqJjFXYWcjqIGN3bxrmwMNKG4xRv7LSJNq3t5lt7kjrB+kL3jBsHXzSTw3eG/bhXWOdpJE1pHl0w4oBK/zjGSNdgxqrTnbY7uFjbd2oFx/8QvAspSsyH6qyBxvPIz3gJzw2ZDd4Z1qrNrESeVxGvaqqXn25w3z2JyGkt0h0NO3ENlYDlYEDZ5DBMpfDxjRwlW7nLFiNQ6DoJmiTuZYvkVGTiA2aFP2/l9I4ZZa0jJSuRtpY0ZgzLrE6pmh0KpqxjX8h4Ah0SdU8cO2DXvPOvkGkA3e1HtAapcwrRddOwojsWq5WyoDuatMmrbHRNsbS6VFrWnTZjnb4gR4n3eVVtXhkfHBodaSd1qA/eSaQGVufPkxmczGGeVNLjjPtJHDyRrmHYyN3D4X1RHNPAcXHQ6BNn5pWAAqhTE7f7GOxO9LTzHsltRXhD8Ui2n1JRWOabLhr/nuZWbHnUT9Zu1x/gqfmZfWpHIDyqWG1wT4pFfRQDdnHGV+I0fS7pPsWMOtFlQUU2iFmU7rh6gGNq31trQs5s3mCS/hSOfXG31QdZuU23xwcJKWB5bdlqfmR2R+jBsyM3ToSyoTknTHRT0CsVpXeEF8yI7TlDL1VrmoL8Sk6hCPSNhM06L0j+3YoZ2VXgEBpWaOUa2IG/dkqqUGLLLSgEXYpKjWD86GACrL9wD2hFJbxGgSUMAsxq2MmpPSDFfjLtwWhLa0WhWE8H1rJwo2YZ7MsffhKbAMknZtZV0JhAbRhNuKIG4+cqMPAzWAmzXOyV9DnPFoqbbrDgCJD6cmaBF8ZkBV5TEAjhQfFGSqvB9K54dM3LWTxHtZBtM+KIVHb4j2Xv1MhtmrUDHe5ByW9dVoT3uPYjRaAJ6WyxSmDHTXRCEM5Z+IIQhsodDzgWzNh2rn9ngtF6aE3XzcTwsbGvqlOxqa6kt9l0rRKHwyVPHCDpCY1TOI/fyebj0D1wVUNaGsXHLVR4HCN3VZ3GcqxQCTZ3yy8eSCSK/GMlEkdvqShVBITCodPUAJfyoRuhIxtjSdLhPn2CsSbcaqcqw2vjjs5WdwZ/NPoAg5Z2eNP3p59L1yeOmOTQyEmrOJcQ+2pMHeiNDwE3QiFvTTyojl2bGB4w6aejUlJRQz6Z/03KDLgh4FzyJZ3LxFbnmTNRPGr/MZBgRLpqCqcOvmi3Vwp4pQzCrYDd4hChdMgKhBAc3ZN4k5ROkJBR69Z1Fu3Czr0eBgNOKaoJgvd6XC3f1z4YUy54LnnU8T8HBNmIj4j0OU11tTz7uE5+zUINsqDOkEvZwpUzbNuiL5WkSW76AUTWc1Cw5PCmUnGIzVQPdU+tN7WAAQw7JFwD3fM9x+/K1Dv2Z3a06G6sfm5VJsMnCzHtmnH1KXiNXUvlp0CxexwtK8Ah9hVDYtYgb4ICB2dPWyHSsNpUZADv25Tn9Qyi3MfNNGCBYTPutMWq93xZe6nPp5qJ7DhTYr/dJ4bTUukMqAjZaj8VEp5zVMe5TXMu1HtBdrzFsnmH9lIsgEavTvo0UXKrgV0Vd1B3VKKTITnNT9nD51ld2fKMaAiUSdPOLmZHHUJBpUvAqzf5+KkIwVxLuBmFvR6bqVxGI4H3WJmmm3pg8DrMcBw+2w19mkUyHivrUJ88jt3x2iLQ3rPYeFUJT1ZjxoDyX/qIeyGBqFOrMtoJ4lEaeUzFjTaMn7G0/4P5kNuSmLHGdiWzRKiYMLn/nMbWrW40Mh8+bsSYPwu5yUYmkv7cYx4Ut55wAr6Cj8QSWoDRlsGjuazubx7WaF6c9zSAe/MGJrEgQv/qoeuh9ZqQSeRY04RzSeEH+zc3aqhAG3IrDKtDCj9sPjE2q2WYm6Rzpq7wkgK8e3o6NSlsgR4L2VHRm3DG+Xcvl7DfN/1xtMAbeLTZ64b7j63Nz1sC24rFvjA96qjHW5tiY7Xr8hBt6CVVBx0G2aMmtgjaObVPj+p7e4Kh3cdKBoOcNnOuFh2KeCyLqbGMn6vMUnLTRcx7tiwZKw5p3yRAjnLTTLBTsiD9d8nVg0ukg+ypaKtQpQWUpO5Fni3C5fsy5gNVxbfk3tzHH3rEhv5tvXhbAvyxQkyDcgGoSHpHfNQx4S5n4JlzPtsFCL0KUiHX3Xb7sgxVjP57SnNW3NN31C9MDhr0Ht7RS2kHsnXcvPDM3Vwd1mtY8OsQhm9Ix2xA5H/+zL96vkRe4pgjDyakbk7r21L5CJoWqOKJrxzLjqSpoytcNg8NM5n/7OlYkFTw1VWHu4y7WKbL0Z1pVO+YHmdvzTEqULhiQIuqtR+hSmiS2eXGkkCw5Ck9V/S0a7F5WRdMa7cbtO4FhLlc9eWXaQbQ7vjdbdKCxF/XBVEv5Th6UK0M3+91JhkBapXIM5AV69lSm4Idm1h5uQbVbd5AXzyVlAjn1S6L6J+1lGyMjxvrI4fFsMwKnkUSq5UcSR8tOcHo5HJ5Q5a1Ni4EmctXE1OOG2eedFrH58ve/lDG6z94RVl5fJwZS8akT62kC16mIEEE6PJIbbRWGJhkfVtqRsJA0W8Csa0w5TxtCRJYDGxNYwuDwZ3TWkwsj8qK19t0WfIhNpAn7RSqGoX/DYKq37M8/SeQhyRqzzLN3G5yI+tLprYdfCyrMZwvoJFSUfE8JXCCdafEmcHkZUYVBiubUMJmwbPM1AlNgE0y62jtnfW0LN5mIHYxMU9/ktTF1hJiMEd8WiKnTGfB+RZGvDsD+3vvq3J0yK3dXQ3c5b8q5nyXTFLqehVsj23C6BJsxk1sMlbR9qHf/zTfX2yGlKy86bDOwpFDNDv9s5a1hm5a47fVJJk6fFXSfWlTg6iPcBmgl7cZWBzeOtAymPpWl9WXMGs8OTyb3IqoPi9nTZq3gSgE1Hvq5mk1Gnk8ub3DUqovOExKrLXCZqP5sEIwZDWGS+WNcFBBOOHO5j6kMCRCnSZwn15+3z3UV3YkCEmLwC0uNGgj5Y8jwbZjWwbpvffgXNsOc1NXPx6VKUT6MCUP9DMQ0hkR/3ItAuzsDNFJ40cJfrzCuBJ5o4Glrpdt4PIn95lnkP1DTH+3yjdoUTkW/oDcUh/6AlEbvIhjP0nsHB0Z5NKYrbkbL8Nnq8S5s53h+eLAFH7h6yCTnAmqEoDIe6MPPTvUh0CbXCKHqiY5X61fWZ1B6CvwF5dD9wOXpgh4pyUsSSeZzfZeDIIWMzKRHw26bLHAtBdaQnW9zKbngLzGaUjqUvnMvlTqu4pNuoNI8B6QbbzYiW5qvK8D1tZ4qjqMmsrEyybNwzt9tW9UXHi+QZwUlu0Ua0XLBwNG+pxF6Iog0VXsv3kSgC0WBV/FSC8iKfOsQhMAVu0jqn2h4y0qeEVs1t9L2xvPb43z58hzNMDDW6vxl6j6L9ZAPCvVUiaGgjH9SeQFkVXlkCbMH2A0JSOjl2ZEgpKhk5y7SSuLFRXhD0SCTRp7d53ljBsa1LkCrMLgjbgtzz5vRgFxCAIy8F5ZcmyZBv2wQBULz0amkxqG7kFRXgxlPVHQfW7uz0jzjBj7jlbFpxMhRWjmNnL7mR95R9YFUQFDD9Mfkc5IU3VBRdE2OZCiPWsb3kuk4hO7NRkT/3dY9Kf3Oqsh4lTujfPCrb4PDFqFebHl8U6vNJXXNBl9s4odrH+aqUJ3xm0TczyEfXXt5hIQS7ec1OZpB61KEE8cH/HJa5bfeMxtIBlOQI8aaDSN0XVJQyX5yRj9YF0rp1eW0dDvCuJMA9BHbZsRHWENzlBT6p3qD4RYavGsZBxRgK2qW9odDeD3QjPmXhfOkB9SPnlubAqwgrAzVGM51csgG3Z9wXLmvwvNhb+Qh6eDsgKN7K6zCMTC4P0pmoN3Yjfh3u3D0R16gQdBSAdoUWlxjZRY+h5C9DojAsIT1kmvtUHT9/6D6RvpukSAuiiEDbnqKD6hvA7R8LVMHJUsAbvmAJGlh9tx0lCh0S6dv6TH4hvTw9rF1BaZHHJi+uvTvG5+1KV2xT1ZjvRUSSkvlYkK4eEB4x6W5DVrbEhNUGKF3FeS5ktyHdoiKyKhdB6PivwuxtY1JJDnxqo7xBnWZKWS9uuK2NdMmUHNPvB5LVGhg3cePg+yNhwVbSWBy6XK1lMVS2rH19bUVdBQ3Abaq6tEVzMFe7aWqsNC0mf4CALRW6SWC2yHbOt3hTbbvUh5vXhhUwfAWUw0NybSlxvLqHsmpxBY32gURUcXC1qNcW3bMgLgOSFKkWKcKV8W6Rs3VZbSSS8yGmHZtbrwcT8G8twBZCf8dHT7kDbXNNIpkMA2WrrmhIFBd7B5HDF3R38jEbjNgQPgW8hH/3xrNEVKBDX3lRHyCTp0YRRlYQ1QqOiVMfrWCmnw0+W95k6HY7YtpzXJNhjf2aCukvKMf2gfN4upjl5kZtW9ZgEYB7nm8EwV1NpsINf2rEANb4u2UgMBjljIO8nLuFx19A4dtzZmDB3K+2HdBAX3S2a3O7hjzaEe3jajoKSbyaacGIkPsmmZXe6yEZ1+2jBKXZ2g7Ooo8wQ5lDxl1QhDiafHkYm8Vt5MhYvqTfMFaOg9Kp0XcTMfKp7d+XNW2pgiPr7Bnz8aRKNqWSIe8NlWxhEeqKChTk3r71HyXmSq6KfmzzjF1kHPsnROzzP7b3g95sgsBaD8HUFrvbl1TOhnj79dsKmSR/xHVnhf0x7Cb4VAczU28JWMpTvFfDRddbjRrlL1c5ZBDiAjtwu0T3UX3SgbSAtwsdI+ou+EV/OX0xKJvzVtLIHIOyqmtKedzvUPo0o1MGpRTCLkSwk5jAyGcSWQ9mRMkAvXlL3iCfQbO91bZZD6hWgGdUZUeIaNMY8MfMTUfRHt8EoDvDsmrBbDTtf8vH2QGgwjoMGfEV7Xrp+LBLV8WhjnMIFw1F9eQzbtHqX9GxSwbAEPzzb/oIiOhlERL2q3TjdXJohW9zx1V/Br7kzWgLOWvJUiCb/K8Zv2DxnKnTUi73Mf2A03EmgK9oNzo6YvfY8uf1Qr3ZU1dO8vSFZm8DktpMSlrHTSW6oQq17gwl7JuajETpSHjlwh0PMLO4bL5Asy3iR9YbjhLZ5Mt5KCJyBd6eyd/C4aMCCEZI+3lyg/xpVQmJAd03mwc9IzWmbEIlzN5KqV6cD7ZCtqmSEcR3IF4mu5wHefMy6U6HCK9yD0QlrxQjADqjNgYSLtmYXtABDbOiP1+PUn30Z5TsOe0F20VJsf1fXqzdpdz4PhTwFh6R6jsPh4FYD2aG7W5zQr1X+8gZ4vGhOAUYFNESoSwyWRZvgFbwOcH7qfEXBM1z7RQaUUAQbAKV9MEUEVOsxETakE1iLOjm4D3HkwqSJV5RiBKTiqLaOVCwROyyjFWaLZhDeISCbTkk4YM/PNebb8ZFRvfWzHHGqVS1YLJ9U1aJNSVWnSbyWjQSZSR7a1U0ilWEHHKyPVTyZcfs3v9s0fhaQdmUSOhHMzqs4L1aGKvPAz3QXkk1ZfyNjtAUCj5gG9Sll4lRzNVF0z4b3DCRC4ftxI7KgP6n7UxLqQ2BE95s0SBZtyJ5R/JxFk3z7AMBa2h5D6MRVnFrDsztAPjD/zRvtdaYWj9YCOmQW3E9dEFlEuMgHzrQcUqMeDDYMMMYyLEpTs3KNAmyKODPV/pJmTYq8xGnXFhG9pcDyspzdwRQmVmvFxkcmm7aqHqOcAXl3xpXXhZb+QuCUk8hDgTn1a1ybhnBUwj5lTGYPCg7ZaYEzGfvHcVnE3VaxbcxhrTLYNYrtqWNDT4CJ3o3gy/ub6LgDkgqXVmpbuKdzGRZYhhPXgz9/52ZxZ6LybZBDUafbfj5HmE7HlKFhmfJRRu/yOZqwZkFUbDDuiHM2Ln9VqNu5FHCdXd8ufFZk3ZFHx5IM9hWiVGEUOpl0ghpj98dk8rDnV1QA5a5/3Vdi4TL7w2HzESS1eZ3R6LJYoZOVn5JJWJkozGhpNM9QSyf1CZ8gJ6k1yb95HawpIwnW6tCDtj0lDR6PRGz4yERO1Pq9vBuWnCyPMX4cyQS4q1wTwyF7Bn1+r5uw7QzvT0ACSNk9fNNq8CAvlHTe/ADJ3ORHOBcGuuUF/VqIdBg7knZE0fO8IdMgfp7r2wjSvMQvFDfaR+5SjbMYGAmYUZK5sncXtQzNx5k/OoTVOWl0ODsfgIIfyPKF+pWAmeCFzbaTUBFnrcFT76HrQ2m2TpxgODR4s6DKsPw2h6Tz96sYqYgTfX2ZL8PQqMpJrBt2XTFCzGr5ohJ9EvnQ8OXu99rpYs9vdcCDOFqvXIq9VmRybF6dNbOrSjnzY7p2yZWRRxHDfE6ILMIE3tyqJdQnYMRS5FJKUR4LRF+lRN+TYg2BMYbtIEfQH5JBn6u4eXc03fQL2BVOD5Zd4YNzM8VpXMgl71ZP5Wxo7AnWS4iHSQ91K9VCyECvB7mE4YWvz93AsKXw7sBMuYMe7l3YpvwzG8o8+oV6JmOZHTnOoUK4OJAjw7hoA+zmavJNxdi2l98PoeLlnQDHbKoFbT8tm3vGsJyIqV2lpfZlOSPWLC0ZNkGxWSb+iJw+7ysbqHxOlP9cftxdkJ62ZAiJAHjPwpsWNc8ib6+o0TI1tg+ieb6EufnyPzPZI5ptkZH1cXUWV9AvcT/U7pQFDq7ZKs1qwhYR8HWx6Q5OWW+UzNw+HvwFbf2gvH6TuGz25iJnqqu55Sk7KOvT0ZyO6QZYAtLRWVNQSCl8u6XKt0YJ0l7Ml6JkVJ+fjqSfUI0a6/RXjHaM+9gd00CKmrC5bXeR07gbxDyMnt+v7+GKGyUvwuD+QB6pLb3v7hC3cn/GfALHWcRxZoMnHk9cu/lt7ulXJtoHNzd7mAxSJn0Vpg/1QuwsaxtA2n5g3gtEyMZmB8VgBZz59DZs1eSxwZwK9ctrg6SLfmEMU7xHHddqcuh28Uc8D3XGY/5ZSQ5hvJf+/aUM2Z0O2XsaItjTxlEd8vF7LRB+uNeFJ2ZcuR2w1aWcvcJE/0aCq4mVmJxfVZE3rn/dZDbxZUs/kzlQpINOKvmySsZ6cPPVjXYEWj9aIemMDVvRc7NwvvWlpGlgFqPHChPJqkxeqaHIDkKZ2s7Z71FYrHVzG5njdzdGvddQMXvR1sa3mXwAmd/E4pFZ8xECNfOwqRYWnP7FzBMT4nCvUIZUxaRgnLH0ILh2hxN8Dyx3jSVjDfg0oDAEU4b5vwCAv5k+J36HuA4vk4fy1jzF2sxgGOVxjjzPhvn95TFWu9YQQF63xzw6CINX6wFtTHBS7ttvANWA6j2CyGnxCJJsKCq3e4XTMnDyIUT8/0HYMyaDwwtl2TiHrTrHZ3t+QtO+IzuKbtFczsz/DaWG3EsBbVSZK1wdIRk2ucHEpyE4QlQm9XcRIAXbfcz7OCdzwsqTxhYyOQR0BTd7s+Kw4wzH/+yrZJxdCyQtjaOH0GaGrOA7gCLZZPNzRHQ5vcX7FRLCBnSB6P6TfaQ3/dTFSlgEjuRG7NWnlG0izUyPzfAAE9SBVJFfy2AmGL1BVw8XpuGGNT0GsyE0Oi0Jvcm0V2ltF47YxQcaJWgVnIXaGG2ysVd1V5QuFKr1l7YpG8LvuiMfLLyzzSXj6ChXrIaQR/ZBw86yV8a1A+OZ3X5MG2ghLBAF2ZS8lU6blMBVLqUGs+b/2BmMN/v8hO3SHAqGR3EuTy42o7Cp6dJ1YEtbmEPf0kkcmIYNdJkvjd+1d3dQgr/QWtyHeMbXu1rWwvmA8rvXN/0RO6UUj3GExR3YMZRXPsBUdKtVIMMaTQ16uKR+sxCPWn/gMJDzi+xtKbNxwqbpUJmRH4zaD3Oo88BbYxYYN0BVgE2BRzxGtLgKfFj8A9RFqEYH8UkMTa6M0nLfmlWoePOnRDsHvy/y25ifK6XqZ2gSTI8HbcnfJ/YGHDU0pTUhOD6e0HoY8uGWrVwmd0m73Zbl2YN4HoiFIx4t/t/2E9iQ3idWBJaNBKnvHg0E7wnjBI1HiSWpDrpVg6BUr6d2NeaqpDlF3gzbnBG90DoqvUI9Cbf3r8pyAbJVVIPLxP19AWPZ48WACx/Y3zuuOBc4MUGZafxO4YWqgfX58jsP3kqdpyJ0jCM2aX7MYeJ2L8y4q+XPnpDc8LvPu2m5qwTJhlBkqXDDVaJ92WZkBl8Wf5Hx1gIQrGfiEVpkNUHG6ToRL7qxWGgUeKsyN/k2ki0DKtC8aqTiyoj5GgANJNa6tN2tiCHQIX8jSDVZFs8AAkKzDxL6zQlwY3FZ3OY5jKHXhmE3yjEJH1tldM0ZQtvCmlmKRl9Fp6ZEWhXJxAUoQtwcv1rw88V/D0MpR0cwH+nSpgqLUzQhYnA9Gg8jUlftOkfIk3Xn4Cw4IIbmFqZMT1B9UqQYAf4pAHi92T8+DI/5zhi4z6NRxS5cFa6P0qjI/kzqWfR9YyaImL8kPDIqOhUPDO9x7nTqp1Rim0WdyYpg7X6Sk9olWKFxbsGdV2/CmZRlJCsOIjFkgy6xwSDEEHZX4dE9jZeGeKLt6wxK38H/FnbdYMyS+0L9fcXyx5QGd3Y62rXM3wU4dU0AFMVyy2iNvQF4kRH07/Utc8c8+DP5cuhVnINpPO9TmN2yqt/XR4kD9+EOYWHoICNgyrRxTP+bPm8fxNB8DhddtO9xmRVqc0E8G4BDGvbmR0ByMuSq7cJvbTgdhKmPLcpEjJo8qvS64S0Zb1qL3Mi3DyH4Iptbxj5zgzDIDhR61bXgOGl0TYsK99AiqcvCZATGuUc7xNuLppKeH8k3WpXxrWv91tj2Eh9rdv3dqNkwQc62fFhU0oS2ZQi+GDwWcI4lAFH6yClsFr4fqozxR1mBPgyCl7uNvHZSaQF0xJJfYn+BpMeB0rAZHQHZ4IJPn9Vk91V5UqmMSwLFtw9Ghg6BKSt/TzHSXjPlDs+v4pfq/d/VuGE6MQO6EtAjFnrzbljpe1YNQPQwgNSAcZKCsHL80EeAUNkNAV2QVT3Xr4w2KQqsuv6+3Ro+rMddCm8rDL/Xbz7ffc+b7dH2/ITZl7xwhC31Eb17VRaRrBwQY8ORxC8KMvXFd+QZLE6Cd6xL3rUYMQ71Rkx4+pFlWz9rUaeT85Kp6Q4QbCBBJmAyK9lxBDz0oD7FctbbIW1/5Wkl4CaF2DCrkxKw0W2Ic9GnFuzR4jfcJfSg3Xb8S/9PkyXoRIfdQxXaPNz7tTYCuzTEB+YWbZ7QzM/GPI9kNsoKRT8xFLJITX9jNlNM8lz1GbcPHEt+b0mP6ecRYW6Rtu88mR6kEvgtGcnB2ZwDthh7hLJ6IVmWTeyZPzNrk7OQ+5QnpPsVO1CDrnqWyxRWLyXpTx8mdxxv3XQ6F66pQTRn9b79p0tJTaSO8dQNiUBq+I9zZlXfYQs33u3vNTpU2q37qGfcRqeljMPYpXNOZwmNS28exxcUCTr2uM8HMZwHZeZnp27JI4sYXKZDTShM60ewAiSzrAr5nbI/I3/zFthqcpZSNcCPvXc1GX29fTflYfkOTu6HyrzmHMRFuO7fDB38TgHUeCMeOJLQ3O/DN06HpOxyTW2GPnWqVVMmLEcZIUl3b2VQPe6i4o9v+ZHo1S5flt/jbV4CZpQUAUlwBdnvKNjdb05m0gVjezCMtsy9Pr87Xik73gxbzHhmXcg/gnyFMS1uPqaHRbvZsSRjCsWtJrFgNhFC9AQj7xcHdKOXzGJSm9M+WX5/BKsWTx0Oqqdc8DtxpQRIW7y6AYD2JI+M6sP+QZnsJ/vKRk3qqMR8thrw+X5FmCRJJbaB+ZU9RDuBeC7hcCGeb9uaz0XBsKqBk/5SH9x+BcZaNxvuTC9HKPZXfDafwmsHjC7uWjnsQCI3ZV4QeB0n3N7cUS2aVDwpHRKVzC7Kv8QKlI6bQkyZDB2TjM8qzMzQ5m5V3o3C7jpJeMMUY73XqT3YEwQ4vAJb9nMbW4VoEK25dTvC48OzqnNQyGDqz/jeAT1OyTUIUJbQm54Ae7NRtxQeZgFU08tmxpxSxeYc9+oWbSXNgjCq5smRHO/R8qOjhsrjItvjvf8rtNAh7cO+jXpTxRcb3q/uhdpfCSibmkv2uU2+AjF1utYD5lRjtHRxxUpH4yRyILDj4tqBMPJPEiyIJgXGtbg9RDlCmfyyvoCX+yjvBvSr34JV9mqu8RshbqWfb0HoJdrYQjUFYqH3Zp7ds4BvZBvftrYfPA/dp9XkxkKq7JLRRlp6tuALxvm+PqvxmNOibvxiJYSp8DlmiklSg74LnMgjEQCL/EEgbNgKJDfXo2GS1lyx2JKJSbfyWHIvvJxFmiUqJnRINp8t98Svoiqw3l2AbFwvP7eDmGkZbMc1jp99MXQ4sNPxWxmYp3NZA9n2BefL0nHDBWiFXhVmNg4rgJG2+QwEk4rndXTwU/fW7jw76m6O1XAsXJHXD9KCp9SGGyHYo3ZtaplRfBV3uyCo4W88Bnl2n8OWfvhupnKbQgfDmqDmGYcP8T9jUW56T1AkeyQDXm4OO84EsAhezlr1CqVZAN8pCtI4GIAjuqVH6oRDeNUCuDkZjoMB04K3FyupL6BX6CL2gntmaWXfIpeRXGsOT+Rfcw5SvYwaiunrq1HVjA9P+oSpqu7VWsQyntwQj6DVm8Tk1QXivlhH8pkOZNLeNRo6ojhLRP1HdIc4JL+lUgVrtLI7HiCIq7zldCbnvMfYvRb5xWaCk6XXBsJtgiKaMlSQ2kIbyXn1MJtHyQPPWCpQB+hSMtI+Pk0E6XzHhp1GKvFxwwSwXvhtt6ILhsiNHY3OKfArNRLiNTRPFIBFOJMdnRq8LKTlAlHfmFrCLGlE3NJd4wfTLsFaNaGeg6nZZMpehtZRWkqsqIgNMnZN6lLmHMzi7ScK8sDgQTEaOaRBEsE04W4/eNrpMLAsm5mdajzOQS8pPmxKcttVyfcgYTWK68DsQZi0GyH5iE9bK+PjIFZ5x6Cyq8dvcu9+r6cvT8aoVGOb4HlDjrFhODdVGqQQqz5gygufMCeDcpAgSX1Om6Y4vswpmRIo3sNJehm5RvK6wG1HhQTeQfrpkNtbAe/cG7WwNUEWkCBjXEpGM4czd2D5cF7h+3KlgR3hSuqG+kw88wcdAjLcuGyWoa8YGuvfK8+cIKFiTN/vGcXuL0FZnnfw4GQdXPUwuZ9CC7hNTC4fEgZqKfnOYWcL5mKnl+ErnvHYQ3FQ==
Eokg7RVQ7oVlAICBMaS9AtR+RopEouIk08AIgHWln1tQyJ4k0s+OWD+7T6Oj2XkQCvMgZKVU/

VhypQp6N+/P+7cuYMvv/wS6enpmD9/vhajJSIiIqK6woSZ6jVlhetbkSiKPQUUlF+gRGRmU5w
ku/pA0pQVrqlqkpKSMGvWLJw5cwatWrVSeey3335D9+7dMXbsWACAk5MTPvnkE8ycORNfffUV
DAw4vZ+IiIhI1zBhpnpHEAQoHt0tHkmOOQkhJ7Xc9iIjM+i5+EDftQfE1q1YTIiq7cqVKzA3N
8cff/yBsLAwJCQ8KRz30UcfQSwWlzpGJpMhOzubS9sRERER6SAmzFRvKDKTS5LkE1Ck3S+/sb
4Ueq28Sipct2OFa6oVffr0QZ8+fdQ+1r59e5WfCwsLsX79enTo0IHJMhEREZGOYsJMWqXIy4Q
sJgpFMZWtcN25pMJ1Z1a4Jq2RyWSYNm0aoqOj8T4fHp6pUeudYXEsgVkJetlSixb6PRr
bUxEIhGEp7b5eyUiIl3FhLmRUKQnIv+/4i/20p4jIG5qr7VYhMI8yOLOoig6olzeKEqVX4N/4i
n2X7tBr9RwrXJPWZdnY8qUKTh58iqnGz8Ua4ubnV2rllsvJmWRDVP4IgqGzzPUxERLqKCT
PVqicVriNLKlynlvOIM5v/SygXnYF2HcibE/bJm/lpbw0crsebk9qHtd0BA4ch3k9fUWsRkuj
EIsbsfCBqC9UMuUxZDcZLbi6zKpyrL3z4LS6RNCxoD0BmSoRT+ndXy5Buf7sz46TV+SQETUmXC9NzX7a6aOcSS7S4I
a8NgjeFqNKnrHGYM5UOoUCz8XdZ+5dZf4dzXqcekIsLb4M4WXxI0UiyXl0MuDjmi/EpmdKxncu8ekrTQPuu5UOfoB0qJkfMYLkGTGUCG0bbedR/0oEw6AoURSbyVZMuRvpqBhbpmD8Sze04A6YTKfDD4LpNaK1mB4HBRXmnAyPZMKi7AjkVNb7A2yNtbWT61BqR8gB0zlCVEzOWB56/rB81oOk+yKIIbOqB9NFNV+zl7ojEDwS1B8C5n04yhxFLRVxVuEdvAjcaqqRnu9QrVN4HFLMUw6VyVeUdAxu2hnPTqGv9EYXnNcPjeJ1xKNlF2fQOdCJqSjxZJKdAtVmO8n54zfbaOecn4ZG2h9Yt4N4B1lBo4DnxD6zMuM8EsXkqf9UIrdAwI6NV2kc7kg6rXKU1P0YmD9wOz1zlyWrWKuPbHSiQ3MfUQGzmB3AYPkgMSKSJ6vXQuU0ZsvbQXrHvIpBSaLy4u1WOTfx5X0MjJ8yRGcvENoidWP1xJm6X5nW4mvrfbzTVTSWJ5oJT4HpDbXeL61z5MewMTWDVf9MXbwxw7KwvH8BRLdMuwIdyGlZ3BAPPOBjyLVx8tYVAd73enDcgf0Pzsdt+OD/vGx9KNzDP4AkH0Rp3ayZdf1KRgOGzpRaxJKdTX7KZK+lMjJDpSuyyFvATMLzsWzbWBNsEB4v5pl0DjhElAcbr8EbIfuVTJgJJlqXlO/43x8V7lsJJR7Y8Bh27+eKe0gVpgSYNXojmSpJ5vhLnW+EG4x4SC7Jv3Rv/cYbHpbmI+Fkx7+WTR0cexWUbXbmj3a7OOl3KhGvkKnnrk4ZqOI5a1LLedVaZo5UQJ8ZcOrJLAHZKoZ4TgKbT5wDbYJ/I6aQvBlkTAEn5Do3X/5jjFJUbSL3TJVhG3ZQCEs5WRTVz3LiX0SE8Q3NW1HYCRwaQLfJ5XLLdLsHJXSvTs7mMa6YKhMQhBVbEzR4dP0CtWXuG33wBEzw+H/CcRKLlkbvkaEnD64hvSU8cE2uvuaCtuOuLxyfORDR6kNqKWKyUdchgGJBg+aJY+w5vqkJ7u3WF/xU5FeI0hoyAVt3gYPNj7oaSWPaFaE3WOSZ5YEHHFqsRmYPcjXwmV3OWRhJ65mWPqwBa2ofUd8Dd6R6wkAOd

5eVVqwESERFR/fJ41hIREZGuq3LC/Omnn2Lo0KF4+eWX4evrC2tra6SkpODo0aMoKCjAt99+W
xdxEhEREREREWlUlRNmJycnbNmyBcuXL8fff/+NjIwMmJubw8fHB0FBQXB1da2LOImIiIiIiI
g0qlrrMLu6umLWrFmwtrYGAKSnp+PBgwdMlomIiIiIiEhnVLlKdmZmJvz9/TFy5EjlvosXL+K
NN95AYGAg8vLyajVAIiIiIiIm2ocsK8aNEi3Lp1C1OnTlXu6969O1asWIHLly9j2bJltRog
EREREREkTZUOWE+cuQIQkJC0K9fP+U+AwMD9OnTB1OnTsX+/ftrNUAiIiIiIibajyPcw5O
TkwMzNT+5iVlRXS0tJqHBQREREREUU0kPsrB5kO3AADDX24DeyuTCo4gIiqtyiPMHTp0wPbt29
U+tmPHDri5uVU7mNjYWHh6emLHjh2Vav/nn3Czc0N8fHx1X5OIiIiItI9vx6+hSuxqbgSm4r
fDkdrOxwiaqCqPMI8YcIEjBkzBkOHDsUrr7wCKysrpKam4vDhw7hy5QpWrVpVrUCKiooQHByM
3NzcSrVPSEjAV199Va3nIiIiIiLdlvjwyXfK+w9ztBgJETVkVU6Yn3/+ecLLLli3DsmXLIIQQ
AgCRCIR3N3dsWLFCrz00kvVCiQ0NBQmJpWbKqNQKPDZZ5+hQ4cOJiRLWej4iIiIiKg81V
qH+bnnnkNYWBgsLCyQnp6OrVu3Ij09HcbGxtUK4tSpU9iyZQt27doFX1/fCtuvWrUKRUVFCAo
KYsJMREREREdaLKCfPFixcREBCAd955B8HBwVixYgV+//13mJqaYvPmzQgNDUXfvn0rfb7M
zExMmzYNn3/+Oezt7Sv1/D/99OOPP/DEe+/hyy+/xJdffolVq1ZVKWGeM2cOunTpTpgsGDB1fYNjc3F8
HBwQgODoazs3OtJcxisQgWFqycSA2LWCxS2eZ7mIiIiIiodlU5Yb5w4QKWLFmCli1b4q+//kJ
+fj5ef/11AICfnx/++OOPSp9r165dOH36NP78889dy6cnZ3x7rvvvVjXscikUAjIzK1ds
jKi+UCgEle20NN0vaMKLAkT1A5frISKixqLKCbNYLIaBgQEA4NixYzAzM4OHhwcAIDs7G1Kpt
NLn2r59Ox49el+TqvuXZs2cjPDwce/fuLdXewMAAnp6eAAC5XA4AGDRoEF577/TV8/fXXVX05Sj
KZotrHEmmDIKhu8z1MRJryeLkeAPjtcDQ+eaezliMiIiKqG1VOmDt27Iht27ZBKpVi//798PX
1hUgkwqNHj7B27Vp07Nix0udatGgR8vPzVfb169cPH330EQYMGKBWyPdZkzufy75n2+y8PX+Pr+ZjWc
nTr1g3Tp09H8+bNNRw1EVHjU+WEedq0aRMa0evIjFU6kIjdrv8WKtmJ+yN+6m4Bm
Hhd6lYtv3xZ0RV7y0eQrnjOykb9NQXAgm2OHCPs+Nd8S1TVHs9qL+aHX3h19YFkBHMYfZKa4eoGJ2lmz3fNx2cEtbkB60C6
+y1hehNzzmcjLNzE2u1AVfnvvEJPZ0Jk2FJ0e0Rgjx2PE9YFxtaAuZzXJQhvq1LmGCVIlW72hvyyo1x7ZaS12nKpnB5g
J2Ip8Zv1+5lEi6TyhbJ8lkDnZrbhgoO+XGm7+UzcxoS6R8EYSX1uxb6Ox6NHt6mz5AMu6Tbzau8b8qIPLRNpPo9d7Ap
G9SGM+33oPGfZxQBBWZq6LDuLNjYUO5pshaxR2+4L6+4wx5jzxLb7bKTzU9oR1d7Lkry9cOJJW2TODcb3HdC4yNrUjwkmb
1Dn4QTDOmIBTGkoJzwt2OPsWKO6aQJ0oVfzXwBAs2bsWvNsOG/u/KkaKP/KI8eSRm4DcjZK6Fp/evsvLK7UUgvQZK9ekGMOdqgYqVrs/qBmZLLU/I8uXqW2vtZ8oJAqGQGr+u/NMGMEAlgmO4Ku35b4Pq0kkUyZsuf5lg+hQ0y0tuvZggAPiFDg+lZF6nLzthqM42Ld8C6x5ysyKgxI8iXgwNDUJDm1lQZ6gXl0+16sSwsJY9yVFhYsDMHxuP1bCSsGHtDEZ2S

5eVVqwESERFR/fJ41hIREZGuq3LC/Omnn2Lo0KF4+eWX4evrC2tra6SkpODo0aMoKCjAt99+W
xdxEhEREREREWlUlRNmJycnbNmyBcuXL8fff/+NjIwMmJubw8fHB0FBQXB1da2LOImIiIiIiI
g0qlrrMLu6umLWrFmwtrYGAKSnp+PBgwdMlomIiIiIiEhnVLlKdmZmJvz9/TFy5EjlvosXL+K
NN95AYGAg8vLyajVAIiIiIiIm2ocsK8aNEi3Lp1C1OnTlXu6969O1asWIHLly9j2bJltRog
ERERERERkTZUOWE+cuQIQkJC0K9fP+U+AwMD9OnTB1OnTsX+/ftrNUAiIiIiIibajyPcw5O
TkwMzNT+5iVlRXS0tJqHBQRERERUU0kPsrB5kO3AADDX24DeyuTCo4gIiqtyiPMHTp0wPbt29
U+tmPHDri5uVU7mNjYWHh6emLHjh2Vav/nn3/Czc0N8fHx1X5OIiIiItI9vx6+hSuxqbgSm4r
fDkdrOxwiaqCqPMI8YcIEjBkzBkOHDsUrr7wCKysrpKam4vDhw7hy5QpWrVpVrUCKiooQHByM
3NzcSrVPSEjAV199Va3nIiIiIiLdlvjwyXfK+w9ztBgJETVkVU6Yn3/+eaxcuRLLLi3DsmXLI
AgCRCIR3N3dsWLFCrz00kvVCiQ0NBQmJpWbKqNQKPDZZ5+hQ4cOOHHiRLWej4iIiIiKg81V
qH+bnnnkNYWBgsLCyQnp6OrVu3Ij09HcbGxtUK4tSpU9iyZQt27doFX1/fCtuvWrUKRUVFCAo
KYsJMREREREREdaLKCfPFixcREBCAd955B8HBwVixYgV+//13mJqaYvPmzQgNDUXfvn0rfb7M
zExMmzYNn3/+Oezt7Sv1/D/99BO2bduGpKSkqoZfHqNt/OTaRVIpHqNt/DREREREES1q8oJ8
5IlS9C6dWsMGzYM+fn5+OOP/Dee+/hyy+/xJdffolVq1ZVKWGeM2cOunTpTpgsGDB1fYNjc3F8
HBwQgODoazs3OtJcxisQgWFqycSA2LWCxS2eZ7mIiIiIiodlU5Yb5w4QKWLFmCli1b4q+//kJ
+fj5ef/11AICfnx/++OOPSp9r165dOH36NP78889dy6cnZ3x7rvvvVjXscikUAjIzK1ds
jKi+UCgEle20NN0vaMKLAkT1A5frISKixqLKCbNYLIaBgQEA4NixYzAzM4OHhwcAIDs7G1Kpt
NLn2r59Ox49elTqvuXZs2cjPDwce/fuLdXewMAAnp6eAAC5XA4AGDRoEF577TV8/fXXVX05Sj
KZotrHEmmDIKhu8z1MRJryeLkeAPjtcDQ+eaezliMiIiKqG1VOmDt27Iht27ZBKpVi//798PX
1hUgkwqNHj7B27Vp07Nix0udatGgR8vPzVfb169cPH336EQYMGKBWyPdZkzufy75n2+y8PX+Pr+ZjWc
nTr1g3Tp09H8+bNNRw1EVHjU+WEedq0aRMa0evIjFU6kIjdrv8WKtmJ+yN+6m4Bm
Hhd6lYtv3xZ0RV7y0eQrnjOykb9NQXAgm2OHCPs+Nd8S1TVHs9qL+aHX3h19YFkBHMYfZKa4eoGJ2lmz3fNx2cEtbkB60C6
+y1hehNzzmcjLNzE2u1AVfnvvEJPZ0Jk2FJ0e0Rgjx2PE9YFxtaAuZzXJQhvq1LmGCVIlW72hvyyo1x7ZaS12nKpnB5g

T0lBbWTfDFKC9MXX4cGTnFt9IYGUgw9d0uaG1vVqujsYb6EthZGsPOUv2tOYIgILdAhkcZqkl
0alY+Ukv2pWUVQK6o/tzvgiI5Eh/lIvFR2bf3NDHSV6n6bWX21BRwUymamhpAIub91ETqMGEm
IqoEKysrrFu3DvPmzcOQIUNgbW2NadOmYciQIdoOjYiIniESiVTWUjaW6sOlublW4jCR6sNEq
g9HW1O1bRQKARk5hcpp3sqk+qmp348T/+rKzitCdl4R7iZllxGn6lJaKgm1GZfSosaNCTMRkR
oLFiwotc/DwwNbtmzRQjRERKSrxCX3KVuYGsKlufo2RTJFcYGyjHzVxLpk1PpRZgHyyrlvuyK
VWUpLX08MC1M1Vb81uJSWIAiQyZ/UTMjNL0JMQgZaN6/dmQNET2PCTERERERUj+nriWHT1Ag2
TY3KbJNXICuVRKeV/Py48vfTyWZVFckqt5TWs0n0kyngUlg0Maz2UloJKdkI33tNZbQ9r1COe
T+fgbOdKUYPrNy96URVxYSZiIiIiKiBMzLUQwvrJmUmjYIgICu3SDlCXVdLacWnyBBfzlJa5i
YGT91PrXpPdVlLaSWkZJdb/TzuQRbmbzqLGe93ZdJMtY4JMxERERGRjhOJRDAzMYCZiYFWl9L
KyClERmWW0ipJpi2aGCLqWlK5S4UBQG6BDD/tu4bPP/Di9GyqVUyYiYiIiIioWktpXs/de0u
pVU1sYlZuJ2YqZUCb6S7mDATEREREVGl1IeltMpz7zp7uZDJsxUq5gwEzVArBJJRERE9ZG2l9LKz
a/ZlHGiZzFhJmpgWCWSiIiIGrKqLKW189htnLyWVOlzG0v1aylKomLVq+tORFrxuEpk3AP1hT
IeV4lMSMnWcGREREREtefxUlovezlU6TjPts3qKCJqrJgwEzUQgiAgfO+1SleJFGqyLgQRERF
RPdC6uRmc7dRP7X5WK3tTtLY3q+OIqnHwkzUQNy+nmyPKzHleJJCIiaqzsmz0pStW8mYkW
I6GaEIlEGD3QHcaG5d9Jamyoh4/83FnLhWodE2aiDsBuLElQdVan/u5sM6ioSIiKj+e69vG3RoZ
YkOrSzxbl9XbYdDNdDCuglmvN+1zJHmVvammPF+V9ZwoTrBol9E9VhWbiHO3ExB1NUkXL+bXq
VjWSWSiIgaM3srE3w6rIu2w6Ba0sK6Cb4Y5YWpy48rC58aGUgw9d0uaG3PVUKo7jBhJqpncvO
LcPbmQ0RdT8LV2DQoqnkvMqtEEhERkS4RiUTQkzyZIGss1eeay1TnmDAT1QP5hTKcj36IqKvJ
uBz7CDJ5zQt2sUokEREREVHNMGEm0pLCIjku3X6Ek9eScH6IQplinLbW5oaQK6AyvrLZWGVS
CKi+m3Xrl1Ys2YN7t27B0dHRwQFBWHAgAHlHqNQKDBmzBh06dIFkyZN0lCkRESNGxNmIg2SyR
W4HJuKU9eScPbWb97EAN3a2cDb3RYR3RYuzc1w/2EO5m86W0/7W+7SUqwSSURURUv+3evRszZ85
ESEgIfH19ceDAAUyePBl7d+5WfPHkydW9EEjRgTZqI6I6cocP1P1OlrkQHpYmYOzc1bmkb/Bfsqgf
Ok5eS8K5mynIye9eCKL7uEpk+N5rapeYAqlKiJw/83Fklkoion
hIEAUuXLsWoUaMwatQoAMDEiRx9uxZREVFqU2Yz549549i1mzZqGoqAhmZpw9RESkSUyYieqAQh
Bw6146oq4l4/SNZGTll+x2thQD13drOHtbgN3JwtIxGWv+MYqkUREDdft27eRkJCAwYMHq+w
PDw8v85h//vkH//3nQ7D+cRUm0z9fUYHHY0Q9/Uh1Tw8fLqG/q11K+5h2QvP2Fff//9F126dNFswERMQUAJK5yRjqDQEVXgmeV2bi
IUESMQcY5TIGN4uQsekJxLpnfeYtTxNNlhrtstgoQYQy4wG7GNWfvTeu6/Fp1
9fb9GFRsS6oFz3kzIeXhd+eHjbOCsH1DPqHJMG9SKcaz7t9UtP5/d2ke63J+FQoLk6Kcg2kf2NYDmAGnnuoy0KB5M+fIgLnKrSPhI5XxSvy0WzPJDwRX9Mw8U+V
dDD0G4c33zzWT74a4+QsXebL1rGebzMuoNqt1j1pKmCTZC1tO8jqujPTM49Xyg/ZJ0nBPS4t8M7mxEL43VeP3VGpyS1bCajGZgNXnVqZ4qf2bfu+
wlCcRRYHL8/h3nEmEgBE3PREw21Vh5tIq8kFR9wRExEqFS5ITYCs8Aj2WRcuUr4Y5jUvVrbDx6uSe/bd2uf/7WT1VRkf9Iy3TcaP2HqWaIBNR1Xy0t0vDqTuK
Emp1ZHz5cuMtX/92JWypUzAkc0bhzUJGeunbChuZ2+9zLMB/s2K/K2JG4YVbrFDGGHPJDrmMA4fO5OXXJfzqHHzKkhDrGvVYAHAttdkM6kOtP3AjL/yOkWBtwv
EOF0HTSOjbBIvLz7JeT2AzqNHkR1jHVNHaKRMErchIoRwLtCKKsD2HjZXb5Ti+gfvsMSSXqN/FbWRPHqJ/tbCWAKjgAEyHmEsYpUUgHzmSmmA0DNP5b8sP0IpH
Sl8h+vOEbI3Ve0SukmMLR4+YvkObYaaDwO8rgTpZuU2cHs4IVrXyVYNWNfmmOfU8kNYUeh2sP5+5nW5X7B2kGi3NuixwACDo2XwKOmDwwUvETmT/Zxs7pvB9
hdJcjp6aMtZ3GM7tCB7N9JT0+AeQoe0j2HU0w6F24XqVK5mMmXvZo1HNhWSUc7q5qXtWyzrN3RuoqOq6ZDQtX+jqkSYMsZ+jlsq3nSDVK7LHqdDUrjS9WeVn
uW5tLQ9WF5i7WjZqCZf0SOzj92wUHtM6gFRnN4Gf/dBxlHNPC2pmE6sXrTtDAUDNyRh6OfTCqxMVFxDaxAvjhDKyLhg8HdfOW95XaqHUhZyf0Rgjz7XkA1OH
jeJXCSS5vCx4DcHyW7fSVEGCxKQmALhGeotUvvTKn9EFV2VvQyvxc/mZrF0b4vBN2g3h3uqwlIiQyJV1ixtY+c8cLz7vCE8rGm/4KCEuqQfS/eBVFk3LpiqJDf
EHsS+ORdd6fMNBRNBSJXPJO0pdJZP/ReiEH53bh3P/ZGzaatAa66tjSRUi3+fSCgEG6f3Oem9MOfZ9Ql5NYtuJ3J/ZgozzGKHAvGDHqmT3brRj/l0LEqpR2D9
4E0QCzkRq8lQ1VUOtZUrBCCbMaeybZDMzOjBGDs7w3mlEVkfW5gLodRtAzg3L2Kes20Nbqc3WCPBQL1YGl+HBFsLULtQfAgrpw4wLGXsaf5RhqFLzzPhyOmN
cPD2YsIEJNmj/zX1UQUBLhU2cKaTzh2o+XsmNXNvHIvSgMDtiDYsqeD7Ry6gWnmb6QAvjFPj/7J1WgUEfuhlCKiGWiohBxzE21yZcZPg6zQJwGlbbgG7LFjqNX

rrlMREQ6iwkzNQiCIOBOUhairiXj1LUkPMosvwKsgZ4YnV2bwdvdBp1aW8FAS18siUg35BcWL
9OU+PDJiHHioxwkp9XOMk1GhnqlplDbNzOGtbkRxGKO2hIREWlLvUqYY2NjMXToUHzxxRcYOn
So2ja3bt3CwoULceHCBYjFYnTr1g3Tp09H8+bNNRwtaUJ8SjairiUj6loSktPyym2rJxGhU2s
rdHO3QRfXZpAa1Ku3NxHVc4IgICu36Jl7i4sT49QKLtJVVtMmBiWjxcUJsb2lMeybmcDcxIDT
mYmIiOqhepNRFBUVITg4GLm5uWW2SUtLg7+/P7p164ZNmzahoKAA3333HQICArBz504YGhpqM
OKGpSEV3nmQmouoa0k4dS0ZCRVM4ZSIRXB3toCPuy082zSDsbTu7t0jIt2gEAQ8ysgvqUb9ZA
p14sMc5OTXzjJN1hZGJdWojYuT45KR48fLNBEREVHDUG8+uUNDQ2FiUn4Sd+jQIeTl5WHBggX
K5HjhwoXo1asXzp49ix49emgi1AapvhfeeZieh1PXk3HyWhLuJmWX21YEoJ2TBbq52+C5ttYw
NTbQTJBE1KAUyRRISi0eKX7wdPGt1NpZpslAT/xUQvxkOrWNhTH09VgrgYiISBfUi4T51KlT2
LJlC3bt2gVfX98y2/Xo0QNhYWFqR5IzMjLqMMKGrz4W3knLKsDp68XTrWPuZ1bY3tXBHD7utv
Bys4Z5E84mIKJiuflFqlOoHxaPGKek50GohXWamhjpq65dXFKR2tJMyuXoiIiIdJzWE+bMzEx
MmzYNn3/+Oezt7ctt6+DgAAcHB5V9q1evhqGhIbp161ajOPR0fDTg6e90IpH2Xm9mTmHxSPKV
B7hxN73CNUdb2ZuhewdbeLvbwspcqpEYG4r68jslKosgCJDJn4zk5uYXISYhA62bV335IUEQk
J5dqDpa/LA4Qc6ohWWaAMDKTAr7ZqVHjDmLhYiIqPHSesI8Z84cdOnSBYMHD67ysRs3bsTmzZ
sxY8YMWFlZVTsGeC4v6e09vbXi6yqqmX292biEiLyXi7/MJuBj9EIoKKso625vhxS4t8GK
XFrBvptu/l5rQ5u+UqCIJKdkI33tNJZnNK5Rj3s9n4GxnitED3dHCukmp4+QKBVLS85H4MOep
olvFSzblFdR8mSaJWAQ7S2PYPz1ibGUCOyvtLdNERERE9ZdWE+Zdu3bh9OnT+PPPP6t0nCAIW
Lp0KVauXIlx48bhww8/rFEcDi/rFEcCoWAzMyyi43pgqeTVIVCQFpa3U7LziuQ4ezNFJy8moRLMY8qXH
bF3soYPu1t4dPBDi2eSpLrOs6GTNO/0/qAFwUahoSUbMzfdBa5BeoLaMU9yMK3m87iw1fbQaZ
QKCtRJz7KRVJqbq0s0yQ1kDxZu7iZibIatXVTKSRizsYgIiKiytFqwrx9+3Y8evSo2fLsH3L
Rnh4OPbu3VvqmKJIhr06FqJRVYLBWDqs6fv4xOEunm9BUVyXIx5hKhrS
bgY86jCojrNzKXwdreFt7sNWto0UU7R1PXfRW3RxO+UqKoEQUD43mtlJsuP5RXIsHL35Ro/n3
kTAzQvGSF+eip10yZcpomIiIhqTqsJ86JFi5Cfn+yr1+/fpg8eTL8/PzQ//9dVxP9wZIt2ZUXXWfQ2J
vXoyBAwdqIkwqR5FMgcuxjxB1LRnz1EQVH5UyYtTA3RrZ0NvN1t0crelF9oiXTM7fuZiHuQ
VavnFIkA66ZGpe4ttrcy5lJyREREVKe0mjDb2tqfR3c3W9lZYUyUVpALCgtxcXFBYcUFKWlpaFBs
/bt24dp06bB29sbKSkpymGZK2AkycJeHszYfIq2AkycxYH14lSbKrgzmryhLpsH
O3Hlb7WAM9cfH9xU9Noba3MoYtl2kiIiIiLdF60a/yJCYmom/fvpg/fz6GDh2KPXv2zAC+//5
7fP/99ypTQeYYUWYJITH7ehuqFQCLhxLx1R15Jw5kYKsvOKym1vItXDc27W8Ha3hzc8
y6W5GQY/3wr2VsawMucyTURERFS/1LuE+d69ezh06BBw4dOHQ6HAzc3FbDZnDF9ozmuBEsrF3ZwQ
5MrXLpFaiCBZxtr+LS3QXtnS+hJmCQTNTZVnSLt5mgBD5fqr3JAREREVJfqXcLcNkdcs1MZtPA
C4B1k4dS0ZUdeTkJpZUO65DfTF6OLaDN3a2cLDxRL6elyWhagx82zTDPtO3Kl8+7bN6jAaIiI
iopphwqzjKrMWavNmJkhIycHJa0k4dS0Zyel55Z5TTyJGp9aW8Glvi84uzWBowCZDKEt3a2cCzj
TWMpXzrEFFpIpEIowe6l9v3AICxoR4+8nNnpXwiIiKq15j16KjroVaJFeUmLSyLREA7uu9
vgOTcbNDHi8i1EVLEW1k0w4/2uCN97Te1Icyt7U3zk544W1k20EB1R/bBr1y6sWbMG9+7dg6O
jI4KCgjBgwBMwAC1bdPS0jB37lz8/fffAIBXX30iJqqlJgw66iarIXaxsEc3u62
8GpnA3MTg1qOjKhhKyoqvLy7F7925kZGTA3d0dwcHB6Nq7ZDq1daWDfBF6O8MHX5ceUtI
UYGEkx9twta25eun0DUmOzevRszZ85ESEgIfH19sWfPHkydOhUKP6Ltw9PPs1X7y5MkoKChARE
QEMjMzMWvWLHz11Vf47rvvtBA9EVHjwRZrxYrJy5Ups374
dCxYsQMuWLbF27VqMGTMG+/btcsKRBKpVMs3lurDpbm5FiMi0j5BELB06VKMGjUKo0aN
AgBMnDgRZ8+eRVRUVKKmE+dy5c4iKisK+ffvg5uYGAJg3bx7Gjh2LKVOmwNraWpPhEBE1SmwTSp1BQeOH6
VTPNs3Q39uRyTJRrBQ4fPozExEQMHDhQuV9PTw9hYWHw+xk7j/vq4zGDdoMQx2HZAQ9tjMHb3
/DtjJNDQ8l7mYuaNqZSZoUZkqpazNs9I3Ka7Oq9P6m2xZYYrOV+hc8+CEAr
QRAFSQ2e0tY6CDVpzQ0VBFjkJ0Q1wGOKb3GxctQWEgIIwP8yuadwPRrtYDtyuKz3bbVGb
DCxRHiCzjLSqnWyV0MWSPn9NGbrTFmUyq3m8zTmT+gJGWLbmJQLP/Wr9YA5YN5Fqmm6nHe8RXOv
9rB4IBqnz3NQT0zt8YPcFQrG5oA8EoAEsFuFw0SFB3ACKZByRHDmN9U87ZF5u61rGLK6p9RWeX
Ug3r5R4Cw7RdVrVWYOkfTLOQ8RZbnSG/F6Hdb4QXVKNfNsvKHdrJp2ZP7yr1x98ngA6qSqRxcQ
nvEKSzd9oWgGdQ7lFVoXOS5L/iPjRyC0gApDVJUt9a6BYl7MzeG4GoBzcWvARSPqNFkPpyFrUs3Pj9K
L1IHMP8INEqiebLOpmAbbyu4NLEIbTrbfJ1Fw2n8cZC0ro3Xq7T3bIlc7Li3n9GaBnI1Gk/fq/6PlOKnx8GtJX9uIEcRYQ1RPLeq8Zs0cN0dQEmDRKMO+8Ud0zDQc+lnjOGTl3DyS7Idm2q6Xpk
M7pGjx6NIUOGAADc3d1x9epVZesqHR0dlAoF/tprcbYmRsXyTw9OXyeQoqQAw7JTTIR2A0Xmx7IXuBmYlk7bgpIoMFKfGdUaMMi3k3E0upmq/5X7sLnctyXoo3F5mqNGmuenvgyoGdDHgqSIomrb/a/PS9VkXPK+VnbdAz0NKkcPI+4cOPQ+UiMCZ6R1TqH8cN5d6ebu5vf97OMy4eHFQS4cKTVwBb8PNeh4RcNl3wO7aPdJvvlqxbYVdTMn5xE6XMVqebd7Dk6rtYYVBrOL94SvRT2JyGZsE0eaR/V4TUA/ez6L
ObJgYqL8LeyhuavqBw/yk+PoiwcSe6VYj/NTe7ebTA5AOL7+l9YgIgoQ7bsYQOuKIoLfmjoJ6uzKnCkmkyB0HlbUqBTjFBegE5TNNfEN+Lq5JmFWL9uIBDlOeMhlMgDgVLLu6lkb/SiPzGQOcwbyKmaDlMXZ5F4PgxMFu/vFBFk3KXDdQrXaEHS5R0aLx90PlSzMbNkxyeOcmb4Qrvhh06Tf1J3xYhcNN8qKbUW/4Fd2mvAfZ8fbMcW7rjZqmvsWmNHgaIOGEW3f3oVJUhJi9CC/FxLA7chmdPLaI8d3wCzhOnEZaqlUKwv1IPCVz2GA5mxOVMB2ZBfvjeCVCcRGVrA+m+8Wz/K1hzJLXV7hQwSzLkLLRfUa0VCQ/dS3JYv+2Uh6zW6XFd9RgO+PCc4LGJDcfVqB+vqbqDI+ZsmGkLg7Mcgy5LRDIfU5ZLQuzCl8u9dIHgxTFzvR0VcLQZXaqBDnvYILtQVIV7/W0lRQrRqVCcIpPk+tJVrH6SL/OVkZxErKCUgNzCrF9V8cUVkmhgWNdVD9sgvIRPBuIjE4n8kVNiMuDXUoYpbx7+G4smqQ2CTTQUrmttYpJeXZAMpNS+

VSBAQEICwsDHv27MHdu3excuVKHD9+HP7+/pDL5UhJSUF+fvGtDJ07d0bXrl3xySef4OLFizh
x4gRmz56NN954g0tKERFpAEeYGwGuhUpUOzw8PODl5YWQkBDMnj0bdnZ22LVrFyIjI7F582Zt
h0dEDURgYCCMjIywZMkSJCUlwcXFBaGhofDx8UF8fDz69u2L+fPnY+jQoRCJRFi+fDm++uorj
Bo1CoaGhnj11VcxY8YMbb8MIqJGgQkzEVElicVirFixAj/++CNmzJiBjIwMtG3bFhEREejSpY
u2wyOiBsTf3x/+/v6l9js4OJSaxWJlZYVly5ZpKjQiInoKE2YioiowNzfH7NmzMXv2bG2HQkR
ERER1jPcwEzVQ9s2eLCfSvBmXHCAiIiIiqm0cYSZqoN7r2wabhHsVAgHf7umo5GiIiIiUoMJcyPBAlFERFRb+JlCR
NrC/oc0jfcwNxIsEEEVERLWFnylEpC3sf0jTRIIgCNoOQtvkcgVV9HQYRVcDa2lTbIdQ69j
9EDYOu9T/se4gaBl3rexoiTskmIiIiIiUoMJMxERERkY7gBGJVNf33YMJMRERERESkAw4
fPoyQkBDlzydPnoSbmxtxahqLj4+Hm5ubtixY0eVjlu5ciXCw8NrZ9Nr9NxMmImIiIiHRA
REQEEhMTlT936NABW7ZsQYcOHbQVYc3Z2Nhgy5Yt8PX1rdJxP/74I/Ly/Ly8mr03KySTUREREREp
IOaNGmCLl26aDuMGjMwMGhMwMNDa6+AIMxERERERUQM3cuRIREREIFSoqSjkN+9kp2aGhoXj11Vdx6N
AhDBo0CJ06dcLrr7/7+Oc+fO4fz583j77bfh4eGBQYMGYMGIT1yUuX8N2/exLhx49C1a1auuu
OxL1798qNafr06Rg5ciuG3r17w9PTEx988G888AGuXr2q0i4uLg5LGG3TJ0/G888/jy5dumDkyJE4
c+aM8vFnp2Tv2LED7du3x4ULFzBs2DB06tQJvr6+WLt2rfIYNzc3PLx8+4ULFzBs2DB06tQJvr6+WLt2rfIYNzc3AMDy5cuV29XBhJmIiIiIi
KiBmz17Ntq3b4/27duXOw37wYMHmHBhmD9/PsaPH/wRGRkZmDx5MqZOnYh5MqZOnYp33nkHP/zwAxQKBT
755BPk5+cDAGJjY/Huuu+/i0aNHWLBgAebNm4+dm79+7hvffew6w6NHj8cqN69q1alyiyZAmCgoKwcO
CpKenY+TIkUhKSgIAREdHY+jQobh37x7fYR37x4+//xzLFq0CCKRCNGKCRCCKNGjUJUVFSZ51UoF8Py
Zs0aPPfcc1li0aBH++ecfAAG+99ZZyuzo4JRuAWCyCpaWJtsMgokaI/Q8Raei/Q8RaQP7HiLd4
+rqiiZNgmBAudOX8/LyMHv2bLz01Bjz5s3DW2+9YPHkyYPmNj4e7uju
XLl0MqlSIiIkL5HD169MDLL7+MdevWqRae1ZWVhZWrlyJbt26AQ8PDzw8ssvsvIyIiAiEhIVi
+fDn09fWxceNGmJoWrzvt6+uLQYMGGYeHChdi6dava8wgCgMDLz99tsAgOeeeeawwgCCPezzw
j+LFF19U/hvY2dnVaDo3E2YAIpEEIoloI22EQUSPE/oeItIPE/DlHj1rVrV+V2s2bNAKm2bNAKm2U2bN
gUAZGZmAgBOnDgBHx8fSKVSvGm0E4YAMX3RIt5eeG///4r97maN2+uTJaB4gJenp6eyinX2z
27tzJZBgA9PT0MHDgQyYWFhyMnJKfPcnp6eym0mDAw0DAwNYW1oiNze33HiqxgkzERERERFRI/
pUqm0zPbp6enYt28f9u3bV+r17t27qxx6X8CDRmXcR7Kt4kD4v7kCKaFlVqMvGXKb6SzCI6A0SwWkzvCIKA7Ozs
Ms/9bMxisbjW16FmwkERERERERERlMjU1Rc+ePj3/qMT28lPK9PT0UvsePnPwIKysrAIC5u
TkePnxYqk1KSgoAWMLCAsnJydWIunLYnLu7l3ns3bt3ER0dDVXxVwBAUlISzp8ep8/DtHJTzJTozXEfSJQNCoWKNDDDuKTEyzqsZbNWiNzzrKR7KxNkQR
AQHBwMJycnuLu7l3ns3bt3ER0dDVXdVwBAUlISzp8ep8/jzFjxgAAunRx
79+5Fp06dYGBgUO24xeKa17hmlWwiIiIiIiIdYGZmzmhtjYWERGRiIiIdYGZmhtjYWERGRiIjI6PWzhsYGIi7d+WG17ed+9i3Lhx
OHToEP755x9Mj QJe/fuRbt27co99nFxrn379uHAgQMICAgpCYWEhPvjgA
+zfvx+HDx9GQEAA7t27h6lTp9Yt obj MzM5w7dw6nTp2q9lRtJsxERERERESkAw4hyJhwm9GQ1N77ON9DV1Hj1rVrV+V2s2bNAKgm2U2bN
Pw999/19p527Vrh19++QUikQjTpk3D5MmTkZKSgrCwMPTr16/cY5s3bw5/f398++23mDlzJpy
dnfHbb78pC4u1adMGzdvRrNmzTBz5kx89tlnEAQBGzduRM+ePWWs U9/jx43Hp0iWMGTMGiYmJ
1TqHSKjtu6KJiIiIio0Zs+fTqioqJw5MgRbYdSbRxJiIiIiIi9kKDCTMRERERERGRGpySTRMERERERERGRGpySTRMERERERERGRGpySTAnBhJmIiI
URERERERKQGR5iJiIiIiI1GDCTEREREREREREREREREREEanBhJmIiI
iIiIhIDSbMRERERERERGroaTsAIiIiIiIi0jxBEHDjbhpOXn6A7g5WkAkEmk
7vHqB6zA3UoIg8D8BEWkU+x0Ji0ib2QUSq7jzIxI+/nUP0vfRSj7m2bIop73rCyc5M84HVM0yY
G6HDhw/jwIED+P7772v6V3l3j5uZWap+enh5MTU3xaPrxPzxx+jYsSPi4+ENiEWW0fC83QlK
+PRt2/fCs83f/58DB06+tC5CJao17He0q0+fPkhiSCjz8WPHsHOzk7btgCgQVi8eLHfatu+88
4uXLiAoKAgTJo0qa5CJqpV7IO0ozrfZZY4cOYYUGiXzd2dHXr16oPQiscpdWhZ/4ueb1a50yaCLu3i+
yo3HnQSZClv+LnLyiMtuYGOnjuM6AXGn3SzCnZjVBERIS2Q2j03nrrLbz99tvkvKnwsLC3Hr1i2s
WrUK/v7+2L9ZBLLVyxm5SUFAQFBWHChAnd0dNRk6ETVwn5HE7H3QM53H+3r16oXAwEC8ec3Xc
1laWiq3xwhw5goKCAhgaqqi4+4P0x4ULF+o0TbhgbhgfzhR8HSQ//B
BGRkaIjo7GmjVr8Ndff2f2H79ulo2rSphl+F7hEEAT/+dq7cZBkAcvKKsPS3c1j88Ut1MjvDzc0
Nc+bMwR9//IErV67AyckJU6ZMUV5kCQ0NxfHjx9G8eXMcPXoUG8e8XMcPXoUr7/+OmbPPo2zZ89i8eLFuHTp
EiwtLdG7d298+umnaaxKSKHa3HCDBhJtIKOzsv7d/OnSRWWft7c3HB0dERAQgHwDmSquZRGCB+FG2xkTYiGB3
wEUf6g8seywsRUUUsLS0r1Xd07doVp0+fxrFsxFjx9FvC6CvXz/d+S/Bl2wd3d3e35bbqGA6e
8yYWFhGDRoEL7++M7lyXPLywuwuvv/64tm3bhoxOCAyLeOGJT87C2t2XEZ+UVan2+
KKtX21r10jPrqAPT1yq8V7W8Brij5vGd4SDjWmzvVY999duxAU4FIAQfvn1F3Tt
2hUAcO7cOXAOXTq1Am7Hd++eXGX8eGHH2L8/eHj58iO+//PGYNn28eHj58iO+//PGYN28eHj58iO+//PGYN28eHj58iO+//PGYN28eHj58iO+//PGYN28eHj58iO+//PGYN28eHj58iO+//PGYN28eHj58iO+/
zIjR45EVFQokFQUoqKKi4ObmhpArl+/jZMuxrL1z5yI/P1H1Z1y2X/m8F75A1ct79f2oNcaMbCPkp
bB09MT3bp1Q2BgIG7fvl3mc/3/3zzzz/3/27+Gd2d/L26:H/eS/ryevGjGFSCh4OdPqA4
tNYJx6NAhDB8+HJ6enujYsSNeffVVbNq0Sfn4yZMn4ebmbmhsjISIwcORIeHh7w9fXF1q1bkZyc

jKCgIHh6eqJXr16lrjKnp6fjyy+/RM+ePdGpUye88847iIyMLPffcfr06XBzcyvzz+N/16oyN
a1aJ0PUELDfqd/9zrNatmyJjh07Yv/+/aUe27dvHwYOHFgrz0OkKeyDGk4f9PDhQ7X/bu3atc
OMGTPQsWPHGj+HLlqz8xLOXk9Gclpepf5UNll+LC2roMJznr2ejDU7L1U59jfffBMjRoxA69a
tERwcDA8PD5X3OQBMnjwZLVu2hLOzM8LDw9GjRw8EBgbC2dkZXl5eWLx4MS5cuICoqKgqP39l
cIS5kZk9ezY+++wz5barqyuSk5OVo5kLFiyAgYEBjh49ig0bNqBZs2YYP3487t27hwkTJuDNN
9/EJ598goyMDCxZsgRjx47Fw4ZWYMHIRarXns5deoUgoKCMHDgQMybN6/cqz0HDhyAi4sLPv/8cw
iCgO+++w6TJ0/GkSNHIJFIcPToUUycOBEffPABJk2ahPz6fGzatAnffPMN2rdvr7wCBQBTp07
F2LFjMWHCBKxZswazZZ8+Go6Mj/Pz8MGzYMPz666+YP38+unbtCg8PDxQUFGDUqFF4+PAhPvnk
E9jY2GD79u0ICAjAunXr0KNHD7UxBwYG4t133y3zNbm6y0zNbm6upb7e1AoFJDJZMqfCwsLER0djW+++
QampqaVut+HqKFgv1M/+h1BEFT6ncf09Ep/FfDz88Py5cuRn58PqVQKALh9+zauX7+OsLAwF
q0qNznIqpP2AfVjz6oMnx9fbF3714UFBRgwIAB6NatG2xtbQEAH374YY3PT/WPt7e3ys+dO3f
Gf//9p/zZyspKZUDp6tWruHPnDjw9PUudKyMYmBj4+PrUeIxPmRsbV1VU5v//xVJjz58D3d0d
S5cuVT7T7Ws2dPREZG4tSpUxg/fjwuXryI/Px8jBs24Dhw8jNzVW5Z+c+HXp0
vXrh/nz55f6QHmWTCZDeHi48hw5OTkICQnBtWvX0LFjR+ONN97ArFmzl1Md4enrCx8cHp0
6dUvnQePPNN+Hv7w8AMDY2xrBhw+Dh4YHJkycDADp27IjycDDwp27IjDhw/j7Nmz8PDwwO7du3H9+nX8/vv
v6Ny5MwDgpZdewsiRI7Fo0SJs375bcyOjo41und4xxYoVWFihco+AwMDeHl54eeff4adnsQ9rvL21
z01U37DfqR/9zq5du7Br165S+3/55+3Sd4eXmp7BswYAAWLlyIY8eoOoX///gCKR5c9PT3RokWLa
sdApA3sg+pHH1QZ33sDRQKBQ4ePIhDw4pn7dPnz7w9d/fn96MyjB3SCet2X8a9vda6n63HUJib
5+6QuoZTGW6qGJkX65bVramiLg9arPAHj2oq1CoVD5//P4ou3Tjw8ePBjjjx/xaAr17w8vda6n
MhBdeeAEvvpACioqKEBsbi7i4i4OOy4cQOpqanLkLfj5+aFXz7eHh4eH
yrnu37+PMWPGQBAADEzJ49u8IPDED1gwyA8kMpLy8PAJT3quTm5uZcxXFUz6KilSLF
Tx9telxJcXHHwYAYGFhAQDIyiruUCSHTqojLz07tqojLz07t0b33//PTIyMmMubl4qZoVCAY
VCUeZrkkgk5V5VIcEVxkVxFVWcEYGFvqE/Y7m+53evXtj4sS
PmTeVoRERBDZu3Ij//e9/9/MDAwQI8ePZRVBMTE5XHWVhYQE9Ps+9PDxMmTMDevXsxa9Ys7NixA/r
65d/3Uj4zZ85g2LBh6qDG9vbxw9ehRWVlZo3ry5cv+qwu1U/Dn1jeaIWxxXX...

EaTJiJiIiIiIiI1OA6zERERERERI2QIAgouH8LuTejIM/LgcTIBMZtvWHYvA1EIpG2w6sXuA4
zALlcgdTUHG2HQUQVsLY21XYItY79D1HDoGv9D/seooahLvuewpS7SPlzOQoSY0o9ZmjvAuvB
QTCwdqyz59eEM2fOQBAEeHl5VfscnJJNRERERETUiBSm3MX9jZ+rTZYBoCAxBvc3fo7ClLsaj
qx2DR8+HHfv1uw1MGEmIiIiIiJqJARBQMqfy6HIL3+WiSI/Byl/hqGxT0jmlGxwWhJRQ6FrUy
IB9j9EDYWu9T/se4gahsr0PYWPEvDo4HoUPYqv1DkFWRHkOemVjkFi0hQiPf1y2+hbOcCqnz8
MrFpU+rxubm6YM2cO/vjjD1y5cgVOTk6YMmUK+vbtq2xz9OhRrFixArdu3YKJiQkGDRqETz75
BIaGhgCAY8eOYenSpYiJiYGxsTF69eqFGTNmwNzcHG5ubsrzDBkyBKyBAsWLKh0bE9jwgx+aBA1F
Lr2hRVg/0PUUOha/8O+h6hhqEzfk/jr18i7fUED0ZTPqHVn2L/3ZaXbu7m5wdjYGMHBwejRow
d27NiB8PBw/PLLL+jatSsOHTqESZMmISgoCAMGDMCdO3cwZ84O84ceHh4IDQ0FKmpqejVqxemT58
OX19fPHjwANOmTUP37t0xb948pKSk4IUXXsDMmTMxdOhQmJpWrx9nlWwiIiIiIiLSuDfffBMj
RowAAAQHB+PUqVPYtGkTunbtitWrV+OVV17BxIkTAQCtW7eGIAiYMGECYmJiUFhYiMLCQjRv3
hwtWrRAixYtsGrVKsjlcgCAtbU1AMDU1LTayTLAhJmIiIiIiKjBsuo3Go/+bz2KHlZuSrY8Px
tCQV6lzy8yNIJE2qTcNvrNHGD1in+lz/mYt7e3ys+dO3FGf//9BwC4efMmMBg4cqPJ4t27dAAA
3btyAn58fBg0ahPHjx49e/aEr68v+vTpU+U4ysOEmYiIiIiIqIEysGoB+3c/r3T7/ISb/ISb
uB8xo9Lt7d/7EtIWbasTWoX09FTTUYVCAbG4uC61IAil+oJ+PHr8+LjFixdj4sSJ+Pvvv3/Hff
/9h6tSp6Nq1KzZu3FhrMbJKNlEDpUhPRO6+RcjdtwiK9ERth0h2LxH3/qtrtcIIioEWH/U3O7du2C
n58fOnXqhIEDB2L//v1ltiovYvx4osvokuXLnj//fdx7do1DUZE68FBE
EtNym0nlprAevDEUqO8tWnDhg34AbjYWP
z11l/45ptv0Lt3b7i4uKBJkybYvReuDGjRvYu3cvnJ2dYWFhAQAwNjZGTEM0tL
Sqh0jE2aiBkqRdv+p7QQtRkJEjQ37n5rZvXs3s3Zs6ciWHDhmHPnj3w8/PD1Klt3w8/PD1KlTce7cObXt58yZ
g23btuGbb77B9u3b0bRpU4ZyrjaMxrMwZA8LwxyzGKI8jDq+4Dk
MOGDcP69evx2muv4fTp0wgP
dXhIaG4sSJE3jjjTcwfPhw6OnpYe3atcpp3R999BEmTNnVjtG3sNMREREpCGCIGDp0qU
YNWqUchRl4sSJO2LKKiouDp6anS/t69e+ObtWvX8fANC3336336LN954A5Ac5cvX0aPHj
/RKISEcYWDuiuf93KLh/C7k3h2LxNy4sP9P9amTRuEhGoc6sP9amTRuEhISU+figQYMwaNCgM
h/v3bs3evfuXebjkyZkyZNwqIxNmIiIiIg25ffs2EhISMjwYJX94eHhatv/+++MDzg4
svvaTcZ2ZmhiNHjtRpnETUOhEkhbtK2zol66gAkzERERkYbExcUYxcUBAHJzczF6F69Ghcvoq
OmDBhgtqlUOLi4tCyZUscPGa9asQVJSEtq3b4/p06fFDxaVyRXvKoqfHO/O4RGJRBCe2ub7
mOoaE2YiiIiIiDcnOzgYAhISEICwGoCMBwThw4AACAwOxfv36336UlOss7ozfcffuXaxaxXYsQLTpk2Dm
ZkZVq5cieHDh2Pfvn3A6Tp29g2w9vTZ2+dX/MXgbZErsaWf4SfCYM4CAiLIl3t7GG1ghQA9+GyvF1zZJwLl7G9MkpNLF2R6XZX
BEYSuv9WfYBfZIRaNGnQBiLDkB+P8eP+13BaxLySQoSmYzWzYqV6ie9qFnTt3BlfT39AWcBlr
CMjNza/BKiLRDoRBUttPScrQYTd3jBQHtY8JMRERERpCF2+2Htq2enp7K7enRnCvWPlZcF8R7iEzQOdpO7lz
+iYiIiDSkf+EveP99MaOHbvVjA/R1k5e1C3nEbjV9rAZPjGNTpw3Wa+eqc4TZ8c2zK7+A/Sk/Wf+Lj+eOwJkm7FlywkkelGNWjjdEvjUhKRMkSFiw3WUhF1mrRGlqWozbO53tq/Pou9b8vaz5m18P3d
9GupVIqWLVsiPj6+RrHizKGxHVM4lqgzGzzfUx1jZP+iYiIiDSkffv2MDExwULF1T237x5E
46OpZdv8fLygkwmw6VVLl5T78vPzce/ePTg5OdV5VEREjR1HmImIiIg0RCqVYvDgwZAGTAGFhYbC1tY
WHhwf27t2L48PAnK5HKmpqqTA1NYULUzVXlxd69uyJkJAqjJQCfP3tjatCmWLVsGiUSC119
/Xdsvh4hI5zFhJiKiItKgwgMBbhvF+/Pz8eRv+bF/Pnz8P
AKGhoVi0aBGCgoKQn5+ePr12+8YuPGbjbC0tNTyKyEi0n1MmImISPShQVFWH58uXYvXs3MjIyy4O7uj
uDgYHTt2hUAcO3aNcybbNw+XL19G06ZNMXLkSIwePVr+LURNRQ+Tv7w9/f/9S+x0cHEpVjm3SpA
nmzJmDOXPmaCg6iJJ6jPcwExGWLyJbZv3465c+di+di165daN26NNcawNmmGYOkpCSkpaXB398fzs7
O2L59OyNZMoSlS5di+/zcHSPvNwgevQwN+ySvvAAAmD59OrZu3Yrz588j
Li4OBgYGmDNnjrJi7Z07d7B27Vq8B27Vq8+eabWo6ciIiIiOoCR5iJiJiIiIqZjUqVAeiqRsRsmzn6N
xYYGBjA3d0dp0+fRrdu3dp0+fPRukxaPBGq5LqpNjY2AID79+9+nrp8eKL5NLvBNX5cDkxL
u3LyQSCcRiMZYuXQpHRc0c8ePCg1LqpNjY2AID79+9+nrp8eKL5NLvBNX5
6igkzEVGJmJgYmJmZKd72bp1K0JCQrBp0ybk5+fDwMBAbhoseKpEwavmKqNVHfUqO4
iTQtSyyComSb72EiItJlTJiJJCgs88+Q0REBLy0YwKrdQV1q1Wz4UYCIqHFiwkxEVS
ONE2djYuNrPq1AIyMzMrX7gRFqgUAgq22lpOVqMRjN4UYCIqHFiwkxEBODixYsoKipCp06dvP
Z37twZf//N5o3b47k5GSVxxISEpNDXL8sW+AgfEoQ
l1j+9efMmnJyc0K1bNN5w5cwZWVlaVlSRkJFq1alWjWj+5eJiIiIqP5iwkxEBODixYsoKipCp06dvPQnB
iRMnEBcXhx9//BB9hGkZEYO3YsPjzpTWPnDtBowbN07RMRERERFRHeGUb
CIiAGKxGCtWrMGtMCPP/6IQXzHESjKmCAFi3bh3mzZtGKdjTJZYs7NCxF/7RVr7YGmG5cImWuEmjT5
O0GzgREREREkzEREJczNzTF79mAd9zAwOtfvy/8Q
wExERERERanBhJmIiIiIiIhIDSbMRERERERERkYxpRkMmImIiIiIiJSgwkzEERERERERERERERkRpMmImI

iIiIiIjUYMJMRERERERERpIaetgN4VkJCAvr06VNq/9y5c/H2229jxowZ2LFjh8pjtra2+Pvvv
zUVIhERERERETUC9S5hvnHjBgwNDXHo0CGIRCLlflNTU+Xj48ePx/vvv698TCKRaDxOIiIiIi
Ii0m31LmG+efMmWrVqBRsbm1KPyeVyREdHIzAwENbW1lqIjoiIiIiIiIiBqLencP840bN+Dq6qr
2sbi4OBQUFMDFxUXDURERERERERVFjUy9HmK2trTF8+HDExcXByckJgYGBePHFF3Hz5k2IRCJs
2LABf//9N8RiMXr16oUpU6Yop2xXl55evbt2QFQukUgE4altvoeJiIiIiGpXvUqYCwsLERcXB
yMjI0ybNg3Gxsb4448/MGbMGKxfvx63bt2CWCxGixYtsGrVKty5cwffffcdbt68iQ0bNkAsrl
7CIBaLYGFhUsuvhqhuZYlFUJRs8z1MRERERFT76lXCbGBggFOnTkFPTw8GBgYAgI4dOyImJgb
h4eFYu3YtPvzwQ5iZmQEA2rZtC2trawbNgyXLl1C586dq/W8CoWAzMzcWnsdRJqgUAgq22lp
OVqMRjN4UYCIiIiINKleJcwAYGxsXGpf27Zt8e+//8e+e+//0IkEimT5acfA4AHDx5UO2EGAJlMUXEjo
npEEASVbb6HiYiIiIhqV7266fH69evw9PTE6dOnVfZfvnwZrq6u+PTTTzF69GiVx/P27GiVxy5dugQAZR
YKIyIiIiIiIqqOepUwt23bFm3atdMFXX32F06+dPIyYmBvPnz8fFXX32F06dPIyYmBvPnz8f58+cxfvx4DBo0CMePH8fKlSt
x9+5dHDt2DDNnzsSgQYNYNYOZuIiIiIiIhqVb2aki0Wi7Fq1SosWrQIU6ZMQWZmJtq3b4/169fD
zc0Nbm5uWLp0KVatWoVVq1bB1NQUgwcPpxpQpU7QdOhERERERREemYepUwA4ClpSW+/fbbMh/v3
78/+vfvr8GIiIiIiIqDGqV1OyiYiIiIiIiOoLJsxERERERERERRREaj BhJiIiokoTBAGQy578XJ
ALeVK0ylJ3REREuqLe3cNMRERE9ZM8NQH5x9ZByMt4srMoD7m750Js3QrSXgGQWLbQXoBERES
1jCPMREREVCF5agJy/5gHRUqs2scVKbHI/WMe5KkJGo6MiIio7jBhJiIionIJgoD8Y+uAwtzy
GxbmFo9Ac3o2ERHpCE7JbiQU6YnI/+8XAIC05wiIm9prOSIiImooFMkxZY4sl2qbEgtFym1Ib
FzqOCoiIqK6xxHmRiI/cjPk8Zchj7+M/MhftR0OERHVc4JCAXnqPRRe04r8fzZU6VhZ3Nk6ik
p37Nq1C35+fujUqRMGDhyI/fv3V+q4P//8E25uboiPj6/jCInqHxYdJG3gCHMjoUi7/9Q27y8
jIiJVirxMKJJjIE+KgTw5BvKUaov1rnEgpyajk63bJ7927MnDkTISEh8PX1xZ49ezB16lTY
2dnB09OzzOMSEhLw1VdfaTBSovqDRQdJW5gwExERNTKCXAbbwFo7vFi
l0jCAKWLl2KUaNGYdSoUQCAiRMn4uzZs4iKiiozYVYoFoFPjss8/QoUMHnDhxQpMhE2nd46KDZd
VReFx00Pi1WUyaqdYxYSYiItJhgiBAyEl9MnKcHAPzFwziVaY21Tc+5a52du6G7ffs2EhISMHj
wYJX94eHh5R63f//hHh5R63atUqFBUVISgoiAkzNSpVLTpo/MaXEIlEmgOGgUmzERRDpEKCqAPCW2ODFO
joE8+TaE3PQqn0dkYgmJrQskNi4QW7dGQeQvUDy8U+FxYutWjQ/FxYutWEFu3rkbkjUNcXBwAIDc3F6NHj8YVz8AKyEmxOSRPVJDO
8bVq1fh4OCACRMmoE+fPmqPuXjxIn766Ses27YNoyWSPtYdJC0jQkzERFRAyUICggZCggZSS
pTqxWp8YCgqNqJJAaQWDtDbOPyJEk2sVBpIvIdW+6USAgISAiCgoI
QHByMAwcOIDAwEOvXr0ePHj1U2ufm5IOBjBwcFwdnau1YRZT4+1X6l+E4oKkH+xcgXxHlPc
OQfD5m3qKCJqjJgwExERNRBCfjbkbefml59u+JpimqIzO0geTo5tmwBkbj8rwQSyxYwfm0W8
o+tUzvaw6I7laOvrw8AGD16NAINxYMGQIAcHd3x9WrV9UmzHPdUmzHPnzoWzszPefffdWo1DLBbBbwoL3ml
P9I8/LRm70aeTciEJezDkIssIqHa+PQr63qVYxYSYiIqqHBIUcitT7yikWFYTbYLQ+EAExwCQqMkyw7NfvC0oW
qfbbt2+HgYGBshiYNAAwaNAivvvfYavv7662rFoVAIhFuj3JoIyEgHUexEhN4+Ddn9a
4BCXu1zFcEAaWm6U6mfyb/2MWEmIiKqBxS56S6Pb7vuGRZpyqOEgtjkRVgQ6fYMnqU6zLT6v
hUe1NvRSIRIHny9UFkaMz7Baugffv2MDExwYZULF+Dl5aXcf/PBgQGZWfL1y4gM8
++wxr1qyBi0vN1pGwqjGW9C+wGH8zv6TZ6yNdtSSbIRQfm0EoRgakLABeD9pTicKFkU4h4YRqKRYQ-xyzjz
4uEdyJNvK0eQhexHVT6PyMgMEltXiG1aFyfJ1q0g0pfWQcRUW6RSKQICAhAWFgZbW1t4eHhg
969OH78OCIiICXy5GamgpTU1NIpVI4OTmpHP/gQfEsg+bNm8KLQYoWgRBgCI1HrLY05
DFnYUi9V7FBxmaQOLYBYqkWxAykytszqKDVBeYMBM1QIIgqCwJIxTkQp4UDbGNC6dEEtUzgiB
AyEpRLcz16G7VpxyK9SBu4ShGRXxxBxeS5CUlAQXFxeEhobCx8HH
6Nu3L+bPn4+hQ4dqO1SiKOwNZ7JlKrfcuMrGAnnNX6Dk/B4m9G0RiSYXrMANg0
UGqMyJBEGpn/kMDJpcrkJqqO/c6qJO9+VPl6IWoiRWaDF+s5YiouuSpCY226I61tam2Q6h1ut
7/KNITkf/fLwAAac8REDe113JEdU8ozCte1ikpGvLk21Akx0DIz6ryeUSm1k8lx60htnKESKJ
fBxFXXWP8TNG1/kfX+x7SHkEhg/z+dchiz0B251yllrUTmdtC3/k56LXygtjaWe1tJI31+4+u
9T0NEUeYiRqQiq6wKlJikfvHPBi/NksnPzSo4cmP3Ax5/OWCqliOqXYKggCItEfLk6
OJ7j5NuQ5GWgCrfi6dnCMnjadU2LhDbtIbY2LxOYiYiqm2CrACye5chizsD2Z3zlareL7Zygl
6r56Dn/BzEFs0rHBlm0UHSFibMjQCn7+oGQRCQf2xdxR9ChbnIP7YOxm98yd8vaZ0i7f5T2wl
ajKR2KPIyoXjqvmN5cixQlFfl84gtmpckxiUJskULiMRcE5eIGg6hIAeyO+chizsL2b1LgLyi
IoUiSOzalCTJXSE2ta7yc7LoIGkDE2aiWibIZSi6+t+KG6oq8s8dz+k8wRBsGdSfTEEmRQmuGxVqZOLqCgmM88QBJDbhKSfuz
L+ivAiCXKa6v+RnQS4DEVbsARZFyW5GVonYakjqKlFgoUm7zQ4SoBgS5DIrUe0+teRxTqa
IzpRiaqNx3LLFuBZEhlwkhooZHkZtenCDHoH8/nVAqKAWg1gCSYsOxckO3ly5gw1SEyYdVh
jmb6rkpiW/F2TxLTsNk/tf/a5lNuP9xfVaA3B2iCLO8uEmaiSBEGAkJOqOqWjJqVo4zFj
bNVStTCXmQ1nexBRg1Xl5Z/0DKHXshP0WnlBz9EDIgzNjjjcRJVFeYMOuoupi+W3uJqbzyCWsDS
EzrK6GAxVyIyiLICiBPiSu577hk9LgShWmeJTKxVN57LLZ1gaSZE0R6hrUfMBGRhiiXfyqpbF

3Z5Z/0nDyh7/wcJA4dINIzqPtAiTSECbOOUiTHVGn6bu72L4vvCSkzMS1JiqnB4JRPomKCIED
ISHpy33FSTPEXQEFRtRNJ9CGxbvVkzWMbF4ibWNZN0EREGlRbyz8R6SImzDpKdudcldpX6uoh
PSESAWJ9QCIpXuZFrAdI9CGSSEr260Ek0SvZr1fcpuRnkaS47ZNtPUCsr9wWlRwDif5T23pQZ
CSh4J+ISoeo59y17l4/UT0mFORAnny75E9xkoxqzLgQmduWJMatIbFxhdjKofj/JBGRDqir5Z
+IdA0/+XWU7kzHFSkTxieJ6eNktIzE9Ok2En1ALHkqYS1Jap9JRkUl53qSsOo/k7w+c24tXEU
V7Nuh6PqxSs0cEFu3gti6tQaiItIuQSGHIi3hyX3HyTFQpCdW/UT6RsWJse1TyzpJufYlEekW
TSz/RKRrmDDrqGpNx1VJEitIRiuTVKpJWEUlo7JPEtaSUdoyE1ZO73lMJBJB2iug3EJuAAADY
0h7BfADrYpOnjyJDz74QO1jDg4OOHz4MGbMmIEdO3aoPGZra4u///5bEyESiiu0FifGt4uT5J
RYQFZQtZOIRBBbOChHj8W2LhA3tedICRHpJKEgB7K7F4pHkquy/JPzc9BrVb3ln4h0CRNmHaX
n5InC83sr3d7o9c+hZ+tahxFRbZBYtoDxa7OQf2yd2pFmLhVWfZ6envj3339V9t28eRNjx47F
+PHjAQA3btzA+PHj8f777yvbSCS8qFOWmq4BL8iLoHh4R6VytZD9qMpxiIzMnqx5bOsCSTNni
AyMqnweIqKGonrLP7UvTpK5/BORCibMOkps4wKxdatKT9/l8kMNh8SyBYzf+BI5m6Y8WV9b3w
jGA4Mhtm7NkeVqMjAwgLX1k6voRUVFmD9/Pvr164e3334bcrkc0dHRCAwMVGlH6lV1DXhBECB
kPXxSmCs5BoqHd6tebFAsgbiZk7Iol8TGBSLTZvx/QUQ6j8s/EdUNJsw6itN3dZvo/9u797io
63x/4K8ZcLhfhnG4CILcxjuG4a12V9PSzcvlz6rJ6v9yxtuGNmN5Qsv5HiN0PzcmmBJK0Szrn1mf2mZ1Ah6mXkAx7IW2Gn2kIm0R5Y+RUdEAMGnhy9jv7SqS0uCOm9ZTYTvHvJ5xnfXKFFD0ET6xZ0pNpb+7sTcVLUoKGEnd8a7ZjOGJQTQQGN+zVZJVUnKuQiqcVoRNz16sTKYqG9SChtWLyWcNvNW9IFiKbKm45pBZZJ2yUhTsklmGsYQkEgW6BcnKDzglV0h9TZNDaZk2JVl2hd9BZT9lfKFDzR8GYbLm8m5jGpiHsN8MDc0qv4oUK8qqJXtuM/TxC+ZDBDS97z3dF8cKM+MEt1ZcxjnT7ssNOOWcSQxwA+XC8DX2HlNGxjyDWPNw8Mdx/JGhyI6xHD2IMyFtK7m1DMWn9SSkEo0y69Q9vJUxsYYAB5ojkxzKsbC5sx+OTHK3pGR4ss+d1xt8O9mU2OQ0QUjBSsZqFsXhZ9DWGdpLPDBkdVLKJYyzWH4z+fQAAAAAElFTkSuQmCC

x1o4+bEuPBGZxyI9zA8//DBefvllnDhxAtHR0ejbt2+rYx566KF2z6NSqRAdHY3k5GS89NJLk
Mvl+Oijj/Djjz/i73//O/r27Ys9e/Zg7dq1WLp0Kc6fP4+0tDQkJydb4mUQERERkZ1z8guG+0
MvomZfIkTtzaaNfdzgPm0VpMoI9iwTUYdYJGH+y1/+AgA4dOhQm/slEolZCbNUKkVKSgpef/1
1JCYmQqPRYMiQIdi7d69x4bA9e/Zgw4YNmDlzJpRKJVavXo2ZM2da4mUQERERkQOQSCSA089f
cyUu7nDyj7RiRERkryySMB89etQSpEA+Pn54ZVXXrnt/piYGHz44YcWux4RERF1jFTeD/rqs
luPObS1Mw4ePIhdu3bhypUrCA0NxfLly/Hggw+2eWx2djZee+jDt3DlKpFKNGjcJzzzz2Hfv
369XDURES9j0XmMAcHBxv/+fn5QSaTvd/f32Q7ERERObQbXcfPhFDIMTiHD4DpunrXDsTuHDh3
C888/j7lz5+Kzzz7D1KlTsWLFCpw9e7bVsRUVFiyZAk8PDywb98GxUVFi6dGmXSmoS
EZF5LNLDDACnTp3Ca6+9hvT0dAghADT1Bj/77LMYO3aspS5DREZpS5DREREVib1DYL71FXWDsMuCSGwd
etWLFq0CIsWLQIAPP300zhz5gx++OEHxMbGmhz/5Zdfora2Fn/5y1/g4uICAHjttdcwfvx4nD
lzBuPGjevx10BkTXqDMPb46Q3CqrFQ72CRhPnPnz/ji1/8IiZPnow///nPxp7mI0eOYMWKFX
pUrz33nutPgCIiIiIepu8vDwUFRVhxowZWJJtttTU1PbPH7cuHHYvn27MVlu6ebNTm90SI5Etq6lt
gJfxcSN8rBoN9QYWSZjfeOMNLxMXFITU1FOTsbty5cvR3x8PEpciIiIislv5+
fkAAK1Wi/j4eeFy8eBEjR47E6tWrMWXLllblhwwZgiFDhpge27bbxMnXFxcMGrUqC7F
sikUC0eOzor7e30BsEIGl+bODflbqdRRLm9PR0bN682SRZBppWvV6wYAHWrFljicsQERER2bX
q6moAwJo1a7B8+XKsWrUKX3/9NZ5++mns27cPMHz+ZxSyHxf+8o6m2mq8dY/rQcNWl4yOqZajDbyIyxHfsKxVvY6VZ+3aRHv9eREBVc5y
2IOHjwYFy9evGPC3Dz3eceOHiLsVhMAhoNNouncPWGVrMbzUYBCoqaqwYDXUXR/
+78oaA9VkkYR45ciRSUlJw7733wsPj5z9qdXU1du3ahbi4OEtchoiIIGzAtbIa7P8yGwAw//5
oBCn4hc5cgYGBAACVSmWyPSoClT999VWbz2Soq5PlFSsrmn39ZnFXX30FnU53x7rKRERERZF8+O
h40QwuFfb2/Fvyt1N4skzCtXrsSsWbMwZcoUX/hL3X//Cvp4EleB2RHMiBhIN/GoKEbH7YWRCZJEF9RSVPrbIbu/nGTYVjY0EoBrJsIkCjZYX6ua
JqNn9TlAIC/Hc3Bsw+PsHJE9mPIkSCHw8PDAuXPnTDoUsrKycNdddwEAKDL2p3W9CoI5BjwFTMaQm60N8zV+xpUIClDRAVe4XcPDuQJZCcAllanzuwXfkDCXBQiSXt3LW/8cW1fb+RxcnPmyzdUmt1z2R4t+ZPr2R8XbUEcoZwrAC3yETBqTQ6fibOB9vnkSo66IdlVOitsirbjgW9vVsFvEbL8rGpPnsXEcFnrrJWmRqXtmyb9oP8s12xwrO9Y5RZ+/hGgN+8Ud4tDpwK4tNZQ7B3zmckYdUNaOykzVwE8NDJ59nDANWlg5AlGnV1EscJwbW/OIFj06Dagz6mDZUWxhvALF+q3HjC7cn3eJMvtjEjZ0EXL9K8CD6TGG2OxzQSiSAoadRO0E5v1PlpsOAm07ZWjk55jxWyZpXy6E/6dk+Z9oFfsfQkBX7NIEBnvJZ9Z/7qlBnPSfEP5VNkbItUFrE9ocHN1BOWX35fGWG+k2UjrIZDdKh6nJZVKYFeyBRSaIZ2mlnADUVKxhU/Gu98hH3GpqLwwMHXA1krWIrqa73R+/5cXjLnohRMXgKsk0/PZzx05BWEzGTj2NkaQEXdzQU2AXBh0k/Y3ub4U7GXrURQSBRZJFUWBFpIzI0a5SRqMzr9G8QQiNBDn+JbzSFlmaQ4zwVQfTcnjbQrZkiaTJC7+YCNeXBEhIBsU5pOkvpzadi2jlG5XzUkpqU0YNkuq6+R/HFTnSqJh0e2tEVS3+iCy0PgvE6yTiTy17STb1OH/pUDcK1GuwJicYAZqE13DMa0XgzVSGlFqJaRSBfqNjOFDB8CNHuJk2jc+EpgGU71BytAdAzNSNHlbalZ/eFm7bNbX7M/o2NTM8R7UMgtbxJiQxz8iuPfpGrmGJ0oJRhJ7Vb5mxOPs8nI6ojHpk5KRSP8y2Zi2ZqiqtL43xZF5gW/OqwHTiM0yuEO5Jz9z24jYI9iwrVMK4fzYx/23XbLB4Ey3/8dg72AZjUfLIRHFTUf1qJs/lC24FL1QzHKGldmMIPGUYgYZPu1W5Xs5cJS7Epb9d/MO2xSGMDY3nm1MGUhXAXPJVbwrnsZR2MPowXZ1Mjm1ISqRHWDZ5C4qqcGwjINZRIRqZbdvQNkKjSULQyJBVGMBbBH+SOBsDL+WEGb3nFOdJOGZqTIUhKzAQ3rhRsxxv6jFijtGwkdlIF3Gdp1Vf0qdE+6vHWLMoqaKOBu7BPeVG7fc8XbS0Emh4Q0Qq2tXaF8dTM2xB/RdFPT0u/ofxhyZcddqKAzvF3zUkoa/gh2QrIpKaSNEp1KxhUjLFU1YdA0cVV2NsJYz2jl1aZpC1Ujdc4VPoHgjqPE7Wmyz8ed1sJ8KPBlTsWf7KPwmX0Axx1Gc5vSAT9iVpHcXTNEy/3ePgnHK8OVhWSAWzEttaYAP9w/F4UiO4aeRgUabfP/k6m97bzk1sSIeJx+Qvwd0O8aMXnpvqHsKbSnuFq0BOBrwoS7AKJodHxUbygWaRjmB3yQY/oHxAQ9+6XVbh3wxZ9dB4i/pnjhD4QBs5k8kSN0F+cPJSL0G5k/xMNwGOSWAUYy+bE5dYuE4S/IQDOcV2Ze4MSt28exgDqWHQLdFpAEHxPFAWP6LvhYU5cqjGB/V6kzXfnqk/iuLF91NZm+gZtzUeNjpgK+dwLsW4Zo6l3r0r4nXzLGXv4ntLcKvmixVm2Mr8RqIZBxtyTVp3Rm+3ZitwxTOnqGMyqoRzz2n/cNG4R46KuCnyDqDTi3oeaGc0KvzsRzkiVDVBp3wDmzVCM+2lLXGHqnMEtScgAQF8yjETZi4dC6v0kE5hRAS3PXEmw8QqnIzwLrvXRLgYqm9cDGYDqNwsFsDPJ1Y1lYDEaJfJymDbbmG8TgwUfqOiT7jnV/D8jdDdMRhxjNtC4BqMq1VzTqNSXjkRwKsJSLPp7BMf+H0NCbKnlZbYcHkfOc1eUYG5EdoRvsqjNd0VKkKl+u58XXyPT2bTaWLvEvBxcAR2DVxhbzDVT09Q4jaI5kxcgbcMh3CJuBvbxCMMyK8vqEedK6jS46yW6Ewh9l9Dk8nnzWA0nd8eUjvnuWg+kxYKbPyeidMUwmyjTgNEGPAiR00HaQKo2x44FhCoEoFZYFDQLdxNi6oiGwm7aVWSyWa+QqN5kA4LxY5j6ZzGQHt9+UR/Ozxn7K6IA5SvKXM4dg88kcqN+8AlVwOs/pfUbexRH40PsYUA0EuqtgSNgThKm/gcRYj0mhHqOcMK1MjmKABlc0x3xpHtASfLOjLIktVUNn/7N5A4VxnCW2gwnzTaqJ3Bi2u6zzLONUKhR/VEa8iAm0s/bLrbfc+t5SfDXyM5kxUQrxdRcp+GDtB7HEG7tRzmX2RrIJRPffdxqzuISsmDRRPrPTFh84GZ03yhGGNaVgGnR4RTE9rgR2qhx1ZFl4sgqKw1yCvWWiB6xSkYeNs3hGUAmxEVCjK5yZuU3oMhqJ6P4nWYQJeMDcU06WsBZ6lQbZ4hZ6fLdJa9tfxPS9aVlRV5u7bARfFxTxrzeb1tXrWNhPrTHzl2eHDT05c+gcgm0JFmyROgRYT9UtFBBl4cq3E+Cy/qhPRzGjRHzCrTE9RSXVcWuqLM0CgHCcPEKMFv4C/Qrs3x/kJE4bbtjO4udYhQTmmYGYgySpY0gV+nq0uPK4FkLV0eUOeKPM7h6+HPFj2eiQTbl/gg+2PFtXqB3Ywwv6eYI8eQjORwr+W5RY+E6SgWfTqjHXmCJpv5dQVzrS1eUOsWBsyRPK9p4E4vSbwR/+uJXgN9/sUJ2bG7BmAe+w+ePlS1Q+7Nh8GkyHMKrpGGi+Mj3XjuktoJhj+nmTzohy4/LETv/xDb2+LGjO7/L3+ABGBzGiwmVjX2yN2XKydqjMwFk9x0bvzQgWXjGrG0jxDObOJ9CMwMwWdJD1hXmRKIBi/Y+E5TJdqXmwvB6I90nR2wbgaFdeMtlFb3CgeG/ZEvxtdJdhQENXPkQAcuHM9LB3XDk4FHT4eVzaaXb+xM//MsYoLBsYgRB6YjPd8TQVOTU/AmT3A55K5+J/HIvDoyUfK6dlAhkZmS/mEtvCBDyvcjoS47qp3/lbZx1S55wU4KAPTR2n31hBc/0Aoz97DFQoqPkk0G/gqvqsSiPDyc+TJBWQIhMgYVx3F5t0uEfjFZUsnHOa+tZ2BpthxA/W3eHORDl1/4wz4T1tyqMgvt+zV+iv38YTWlFQLwYBzu9zaPxtXzwOs8uDMG2XVQ+gMkbFBKSjAakjZTOo7JFIEX1+ZF/KzvmgbHcGPMPE5fX6CmjbfOjkJQqTb4yYGrFbExIbcmZzbFkPwBIX0DP1A9hzLEjXxB3hxCoLDIhKZ8zmCVDGtxxbLtdJcpQuoCCqMaMu0rvQj2CoM3QPC3NEAQgYC0UfW6Od9GhYCvKVwAw4jYAtwP1+wAuZT/58Q8f8Pv8E3bB9J0+QaV/yzXpZkKoc0tVDF1rCNgcMyw9SdQwBezFSvbvkjK/J6kgm+zVfo0MRWBbdI2U9VLp3QBX9jlsYxOOYqL2QDBHFIrKQwRCO4rVbF9DVx1Qsv3Q1PQSmvL1g8PsRgIVZjNoM2d9m3w8xjXR8ZXEUJH2FKhDfdvtKMU0C7G0qVCUwHwSgRz1vjbV3zFZ6VlF8CpUj8CKYWPVS9DaygJC0xA1+OmAAdTZoSqdC6jjK0OlrfZDX/4zwD7d3Sn/yCQG9DUh2B2OMDXW36lP6YWmUKiWoHjt2dWG2o7DtYw9HAJHc+Xdl+K2gXpb8ODbo+dLnjoxJl+HO/jExHXMbpWrU8kGOQ7Xpz1y9I+7LdH2Ju0+2a2Kpg7j0oN5nsDQ2MOlQJvoNu+GYPD+ARnqKLHb1mgFuQkAYmcm9O76NyPmCuAXc6dnpgsaSfNtJ0DG/TKZOIVRgt6H5fxZkNB2HpWzFS2whWwKxHmgdTC+wNIc0c/2k67dgGvoW0Pv9NQvCaIYPAD05i6jt0XSiGtOlcwC9r/NBSYz8Xnau2pCvs5TcQEtbn5xqlSMHq85IYXrr0AGbhfE4DDqlSXQuQAmOWIwsgJV7R3W9ma8Y3z66m8GLT8rkv+4LgRA4KoCIySWEdtd8EPZ4hEaBjq8ZL3WbCAkDG8kDvR9hlyxkC5uO8yWX7+bB7vQABe0c6rXtLvTL6+zCfBPGb82bGT04v7+xTBDwTDmAV/eZfT9uBuhhHmpFZy7xthAX1FiY4jNtKjkYMK5lxmxIXmSV3DAj46VVG6FT/vN1O6CcZHjGbpTw70yYkJ4lQI9xlp2vf68LNsHB0CakTMwM3JDuH0GG+YvZ0MhH3Ov6A+B7mkhS4RPsjZLK6OyXQhiDKeXEe5EPRPSxRYCtrXMGvfwqgEyeRBfuUWkl8nNEH2nYMSb9d3qScf8E0bPDNwhP9UJUbIuImHDZ/TO8rJAQExRdCfxjOo+nSIuIwBUIOk4mgqMbrZcXPyY1BKzUsQDHE4lBNFL58IjmVvuJkDeM1CfEIiTB7zQBLL/bRxWsgzUXIUcxwwfF/cQdxx6YRGGzK07DWmnvlWLRYD0dBfBKnY6c95BWVOwIuwQ5WDOXtHlIzy/gAvv8pKGhkPi+XrD3tkoqDIA/Wvtkz5QzeP7Z+wFSQvS+8BgJZuUZsVKTYLNEffcvzY9kcjY6bMnzK/AAmOzjE6hbZGArKKmJBp+zPWPOkvLGK1nJrtkV6rIzW9ee9cgXBuXV1pe8/DVE/RLDY1pKaOFSPG5JdYyAp5gTjJoBp4zG4Hmb0GuAVjBH97SHdl7q6wSYMMjmN8E+tGE3xVkTJK/PWnnDoZpT9+3KF4I9dOIXCNbVNj4sGSmR1PLEkw3HzHYm8EaDsGnmzMR4y0tHqWYrVwTOQ/HE+MyfbTmYTJp8ZAFnljmWGtZI5/8S3nmzxEhfJjB3GxqS0+ZZlfZ4vFx9HzBgXI3CzPmI4iDRuYjrYaSK5fNZP3cTYBx2b2WTKUEZpDl7YNhtSkqHuXlD+EVKkCmdjcGI8qzT8VzEa9KQgPxpo1hHOrSiFgvUsszyVzbmb7dpUvFYgFXbqpc3rXjsC3bTr5EzbqtNjZlCKvaY3J2EkP+NpP1u5lWizIL5dhSaIPLJFFvs+UbjM0IWK8lfG2PLvVk1BSHSmE9blsKx6uD+t9rKiPfENV9U1JFOuDUhq8JrTXYrHGJT0qzk5nkpG7/R2m3pV1oXeCcJkRFAEXQVcxH4e++Bm8jWr5wvWd3jLHDgLtxJGoPmDl0EsGyD+FwYKjnxwaM5EJYhqHR2Vl/5AVgFNZtv9b54MArSfNJQrmD1wE/5eDJvCk+X/AoVDj7QwM+IKGdjXyCSZnPJVD0Rb1dHItxKfGQVjtDHSPqvZA3TXUJpVSBDPv7r+yZEntoDX3dwDBNvNgiv5U0/XPePI/FZpO82kRKZaJaHeUWoPE+xFuXyAtrxH3PEtM8KJ/Wj5oCk6uzJIXCYZqHH6ZMzx4xOhR5BIcE2lHbP3D2PxE/ipQ+U+BazPs5BDxyjubgxYUbsmZZY0a45kXzOLQcX+6LvRPAULxtxSUZgiOmDp6Ixzr+NxEF6Im4KTt2XI7y3nvVevVvqbvwsEmk7UbfL7wvxzo6UfncSIE5BGM68pFj4u/xbGl08ueHlLgKLUn5ri4m8lxA5i/JzmO/t5UM6HtnlKD6Lb6CkADnYh3pyzKNlTqhY8rbjPGE69jJRQrbgRVSWUQ0BgoRNyxHfqjn1N6QR2v/ux+++fFNSk6yx8mAAbS+nDtTyQjIcUpDYiyMjh5qxs2jmGcOkoRiT6MeJ4DmYnAr4vNS3/4KPmG6QS7b0HDNB8R2i2/XWyd3MoFl3IHjr1Y42SQnGdVhOjijuaVzIQeVU6+UJWd/GpRPHjbUY9HYaaOFOsRAh6QD77n0zmEOczmlBLZD5xvCaNePHDSRdHUiUbTJyY0e+I43UBVqIx+DM6PDZ7P7oOzD8WIm2I7InT3TuUUBuLXRYkmVEbgHNjoO79GUF3I1rYX7UwA7TXYpcqI0PIeFzKC5NQyQnXPmNmk+Ln4UOV9uiF48SRV0KMHKuR9uXI4tkI9gH9SgJgPsymcEzT2tyVCgDYo0PRHkkF0Aae1rfrWzUiXlr1Pe1+4p9FYUfSs6hTzJuVGQYA5PeH9SkyvrKvG4b+r/C3UTwIjQtU2MhLUnz+P8fcQr2oJLDEcgGD8y0ofPxjhEPGeYHsMzK2tSqvPBUCotYQEaDuLOtEHlfh8zCPNyOxqU2WVqrj8iDK9isBP6APJWWsrd+dJftJmgtvHnIkUzmdMP2h+wbDxA0q/QRLYiIJfvXWIVjXyb6ztSwaNBjBF6y5HdyD6c0tNQWM3eYqTFZ+lSW8GgM4NjQUOk2njT/Er8FqK+DP+oUVrNMMDvwaCaGpxWJM7eFrK3B8QMBjJSG7pYyJ1DYTpY/G/PEKhaDSMb9xPHTUFHR0mJTygLQbovsgrL5q3dC8jwXgeFtMvo2F1MT8F6Y3SVEr1N0gSkctHe6Pr4Wcyxzb2XHbUUGuU/XWy4dBj9wuFYVlr0zSSmV1d1zq4Lgp+r9oaK6dd/4g3ukPfwTXHLx7AA5Kk/wIzbc7q3y5ex6pR6KEYPQkVvDCZ3M0cr1J0RuGk6+iUk2B4+fdGUbaFo+AL4SqymetEk6aQD/Y+mbnIvsrs8c8Xv78SUsLwaZ+5bBzGNSvKtlfL3h7zUwwVhxOebFh9RtIK4wqlAzFlYhp5Ub83J2XEbnO7fcEZ6DHVVz3qA0IZvMRSFzkZSnOxvXEnzMHy7rUMaRKpOfdrCjcXK0yIWlB5gi5xjKxYySqg99xk34E4m8JcRqBfpbCGvA9tPD2cPFbedc2HeXHJk6ldyUzZJRcg/6vNJ3dB4uN00PSQkK0ABDI0bw5hzYkPIm0jtJvVAKeX4BMcj0uGbGqGmIAdy7YsJUNDcAACrZdkPrWeDGSLhPO5Gb6vDU7+NMWV6pXgXyDpbqVRF9mgqoBZiA3ZtTEjjLjb9HFiqWDaEBdr/bFEDeiVwD7l+7fbtpzq7KD3A4YMbY1MG1s9Zx/7GEjwhRcPVFpdg5AuI0r/i9D9MXJRXzT8E9vwhyeOdS0g+2+oHd/u2czEbQNXn+ymbpZ0eywvFePUTCwPzNlCRF/5lvvhx+kS3gPchOh2wAGDSjQO6krnLQjeK/kNVUNiR7fr7n4Yq5RFsp5DuR0wtQhnJWIKQ2Gnsi3z99hJfAGRJBvXvKUZu5s/oKqXprXaNqmm2FmYVgiuhBKvWEZsUrlnYAvpzXlfepfuqnwGHYaWAnGsHRPJn9GOl70vyuKDvWZaK6LeFBiGtRfqlpQ3CEJiuS43ejEYaTzXpR64XsAnVTVKDJShzyf7CUO4SjBPDhJ09RiQEr0gzZ2s9Rr8FMiHS0F6qJ6jKzHHNR6uH/Tbza/AfdKsq30rwc7p4ZHX+vohb3Fu+yfoEj8pwoUs5pyFPStDz3u77jX+rMVPn0vx4j7jCUHEC6e8Xg2DQo7Xm2XTqFwl2fLufAdzaIxHTzVrGvwqUTN4L3VNPRwHK4hkKizIIe+AS9/R+vChjFJvYhG5qMx0gTeO0tLdlyLCJihJw+PHjMCUVGGSwK5VzGE3ABgYNz4iqUNRnYt3ThQdhITuYQdgd0AEY3EJdGsV4uW54OpNK5RtRKEImBbNdQ+1KpcFdU8+hOJAkFwz5sIKXOtzpU27QRmzmQq2e+ZWK3Gkxb+9wWxvDD/Y0K2CeS3M6umk8Cc+qlZ/wfnuXbvTGg1nZSgt5o8evSqpMHwJuGjbUKy1Qro+BNBwqNqnlVbyITH8kWDWyfhRSKIQMc3mH/WejmBJp7jrPUtWVdIeNaPsvMGjFcqMxmNXzGcE8VC8nkOhjCvfszWPF+zWwCQKsodGJ2hQWkqRDLWDjmoohcSSfRdoGfbiiZdCQYkaVzdoC64TfwxKnaQUJNIuxHBkjijzjFpIugvfMpvxJYlkEHxV8A6JmSQHPPxCV1fVJ+/x0ozNlQcHszpd/s6lHMAhB6Ee6hQMtWSL+Il12FWo4H6GVvkNjZg07pnxrbe+Yl3h5Bl2D0X3Bh+TUAfVgzK7JaWxPWgjEXKwmW1TNYL/VGS3QlOkhmzwwsMSEIOLrKhAzbx8bWmgm0QFgqvGGUoyDe8jLzbnnmDm1Dt4tVSzBxZ8aCdjmj+gwJrhjT4yvnDSchq3hbrWYQMFx5ynD4bbZpvKlnn+0m4NLHwXhVyi1wuMqsJvtI0c8e8mV6Cf0wyC9jr3x2vQ7i6jV+xqs6ri+GGH4TXrUtK5zFD8vSFq9mkc1Xbe2bUcljYGAMg5GMXjqS4mQVfjovIDVpXzDD0dKWYPy9VW+TZFhk9HfdOcGmVuU1IyC0p8Q3wNH+9iPO+cBwM1wqxPNPnlSzRyyq9kTaUpNFFHVXtWmVpuRAX0FXTSVqzz7aalzlxBcL/SlXzbspR4mH9vEw8+W0aXPn7GybZ17iwBp8aHMcWdQxJxkEi1AMcvELtR0xoiZE9LxP8PzPjUNhgT1Jq2qbL+rNVz3fIYd7Mmm6KeqETR9Fy1pmMtyRrlaZu2W8vuWe/WGvS+Tt/aDU7j8A73FVvZoMCSr3sdZ/J2Jn2YWnq14x4X2HLGlxMm9wmRBbJaMKA7SCYkrBWvOPtWEthOXUaPbRRwBtTIdTjuyN/QMKz4FKj+F9CQT8H8izK6tywrjp3fy5dFIMWFOYmv06jy2SzS9/m8TzZpF5nEzOY4sO5Q3UizhBJSk8zI4NvPMjYZI3sD/jvjrVDvEIsQhUIawzpV3ppX9vNN52RT2lzHGeJ7T/88XaEzBDvj1nQaWkzb+PZRE9Lcl29d5GnBeajzqWaQTtWyU3Kl00gqDZg4FO+dPq8PKG7nPv0A6U4EuD7HP34Y3kxNHYLHlCXedAG3W7/c9JHrMYVk4YMr+vo4kIkVzOCv9s/pRSxoYNmwvx8Uo4jf0Lz7TqOxsXBxFmaFMXUD5sc4ixsf1dX+aeVUczs23iANp98KjJotQ37K2eEuUm8Yq9QgVMFHb6IJuIyxebSqYF5xgqzQZkzCbprQbTsYyKqhC4zS1fmd6yKvQEaWGmGvKpIZsBaI6Spgs28VZMHN0IIh8hxa3NZ4ZKfDV3m/wF7tJqYqU8xf+dGeRfbsD1qDuSAZTP/9kr3EaGHEngTBuycv3CFMKGyBJP2oBaLJJb+BKTZlzeoqUpSAJdqGtDnDmSQyWKI09UVU3jdTa0MvGUiPFOWfm1QX0cB3nG+rmWIHcWGRvCEhhE1dtkSYtNhvRB7K/dtZpfPmOixLMrERsPxXqVC6CaPmfxwv1yP3mRqnCNpUKpJ2IGaVY9BrX5M5yZMN04ac8D5OLNsFU/CZyrOvCuE7aDKLeGqBsOlnhMGfwdIGEnCyu8uZaRpJ0YJ9yBLjVn8MEBTxMZfjU5/Y9hb1+YlNN1aw4YRZFCjCMa7v3JWs5yIzRkc0zH4nKlTxYHo7yb8g2eRtyMmz80uWqIW8FRzO8CWcw7SZxIQ5ivcU9l1C9wiBJZ4wRK9xPKnyy8W1KYgF76x9o6mrH5fr3VC0i1ZbRcpYnQBJoo1kFPjtNvAcFcyELGazcXsqQT+06s7H3HeZqtk12i47OwfbKdmttYJfAIHIRv5rh6uTjEeERA7FR1ZO6kwO4kGKBzjbK0x9MHdOcW9WhFyO0c3XmJlUWHy3xS4gwZLlz/SGlLJWHxsi36jjR0KuO7oQBPNFVr+YXmdrb6NpcxS3FFe91MGkZdmZlRiHh60Zh2tDcMgVtEDSUkfGTxaC1c6HzP5u7dJ5X7B0OyVPpZLvAh6wkH9HAUXDOhRE7wBpnfQG1pb/Y4Upi3WQ4GmnYcahmfOzOPHaX0bwQcfPjZTufZ8pdWF3fc+MwYbebGMYq0RezajMGAYhEuMCeObtvs4zClrJUU0BEhM4XbOhQnCyM9kUcvc9oKEOnhcXY4B4+T8qzGpUaNoMt3pgWkb8xJ6Q0QzLZwo/O1Py9WspGI8fFp0A5ZMOV5GOEi/5J2Q+RC5YiOqezqYfewc4o1X2JIQNGuLyc3VtdHNLAYNC/LAQN6HZxmVv5qCBVY4N4KSEfZxxGE8iVu6mBH+HxNnPWKSIGhHHvFk0X+CTE/vLWCbBkVaKhRNOrUMjItqy9MhzYJ/uEUFu32zbcbZlVrTI4f8yF53WNKIBFvKCOvjxnU/Ay5WVUKkfM8c8OWTA6iyHx0Ijz8EsP2PQ0eXJGdaZQh2mWzUS+nzkpZ98tqEE3Wn+XwRVWiUNl5XOmtebS5lzxW8SQRR6ZqaTA4ugyPDj+0AgLrHXJL5EW4d+HoWGpqjwqLU20w1n1UAIkM8bZj2fXLqZGiM86X4eS+NPymBqTH/NTEUwBeklfuHXc8Mm7c+P5sNlDiZ9jPClmkfiDSHRzf8yvGbVDhMzRBVY3xtiXpDKSBa7tUMx5FdfzxkN6HSdZWfZ9yYjz6yu9imLjcPMCkyuytNTDl+hS3PnNBECwIkmkadrcFMMo5UEzubDLVyoX9IwNJA9BzLOxPp4DJ1YB+VRAmmOC6zz5HOg5HyS/tHW8YE5OHZ6s2CSHp0Jw1HJz0XEYXgRW9hPfsxzG+A0qdA7p8l2gSqIw4LujbBtHfDpwSddxkijFQTx+kClUYDeobj4U31W3Ym46gl3z4RIwkYLXqE4mn3nxVX0blH0aWOgnY7sflOGG1X8yzmmJS0TSnP9PF3y18Mtjk0TzoBFs9YOsf5qxYQw3SLRWCNl1Zdb4tJIlfpEHK/j/4s8RiEhq7Mmf1GYX4P3mJ9tSJiMYfLwScBzLl7OSVSYQpgc8pXbOAVpZIMpn67tWkpJ5RJlm0Bmt9KFpKkB1nHDdl/3T5rIVxgsXTVtCMp50SuT3q7eUHtgnE0bRkuT2twE0rYHVGGkzRRFBuaS9s+Gqtx7UtoVV9qBvWBTJBNsF8u+tJdh2cjBT7W0zC2xvHhxCzQ9aRn5SD8Fmo7KYTpwWbZlN3pxNXpyDY/JmjyZYExEo1AkgEqYtQe6dl8ZIj4eFhGAKI3dxd9bHkA/kDPwvpNhsSxewVmVQ+JmWJ1cDf4uX0bRXOhC2iYi84ceRjmD4RmRVSDnDahzdbV71qfkEw4jj+wY4WnJvqQzzo2b9WNRmCJj8zB3W5mx5SLo7W5XxYrKrCBM89a0nX4kiJs0CC3kqZDGjdjy8ESC3ezfDjaBZr1HAbQfFGE0DIXr9CFdvm8ZAbBQFEK5bRWhcZkz3OyNEFlOgdN3fADDXaZLgVwNx9SOYSaMGOLyCHkt4bWVg7GDmyYDjqEkbhvC6jaRmf5D8VkPe0bC3udYP9L6x/Krqw8n7kdO5+BUHKEFEdHYm9cMNA1B9SjY0c8tAe2SCVnqa2DxF2Sgwk0LExS4u84Tbef1Iy4GzA4HY3ebmxvbf+JwK8pdrv5TzjDVdWIr9zD3qH5TejkZ6q/qLBVx90mD1GnW1xU31/7Xvn8oi4EdAycD1/ha3GV24Y1nKI/IHJbF3oyxxS5unGGQA3cB3jm26Sn7q6SGB0B5XPxWhrAhdDE/GxsmFUpiWbXYTqrXhpBo8sSBpI/nqJ3y1LLEdYD9rH2+LLIc3UJaC+g2jb3N/9MJa3zMpzNnBrdKRcH7PX4q8fRuiQ6R6zldlbDoIANM+ErbD0XWihqwBLg+lIMKhhSdS0rR6fhUbV6/wYjHDSlcv+rSdrtmyWaHB7Pg6mFlpjJTlaI4XuVqk1NLbANiqmL8h/nYmc1SRlZo3KuqzibVpGahtc4UB9AErYsFTwuZ2ePRIpp5ntfyWAZr92tlSAdZCFjCl+IBIdG6ymAUYqYaDH6eolzf4ymVdmZpFSGWQahXGkGwU5Ek7N7qSALLLJeCQ1drwNXgSnXmHMsMh2uYqh+7RUhDM9VlTAuA5rTUcI9pzYcTtXFRGxz/d8fg3SvGQaVXPCsjQ9WTqY1Ti/tKGLqmZK8iyGQrrEtAbWfuLK3S4ZijIb5WjomrKMTFSE3oHhWbFYvadpTpZdV0v4Ci0Vws5DsqNKcbwq2Gio8hPRh2YkSGCOgkV5m/M7bVXb2+M0rmxvj2f9IKJfn0d6B9n8XBuB3Q8m3YBajxX0Dz+9dR8tk3Ox5Un+Axx7h0Zu1tMq4J2i2R0EABljBMiVJbRAbi1O7vXYl7Gkji4GA1MjAQnadx2JU/mA0W9zWmW9WjUzKSUIQAkCUhjyC9N+hdtbZMn3pyaHcHTIT5cHWLOCnkDtqvGaN6iVyA1e+zSpGqFrbjk28uOgDhJbCH/vv6k6BCVNNlAcDtcxk9PoFvuHwpV5h/xJLvkP6H7x2hZyTGpxIvWcqCGeh08LqIzqTxQDIxJ/mxXaUbTM0mkOrNnQuXC4bzUfTgHzLzcsGFRqhPBk1llcwn45mtDUe5p73VnmtB9OpL5IpqT/NIIlmKj/R5YCcSRCJh9Ahhbkwn/eBjTbEwZpxTK3abIAnZL9vD4ktVC1xNqyDzb4HOuGHZK5qsOlgDGrBBXk+wm5Re3NcxhGCJz2mPQlVCeXOmy8mxJ0yvaD1J8u8XxHJ3UgPRM7Gn2JaNqz1P2EDGPZl6uEw0L/aHjF9wDYk5wy/0tZQa3kJRYqxIbp0nMBA1cLT3zMkB6SuX+t5mzR9HZXXJ/pJ99tvbYHrnVMiQ5DXqG3vO9HTEuB8ioE0Xx2kFI6xeiTdD4nYAD3F/sPqqY6PlAGeSNhFJ1iH58HTgEPXgLIwQjbyKpcs8ivTu7+6Xh8T99Z9kStBBrNvfeO9wH8XbVySv2QF+Q1CNk3lKQAoR1V6I6vDOc0AnHjP5VLSvXrGUNTM4HoMKUQ5k0b1cuIMN7MO8GXnWhCP6XpWoLzR6dLcMS/4cPv0FjsftKG4w+mlStPHY+2CBmS47pC8LX3WaDFwSZBVM2FhAw9gohOlu2Jrq1CIcuSnV+D3Nn24tsaDfoMwCZy0RuJ7Cz4oc8bhrHs+vqfd3OxNcLNZMWJvd4hUDZ+c6J/bn+ODwUgFH2nXbt3qzLC8PnhxJP6FZR94w4hYcC1utNM76+e6oN3zhvcprlYP9gtgTWhu3MEBqgLEHKjngMHEQ3jRpHFbafozhKAAcE0gRlyOLBvb9/2F7I17Sv0OgnyvFQBzjHsC4e/lj+NaUpfRxUGC3tPvOC3Aic+5iRd0o7bJrHsVBPedkNyamWF7dFGkxjhpK2Ve6j7hcWXIS7Gnc4EOFRgu8dPyvpKxzYxGhJp/kfhm3/ZHqTu6HTdwe+Tn6z6c1j4Il3F0WkGj/YDzOCcnwTjcixKh9ha4kr5/j5ZMHEh1wYxZ1Dj7GrjujTVrJKXUhnL5Lv5+X1Xcty5Y1cBNUB6Q6zYkVC9GjiFCl9yeFUaTBsqrMZuvvRc/V66k4ZDw/U1SaY4rcp/FQLnAVn1COr+w8oqbqb3tSr92HKvXL4LJuMJaY/ujD5HQo0qZxTBmjPBAgzjJbdZjH3IdmqSYQ3nemSgMKRkkAtOmM/7T7apzZ2jebmbf6tYUlBOqQnWIKmEQRLowcd4zbM5b1n2yEBpaEjyIpFR9mItGJvAOOgRuwP+5DWr/2MjMDGr+TaB5M+cxtY8Vo/JBlFprSvAKa8uCDnutoK2Bdi6v+xeaNHNkW8QIGbePqNxEr/OYJkqwK/XW/sXpSY6kCj3qKtGd6o9s0bq/Eu4hb+/GzOAXLfpP1pHOvqrAb/sW3J5nKCzrZHmSAkSzTQrGcTf3A4iG4t3rPL2cxQ+zZ3EqZbZWb7rQlt2cIaH/X0o1Hkx4e1NVy6hB2LuJnlWoJ4C+W58BhMnr9OdqGjJVZoClXrGBB9+gi4cYrumAXt5LnP/v6mVgWVP2i1zeBy5aq2Iva6TUxJCBNWwcrfXy/MDdzvk8UJ0BTRFiWkiBx1WaJ2mYVwRHyQxXJsMr4iibwStLUFpyqjPU0pcB7pwGbaaxkiLGzfRERERkZ1z8guG+0

F+2mAEKz2tGCERERERkWNgDzORHSkqqcbGfWeQX1zV5v784ips3HcGRSXVPRwZEREREZHjYcJ
MZCeEEEg9kgGtrvGOx2l1jXjn8wwOzyYiIiIi6iImzER2Iu+q5rY9y7+kvlaFvGuabo6IiIiI
iMix2XTCrFarERsbiwMHDhi3JSUlYeDAgSb/fvOb31gxSqLup6mpx6ff5XfoOWezSrsnGCIiI
iKiXsJmF/1qaGjAqlWroNVqTbZfunQJTz31FBYsWGDc5uTk1NPhEXUrg0FAXaxBem4Z0vPKkH
+tCh0dYK2ta+iW2IiIiIiIegubTZi3bdsGDw8Pk216vR45OTlISEiAUqm0UmRE3aNKW48L6nK
k55XhQl45qmu7lvC6u/axUGRERERERL2TTSbMJ0+exIcffoiDBw9iwoQJxu35+fnQ6XSIjIy0
XnBEFmIQAgXFVUjPLcP5vDKor2o63It8J7GqvhY8GxERWdLBgwexa9cuXLlyBaGhoVi+fDkef
PDBNo+tqKjA+vXrcezYMQDAb3/7WyQlJcHd3b0nQyayOiEEGvUG48/augbkFt1ERD9vSCQSK0
ZGjszmEmaNRoPVq1fjhRdeQFBQkMm+rKwsSCQpSpKWl4dixY5BKpRg/fjwSExPh5eXVpes6O9v
0dG5yENW1DbiQV4ZzOU1DrTUtainfSYjSA8MjFTibVYricm27x0f084aqvy8/PIiIbNChQ4fw
/PPPY82aNZgwYQI++wzrFixAoGBgYiNjW11/DPPPAOdTod3330XGo0Ga9euRXJyMjZt2mSF6
Imso6ikGqlHMnCzxXen2no9Nrx3GgMCvRA/bTCClZ5WjJAclc0lzOvWrcNdd92FGTNmtNqXnZ
0NqVSK4OBgpKSkoKCgAJs2bUJWVhbS0tIglXYu6ZVKJZDLPdo/kKiDDAaBvKs3cTrzOk5n3MC
lgnIYzOhGdpU5YUS0EncPDsDdg/zhL2/qRSgo1mDNW9+g5g7DtT3c+mDFI3fDz48fGkREtkYI
ga1bt2LRokVYtGgRAODpp5/GmTNn8MMPP7RKmgxM+ePYsffvgBn3/+uXGE3csvv4ylS5dixYoVC
AgI6PHXQNTTikqqsXHfmduW1swvrsLGfWeQtGAkk2ayOJtKmA8ePIhTp07h008+bXP/smXLsPjx
xevBje3t4AAJVKBaVSiblz5yI9PR0jRozo1HUNgGNpv1eOyJz1NQ14Ke8cpzLLcX53DLcrDa
vF7lfXw/ERCowIqovVP190cc46kGgoqIGAODt4oS1j96N3YcvQt1G2aiIft5YOmMIvF2cjM9x
JLyxRWR9HBLZNXl5eSgqKmrVMZCamtrm8adOnTSZjjZ69GhIJBKcPn0aU6dO7dZYiWzNgUzoU6d
O7dZ4iaxNC
IHUIxm3TZabaXWNeOfzDLzwaBzbIrIom0qYP/74Y5SVlZnMWwaAl156vvsGiqbw
kAAMXFxZ1OmAGgsdHQ/kFEbRBC4MqNaqTnlSE9tww5RRoYRPvdyDJnKQaHyRETqcDwCAX6+rq
Z7L/dezJQ7o4XHr0bK9761jgsyU3mhBX/dRcigpq+sPL9TETdgUMiuy4/Px8AoNVqER8fj4sX
LyIkJATLLli3DxIkTWx1//fr1VlPZZDIZfH19UVxc3Ok4pFIJpFKO7lLb9HoDfjz+M0bdWJ
QQGBgL4eUpZs6ioKHz11VdtHv/ll1+abKuvr0dlZWWXFvzi+i3UU3T1el6pw+Xq18X
GtTt8t13OWwKHWcWHyb302nzBfunTJ5OcpU6ZgypQpVoqGHJkQAtfKtDif25QgZ12pNKsX2dl
JioGhvoiJUGB4pAIBcjf2rBCR3SutrEXG5QpkFlTgbHZph557NquUCfNtDBkyBB4eHjh37hzi
4uKM27OyshaAaGtrq+FGjRuH1l19HQUEBwsLCAAAnTpwAAIwcObJLsXC9C7IkIQTKbtY1JcJc
YlxNUoqatGVTmMfD5nJugntGRGl4HubLMrmE2ai7qSr1yPjcgXSbyXJpTfr2n8SgL4+rhh+a7
GuwaFyuMjYi0xE9q2iSofMyxXIKGhKks1tD9uirbt96bveztXVFUuUxLsX27dsREBCAaJgYHDl
yBN9++y3effddzkV6lJeXw8vpCkuou9Rq79HFCiNIDIf6e6O/viRBl0z83Fyf8Oe2UWaNcwoO8EBHk3e5xR
B3BhJl6FSEErlfUGnuRL12uNCmPcjtOUglU/X2NK1oHKdzZi0xEdk2jrcely5XILGhKkovLT
c81921j8XO5YgSEhLg5uaGLVu24Pr164Mi3MS2bdswZswYFBYWYtKkSdi4cSNmzZoFiUSCt95
6C8nJyVi0aBFcXFzw29/9fklJSdZ+GTbvfWkN9n+ZDQQCYf380ghQc2tpRxl7jEtPk+EYXe437
+rgak+L+txJkpdwN0tt8t4qfNviOiw4CgLuLMx6bOpjfz8jiJEJ0dWq9/dPrDSgvd5y5DmSqv
kGPzMuVSM8tw/m8UpRUmtdr4uft0jTMOkKBQWFyuLnY1v2lP779Hco0Ta9F4e2K1xLusXJE3U
+p9LJ2CBbH9od6irauAZcuVxqHWReWmP++c3aSoFFv/teFtY/e7XBDsh2t/ekNbc//++hHXMg
rBwAMj1Dg2Yc7X360N2juNW5Oii3RayzrIzUmxS3/6+7a8e9UzWXt2uppDg/ywmNTHbOsnaO1
PfbItjIA6ja97S7rjYrmucjlyLxcgQYz5rI4SSWIDvHB8Egfg3cpichu1dU3Irvwp
nGIdcH1KrNXn3V2kiCynw8Gh8kxKEyOAYFe+Mv7ZzgkkuzKtdKfR01cLXXsmwMdIYRAmabOpM
f4SkkNbpRru9xr3LLHuL1e444KVnriT4visOKtb41zmt1kTljxX3chIsib39mo2zBh7iU+OJq
Nn9RNd1n/djTH4e6yNjTqcelKZVOSnFuG6xW1Zj3P11NmHGY9ZICfzfUiExGZq75Bj9yim7d6
kCuhvqYxa+FCAJBKJAjv54VBoXIMDpMjKtin1r/HBJJZH909XoUldbgyo0qFN5o+u+VkhrUt
lMi7k6ae41bJsed7TXuKIlEAmenn+uHu7v2cbjRLGR7mB30Eo54l7W0srap5FNuGTIIuV6+UVM
1eZKlEgqggQHwyP8ENMRLTKpVDN5VDTILKpB5uQI5RRqz1mQAAmA0EAvDA5t6lWODvGBg4gDSK1ikZ
ODvFp94ZhsNITSQtG9sohkUS2rrnX2JgUW9OxCm3uNfCM8uNfd0glfK7E/UeTJjbjQ0

GpBdWGlcsOtamXkL1Ph4yDA8QoGYSAWGDJBzMRoiskt6gwEFxdXGlayzCyvNulHYLETpYexBV
oX6wqMTbSGHRBJZn65Bj6KSGhSWVOPK9aYSToU3qu84+qM9Mmcpgn8xnDpE6cHvTERgwkw2ru
xmHdLzmhLki/kV0DW0v/CERAJEBvs0JckRCvQP8LTY/Bkiop5iEAKFN6qNq1hnFVZ2aPGdAD/
3pjnIob4YFCqHt4fMInFxSCRRzxBCoFyja1XX2JK9xs3JsT97jYluiwkz2ZRGvQHZhTebkuTc
MhSZOXzc270Phhl7kf3g6cY7okRkX4QQuFamNS7SdelKJaprza9n3NfHFYPC5MZh1nIvl26Ml
ogsSdegx9XSGmNS3Lwgl2V6jT3Q39+LvcZEncSEmayuokpnTJB/yi9HXb0ZvcgAIvp5Y3iEAs
MjFQgL9GIvMhHZFSEESiprmxLkW/WQm4c5m8PXU2aSICt93boxWiKyBGOv8S/qGl+v0Jq9in1
bFN4u6O/vhZAWyTF7jYksgwkz9bhGvQG5RTeRnleO87llKCypNut5nm59MCzCDzERCgwN94OX
u2WGFxIR9ZRyTZ2xBznjcgXKNTqzn+vp1qcpQb41zDrQz51zholsWH1D8wrVlu419jCpa9zf3
5O9xkTdiAkz9YjK6pa9yBVmlzMID/Iy9iKHB3rzTikR2ZWbNfXGOciZlytww8ySd0BTiaaBob
7GXuR+Sg+OpCGyQUIIVFTpcPmG5XuNQ5Se6B/g2dR7rPRAgNyd34WIehgTZuoWekNTuZP0vDK
czy3D5evm9SJ7uDpjaLgfYiIVGBausNgiNUREPaG6tgGXLlcYh1l3pIyfSx8nqPr7YlCYLwaH
yRHq78UvxkSdJIQwKbOmrWtAbtFNRPTr2mruv+w1LrxRjcKSatTUdb3X2KSusb9np1ayJyLLY
8JMFqOpqTeuaP2TutzsD4+wAC8Mj2xasCsiiL3IRGQ/anWNuHSlaf5xZkEFrtyoNnv12j7OUk
QF+xiHWQ8I9DJZfZqIOqeopBqpRzJM1gSorddjw3unMSDQC/HT2q8X3txrbDKcuqQaxeVd6zX
283ZBf6XpCtXsNSaybUyYqdMMBgH1tZ97kfOLq8x6npuLM4aF+zUNtY7wg48nV3IlclTXymqw
/8tsAMD8+6MRpPCwckRdo6vXI7uoEpkFlcgoqEBBcRUMZn57dpJKENHPG4NvJcgR/bzRx9mpm
yMm6l2KSqqxcd+Z284Tzi+uwsZ9Z5C0YKQxaW7uNS78RXLclV7jPs5SBPf1MPYWh7LXmMhuMW
GmDqnS1uOCuhzpuWW4oC43u+RJqL8nhkcqMDxCgchgbzhJ2YtC1Bt8cDQbP6nLAQB/O5qDZx8
eYeWIOqah0YC8qzeRcWsect5VDfQG8xJkiQQYENiUIA8K80V0sC9cZEyQibqLEAKpRzLaXVRL
q2vE//voHKKCvVFYUmORXuOWw6nZa0zkWJgw0x0ZhEBBcRXO5zYNtVZf1Zg13NBV5oShxl5kB
euBEvVS10q1xscdmc9rLY16A/KLq4wrWecU3URDo6H9J6Kp3F1/f08MCmsq86QK8YW7Kz9miX
pK3lWN2aPdKqp0OJlZ0qHzN/caNw+nDvX3RLDSE55u7DUmcmT8JO8FOrrwRXVtA35SN5V8uqA
uQ5XWvF7kEKUHhkc0zUWODPbhXDwisnkGg8DlG80JciWyCiuhM6MWfLN+fT0wKLRpka6BoXJ+
cSayorPZpRY7l9zLxaTHuL+/J/zlbhwhR9QLMWF2cOYsfBHU1wNXrlfjfG4p0vPKkXv1pllDk
1xkThgSJkfMraHWft6u3fhKiIi6ziAErpbUIONyUw/ypcuVHaqJ6i93w6DQn2shcw0GItuhrT
PvBn9LfZyl6HdrrnF/f0/jgly8+UVEzZgwOzBzFr5Yt/ckXF2cUFNr3hfGfn09MDzCDzERCkT
392UvMhHZNCEEisu1yLzctEjXpcsVZo+aAZrmJg4OlRtXsuaNQSLb5d7BBbV+MyIIC6cMZK8x
Ed0RE2YHZe7CF3qDuGOyLOsjxZAwv1sLdvmhr4+bpUMlIrKo0srapkW6bvUiV1bXt/+kW7w9Z
Mbe48Fhcih93bpUs5WIek5sdF98/n2B2cf/ekQ/JstE1C4mzA6qIwtf/FKgn7txLrKqvw/Lnh
CRTauo0iGzRYJcerPO7Od6uDo3LdJ1a5h1kMKdCTKRnYro540BgV5mff8JD/JCRJB3D0RFRPa
OCbOD6ujCF0pfV0weFYrhkQr4+7IXmYhsl6amHpmXK4zDrK+Xa9t/0i1uLk5QhfjeKvUkR4i/
J6RMkIkcgkQiQfy0wXecjgYA7i7OeGzqYN4cIyKzMGF2UB1d+GLoAD9Mujukm6IhIuq8mroGZ
N1KjjMvV6CwxPzyVLI+UkQ3J8ihcoQFenIIJpEDC1Z6ImnBSKQeyWizpzk8yAuPTR2MYKWnFa
IjInvEhNlBdXThi44eT0TUno6WtGtWq2tEduFN4zDry8VVZtV/BwBnJwmign2Mw6wj+nlzcUK
iXiZY6Yk/LYrDire+NVYJcZM5YcV/3YWIoDu3P0REv8SE2UF1dOGLWFXfboyGiHobc0raNffw
1DfokVN009iDrL5aBYM5te0AOEklCA/yxqAwXwwOlSMy2AeyPlx3gai3k0gkJjfL3F37ILKfj
xUjIiJ7xYTZQXHhCyKyFnNK2q1/7zTuHRqIotIa5F69iUa9eQmyBEBYoJexzFN0iA9cZfwoIy
Iiou7BbxkOigtfEJE1mFvSTlevx7/OFpl1zhClR1OCHCqHKtQXHpxCQkRERD2ECbMD48IXRB1
38OBB7Nq1C1euXEFoaCiWL1+OBx98EACQlJSEAwcOmBwfEBCAY8eOWSNUm9SVknbNAv3cjatY
Dwz1hbe7zELREREREXUME2YHx4UviMx36NAhPP/881izZg0mTJiAzz77DCtWrEBgYCBiY2Nx6
dIlPPXUU1iwYIHxOU5OnC/bUkdL2gFAXx9XY4I8KFQOuZdLN0RGRERE1HFMmHsBLnxB1D4hBL
Zu3YpFixZh0aJFAICnn34aZ86cwQ8//ICYmBjk5OQgISEBSqXABP/sfQboq
GiIiIqGuYMBMRAcjLy0NRURFmzJhhhsj01NRUAkJubC51Oh8jISGuEZzc6WqJO4e3aTZEQERER
dR0TZiIiAPn5+QAArVaL+Ph4XLx4ESEhIVi2bBkmTpyIrKwsSCQpKWl4dixY5BKpRg/fjwSE
xPh5eXVpWs7OztOneC4Qf4dKmk3arC/Q73+3qLlbB6JxLHew0RERC0xYSYiAlBdXQ0AWLNmDZ
YvX45Vq1bhiy++QJCAvbu3Yvs7GxIpVIEBwcjJSUFBQUF2LRpE7KyspCWlgaptHMRm1Qg1Qq9
uYcmXYlVxvu6I6u+LnCuV7R4b3d8Xdw8N4loKdkgqlZg8dqT3MBERUUtMmImIAPTp0zD4+
HjNnzgQADB48GCBcvXsc+dOLF68GMFR6eTTXLVSoVlEol5s6dy+dOLF68GN7eTTXLVSoVlEol5s6di/T0dIwYMaJT1zYYBFSuru
WVehI14bOogrE87BW3dHUrauTpjydRBqKx0rNfeWxgMwuuRxRUWNFaPpGbwpYH+C+rqjTFMHAO
jXl38/IuocJsxERAACAwMhEAgQFxdnslzsrcWsCwCmMvu2uAi7I7UjD04d1IWDBIkC46UyZj2MjpUk
iIuLM27PysrCTSUxqisnczOCPDMk7NsQxeNXm8Cwocm
Li6umLp0qXYvn07AgICEBwNHjuDbb7/Fu++gCh6MTWrhVmw5TPMknmwoVUZdpdPMgwMJ7JyaYmX9v8bnqoPUZ9P/Fu+++C61Wi2XLlmHHjh2YNm0a1Go1Xn75ZUyfPp

0rZ98GS9oRERGRvWPCTER0S0JCAtzc3LBlyxZcv34dkZGR2LZtG8aMGQMA2Lp1K1JSUpCSkgI
vLy/MmDEDiYmJ1g2aiIiIiLoNE2YiohaWLFmCJUuWtLlvypQpmDJlSg9HRERERETWwsKJvURQ
X3fjY64USURERERE1D72MPcSXCmSiIiIiIioY5gw9xJcKZKIiMg2FBUVYeLEia22r1+/HnPmz
Gm1vaSkBBs3bsS3334LABg7diySkpKM5fCIiKj7MGEmIqJuE9TXHWWaOgCcDkLU7NKlS3Bxcc
GXX35pUmLNy8urzeOfffZZ6PV67N27FwCQnJyMhIQEHDhwoEfiJSLqzZgwExFRt+F0EKLWsrK
yEB4eDn9//3aP1Wg0OHnyJHbs2IEhQ4YAAJ544gkkJCSgoqICcrm8u8MlIurVmDATEVG34XQQ
otYuXbqEqCjzbiC5uLjA3d0dBw8exwoEAhw4dwoABA+Djw7rmRETdzaYTZrVajVmzZuFPf
/oTZs2aBQDIyMjAhg0bcOHCBfj6+mLhwoWIj4+3cqRERERE5snKyoJSqcd/H1P1
sNmFuaGjAqlWroNVqjdsqKiqwZMkS3H///6I5ORk+Pr6Yvbs2VaMloiIiKh99fX1
yM/Ph5ubG1avXg13d3ccPnwYjz/+OPbu3Ytx48aZHC+EwkVLLlxAbG4ulS5dCr9djy5Ytpp
/HBBx/A09OzU3FIpRLI5VxXgOyPVCoxecz3MXU3m02Yt23bBg8P0/8BPvroI9rxm7OBzs+/fj3/
/+9/G5DglJQX33XcfPv74xatKhKH7MGrUKDg7/xz22LFjsXPnTpSVJ1Fgxmu7HGwLWZ5MJ88mT
J/Hhhx/i4MGDmDBhgnH7qVONnMGrUKDg7/xz22LFjsXPnTpSVlUGhUFghWiLr4OrDRGQtbH+6x
t3dvdU2lUqFb775ptX206dPIzw83KQn2cfHB+Hh4cjPz+9SHI2Nhi49n8gaAhXuKL3Z1P4EKT
z4PqZuZ3OD/jUaDVavXo0XXngBQUFBJvuKi4tb1RxsXmHy6tWrPRYjkS2YNykaQ8P9MDTcj6s
PE1GPYvvTeZmZmYiNjcWpU6dMtl+4cKHNhcCCgoJQUFAAnU5n3FZbW4v7xEtobt
D/U0m+thXrduHe666y7MmDGj1b66ujrj8KVmLi4uAGDyQdIZXDCA7E3/AC+seWSktcMgol6Iq
593nkqlQnR0NJKTk/HSSy9BLpfjo48/oi///3v0Ov1KC8vh5eXF1xdXfHQQw8hNTUViY
mJ+MMf/gAAeOONNyCTyYwLohL1Jmx/qKfZVMJ88OBBnDp1Cp9++mb+11dXVFfX2+yrTlRbmt
4k7m4YAARERH1BKlUipSULz++utITEyERqPBkCFDsHfvxXgwcOBCFhYWNGkSNm7ciFmzZsHf
3x/79+/Ha6+9hkWLFkEqlSULg4ffPABvL29rf1yiIgcnkQIo/rGcsXLgQZ86cMelF1mq1k
MlkCA0NRb9+/eDr64vVXXzZ8+fVCLg+WmmpSNm7ciFmzZsHf
desITIESiVXtYOwaLY9hDZB0dre+yRTfUwv/7666irqzPZNnnyzDzzzDOYOnUqjhw5gr/97W/
Q6/VwcnICABw/fhzh4eFdXvCLm/wBAoVAgODgYs2fPRxV1
NdauXYucnBwcOHAAaWlpePLLJJ60cOREReRERETkam0qY26NcnOkbMxavBWLAlWrMnDkTb731l
o2ZM2daOzQiIiIiIiJyMDY1h1h9laOI+HyD444jwetj9E9sHR2h+2PUT2wdHaHntkVz3MREERERE
RERD2FPcwAhBAwGHr9r4HI5jk5Od49PrY/RPbB0doftj1E9sHR2h57xISZiIiIiIiIqA28ZUF
EREREETUBibMRERERERERG1gwkxEREREETUBibMRERERERERG1gwkxEREREETUBibMRERE
RERERG1gwkxEREREETUBibMRERERERERG1gwkxEREREETUBibMRERERERERG1gwkxEREREE
ETUBibMRERERERERG1gwmxFAwcOxIEDB6wdRrv+/e9/Iycnx9phTJ0
5g4MCBKCws7MHIqLdg+9M7se0ha2Pb03ux/SF7xYTZir755htMnTrV2mHcHUFREZ566qk7Nm7
UcSdPnsRzzz2H2tpaa4dCvRTbn96JbQ9ZG9ue3ovtD9krZ2sH0JsplUprh2DXDAYDfL29rbHLc
tbH96Z34OyVrY9vTe/H3SvaKPcxW1HJY0nOgvG4JFHcP78eeMPFM2GdRu4/dZEbRWUldzyHV
qvF+vXr8atf/Qf/Q1fLrG2ZcX6t/P45JFHHHKfFMTAwmTJiA5JiDGbmD4DKtsFcKBMZJgd9VgRSkVFdXG/dPPnDgRu3btw1PDZtowpsssXLn
9//3vExsZizJgxeOWzZcqGxzOWV/DqiiJvgkweqkQK8/5H5HHJgnvfAKtmRRSHa9i27ZtAtAq/935C
SJM6FCxfi+eefx5w5cxAXF4e6oNUDYnnvK5zdb4GQkIAxY8ZgsyLqgsLcWgi+cfaSWloYTJ07g0Ucf
BQBMmjSp3aFpX3/9NfpH/4NWbMmIFhw4Zh2rRp8eDj8MqkZXmvAqo7rT+Oqrr4Hno93n33XxUyZMgDhw/HH+4n3n
zhxAkOGDMH333333+PqVOnyjvjw4Zg7dy/7UajV27t/jP/ia//+xnam+yf//735g1axZsvfL/7u94Ifo+Ym
LwwAMP4I033kB9fb7bff0fOKFkWx/HBfbHrJlbHscGu9TlkEalWolSMyulx6KP/5YCCHEmjVmNxT0aZ+N
zzz0ncnzJyxLFjx8To0aNPUltLUlSHc/xBNPiIkTJ4vqv4q8vQ/5a5Ofni14Vr14q4uDhDhn
w8X27duFWq0WJ0eFHPmzBFBGybSR0tKNW7733733J4NsB6oP3ykKDEN40JzPkNqKoHjLRRzI
GNjozh37pxQVTiiy++ENXVaK4uFiMHj1a2xGhYgjGLoXgTTGXS2C8TAgQPF4c
OHRVZWligvL+/eX6QN0mq14t577xUe9fVrzTk5Ij8/Ij8/X2/zevFmoVCpx7tw58cUX/XeZgfl
O77//XqhUKjF58mvv8Hr5fff/0r8XLi7x958cUXUVdqSqiuLiTfFZl5NjkcCrPrpvUvp+qw7HuHLCLw4Yg/FUOF
6FDx17/+1eQcv/vd78TTTzsVYbVKsx7iIoqRJKsPLLto0awXbNNNs8X3wQoKCpWvVKGjhxaf
X//flFQUCD+7//ZPfDxDfF/lQKcL/ds+5sehW8e2x3x/SFHHx5/SFHhGy/Yjx7QpFIWyj
9v3L9hwwGyxefLk2z4/Ly9PqDhwGl+2lqmmXPzXgV/f+rbBkcS4n+fHbyT3WbFEOkUKzlkUKUcqDbtemoyyqBPUxb
9998LIZo+NJYtW2zyzO9+zvxpz/9SQghxJR4xbxqOjLr0eL/37yAzUZzE7MvOhMznji5g+fbrJ8VqtsVsTExBhfy4IFC8RD
Dz3Uod+FoykrKxM7d7d+4UVVVxm06nU6oVCrxcxGuBvzK1eu3XVXm0//fST8cOmq
qpKDB06VLz33nvMnz9/naq0rEE01Ne8y4kM5Fvqx+V29mlkNzhmGp5zkqfNUeDsfBPLKMFy9rB8B5usfnvNbd/AaLh3VPPeY
LevHkiOTnZ5JzHjx9x9vN16yL2x/HBPbHrJ1bHscF9sfclScw2xDwsLC0KdPH+Kc6zTbBPjcPiBPbmdxvcyPPyt8eaGhoAAC
8+OKL+PTTT437nnzySQwbNsytzNwZr5yUMtQzkJ9BAyGQ1AIwgFDs1AyrNoEqbxmrsSLhTbJt0b52GKtrJbm4kamWdKMoGjoZAaCl

```
jIw02dfyur908eJF5ObmtjqnTqdDbm6uyWvpzfz8/DB//nx8/vnnyMzMREFBATIyMgAABoOh1
fHTpk3D1atXjT/v3r3b+Dg8PNz42NvbGwBQV1eHvLw8NDQ04O677zY5V1xcHPbu3WuyWEnLc7
i5uaFv375wc3MzbnNxcYFOpwPQ9Dc+f/48PvnkE+N+cWvIUm5uLkJCQjrwmyB7wfbHMbDtIXv
DtsdxsP0hR8WE2YbIZLLb7vvDH/6A+Ph4488+Pj44deoUAEAikbT5HIPBgBkzZuCpp55qtc/P
z++O1xW3WZjBYDBg7NixeOml1rt8/LyMj52dXW9zSvpHUpLS/Hwww9DLpdj0qRJGDduHIYPH
47x48e3efyuXbvQ2Nho/DkgIADnzp0DAEilrZcaEE2jQwC0/vs3fyg5O//8v3fLx7c7Z8vnL1
26FDNnzmy1zx4Wa6HOYfvjGNj2kL1h2+M42P6Qo+KiX3ZCoVAgLCzM+M/X19d4dzQ9Pd14XGN
jIyZMmIAjR44gOjoa2dnZJs/T6/XYuHEjrl27ZtZ1f9kgRUdHIzc3F0FBQcZz+vj44JVXXkFW
VpblXrCd+/TTT1FZWYm//e1vSEhIwAMPPICbN28CaGrwf/l7DQ4ONvk7mfOhGxERAWdnZ+OXh
2anTp2CUqmEj49Pp2KPjo5GXl6eSTzXr1/Hq6++ipqamk6dk+wb2x/7wbaHHAnbHvvC9occFR
NmOxYeHo7JkycjOTkZx48fh1qtxosvvoj6+nqMGzcOjz32GDIyMvDiiy8iJycH586dw6pVq6B
Wq41Dmtrj7u4OAMjKykJVVRXmz5+PqqoqrFixAhkZGcjMzMTKlStx/vx5REdgYGo
ra3FP/7xD1y9ehXffPMNVqxYAQCor683/l4zMzM73RB7eXnh4YcfxptvvolPP/0UBQUFeP/99
7F//3489thjt7373p7HH38c//znP7Ft2zao1WocP34cSUlJ0Gg0vMtKRmx/bBPbHnJ0bHtsF9
sfclQckm3nNm7ciFdffRXPPvssdDodRowYgXfeeQd+fn7w8/PDnj17sHXrVsyaNQubm4YO3Y
s1qxZc8chUC3J5XLMnj0br776Kgok0KCvDCCy9g37592Lx5M+bPnw8nJyfcddddSEtLg0Kh6OZX
az9++9vf4qeffsKmTZtQXV2N4OBgzJkzB0ePHsX58+cxe/yxTl1n7
dq1kMvl2Lx5M0pLSxEWFoYXX3wRDz/8cJdi37JlC3bu3ImdO3fCx8cH9913H/74xz92+pzkmN
j+2B62PdQbsO2xTWx/yFFJxO0mbBAREREREH1YhySTUREREREREREREREREREREREREREREREREREREREREREREREREREREbWDCTER
ERERERNQGJsxEREREREREbWDCTERERERERNQGJsxEREREREREbWDCTERERERERNQGJsxEREREREREbWDCTEREREREREREREREREREREREREEREbfj/CeFDEnEDD
8kAAAAASUVORK5CYII=",
      "text/plain": [
       "<Figure size 984.597x600 with 6 Axes>"
      ]
     },
     "metadata": {},
     "output_type": "display_data"
    }
   ],
   "source": [
    "#sns.pointplot(data=df_ci_plot, x=\"program type\", y=\"value\",
hue=\"program type\")\n",
    "g = sns.FacetGrid(df_ci_plot, col='task name',  hue='time
point',col_wrap=3, sharey=False, margin_titles=True)\n",
    "g = g.map(sns.pointplot,'program type','value', dodge=True)\n",
    "g.set_axis_labels('', 'score')\n",
    "g.add_legend()"
   ]
  },
  {
   "cell_type": "code",
   "execution_count": 45,
   "metadata": {},
   "outputs": [],
   "source": [
    "n_times = 10_000\n",
    "change_score_dict = {}\n",
    "res_dict = {}\n",
    "i = 0\n",
    "creyos_tasks = {'SS':'SS_avg_score', 'DT':'DT_final_score',
'ML':'ML_max_score', 'RT':'RT_final_score', 'FM':'FM_final_score',
'TS':'TS_max_score'}\n",
    "for t, score_name in creyos_tasks.items():\n",
```

```
    "    diff_score_1test = get_test_df(df_2programs, score_name)\n",
    "    res_init = 
stats.bootstrap((diff_score_1test.loc[(diff_score_1test['trt_grp']=='Init
ial'), 'diff_score'].dropna(), ), \n",
    "                 np.nanmean, confidence_level=0.95, \n",
    "                 n_resamples=n_times, method='BCa')\n",
    "    \n",
    "    res_virtual = 
stats.bootstrap((diff_score_1test.loc[(diff_score_1test['trt_grp']=='Virt
ual'), 'diff_score'].dropna(), ), \n",
    "             np.nanmean, confidence_level=0.95, \n",
    "             n_resamples=n_times, method='BCa')\n",
    "\n",
    "\n",
    "    change_score_dict[t] = 
res_init.bootstrap_distribution/res_virtual.bootstrap_distribution\n",
    "    res_dict[i] = {'task name':t, 'program type':'at-home', 
'ci':'low', 'value':res_virtual.confidence_interval[0]}\n",
    "    res_dict[i+1] = {'task name':t, 'program type':'at-home', 
'ci':'high', 'value':res_virtual.confidence_interval[1]}\n",
    "    res_dict[i+2] = {'task name':t, 'program type':'in-center', 
'ci':'low', 'value':res_init.confidence_interval[0]}\n",
    "    res_dict[i+3] = {'task name':t, 'program type':'in-center', 
'ci':'high', 'value':res_init.confidence_interval[1]}\n",
    "    i += 4"
   ]
  },
  {
   "cell_type": "code",
   "execution_count": 46,
   "metadata": {},
   "outputs": [
    {
     "name": "stdout",
     "output_type": "stream",
     "text": [
      "SS 0.9074\n",
      "DT 0.203\n",
      "ML 0.3582\n",
      "RT 0.0472\n",
      "FM 0.2717\n",
      "TS 0.4751\n"
     ]
    }
   ],
   "source": [
    "for t, score_name in creyos_tasks.items():\n",
    "    hist = np.sort(change_score_dict[t])\n",
    "\n",
    "    idx = find_nearest(hist, 1)/n_times\n",
    "\n",
    "    print(t, idx)"
   ]
  },
```

```
  {
   "cell_type": "code",
   "execution_count": 47,
   "metadata": {},
   "outputs": [
    {
     "name": "stderr",
     "output_type": "stream",
     "text": [
      "/Users/rye/opt/miniconda3/lib/python3.9/site-
packages/seaborn/axisgrid.py:718: UserWarning: Using the pointplot
function without specifying `order` is likely to produce an incorrect
plot.\n",
      "  warnings.warn(warning)\n"
     ]
    },
    {
     "data": {
      "text/plain": [
       "<seaborn.axisgrid.FacetGrid at 0x167df7fd0>"
      ]
     },
     "execution_count": 47,
     "metadata": {},
     "output_type": "execute_result"
    },
    {
     "data": {
      "image/png":
"iVBORw0KGgoAAAANSUhEUgAAA4kAAAIzCAYAAACtCFzyAAAAOXRFWHRTb2Z0d2FyZQBNYXRw
bG90bGliIHZlcnNpb24zLjQuMywgaHR0cHM6Ly9tYXRwbG90bGliLm9yZy/MnkTPAAAACXBIW
XMAAA9hAAAPYQGoP6dpAADJsUlEQVR4nOzdeVyU1f4H8M+wDIuAgoIghCgqCIKCIJgQLuGCWW
YuqbjgminYIio/vdFi6RXUmwtqCq6lZm430wqt1ApRxDUBkdxYBwUZ9m3m9wfx6FwWGQSH5fN
+vXw1nI3vI3Sc7zznOUckl8vlICIiIiIgKgpuoAiIiIiIqOlgkkhEREREQCJolERERE
REQkYJJIREREEAiaJREREREJGCSSEREREERAImiUREREERCRgkkhEREREQCJokkk
Mvlqg6BiFo5zkNEpEqcg4gqMEkkAMDp06exZMmSBh93ypQpmDJlSoOP2xtGlxcXO
Dg4ABvb2+sWLECDx8+rNL20qVLVLeOedd+Dm5oZevXph4MCBCAoKwv3791UQOVHD4DykWjY2Ngp
/7Ozs4ObmBj8/P5w5c0ah7eDBg6u0/98S5cuVdGVENUP5yDVqpw71229TCaDp6cnbGxs
cPjwYQBAcnKywtfUcDRUHQA1DTt37lR1CK3akSNHsHTpu++ja+ja+++gq//
vorDh06hHbt2gEAoqKiMGvWLAwZMgQrVqyAgYEB7t+/j4iICIwbNw4HDx6EpaWlai+IqB44D6
ne2LFjMW7cOABABAaWkpMjMz8d1332HOnDn417/+BV9fXwDAxo0bUVJSIvRbsGAGAB7Ozs8O677wp
lRkZGLzZ4oufEOUj11NTU8OOPP+KDDz6oUnfx4kVIJBIVRNU6MUkkagI2bdqo157DZ9++qlQ
5u7uDhcXF7zxxhv47rvvMGvWLWLADAli1b4ODggPXr1wtt3dzc4OXlBW9vb+zYsEv/BqIq
PkzNTVFnz59FMpGjBiB+fPnY9WqVRg4cCAsLCxgZ2en0EYsFsPIyKhhKKXyIiZTg7OyMmJgZ//f
UX7O3tFep++OEH09OzZE3FxcSqKrnXhclPCOHCBVy4+++i7i7///rvG73Xu3Dn06tUtQULQUFNa77
nzJlCpYtW4avvvoKvvvoKAwcOxIWB5cRufWwaXxS7dOuF5nsQuiFP4k/Wqz6RbYTe/jW2
Hvb09PD09sWLFChQVFl9//zFu+++i7///rvG73Xu3Dn06tULQUFBNa77
nzJlCpYtW4avvvoKAwcOhIODA95++21cvXpU6cwadIkODk5oVevXhg+fDj27t0r1EdHR
8PGxgZRUVGYMmUKHB0dMXDgQBw8eBAyQ8PGxUrv8elS5fWuvSq8u+Og8fPqz278PW1hZBQUHo1auxQtvqmJiYYPny5RgwcRI
1RZyHVD8P1UQkEuHDDz9EvLw8e+vTx+HDt2bL/3GP59UMx/DxBqBRyGUN+NeD12GnBx1EOO1dnovcmiF2
dhsyJOHjrRdr0sdZSx4uhJsH3UPkzJZtYAhOpbIJQKQNYAy/RIqoBeLD1gTjKkxqN+bMR5rZEEC/ZRWxOv
.
.
```

Cfv27cPp06dx8OBBODo6ori4GOPHj8fDhw/x3nvvwcTEBIcOHcLp06exfft29O/fv9q479+/j
6ysrBr/nrt16wY9Pb1q69577z2cPHkSr776KkaMGAFXV1d07Nix2rYhISHYvn07+vXrhzfeeA
Nubm546aWXavy+RM0B5yHVz0M2NjZYsGAB/P39q60fOHAgXnrpJezZs6dK3eDBg9GvXz+sWrW
qxu9N1JRxDmo6c1B2djbOnj2LU6dOCXXnzp1DQEAADh48iJEjR2LlypUYM2YMkpOTMWTIEOFr
ajhcbkoK/8NWLhW6cuUKevbsiS+//FKoe/nllxEVFYWLFy/inXfewbVr11BUVIS5c+cKCY2Zm
RlOnz6NgoIChUng2rVrmDt3LoYOHVrrpFiprKwM4eHhwhj5+flYsmQJ4uLi0KtXL9y+fRujR4
/GsmXLhD5OTk5wc3PDxYsX4ezsLJS/9dZb8PPzAwDo6upiwoQJcHR0REBAAAACgV69eOH36NGJ
jY+Ho6Ihjx44hPj4e3377LXr37g0AeOWVVzBlyhSEhobi0KFD1cZsaWlZ72cBP/vsM8hkMvz8
88/CpGhpaYnBgwfDz88PpqamQtuFCxciNzcXhw4dwoULFwAAHTt2xMCBAzFt2jRYW1vXKwYiV
eI8pPp56ZFk6dOuaOc1DTmYN8fHzw9ddf48aNG8JKqhCHQ1tZ+rrGp7pgkU
U8PDzg4eGB0tJS3LlzB3fv3kVCQgKysrEKDVR69+4NLS0tjBycn4XWHDh2E
NTRU+BQsODn7mpAhU/aSpcuItLCwEAOH5vIKCAty/fx937tzB9evAVRstvA0Jycn4XWHDh2E
2CsZGhoCAHJzcwFUbAxjbGwMe3t7lJWVCe0GDRqE1atXIycnB23btq0Ss0wmg0wmq/Ga/
RKJqq3T19fH+vXrkyZycjDNnziA6OhrR0dHYuXMxMnvv32W4SHhwuTvTvgsxqeffgp/f3/f3
evXqhpKQEp06dQkhISK39qGBwaAJFSYnzX+jKySU4sZEwASYnJ8PBgpQKBnvoviawcBD22IqCVjkkhIe5
+OHDlS47i7d++Gm5ObvjfDvrE/p5MmTxuxVBYWJFle2djY2NMXbswWIwdOxZ
Ar90qVLOHDgALZs2QIbGxv4+PgAALp3746tW7diw4YN2LZtG0aOHFnlEzZlLVq0CElJSdixYw
ecnZ0hFotRWFiIgwcPPte4QMVdPSSsrK4SGhlZbb2FhUW35ggULMHx5BrH7dKlS7X7XlP/30E4K
Dg7Fv3z6FNmpqahg6dCguXrIb7/9FgBw9epVzJs3DyEhIVU2qHFzc8ocuVKZGdno337
9rVeJ1FTx3noxc1Dz5KUlASJJkJybvVqz9Rc8Q5SHzkI+PD3bt2oXr16/jxIkTGDp0KDQ1N
evUlxoGbzUAFS563Tp0iV069Y3bYSpeTbFjIwM3OSSgogZFwSpRt7/fP
bZZwCAtLQ0YSxDQ0NoaGhg3rx56NSpEyYYGKkyWLsyjRg26hGHDhsHd3R1isRgAcPbsl
DXfTr1w9paWlo3749HBwcn/n3/9T2+oVq+1nyYWGh0t9r9r6e3
u3fvokePHgAAKysrFBYWYvu3vu3dVe5507d2BsbMzzzahzyahZ4jz0xIueh551
0XUpHEOekLVc1CfPn1gbm6O77//nmpF65/bg/33333r3NVUB3KkkAICBgQEuX76HhYf
jqq6/Qp08f3f3Lt3D1u3bkVJSRmwFt7d3R2hoaeFf9q2bk3F4ZMwXvv7e2r3mpqaitUBsFmPQoEFVvoe2tqdoY
+9a9/Yc6cODi2bZvCocvKcnR0xPfffw97tXUwFfffw97e3uYmpri8uXL2Lp1K0QikRBffY0xRwZMZ479+6Fn58f
3nnnHZiZmeHPP//Etm3/b4Ovr2+CfZHXt2hVz5szB1q1kZqaitdffx2mpqZ49OgRgjhZj6hqioK
OzYsQMA0LZtWyxZsgTBwcGYNGkSxo8deegm5ubmIjIzEkSNHEoayueGqFniPPTEi56HKq
Wnp+PKlSSAKjbNyMjIjwb5wJEjR/D6cucH7E0VP8DGFOJtOrCfMUGdo9u1UuH+sLvwchgwh0QnHpNn8bOv5OSmGqjPoGnzXDCIiF9BrKqmuWmR
IiIiIiIBHwmkYiIiIiIiARMEomIiIiIiEjAJJGIiIiIiIgETBKJiIiIhIwCSRiIiIiIiIiIBE
wSiYiIiIiISMAkkYiIiIiIiAQagg6guSgvlyErK1/VYRARAGNj/VfagHhrJQjwHSU4ZZZnQJ5eY2W2X1zHAh1ClqCEH48iN
69u2L4OBgdO7c+Zl9v//+eyxatainT5+GhGCkpKQNaAARiCAK3RrCQ0PrFSPnIKKmpaRsn
oeaA8xBR09KY8xCTRCCJq9cLcwrB//36SWKbCbjY9vYkGb4CqITlp
fK8vDykpqZi69atLk3F8atsLe3F8q1tbUbJX4iCqSZWUlCAiIgKthsTlpkTUqpWUlCAiLGL+/v7w
yMDERGRtbYTyaTITAwEvHr1i3Fx3Cz4NhY+KOv3/ruPBAREVHzwy6wPBAxBqLhz
nw93dXSgzMDCAnZ0dL68WGO/LVu2oS0FHPnzq1S19xbA+AGNjYxgYGDRKzRER+ESNiahzVCH
S09PBwCYmZkplJUJuymCAtLa3aPteuXNERVM/+++47ZGRkVm/desWHV14e/vj8+fP8Pru8PIYAhjix
ozB1KlToabbGz+aIioaWOSSEStWmFhIQBUEfZQS0sLTk5ph5VdoxFBRg0aJFWLRoJKKpNEh
MTE5GbmwsfHx8sWLAAMTExCA0NRU5ODhYuXAFjvWDU0mGASERFR42OSSStWmuVmMiUiQlJQoby
XF0NHR6dK+xUrVqfWDwcXZ2NggPz8sPhPPTAwdY2NggPz8fmzdvhr+/v/73uJqqpiWBo
2EbpfkREREnTkYpJIRK1a5TWJKhFvHU8lVvp3J+8SFsPyJyQkAUF5eDDeDyNE5
Prrr+PTTz+FpqYmNDU1U1Ffr16NEDBQUFyMghodJxxmyRySKUFSvcjosbbBD22IqCVjkkhIe5

SPb04lAgAmvdodZu35Dx+1Hra2ttDT00N0dLSQJEqlUty8eRO+vr5V2v/8888KX1+9ehWBgYH
46quvYG1tDZlMhldffRXjxo3DvHnzhHbXr19Hhw4d6pUgViork9W7b1PHeYiIVIlzEJEiJomE
facT8dedLADA/tO38f743iqOiOjFEYvF8PX1RWhoKIyMjGBubo6QkBCYmprC29sb5eXlyMrKg
r6+PrS1tdG5c2eF/pUb33Tq1Ant27cHAAwbNgzbt2+HlZUV7O3tERUVhe3bt2PZsmUv/PqaC8
5DRKRKnIOIFDFJJKQ9fLKELfVhvgojIVKNgIAAlJWVYfny5SgqKoKrqyvCw8MhFouRnJyMIUO
GYOXKlRgzZkydxvvvvwww9hYGCANWvWID09HRYWFli2bBnGjx/fyFfSfHEeIiJV4hxEpIhJIhG1
eurq6ggMDERgYGCVOgsLCyQkJNTY183NrUq9hoYG5s2bp7DclIiIiKi54H7qREREREREJGCSS
ERERERERAImiURERERERCRgkkhERERERQCJolERERERQkYJJIRERERERAiaJRERERERJG
CSSERERERERAImiURERERERCRgkkhERERERQCJolERERERQkYJJIRERERERAiaJRERERER
EJGCSSERERERERAImiURERERERCRgkkhERERERQCJolERERERQkYJJIRERERERAiaJRERE
RComk8mwfv16eHp6onfv3pgxYwbu3btXY/sbN25g2rRpcHJygru7Oz766CNIpVKFNidPnoSPj
w8cHBwwatQonD17trEvg4haCCaJRERERCoWFhaG/fv3Y8WKFThw4ABEIhFmz56NkpKSKm0lEg
n8/PxgaWmJI0eOICwsDLGxsViyZInQ5vz58wgMDMSkSZNw9OhReHh4YP78+UhKSnqRl0VEzRS
TRCIiIiIVKikpQUREBPz9/eHl5QVbW1usW7cOGRkZiIyMrNI+JSUFnp6eCA4OhpWVFZydnTFu
3DhERUUJbbZt2wZvb2/4+vrC2toaoaS5Ysgb29PXbt2vUiL42ImikmiUREREQqFB8fj/z8fLi7u
wtlBgYGsLOzw8WLF6u0d3Jywtq1a6GhoQEAuH37No4cOYIBAwYAqFi6GhsbqzAeALi5uSEmJq
YRr4SIWgoNVQdARERE1Jqlp6cDAMzMzBTKTUxMkJaWVmvfYcOG4e7duzA3N0dYWBgAQCqVoqC
gAKampkqP9pV4nUV0xSSQiIqIGkfYoH9+cSgQATHq1O8zat1FxRM1DYWEh
AEAsFiuUa2lpIScnp9a+q1abmL2vj8vTGTo2buhwk
lJ43VKvk6iumCQSERFRg9h3OhF/3ckCAOw/fRvvj+t4oiaB21tbQAVzyZWvgaA4uJi6OjrJ
rXwcEBALBhwwZ4eXkhMjISXl5ewnhPq8t4tZHJ5JBKC+rdpvxsVh9Ok+adNyYS1YXrhxmyd6tQNsZHTFwpjWilDyl
PEmUyGTZu3IiDBw9CKpWib9++CA4OhGPYSEhODatWvQ0tLC0KFDsWjRIhgYGAht
Tp48iQ0bNuDBgwewsrJCYGAgXnnllRd1SURERK1S2sMnCUTqQ77JrqvKZaYSiQSWlpZCuUQig
a2tbZX2SUlJSE5OFpJBoGIpadu2bZGRkYF27dpBV1cXEolEoZ9EIqmyBFVZZWWy5+rfVMnliq
9b6nUS1ZXKF1xzy2ciIiJqzWxtbaGnp4fo6GihTCqV4ubNm3BxcanS/ty5c1i4cCHy8vKEsvv
37yM7OxvW1tYQiURwdnbGhQsXFPpFR0ejb9++jXchRNRiqDRJ5JbPRERE1NqJxWL4+voiNDQUp
p0+fRnx8PN5//32YmprC29sb5eXlyMzMFJ41fOONN6Cvr4/AwEAkAkJiYiJiYGAQEBcHR0xKBBg
wAAfn5++OGHH7Bjxw4kJSVh9erViIuLw7Rp01R5qUTUTKg0SeSWz0RERB5qAxo4di+XLl2
PixIlQV1dHeHg4xGIx0tLS4OHhgRMnTgAADA0NsXv3bshkMkycOBHz58+HnZ0dwsPDoa6uDgD
w8PDAF198gX379uHNN9/fPnsWXLFlhbW6vyMomomVDpM4nNactnoOVuh8xtn4mIiFRLXV0
gYGBCAwMrMrFJnYWGBhIQEhbIuXbpg6ZbBpg69attY45evRojRo1Sq3ydYJnPZ8hlo2dshc9tnI
iIiIiKqpNIksbls+QhUmic1py2eg5W6HzG2eiYiIiIqJWpM4kce+krUdHmVtmHsBUSiAU4xYNZ+s
TUFMhkMqxfvx6enp7o3bs3ZsyYgXv37tWp7/f8b/fffw8bGxsUHZT5JPjCnrlJ+88eRI+Pj5wcHDAqFGjPb
s2cYInYiIiKjBqTRJ5JbPRNQUKHNe69NSUlLwEYHTUTnKl8G0tu+UxEqqTsea2V
ZDIZAgMDYW9vX6WO57USERFRc6bSZIbvlMRKr1rPNaR44cWW2/LVu2oLS0FAsWLMD58+eF8
srzWpcuXar3Q3s3Nrdakk4iIiIkipqHeSmJSUhD/++AMSiQRTpkzBgwcPGcMiYiai/qc13rt2j
VERETgu++Q0ZGhkJdY57X2pClPdf45ERC2D0klieXk5tXDcejejJsJEIowYMQK
bNm3CgwcPsHfv3bZSZSI6EVQ9rzWgzWGoICLfq0CIsWLYKVlVWVWJLGxzmtt6RE/RyKi
lkHpJHHz5s11Z5s34/vvvvsWLFigr3/+98NHigRUWNQ9rzWgzWGoInQnSQeOnQIAqXS1Bi
6U29raIiAgAKGhoQ0aBaIAQEBFRY1L2vNvNZDbDyckJAIR58PrrrqNPwmD75pNHOa23JZ5j
yvNaWgT9HIqKWQekk3ke1dR07doRgbc2vTBIrz2v19fWt0v7xdpqqtc3t1b7nn39vWW
+Prq1asIDAzEV199eW81nHjxgnteF4rERERNRdKJ4mdO3gLL1epu3DhAjp37twwgg
RERvQhPn9dqZGQQEc3NzhISEKJzXmi0FbhISE4XE6zdnX6EuzaBfp0jcK8c5yFy3/OZ
6vvPIKDh06hLi4OHz88s4OHz+9V8dkjhxGbgASiXdv3j1ER0cjIiK
iyrbvRERNXUBAAMr+KyrbqwMrKyrbHHjRuHrKwsbNmyBfv27Tqwg28oOImIGo2y5
7U+zc3Nrdp6ntdKRKQGSSJDhUJ7tLO33vvA3NGFwHGiVCiGSTVW0I+ioQ+PbsNrMrqwKsqdi
IiohelXncS33vvPfj4DwMDw0MMeDwNDRsjFiIiIiIi4KwsAR4lVUVFmqK1D4
nYvXq1Th//jyysrIiSaWXmx47dgypqZ7gHhVPr0ySFIcTg4E
HGXlMuHrgqJSJKXkoGsnA4hEIhVGRkREylI6SXz99dcbIbcIGiIIqp8xD+qxy8kuEssKSc
ny+5xKsTPUxc2RPmBvrqTBCIiJShtJJ4oICxcojDiIiImqGUjjLzsHJvLAqKy6qtv5uei5V7Yx
Hk68xEkYgaXdqjfHxzKhEAMOnV7jBr30bFETVPSieJUoLDhw8jOjqaUqUhoaGGCFxeMGDMjEFxwZ
vvgktLa2GjpGIIaILlcjvAf4mpMECsVFJch4kQclk9l4dJTImpU+04n4q87Ffum7D99G+N+rP8FfuDmUm9k1n8D++P

763iiJonpZNEqVSKqVOnIj4+Hp06dYKxsTHu3LmD48eP4+uvv8Y333wDfX39xoiViIiImpC/U
6W4m55bp7Z30nLxd5oU1p3aNnJURNSapT0sEF6nPsxXYSTNm9K7m65Zswbp6enYu3cvfvnlFx
w4cAC//PIL9u7di0ePHuHLL79sjDiJiIioiYm9lalU+8u3HjZSJERE1JCUThJPnz6N9957Dy4
uLgrlLi4uCAgIwM8//9xgwREREVHTk51bjP/+cQe/xCYr1a+gqLSRIiIiooak9HLT/Px8vPTS
S9XWvfTSS3j8+PHzkRERERNjEwux807WfjtSiquJD6ETC5Xegxdbc1GiIyIiBqa0kli165d8
euvv2LAgAFV6k6f6o3OnTs3SGBERERSkejn5Jfj9WirOXk1F5uOi5xrLqUeHBoqKiIgak9JJ4s
yZM/HBBx+gpKQEo0aNQocHOfDw4UN8//d27d7FlyxYcPGQQMU/JmKxGPPn
z8eECRMaPEgiIJqfHmFpfjjjehrOXElFelZBje3aaGvg5V5m8m0OrTCXK5vNZzEgFAV0sDM3x68
vgLIqJmol7nJL777rvw9fXFlStXkJOTg7Zt26JPnz4wMOAnhERERM2JXC7H7H7ZQc/HY5BRfjM1
FWLquxbTfztvDq0wmutiYQa6oL5UG+zgj/Ia7a4zC6mOljhk9PmBvrNUr8RETU8OqVJP73v/9
FdHQ0Pv/8cwBATEwPgIUWLyIL7/8kokiERFREySXy3E3PRe/Xk7BPwFbGK6vtIi5NcLBfFs0J
sjEkiERFRE1JYXIbouAz8djkF9zPyamynOptDgATw8PKqt
8/DwwNdff/3cQREREdHzu5+Ri9q/ucwTp7nLOm3QSsVGX+ZumIoDeHHqCgPlwaLSUUUZlTmtwUQ6
zfi4MGDkEql6Nu3L4KDg2s8WiwxMRUq1NTU4Or/rqiqVLl6JTp05Cm8GDByMlJUWh36
hRoxAaGtqo10JEzZ/S/xKYmJjg2rVrcHd3r3J1J38+ZNGBoaNkhgREREpLzi0nJciMvAmSup+Dt
VWmM7TQ019LM1wUAnc3TtxOcGVS0sLAz79+Mz4fY4kA4fzVUlhsyU1hPEqp1sZ9zFFRs3boV9v2vdv2Q19tbe0XfWlE1Awpn
SSOHj0amzdvRps2bfDqq6/CyMgIWVlZOHXquXDzT4YUU6c2RpxEREREUi5i5TmPPPx2JRV/3khYS
3HUZi118XAPubo38sUejqaLzBCqklJSQkiIiIQGBgILy8vAMC6devg6emJyMhJ5UqH9qVOq9nUFhYiFWrVkufs8SSpwmef
fYYVK1YI5XK5HMOHD4ZDzZW4z4F+cm6fj5eDmlOHMm2Mg/zVhuqSQn2Ah6ki/b16+SYAsCz
a5hExMfH4/8/8/HyFVVoGBgaws7PDxYsXqySJfv3x6RxWhM4/QE8Wk5ORW/BwkJCTA2NmaCSET1on
SSqKGhgbVr12LevHm4dOKSHj9+H19/ffrtre2ANFSTDQRMERE9JT2rAGeupO
JOx14OXXCAAczGOiKa2xhqgWWeng4AMDMzUyg3MTFBWlpalfWLBQKNu6dSu0tLTg6uoK
ALh16xLhx6xZ0dXXh7w7++m+ry5cw8PpZL+OnhJpRhbPNHPNVYlCQoNfcPHXXOHfHDAIIeg6NBVHBsj
k+nd+/eHd27dwcAZGZzmQi0KRoLy8HOrq6s/oSURmOHRAE5BBXH3smtsp64mQp/uHT
DQyRw9OxtCjXcNm7zCwkIAqPLsoZaWlnBnsDa7d+/+/GN998g6CgIRLv3x5AxcY2ubm58PHxwYI
FCxATE4PQ0FDk5ORg4cKF9YperTPQ8M29erb1KmpiRRet9TrbA34s2wYSieJ+fn5WLFiBezs
7DBlyhScOHEC0HECixcvRn15OaysrBAREVHlkzAiIiKqn8zHzLiFpCHEaLwpwyAcKlHaA4mhNc+SpJRr
qejGdrpVV2GSE1X5WYyJSUlChvLFBcxQ0dHp8Z+crkcxX375JTZv2oy5c+di+vTpQt2HTTtQOHTtqXF
wMPT09AICNjy8xb+y/tuokwmh1RaoHS/5kAmkyu8zs7OV2E09Dxa08+yMRNgpZP
E0NBQ/PTTTTxgwYAAAYM2aNC1tcW8efPwn//Ghz/Bo8VizZk2DB0pERNRalMtkuuHb7EX69koK/
/s6CvIZ2IhHQ27oDBjp1Qq8u7RU+AafmoLDdYlEAktLS6SSGcIpHU+ChpaWkpgpKCcPzcSev
BgzZ85UqNfU1lU1ISmpuLGRD169EBBQFycnLqvRt9WZmsXv2aOrlc8XVLvc7WgD/LhqF0knj69G
ksXboUr732GuLm4pCSkuJUSYuEu4pCSkoLFixdjyJAhKCsrBX7+Eu4BZucU1tmunJ8YrvrvTv
hld6dYGTAIw2aO1tbW+jp6SE6lqEqVSKW7evEAlfX99q2kx4Xn31xfvTY11tZDIZXn31
VYwbNw7tz6s0Tyq9fv44OHTrIsealk8XFiFYZs2ZZZ0Y1tZHbdFi45fnS45n/Ui
IiIJSFMJseNO4/w2+VUXE16qPAp+NEAOy7GmFQH3M4dmsP9efYfISaFrFYDF9fX4SGhsLYA
jm5uYICQmBqakpvL29UV5ejqysbysqLOjr60dx8WWxuHDx8WHvfp68fMJSaFrFYDF9fX4SGhsLY
V7O3tERUVhe3bt2PZsmUqvFIiai6UThLNzc2RkJAApVIJNVvmtsiIiKi
qnLyinHuWhWOxXuEdMdDXh+c9dQ+N2NT+fRs1bQEAAysrKsHz4cEgkEri4uGDIkCGIiopCYmIi
8VITk7GkCFDRVDbGu5hgDAwNCKoX2nrtY3XQGC2GdzgpImXgqDAtzcHXkuBdmeCqlrOOr48a
B+/HhVXB4RNTNKJ4mTJk3wSO2yWOwQm3nlmYOv4fQFZbeZrYDqeqVL4T7M
dy8aZyym4nPgQ5bKanjYEenY2xEAnczh17wANdd41bOnU1dURGBiIwMDAKnUWUWFhZISEgQvo6I
iHjmeBoaGpg3b57ClMiorpSOkmcMmUKjIyMcOHBfzx6gwYUKDB0lER
NScSQtK8Mf1iruGkuzCGtu10daAh6MZvPqYw9RI9wVGSERE9ES9zkkcOXJ1kYek161b1yABER
ERtQRyuRy3HjzGb1dScSlBgrLymu8aG1Yb5mTz2aPRLokUwMDAQ2gwePBgpKSkK
RBw8ehFQqRd++fRECHIzOnTtX2/7GjRsRsICQnBtWvxyFaKCWlhaGPGBWPBgpKSkK
/UaNGoXQ0NBGvRZVSfyiUvx5PR2/fjUjRqZ8X+FX9n3r1aHkXd7gE7x1gEFE86hQzOXDuD
r2wsDDs378f4s77f1euRMeOHRES0TTzz2fj+PHjEIvFCm0lEgn8PwfPLJ8jKysJHH32EJU

uWYPPmzQCAvLw8pKamYuvWrbC3txf6Pn1INrUscrkcSalSnLmcggvxEpTWci5X104G8OrTCf1
6doSWJu8aEhE1FLlcjrLyJ/NvQVEpklJy0LWTAUQiniOrDCaJRNSqlZSUICIiAoGBgfDy8gJQ
sXze09MTkZGRVZbWp6SkwNPTE8HBwdDQ0ICVlRXGjRunsOT+1q1bkMvlcHZ2Vri7SC1PYXEZz
v+Vjl8vpyI5M6/GdlpidfS3r7hraNlR/wVGSETUOqRk5iH8hzjk5JcIZYUl5fh8zyVYmepj5s
ieXLWhBCaJRNSqxcfHIz8/H+7u7kKZgYEB7OzscPHixSpJopOTE5ycnISvb9++jSNHjgjnxQJ
AQkICjI2NmSC2YHfTpfjtciqib2aguLS8xnaWHfUw0Mkcbj07QkeL/+QSETWGlMw8rNwbi4Li
smrr76bnYuXeWAT5OjNRrKN6/4slk8lw69YtSCQSODs7o6ysDO3atWvA0IiIGl96ejoAwMzMT
KHcxMQEaWlptfYdNmwY7t69C3Nzc4SFhQnlt27dgq6uLvz9/XH58mUYGRlhzJgxmDp1KtSe4w
B0DY2WewzC06uARKKmea3FJeU4/1c6folNwZ00aY3txBpqcLc3xSBn81a3xKk5/ByJqGWRy+U
I/yGuxgSxUkFxGSJOxGH5VJdWNS/XV72SxGPHjmHNmjWQSCRQU1PDwYMHsWHDBmhqamLNmjVV
nuEhImqqCgsrjiP433lLS0sLOTk5tfYNDQ1FUVERQkNDMXXqVBw7dgxt2rRBYmIicnNz4ePjg
wULFiAmJgahoaHIycnBwoUL6xWnmpoIhoZt6tW3OVBTEym8bkrXejdNih+j7uLXSw9QUFTzmx
BLU32M6G+FgX1fgp6O5guMsOloyj9HImoZZDI5HkqLkJFVgIysAtx68Bh303Pr1PdOWi7+TpP
CulPbRo6y+VM6STxx4gSWLFmC119/HYMGDcL7778PABg6dCg++eQThIWF4b333mvoOImGkXl
ZjIlJSUKG8sUFxdDR0en1r4ODg4AgA0bNsDLywuRkZEYPXo0duzYgeLiYujpVSxpsbGxQX5+P
jZv3gx/f/963U2UyeSQSmveKbO5kz11qLxMJkd2dr4KowFKSstxIS4Dv8amIDG55g8LNNXV4N
rTBIP7WqC7RVuIRCKUFpUgu6ikxj4tWVP7OTYmJsBEjUcml+NxbjHSswqQkV2IjKwCSLILkZ5
VgMzHhSiX1Xyk0LNcvvWQSWIdKJ0kbtmyBW+//TY+/vhjlJc/eQ5jzJgxePToEb799lulkkRl
t55PTExESEgIrl69CjU1Nbi6umLp0qXo1KmT0IZbzxNRXVUuM5VIJLC0tBTKJRIJbG1tq7RPS
kpCcnKysMkNULE0tW3btMCiuSGGo5Sjx49UFBQgJycHBgaGtYr1rJadsxs7uRyxd
equta0R/n47XIq/ryRhvxa7hp2NNLFwD6dMMDBTLhrWF4uB1D/Ny4tgWl7XTzMKQIAmLVv06J
/Z4no+cjlckjzS54kgtkFkGQVIj27IiGsbZfo51FQVNoo47Y0SieJd+7cwZIlS6qt6927NzZs
2KDUeMpsPZ+RkYEJEyZg6NChWLx4MUpLS7Fp0yb4+Pjg0KFDaGlpYejQofj8888FnymeMmSJfr16zNmpoWi2a0yQvL+sbwybWO0H4ntG1XuzFUgYDzi0JjbTUqJxD5h2WJtyaXJ5eBmqjHR6k5vevv2UzzTlqlrOnqOrt
2KDUeMpsPZ+dnQ0/Pz+4urpi7969KC4uxr///W/MmjULR44cgZaWFreeJyKcqWplNraQk9PD9HR0
UKSKJVKcfPmTfj6+lZpf+7cOfznP//B77//ifzdzWfcbHV0LyqvRZMtjRIjeb2dpzf5/9t/jn4jFwE9GfLEWpJqhapQLZ7b7mk4hq7f16x48yEbqe4ys/MAHsMoWH+QE7x0h4/37DTVFI+r2fYD4J
R+169fR4cOHeqdIFLjKS2TIfZ4gaHJd0bl3+qUqpGbLtUuR
A4O0h3VQcDRE1BXmFpRWJ4D/JoCS7QEgMi0tq3virsehqt87HAZSldJLYvn17JCUlKezkVykp
KQnt27ev81jKbj1/6tQpFBYW4tlEgO4zqfKwljk81E2gr6Ve8Egd9Dbl9hDdXOMvLQLYYYGpzI5B0Ybyf6jv2jf2Ax5XXqPfPRuvFUMGexqQPA1Z24tTKkL
BRGRkYwNzdHSEgITE1N4e3tjfLycmRlZUFfXx/a2tp44403EB4ejsDAQHzwwQfIycnBihUr4O
joiEGDBkFNTQ3Dhg3D9u3bYWVlBXt7e0RFRWH79u1YtmyZqi+XniLZS6K6k4dy0NeYU1f7L
coa02vPp0godjJ7Rtw2fua2PWvg0+nNBH1WEQ0QtWUFSGjOwChbuBGVkVCWFtqzKUpSVWR0dD
HZga6cLEUBcdDXVQVi7Drh8T6jyGU48ODRZPS6Z0kujj44P169fDxMRESOxEIhFu3LiBsLAwv
Pbaa3UeS9mt5z09PeHp6Sl8vXXrVmhqasLb2xsAtp4norqrnWYmRlWrz4yVTwvHe1+PCWTyrb7xSWnhgYWSrgRsZJFtJgFpM1ScuQUQ3nEZuq8l66Hf1TU2eu6FmscjOh18S7WkMDQrQlvv4+D0H3CVDR5mJ+O2Ilf8lxdPvkK/IKIaiFPsx8kz2UMfC1aTvGCX8y4UA+mp68L4Ig7hVq5C9xhqNyXxRRuFNxSKbkwaBUdyU3cUOyGXcKz9KAb+7n7RcjVYuzeTv/bN2YtsY+ryOmyezUVujbvN3Kh7hP33iYYCcGZtH7DtPN3Z4ZNeVqndoA3P+2X1mbF53rzrbxQIcvbK+0
Jo7tLnpP2vPCcHBAVtYAcoXmQHufZfjgtzCcbxGI3oKRvIiU9Rg9o9/YJ6f2Uz0x33/k9MqNTRLmFu3k9y7k1R0Du4uPcvZbuKyjxrPUKqQ6JnH1BLKxEX2f1rsvuFTQ/4bwNr9XcTQN3a1PB/GKmVWwuwdXd+j2dVE0Qz0ADDJ7aOR0E1ADSH5QgXRJ4aCmi
hWCtvU3F5wVfRa2/0e0AQZ8DCqo

TJgwATNmzMDHH3/8zPGU2Xr++PHjAIDVq1dj9erVCuNUtuHW88qRy+UKyxEKikqRlJKDrp24d
puIXoy6zkN5haX443oafruSioysmp8Rb6OtgQEOZvDq0wlm7fn8KhHV7kW9FyoqKauaCP5zh7
C2s1qVJdZUg0k7XZga/bMk9J9dQzsa6kJfV5Pv76hOlE4Ss7OzYWRkVG1d27Zt8ejRIwCAkZE
RCgqevdGLMlvPR0REPHM8bj1fdymZeQj/IU5hO/jCknJ8vucSrEz1MXMkd4Eiosb1rHlohk9P
FJaU4bfLKbgYn1nrMzbdzNvCq08nuNqaNKlnZIio6Wro90IlpeWQPC6sNhnMyav5+B1laaiLh
MPkOxrqwsRIB6b/3BVsp1f1LEEiZSmdJNrZ2WH79u3o37+/wtEVJSUliIiIQM+ePQEAf/31V5
XzD6npSMnMq/U8mbvpuVi5NxZBvs5MFImoUdRlHgrecaHW7dh1tNTR394UA/uYw8KEcxUR1V1
93wuVlcuQ+bhQYUloRlYBJNkFyJIW13jcjrLURCJ0aKcN03/uBnZ8apmokYG2whFmRA1N6SRx
0aJF8PPzw+DBgzFw4EC0b98ejx49wpkzZ5CXl4ft27cjJiYGa9eu5d28JkoulyP8h7haDxwFg
ILiMkSciMPyqS78RIqIGlRd56GaEkQrU30MdDKHW8+O0BLzriERKUeZ90LrDl5Fn24d/jlkvh
APc4pq3UFZGSIARgbaMDXSgck/S0JN/9k8pn1bbe7ATCqjdJLo5OSEw4cPY8uWLTh37hyysrJ
gamoKT09PvPPOO7C0tERUVBQCAgKqHE9BTcPfqdJqDxqtzp20XPydJuX2wUTUoJSZhyppaarD
za4jBjp1gpWpQSNFRkStgTJzUJa0GL/EpjzX9zPU10JHQ52KJaL/LA01MdKFSTttaGrwgy5qe
uq1n23Xrl2rbBzztP79+6N///71Dooa1+XEh8q1v/WQSSIRNShl5yH7LoZ4d7QDt2Enogah7B
xUF/q6mgoHyj+9TJQrHqi5qde/trm5uTh//jwKCgogr+Z2++jRo583LmpEBUXK7aClbHsiomd
Rdl4xbstzuoio4dT3vY2uloZwZERHw6f+a6gLXW3OUdRyKP3bfObMGbz33nsoLCystl4kEjFJ
bOJ0lTwHh7sEElFDU3YeUrY9EVFtlJ1T+tub4u0h3aCnwyMkqHVQOklcu3YtunbtiqCgIHTs2
BFqanygtrlx6t4BJ87fq3P7qL/S0bOzIXp369CIURFRa6LsPOTUg/MPETUcZeegwX3Noa8rfn
ZDohZC6STx77//RlhYGFxcXBojHnoBunYygJWpfp0f2M4tKMWX312Du11HvP1qdxhwkiSi56T
MPNTFTB9dzbhRDRE1HM5BRLVT+jZgp06dkJeX1xix0AsiEokwc2AS52RP6Cr5fM/5mxlYvi0a52+m
V/ssKhFRXdV1HtLV0sAMn55c3kVEDYpzEFHtlE4S586di02bNiNiEb6bkNiE5Obkx4qEXxNxYD0G+zrAy1
a+2vouZPmb42KKjoY5CeV5hKb76702s/+4asqRFLyJUImqh6jIP/e8h1kREDYVzEFHNRHIlbw
n5+fnhxo0byMvLg5GREbS1tWRUw6tSpBg2yKSgvlyErK1/VYTQ4uVyODzb+gZz8EgCAjlg
dH7zdB13NDCASiVBSWo5jf9zBT9EPqhwcq6OljnGDuuGV3p2gxk/Y6AUyNq7+H/SWrKXOQcCz
5yGipojzUMvBOahlCQz7E4/+uZHR3kAbIe++rOKIGk9jzkNKP5NoamoKU1PTxoiFVEAkEkFD/
ckNZV1tTYUzEcWa6hg3sBtcbU2w40Q8HkieLDUuLC7H7h8TcOFmBqaNsEVHQ90XGjsRtQzPmo
eIiBoT5yCiqpROEleuXNkYcVATZ2VqgH9Nc8GP0ffx3z/uoKz8yV3F+PuP8VH4BYz27IKhri9
BnTveEhERERE1W/V+N//w4UOkpaUhNTUVqampSE5ORmJi29eQ8VEToqGuhtdetsInM/qh
m7niJ2ylZTIc/DUJK3Zfwv2Muu2aSkRERBVkMhnWr18PT09PT9P9P9O7dGzNmzMC9ezUf0CYmIg5c
+bAzc0N/fv3R0BAAFJTUxXanDx5Ej4+PPjePjA09PTw
wePBhDhgzBkCFD4O3tjddffx2fffZY8RJTYhZ+zZY6uuMyd49oKWprlB3Lz0Xn+2KweGzf6O
0TKaiCImIiJqXsLAw7N+/HytWrMCBAwcgEokwe/ZslJSUVGmbnnZ0NPz8/tGnTBnv37sW2bduQ
nZ2NWbNmobi4GABw/vx5BAYGtKkSTh69Cg8PDwf/58JCUlvehLI6JmSOkkcfXq1ZBKpViyZ
An69esHDw8P/Otf/4KXlxdEIhF2797dGHFSE6MmEmFIXwt8NqsfenUxUqgrl8l8lx/M+7+HjHBd
xOyVFRhERERM1DSUkJiIii4O/vDy8L9ja2mLdunXIyXIyMhAZGRklfanTp1Cq1ahe7du6
Xr14ICQlBUITYmNjAQDbtm3fH19YW1tfY0V1tjSVLLsDe3h67du160ZdHRM2Q0kni
QkyfPh0jR45ER45EQUEBBJk2ahC1btm2Dt1GiNOaqI6tNXB++ ++N7Y+bInmijrfiIa9qjAqzcc
wnfnLqFopIyFUVIRETUtMXHxHxyM/Px/u7H4sUq7fv3749/3749NmzZSS0urTh/Tfbrs
5kMhliY2MVxgMANzc3MTENPwFEFGLo/TGNSUlJejSpQsGoGvXkhISBDqxowZg+4IaLjpo
FkUiEAQ5m6NW1Pb6OvIWYeIlQJwdwKiYZl289xLQRNujVpb3qAiUiImqC0tPTAQBmZmYK
JkhLS6vS3sLCAhYWFgplmzZtglgshouLC3Xk4xKSUXWFglBJMvkWWcdXXgSVSl5FQUFBLN/qaxlOGhkbL3Jzu6ZMuRKKWe52tA
X+WDUPpJLFTp0548OABXFxc0LlzZ+Tl5SE05ORkWFhYQi8IyeHywtaqbRsx3h3dC5cSMrF
ThvCEAeCQtwtoDVzHAwRQTBneHno6mCiMlIiJqOgoGoLCwEwYrYvFxRvxLS6t076b2796NbF
BQWjfvr2QdFY3XuQuzDXuUzi/WhpiaCoWGbevdvytURAqvW+p1tgb8WTYMpZPEoUOHIjQ0
fDi6du2KdevWYc6cOYiIiIgtra...hi2ndvh219u49w1xU8s/7iejut/Z8HXuwdcb
E1UFCEREVHToa2tDaBitVblawAoLi6Gjo5Ojf3kcjm+m+/PJLbN68GXPnzsX06dMBQFiG+r+b3j
xrvGeRyeSQSgvq3b8pk8nkCq+zs/NVGA09D1MjXUiyC4XXLfln2ZgJsNJJ4oIFC3Dy0n
Iw4cPR1BQEBYsWIATWIATJ05AXV0da9eubw4qZlo60JP5+ecLPriJ0n4/Ewp0ik+aXIOozoDfTt
YYzJQ3ugnV7VZyqIiIhai8plphKJBF27dhRez5w5s4q22dnZCCgRKw3+m2/gZ62KL
65dO+jq6k6IkSj0kUgkVZagKgesp7VEXZmY+n/9oLBCUQIoRA/9aRBcicMeZZZuZZtP0sWnp3yfNapwj5G/bPAVXG/
79epSWlgIAPD098f333333+Ovv/6Cvb29wuRGZ8uuRGZGdlhM9muuHIub8RGfNAYRK+dCsTcfey8faQ7hj
gYArR04vIiYiIWglbW1vo6+sjOjoanTtXfOguk8kQHx+PqVOnVuv/wQcf4PPPPEdsbGxwbPnzp96FL7X9++PLL7/EhAkTAADS0lL8+uuvGDVq1H
BGdnZ1y4cAHjxo0Tyo9G9G3b9/GuxBp/w+aagPadN+sjzNOsDuhNqpjsnDVS0XmOvnLv6ioXkNazTfSsqpwPWyK/LCAIg0
6yOt4d0h2tPE+w4EY/Uh09u+xcUlyHiRByib6Zj6nBbGLer/zIYIiKi5ii5Eb+5kgsFsPX1hXxWxehxYoEwv0wsDpF7b8t9GnV18/z4Dl1Ki6Vx0hyzUr/HjhuLzvkLyQ==

KCubk5QkJCYGpqCm9vb5SXlyMrKwv6+vrQ1tbG4cOHceLECSxevBj9+vVDZmamMFZlGz8/P8y
ZMwd2dnZ45ZVXcOjQIcTFxeHzzz9X4ZUSUXOhdJJYVFSE9evX4/z588jNzYVMpngLVyQS4dSp
Uw0WILUc1p3aIni6K36Iuosfou4JSwEA4K+72fhXeDTeesUaQ/paKDx0TERE1NIFBASgrKwMy
5cvR1FREVxdXREeHg6xWIzk5GQMGTIEK1euxJgxY3D8+HEAFWdXr169WmGcyjYeHh744osvEB
YWhnXr1qFbt27YsmULrK2tVXF5RNTMKJ0kfvHFF/j222/h7OyM7t27Q02N28pS3WlqGG0Z1e
42Jhgx8k43EnLFepKSmXYdzoRF+IyMN2nJ8w7cDcqIiJqHdTV1REYGIjAwMAqdRYGIjAwMAqdRYWFgpHjkVE
RNRpzNGjR2P06NENFSIRtSJKJ4k//fQT/P39MX/+/MaIh1oJCxM9LJvigsiYBzhy9m+UPPQc
VKqFJ/suIDXXraCj3tnaKjzgwgiIiIiohdF6XffpaWlcHFxaYxYqJVRUxNhWD9LfDqzH3p2Nl
SoKyuX4+i5O/h0ZwupElVFCG1FjKZDOvXr4enpyd69+6NGTNm4N6ezW2v3Hj7BqZNmwYnJye
4u7vjo48+gl8Sq+Ht68uRJ+Pj4wMHBAaNGjcLZs2cb+zKIiiIiGoTSSaKnpyd++23+Q5Ktk
Q10sersPpo+whY6WukJdcmYeVuyOwbe/3EZxabmKIqSWLiwsDPv378eKFStw4MABiEQzJ49u
8oZY0DFFvJ+fn6wtLTEkSNHEBYWhtjYWCxZskRoc/78eQQGBmLSpEk4evQoPDw8MH/+fCQlb
3IyyIiIiKqlzotNz169Kjw2t7eHuvXr4dEIkHfvn2hq6tbpT3Xv5OyRCQXundCQ5d22PPM
4cvuhUCeXAz9euI/YW5mYPsIWtv9z15HoeZSUlCAiIgKBgYHw8vICAKxbtw6enp6IjIssrV8
SkoKPD09ERwcDA0NDVhZWWHcuFYt26d0Gbbtm3w9vYWtq5duHTTz99cRdHR
EREVA91ShKXLl1apeyHH37Adz/8UKVcJBIxSaR6M6Ht9TXgv9bDrgYL8HXkbeQW1Aq1EkeF2L1vs
vw6tMJ4wZ2g652vU9wIRLEx8cjPz8mik5MTnJychK9v376NI0e
OYMCAAQAqlq7GxsZWmFnd3NwZiFdCRERE1DDq9C779On9nTjR0HkUAkEqFfz46wszLCvlOJ
iPorXaH+zJVUXL39EFOH2aJP9w4qipJaivT0it8vMzMzhXITEx0KpaXV2nfYsGG4e/4cuzM3NE
RYWBqDiAOyCggKYmpqCm9vb5SXlyMrKwv6+vrQ1tbG4cOHceLECSxevBj9+vVDZmamMFZlGz8/P8y
EADx48wK+//orRo0fDwMCgwYOk1klPRxOzR9nB3b4jdv8YjX9Yj0fSYqucV4J1h+6hn6hn49TTDJuwc
MdMUqjJSas8LCQgAVh1k/TUtLCzk5ObX2DQ0NRVRVFREUJDQzF16YMRUVFNY5XXFxc3TB1
oqYmgqFhyz0W5umzUVv6tRIRETV1Sq/Xu337NqZPW7eHx0xe5wt+sI0dO3I4jqQFtbG0DFs4mVrwGguL
HSc1Io5dG2PT2e64dCZJPwSm6JQdyFOgpt3szHx1e5wt+sI0dO3I4jqQFtbG0DFs4mVrwGguL
gYOjo6tfZ1cHAAAGzYsAFeXl6IjIwUnmv8301v6jJebWQyOaTSgnr3b+pkMrnC6+zsfBVGQ/R
s/CCDiFoypZPE1atXw9zcHBs3bhTK7N3dcebMmZ77rsICQnBl19+2aBBEuloacB3qA369
HSfjkZH15M1yXmEptn1/E9E3MzB1mA2MDLrGYlIUeUyU4lEAktLS6FcIpHA1ta2SvukpCQkJ
ycLySBQsZS0bdu2yMjIKRVFmCqqyyp84TbWnkcsXXLflaiYiImjqlH
q4cuUK5s+fD2NjY4VyyIjzJO7F9HR0Q0WHNH/6vFSwUj+3eG2v/cNbyW9AjLt0fj19h
kyJ5+x0lUC1tbW+jp6SnMXVKpFDv3qz2TNhz585h4cKFyMvLE+ru37+Plo0T1MbBv2ThXIXrdK
GRcuXFDoFx0djb59+zbehRARERERE1EKWTRJFIhz86pcBlHIZSSUoLS0S0tNo6ooaiqGa9t7ys8a9pL
rDsqKdQV1RSjj0/38Lqr2ORntVyl+ZRwxGLxfD19y8ChOnz6N+N+Ph4vP/RZ/Z8vP/++zA1NYW3
uRmZkpPGv4xhtvQF9GBhUhMTERMTAxCAgCAgLg6Oi
RFxeHadOmqfJSiYiIiOpEsjDmOE0kEkvNx/BBLwqLaps+Yw+Ls4ObmNbmJDwiQgdEEW+NS6w8a6iE7
1tBQV/xVvpWcg4/CL+DE+Xsol3HZGtUuICAAY8eOxfDwx4FdXBwB4eHjgiy++wL59+/Dmn4Dwc
IkTJwAAhoaG2L17N7N2QyGSZOnIj58+wL59+/Dmn4Dwc
JlC6ytrVV5mURERERER1IpLLlVuX9+DBA4wdOxYxYlcjJSXo06cPKw0MwxcKgGWdyd0Fpy1pw9
dGitelSkvlyErq2VupBAY9iceSSvukrQ30EbIuy+rOCLlpT3Kx86T8UhMrrobZeeO+vDzsYVl
R30VREaNwdi49f0sW/IcBLSMeYhaF85DLQvnIGqOGnMeUvpO4ksvYTjx4/j7bffRkFBAW7cuc
AGpVIoJEybg6NjLTJBpKbPrH0bLJnsDN+hPUUeou5eRi093xuDQmSSUa/ikV8RewHfaBTw1mSfd8tNcv5Ftg2TTv
VBUpERERE1IQpfSeRqrsTt3NZ8X6nSEk5Amo1ZJTe9fSqUIPQvD2P1uvPVhpSl2t0NwwmLsMrsp
JonUIolEIvTvZYoVs9zQ6xr8...

7Gz4+fmhTZs22Lt3L7Zt24bs7GzMmjULxcUVm42cP38egYGBmDRpEo4ePQoPDw/Mnz8fSUlJy
l4qtTLmxnr4P9++eHtId4g1Ff/X+DtVik92XMSx3++grFymoghrl/bwyWZSqQ95J5GIiIiI6k
fpJDEhIQFlZWWwtbXFqFGj8MYbb8Db2xuGhoZKf/OSkhJERETA398fXl5esLW1xbp165CRkYH
IyMgq7U+dOoXCwkKsWrUK3bt3R69evRASEoKkpCTExsYCALZt2wZvb2/4+vrC2toaS5Ysgb29
PXbt2qV0fNT6qKmJMNT1JXw20w12Voq/0+UyOY79fgef7LyIv1OlKoqQiIiIiKhxKZ0kXrx4E
V9//TUGDx6Mc+fOYcqUKXB1dcWsWbOwbds2XLt2rc5jxcfHIz8/H+7u7u7kKZgYEB7OzscPHixS
rt+/fvj02bNkFLS6tKXU5ODmQyGWJjYxXGAwA3NzfExMQoczZXU2hm308GHE/rAz8cWulqKmwC
nZObj8z0x2H86EcUl5SqKkIiIiiocSh9BIaGhgb69u2Lvn37Ysi5dw4MABrFmz
BiKRCHFxcXUaKz09HQBgZmamUG5iYoK0tLQq7S0sLGBhYaFQtnXrVmhpacHV1RVSqRQFBQUwN
TWt03jK0tCo1yOcTd7TpzyIRC33OutjkLMFnLobY9eP8biUkCmUy+XAzxcf4EriQ8wY2RN2XY
xUGGUF/hyJiIiIqCEonSRWevjwIf78809ERUUhKioK6enp6NSpE7y8vOo8R0vRmFhxfECYrFYoVx
LSws5OTnP7L9792588803CAoKQvv27Trry9Cw
DT6e8zL+uJaKLYev4XHuk98lyeNCrPo6FkPdOsNvlD30dDRVFid/jkRERETUEJROEr/44gtER
UXh9u3bUFNTg5OTEyZPnoyBAweie/fuSo2lra0NoOLZxMrXAFBcXAwAwdHZ0a+8nlcnz55ZfYvH
kz5s6di+nTpwOAsAz1fze9edZ4dSGTySGVFjy7YTP09PEOMpkc2dnc9KQ6di+1xRdz3PFN5C3
8fk3xzvTP0fdw4a80TBthi742JiqJrzX9HJkAExERETUepZPE77//HsOGDcMbb7wBd8uXhi8
7E41qFxmKpFIYGlpKZRLJBLY2tpW26e0tBQ6dLBtQxQiUUUifi9ejJkzZwp17dq1g66uLiQSicK4
URSZQlqfZSVNc1dLZ+XXK74uqVeZ0PQ1lTHDJ+e6Gdrgl0/JuCRtEioe5xXgi8PXoOrrQkmef
dA2zbiWkZqePw5EhEREVFDUDpJ/PPPP8+MW3F6uIRCIRoaGnv14enKQw
NmzWet7h48WJERkZizZo1GDlypEKdlypEKdSCSCs7MzLly4b51iIqqLXl3b47NZ
/XD4zN84fSkZT+VnuBgvwc27WZj4anf0tzet9f8XIiIiIqKmRukkksbS0FDt37sSFCxdQWloK+
T+3L+RyOQoKCnD79m1cvXq1TmOJxWL4+/sjLCwMxp06YMKCAsTEKCcfu3jAkbk+0cvzhFRHF/5z9
8f2traOHz4zME6cOIHFixejrX79+yMx8+OzYZw8Ms8uorMDIKr
SiWqlLdbAJO8e6NezI3acjjEPaoyfLkfOLyrD9eBzO38zA1GE26ND2+ZY7ExERERG9KEpvie
9WqsXbsWEokESUlJSElJQWFhIS5fvoz4+Hjs2bEG1pXv9+Du/MkRhIb6fcfd4uexorjdlV4Pkq+eqA/LnR7NPQeNvDO3XBB4KEBV0pt9ZCXw+hWYV6I1vj3Jw+xRW8+jv
nHWbPnZ+1O+HvCLdMvu5qOm0Vn7D6qGqtrn7MLt3ZtpMyjugvhEqyr9d2iYwFL/mlq7DpwlRSgoN2x2lfL5mL9Gbb3twVz1tYRuK7fGRIaHhsh26JLhtEeEsxYF/FDs7MvQxwCRdtkB3C+JCkyF6SNo8VGVvQ5WJDPvu/eSS3gcLAyBoGDcN1oirbyNR0Ra2wRLdVtGdNL6JAYIW

WWLVuG8ePHN/KVEFFLoHSS+MsvvzRGHEStnlhTHeMGdYOLrQl2nIhHcmaeUFdYXI5dPyYg+mY
Gpo2wRUdDXRVGSkREDU1dXR2BgYEIDAysUmdhYYGEhIQa+1b33kxDQwPz5s3DvHnzGjROImod
eAgMURPTxcwAH013wZuvdIWGuuJdw/j7jxEcfgE/Rt9HuYwP1TcUmUyG9evXw9PTE71798aMG
TNw7969GtsnJiZizpw5cHNzQ//+/REQEIDU1FSFNoMHD4aNjY3Cn0WLFjX2pRARERE9NyaJRE
2QhroaRr1shY/9+sHa3EChrqRMhm9/vY0v9lxCsqTibqNcLkdZ+ZOksaCoFEkpOdz0po7CwsK
wf/9+rFixAgcOHIBIJMLs2bNRUlJSpW12djb8/PzQpk0b7N27F9u2bUN2djZmzZqF4uKKnWrz
8vKQmpqKrVu34vfffxf+BAcHv+hLIyIiIlIaT+0masI6dWiDoMl98UtsMg6d+RvFpeVC3Z20X
Hyy8yI8Hc1wJy0XOflPEprCknJ8vucSrEz1MXNkT5gb66ki/GahpKQEERERCAwMhJeXFwBg3b
p18PT0RGRkJEaOHKnQ/tSpUygsLMSqVaugpVWxmVBISAi8vLwQGxuL/v3749atW5DL5B2doa
BgUGV70lERETUlPFOIlETp6YmwqsuL+Gzmf1gb6W4bXm5TI7frqTiXkZutX3vpudi5d5YpDz1
fCMpio+PR35+Ptzd3YUyAwMD2NnZ4eLFi1Xa9+/fH5s2bRISxKfl5OQAABISEmBsbMwEkYiIi
Jol3kkkaiY6tNPBBxP64M8b6dh/OhH5RWV16ldQXIaIE3FYPtWFO6NWIz09HcCTw6wrmZiYIC
0trUp7CwsLWFhYKJRt3boVWlpacHV1BQDcunULurq68Pf3x+XLl2FkZIQxY8Zg6tSpUFOr/2d
zGhot93O9p381RaKWfa1ERERNHZNEomZEJBJhgIMZenUxwtb//oX4+4/r1O9OWi7+TpPCulPb
xg2wGSosrDibUiwWK5RraWkJdwZrs3v3bnzzzTcICgoSzo1NTExbm4ufHx8sGDBAsTExCA0N
BQ5OTlYuHBhveJUUxPB0LBNvfo2B2pqIoXXLflaiYiImjomiUTNUFs9LXTt1LbOSSIAXL71kE
liNSoPpi4pKVE4pLq4uBg6Ojo19pPL5fjyyy+xefNmzJO7F9OnTxfqduzYgeLiYujpVTwLamN
jg/z8fGzevBn+/v71upkdgkEjVTBUWljdq+
tahcZiqRSGBpaSmUSyQS2NraVtuntLQUQUFBOH78OBYvXoyZM2cq1GtqakJTU1OhrEePHigoK
EBOTg4MDRWfLa2rsrKWe+zJ0xvxyuUt+1qJiIaOj70QdRM6WprPrvRc7RvLWxtbaGnp4fo6G
ihTCqV4ubNm3Bxcam2z2WcfR4cOHeqdIBIREnUyGwYMHY/PmzQrl169fR4cOHeqdIBIRERG
9KLyTSNRMOXXvgBPnaz7wvUr7HhVAvYPZDNhwmxXZ2wT5IfpRb29vlJeX
IysrC/r6+tDW1sbhw4dx4sQPJLF68GP369UNmZqYwVmWYcOGYfv27boCysoK9vT2ioqKwfft2L
Fu2TIVXSkRERFQ3TBKJmqmunQxgZaqPuZ+nVH3/xtC5m+uhqxuMYahIQEICysjIsX74cRUVFcH
V1RXh4OMRiMZKTkTkzFkyBCsXLkSY8aMwfHjxwEAq1evxurVqxXGqWzWz4YcfwsDAAGvWrEF6ejo
sLCywbNkyjB8/XhWR0RERKQUJolEzZRIJMMkT2xcm8sshBfZ1iZ6/shyBfZ1iZ6
ldb38VMH0G+zjA31nvBkRERERFRc8UkkaiZMzfWw7+muaBtmyfn/OmI1bFsal8sn+rCBJGIiI
iIlMLlpkQtgEgkgob6k898dLU1eSYiERERREdUL7yQSERERERGRgEkiERERERERCZgkEhERERE
RkYBJIhERERREQmYJBIRERERIZGASSIRERERREJmCQSERERERGRgEkiERERERERCZgkEhER
ERERkYBJIhERERREQmYJBIRERERIZGASSIRERERREJmCQSERERERGRgEkiERERERERCZgkE
hERERERkYBJIhERERREQmYJBIRERERIZGASSIRERERREJmCQSERERERGRgEkiERERkYrJZD
KsX78enp6e6N27N2bMmIF79+7Vqd/MmTOxYcOGKnUnT56Ej48YxQieiFoh
JIsGsg67wulOHNiqMhIiIqHUKCwvD/v37sWLFChw4cAAikQzZ89GSUlJjX2KiooQGBiI33//
vUrd+fPnERgYiEmTJuHo0aPw8PDA/PnzkZSU1JiXXQtBJNEwsQh3WHfxQj2XYzw9pBuqg6H6
onJPjVn/P2l11qykpAQRERHw9/eHl5cXXbG1tsW7dOmRkZCAyMrLaPrGxsRhn0kG
nftm0bvL294evrC2trayxZsgT29vbYtWtXtY19Os8Q5iEiRypPExlheMXjwYNjY2Cj8WbRoUWO
E3yKYtW+DDyf0wYcT+sCsPSfG5orJPjVn/P2l1liw+Ph75+fd3cXygwMDGBnZ4eLFy9W2/7s2bP
uXPw9vbG0aNHoa+vr1Ank8kQGxurMB4AuLm5ITo6unEvohYqBfEq/H0YIUbSM4p2E9kLknkEyY
Oaq3NEjQ64ujfKW7JjfxjBRdyxnmxrpKVzAQ0rpJb/SDKZwvLQ2oaxIaZDT3aXnyKdcN8Y5Eow7WMM2Fx
Oji9vbGlRpfSdy8bk6lBnl6x0ScVpaTSqySNHVtanaJSdNMq3fByxbLyFQUCWaBCTfJbdE6ap
QzT0p/ODhHl4bCoHZ8UZOVoBWRZyvh8crNEwqOm4ZP93J9nQt0FsvuyjWIsSM2eOQpq5ZxYsF
PBQtmQoJ9ZQNQsI3k3ksFTCPOExeXOCzfQP4pnEl0c7IzHOZN5fOJ2FSTNIXUEYb58pO4A0b

XqGmpoZhw4Zh+/btsLKygr29PaKiorB9+3YsW7bsuWIViURQVxc91xhERPXFOYiIVI3zEFHro
dIkEWj45RUffvghDAwMsGbNGqSnp8PCwgLLli3D+PHjG/lKiIiIiIiImj+RXC6XqzoIIiIiIi
Iiahp48B8RERERERERJmCQSEREREREGRgEkiEREREREREECZgkEhEREREREkYBJIhEREREREQmYJBI
REREREZGASSIREREREREREJmCQSEREREREGRgEkiEREREREREECZgkEhEREREREkYBJIhEREREREQmY
JBIREREREZGASSIREREREREREJmCQSEREREREGRgEkiEREREREREECZgkEhEREREREkYBJIhEREREREE
QmYJBIREREREZGASSIREREREREREJmCSSQC6XqzoEImrFOAcRUVPAuYgI0FB1ANQ0nD59Gj/99B
NWr17doONOmTIFALBnz54GHbelsbGxqVKmoaEBfX19X19ODg4YOHChejVqxeSk5MxZMiQZ463cuV
KjBkzpjFCJWoUnINUa/DgwUhJSamx/syZMf3ZMzA1NRXavfbaa1izZk21bcePH4+rV69iwYIF8Pf3
b6yQiRoF5yLVqM/7m19++QQW7du3CX3/9heLiYpiamsLLywvvvvPMOOnTo0Nght3hMEgkAsHPnT
lWH0OqNHTsW48aNE74uKSlBYmIitmzZAj8/P5w8eRImJiY4cOCA0CYzMxLFizAvHnzMHDgQK
Hc0tLyRYZO9Nw4B6mel5cX3n333WrrjIyMhNdqamr45ZdfUFxcDC0tLYV2jVd2ycnJuHr1aqPGS
YOBephrLvb44cOYKlS5diwoQJmD59OnR0dHD79m189dVX+PXXX3Ho0CG0a9fuBV9Fy8IkkaiJ
MDU1RZ8+fRTK+vXrB0tLS8yaNQs//fQTJk+erNAmOTkZQMWk+b99ZiUWUWRkVKd5xNnZGTExM
Thz5gyGDh2qUHFfixAn07NkTcXFxjRQlHtYt2xsZPP/1UKHN3d4eLiwveeO
MNfPfdd5g1a1Zjh92i8ZlEwpQpU3HVzB9tZfNtYTw8TqHuPydVgEGRGRAwGqn0ZyNBIGBCQbHRh5PnT
lWH0OqNHTsW48aNE74uKSlBYmIitmzZAj8/P5w8eRImJiY4cOCA0CYzMxLFizAvHnzMHDgQK

wYdOrUCX5+fjhy5AjOnz+PtWvXYt26dTAxMYGmpuZzja8MPT09vP/++3j8+DHWrVv3wr4v0Yv
GOeiJpjQHPcuIESPw888/Iy0tDd7e3qoOh+i5cS56oqnORV27dsWcOXOwb98+zJkzB8ePH0dM
TAx++uknvPvuu4iKisLixYtVEltLwuWmBACYPHkybty4gdmzZ2PlypWYO3cusrOzsXv3bmzat
AlmZmZ44403IBKJsHXrVuTk5MDW1hZbtmzBpk2b8MEHH6C8vBy9evVCREREtRsdAICXlxeGDR
uGzZs3Y9iwYbC2tq5XvKtWrcJnn30mfFJnZWWFTz75BP/9738RExNT778HoGLHr6+//hpr1qx
BSEgIcnNzYW5ujg8//BAzZsx4rrHr46233KBAwdw8OBBTJgwocZnA4iaM85BTzS1Oag2w4cP
x+eff46BAwfCwMBA1eEQPTfORU805bnogw8+QM+ePXHw4EGsWLECeXl5MDAwgIuLC7777jvY2
tqqNL6WgOckEhEREREREkYDLTYmIiIiIiEjAJJGIiIiIiIgETBKiIiIiIgETBKJiIiIiIhIwCSRiI
wSiYiIiIiISMAkkYiIiIiIiARMEomIiIiIiEigoeoAmovychmysvJVHQYRATA21ld1CC8c5yC
ipoXzEBGpWmPOQ7yTSERERERERAImiURERERERCRgkkhEREREREQCJolERERERERQkYJJIRERE
REREAiaJRERERERREJGCSSERERERERAImiURERERERCTQUHUARNQw0h7l45tTiQCASa92h1n7N
iqOiKju+PtLRKrEOYhIEe8kErUQ+04n4q87WfjrThb2n76t6nCIlMLfXyJSJc5BRIqYJBK1EG
kPC4TXqQ/zVRgJkfL4+0tEqsQ5iEgRk0QiIiIiIiISMEkkIiIiIiIiIiAZNEIiIiIiEqg8SZT
JZFi/fj08PT3Ru3dvzJgxA/fu3qxA/fu3aux/Y0bNzBzBt2jQ4OTnB3d0dH330EaRSqUKbydPwsfHBw4O
Dhg1ahTOnj3b2JdBRERERETUIqg8SQwPD8fWrVvx6SefYsuPPzBwdWBAASr8/iHlFmyRGhz/vx5BAYH6WbjsvfyRwbjzl+e9fpd2W5HWy9ocGvgEUrz+oKSmvAAXgh+SpAr4r0+0FMUTmpzl9gnu9GD/vVwM++uMbsCCadrxkneY6vvHFgYAEAqlaKgoACmpq
ZKj1cXGhoqX51LVCORSPE1f1LVCORSPE1f1+JiIiIqD5UmiQXGYCAMRisUK5WNZoxmMWQL75VmjbZBAWWWcnJyau0aKgoACmpq
GYurUqTh27BiKiopqHK+4uCFkUE4uPi5YlVTE8HQsM1zjUHUmNTURAqv+ftKRERERPWh0iQxOzsbAGBsbKxQbmhoKNRVdefOHZSVlWF
iZWvAaC4uBg6OjqVlVAJ5eXwWVLAVITtQniMjISX5lcw3tPqMt6ygRySKUFzzUGUWOSyeQKr
7Oz81UYTeNiAkxERETUeFSaJMbKvQpZUFzPrTXmHcHJyE5oeHfhgGj4opNVXyRvf/Sz1sL9sdLZ27zQqDZEDAiYHF7Ap0n1aBIe
yMDLRr1w66urqQSCQK/SQSZUlqPVRViZ77jGIGotcrviav69EREVEV8qfWjJ1tYWWenp6iI6
OFsqkUkllu3rwJFxeXKu3LxXKu3PnTuHHhQsXSIi8vTyi7/f/8+srOzYW1tDHZFIBGdnZ0YxHZFIBGdnBH4m1d
7dt4F0JERERERNRCqDRJFIvF8PHxwfLly3HixAlt3bvX4O1tj549emLVp00QOFFc/ODgGjFtH/4dlKJAOGFtSq+E2mPsV5wj
FFoSHcgLe2DN5qj76J8WmlD3UEWPi9bcIMqPxeZIE3ilcuBjYfbhDAHPSxTVZ8GVLGAgCz4uAwDGCxIUUbUQIFSPmvBwyXmbqDXOrIuaI
/iaBtb6fCkrTIFyAhgAKaFAMKAjyfkQ7MLBzZC4vwQKuX2MDQMQCLIm1C5uNAzOO8HUw9vqNnn
29AX18fgYGBSExMRDPz8v8fkcQ0QaY/D91Uu2rr5d1b92hbCYhz8eaRO0y3cmMr0nrgTa9Y8/yb
KJiIiIiIiaBZVvfxgQEICx8di+fLLmDhxItTV1REeEg6xQUdTA4aW0IQDhDsgsZ5qr9c
bMpkMEydOxPz582FnZ4fw8HCoq6sIHTPDw4q/Q2Lip3JOCMi2zGhUlNfR/bvFxYW9gYGbXyxZWx2qM6XrPiLtaZr5vrFTqqIqTgIb
IiKiZkGlzyQCgLq6OGpbO4gL5LqCDAxEY6FlcdznZDN5YbN4bY4SXNiUh65lXU2Z64pSeXqPLXl5kxqYZhQOObAb2e2XDMiyZP6
sPGjRtx8OBBSKVS9O3bF8HBwejQoQMSExMREhKCixcvIqBUZ2bF8GW3vwJvHXqtK7vVdmvOkpQO4DUNVOBeKwWTbnZfmuD+MhVj7r
FhwwY8ePAAVlZWCAwMxCuvvvPKiLomIio31R+5GINRSSXCwsKwf/9+w7ArAFjxibIAgcOkEIB8o3L0ZpMs2ZlNwowQ2Z
r7JQMVGyE5efnBOtLSxw5cgRhYWGIjY3FkiVLhDbnz59TT859HYGAgJkahKNj8+3LDwhwPz589HULs2Kc
i7wsIiIionphkkhErVpJSQkLg7+8PPy2vNRaYt26dHcjIyEBkZGDf9ikpKfD09Zs+eDWcDsr
Kzg7OyMcePGISoqSmizbds2eHt7w9fXFPwf3dXfT3MyD2xa7e9e3euF3l8pRENaRPEDEzNEZVLSNc7bqPBGzKCCm7b4bHpHV5pfYHAEd3XD2WRc
NNb1e8atHSExJb2bWOWsAj1WHM2H+KMoMj8NwBD9DiAVTqYApgP1Kc7AhV0Xf3FwiLfl0ZFBVBmuzcAYqSoWl/b7XvkmjBvPvQ2sLMChisM
DtAN9tNQfiLxSxOa6JYSkqmLPa11Dl9pRxRo2cvBPjYOFn5uqmaxaHaCrh87BoYrqzhNeBZeMbJ2ptkrtNr6cRqbBryYt8njtNbHOEz8y4
MSRKsI5tBwFnXm32C4sEbWtJ8bxqMPcOTVkHsNt5M3cNr3XfKVI8bFvm+aFmHR4Pw86A0Amxjw3OXFWmDuGr1V+RCc+Zsk0Mnt0NvpqdFMn
IiSS2wK8tQGF2bAKNrBrmVOxHvZ0NBxMtB2o+P3Z3dcvyPD2MQjObrDsuTUoyEwaCI2jd5GuQlnPdR1BNrwpKXqq9d2eQP8U5ZoD/DKxRKN7
xDMCYW3JgA2hZNk7hI0hOM1vXHJ/oz1ykR7oSWwF0p4nO+TqUO3QyEeZpz8xLDBjqm6DVhJ04ZJkF3G9
dkfMu9YUrEtGWJIVYb4Z0oeGa9pAEwjhxfSzQ3j3n0rhx5L0/hn2Dy2JjqZ/23fAVnr9p1Zgr1yd5U
NgY1TtpYpXh3TJo6FcmOjZl7Kx2nHVbP1jjGw/iF2LmdMBFSAEKrw09wHhNNDFkQ/MNQC2aJNPvmhWh
d7f/0/TNTvHFZt4yMB+D4/zeZ0e5Fd4TH3jtuhXGJ0ytWrf9JFNwAK77SmwXAUSvJjjHGdMDexuhYl
hmyXfG1CAPUjmZn7qYhHwzh0DJ2o09HmZqsE1qT/hn8qqFR8tGpyp3FnbmWsMlW6BDxH1gCqUBv+C+GM5
8sS7f8oPkkn1xrnqFd2+NXE9cuJqhaC8xCnCkBuoO3ewfdoAOIj/aiLW2ZJsoj2HDn8tWsmq1sOwFT
RWmcdEFCv9rGT7yBVpUkj7+ruEd6QbhHSmCkYrPKkMtsP5h2+CNnI3DI+oP8cJNeeoFaa0mAjcZpF1
iPcsn+xuVPy6j1bO2c6hFKkVMpcGe6XoerOUVhE8wqWTc9omhZXq9Uc0+1s4mhbn4jMBA1UbGxBpE1
Ruy/bo5zDuHUMl6ljd1nBMIk8FyomOuLmyVdakIT2rWwVPiLWUAG7KXXYXdMvvf+1v3eXoDhpVOX
YyGJ4m6m2bZbeyWmdaBNnGT2ahpCIPaB5h+7K5WTRXRXSTi4skU2ZlKbXkBQCJQb6vYd9LZtDu
4dBa1gyhe0yU0qIdDnbn7fj4HTqV0n8+YQxkTs7t7u3D8Fk2qlNBX2k4GXs70XUTvZwfI1YnT

llZLYOCx9bqnlq5JYdE5LJKj6CajsNbW9+XvCBxvIHDmOhOFRp3e/WX0GaaIVsxhCFCILOPsUq8tY

qFE4e/ZsY18GEbUQTBKJmqHbKTk4H5ehVB+nHh0aKRoiInpeYWFh2L9/P1asWIEDBw5AJBJh9
uzZVY7TASp2S/bz84OlpSWOHDmCsLAwxMbGYsmSJUKb8+fPIzAwEJMmTcLRo0fh4eGB+fPnIy
kp6UVeFhE1U0wSiZoRuVyOX2OT8e+vY5FfWPuupk/rYqaPrmYGjRgZERHVV0lJCSIiIuDv7w8
vLy/Y2tpi3bp1yMjIQGRkZJX2KSkp8PT0RHBwMKysrODs7Ixx48YhKipKaLNt2zZ4e3vD19cX
1tbWWLJkCezt7bFr164XeWlE1EwxSSRqJkpKyxHxQxz2/HwL5bK6H2ehq6WBGT49IRIptzyVi
IhejPj4eOTn58Pd3V0oMzAwgJ2dHS5evFilvZOTE9auXQsNjYqtJW7fvo0jR45gwIABACqWrs
bGxiqMBwBubm6IiYlpxCshopaCG9cQNQMPHxdi45HruJ+Rp1Deto0Y4wZZ41RMcrXHYXQx08c
Mn54wN9Z7UaESEZGS0tPTAQBmZmYK5SYmJkhLS6u177Bhw3D37l2Ym5sjLCwMQMVZrwUFBTA1
NVV6vGfR0GiZ9xee/hxVJGq510lUV0wSiZq4v+5kYcuxG8gvUlxe2s2iLd4d3Qvt9LTQ394UH
2z8Azn5Fc+u6IjV8cHbfdDVzIB3EImImrjCwkIAFee2Pk1LSws5OTm19g0NDUVRURFCQ0Mxde
pUHDt2TDjXtbrxiouL6x2nmpoIhoZt6t2/KVN7ajO4lnydRHXFJJGoiZLL5Thx/h4On/0b8v9
ZXTqkrwUmDO4GDfWKTzpFIpHwGqjYydS6U9sXGS4REdWTtrY2gIpnEytfA0BxcTF0dHRq7evg
4AAA2LBhA7y8vBAZGQkvLy9hvKfVZbzayGRySKUFz27YDMmeeoxDJpMjO5u7vVPT15gfZjBJJ
GqCCovLEP5DHGJvZSqUa2qoYdpwG7zcy6yGnkRE1NxULjOVSCSwtLQUyiUSCWxtbau0T0pKQn
JyspAMAhVLSdu2bYuMjAy0a9cOurq6kEgkCv0kEkmVJajKKiuTPVf/purpD2Pl8pZ7nUR1xQX
XRE1M2qN8rNgdUyVB7NBWG//n25cJIhE1WWmP8rHmwBWsOXAFaY94J6aubG1toaenh+joaKFM
KpXi5s2bcHFxqdL+3LlzWLhwIfLynjjnfv/+fWRnZ8Pa2hoikQjOzs64cOGCQr/o6Gj07du38
S6EiFoMJolETcilBAk+3RWDtEeKy3l6dTHCR9Nd0dlUX0WRERE9277TifjrThb+upOF/advqz
qcZkMsFsPX1xehoaE4ffo04uPj8f7778PU1BTe3t4oLy9HZmam8KzhG2+8AX19fQQGBiIxMRE
xMTEICAiAo6MjBg0aBADw8/PDDz/8gB07diApKQmrV69GXFwcpk2bpspLJaJmgkkiURMgk8nx
3W9J2HTkBopLyhXqXnu5M94b1xt6Opoqio6IqG7SHj75gCv1Ie8kKiMgIABjx47F8uXLMXHiR
KirqyM8PBxisRhpaWnw8PDAiRMnAACGhobYvXs3ZDIZJk6ciPnz58POzg7h4eFQV1cHAHh4eO
CLL77Avn378Oabb+L8+fPYsmULrK2tVXmZRNRMqPyZRJlMho0bN+LgwYOQSqXo27cvgoOD0bl
z52rbJyYmIiQkBFevXoWamhpcXV2xdOlSdOrUSWgzePBgpKSkKPQbN/TQbNWoUQkNDG/VaiOojt6AE
X/33L/x1N1uhXFusjtmv2cGph7GKIiMiohdFXV0dgYGBCAwMrFJnYWGBhIQEhbug6XX4bpg69att
Y45evRojB49uiHDJKJWot5JYlJSEv744w9IJBJMmTIFDx48ENbUKyMsLAz79+/HypUr0bFjR4
SEhGD27Nk4fvx4efnB1dXV+zduxfFxcX497//jVmzZuHIkSPQ0tJCXl4eUlNTsXX
rVtjb2wt9n94tjKipuJeei42Hr+ORtEih3Ky9LhaMcYBZe27BTUREREQvltJJYnl5OYKDg3Ho
0CHI5XKIRCKMGDECmzZtwoMHD7B3794675xVUlKCiIgIBAYGCjt0rVu3Dp6enoiMjMTIkSMV2
p86dQqFhYVYtWoVtLS0AAAhISHw8vJCbGws+vfvj1u3bkEul8PZ2RkGBgbKXh7RC/PH9TTs+j
EBZeWKO6i52BjDz6cndLRUfqOfiIiIiFohpZ9J3Lx5M77//nusWLECf/zxB9v/zxB+T/7Bm8ZMkSyGQ
yrFu3rs5jxcfHIPz7kKZgYEB7OzscPHwPixSrt+/fvj02bNgkJ4tMqD5tNSEiAsbExExE0Rq
ssrKZdjzUwLCf4hTSBBFImDcIGvM/MG92LCSIREREREqYzS70QPHTqEgIAAvPXMzXWygvf7LBhq2tL
QICApR67i89PR3Ak/OBKpmYmCAwEYuAPAzaa1KewsLC1hYWCiUbd26Ftzuw7CEJtgvHhmdvBDX8mBBjzPJ4MJVMoB5l5KIxSlx8Nxn4/TkJtTTd5WlL+fNm1iFEpLCvWxpQ9ZIYiqGPhc3M/4M3RJxdGmn7eDNMG74bLtE4mrxGPNdA6IvsdRb+eXz8GbAOgfP1pKGyR3h0PyOOSvT2/7Cl5ZomYsP7jGhF9YRuZ+gP72HJW5ycBxOAcW1m7dvzWdiW7mQrEcVexhnVeQ4Ft8OzORlH0Wp2mIbx2G+SLTZD6OtBC+YUlCS6UiVCu+ifMmLoQQN09zvyZlJ0IBUFqBMZGpL8egpzTDbYLoH9KgijrjDnq0bnIxs0hFfmbr5v8aiGr95fs8/Mqhj2eMYuNxeq8gm9yG1eeGdRNwvhJI+r56qC3UWM0gGpHqO/s2BZGoXYIrYSOD80JKQ3z1KhJS1WTA6x3W7oZtd9/0hBFmrZx6JXdE0xeclyQzBQIy+rMk95dKeRi9oRvn8+k53HOOnO3ezKi0k0Dfx/e9tzC54B3cBhUm/mD3SbOJDoo2Mc=

3ppkSPgrymFltSMnAut1irXWIhxkv/44MRvi4GioyIiIiIqOV0vqUxc+ZMxMbG4uTJkygtLW2
LmIiMTsHtSvxj+5lGCWLXLlZY+UIAE0QiIiIiMho630n85ptvcOPGDcyaNavJ7SKRCJcvX37o
wIiMxamMW9h2IBOK2jqt9sc8HTFvki+sLS3ucyQRERERUfujc5I4efLktoiDyOjUqdX46uc8f
H/qWqNtz4zqjUkjPSBuuMI9EREREZER0DlJXLhwYVvEQWRUZFVKbPzmIjJ/v6PVbiU1x9xJvv
Dv6/TIY3J1skaJrAYA0N2J6wgSERERkX50ThIBQKlUYs+ePUhNTYVMJoOy9vT0CAgIwdepUSKX
S1o6RqF25ckOGj/deQFmFQqvdzbkTFoYNRDd7a4PE9XxwP+wScgAAzwX3NUgMRERERGT8dE4S
ZTIZZs6cicz8MTHTv3h3Ozs7Iz89HSkoKPv/8c+zatQu2trZtESuRwR09dx2fH8qGk57gYth/
bti1v/0h1RiZqDIAFfHTljyrL/Bvj8RERERmQadk8T69Qd37tyAIAATfuZM2ewaNEifPERdETKyT
i5cmWrBklkaLUqNT4/lIVf0m9qtYtFIkwf1xchCe9fpDdiIiIjIBOi+BceTIEbz22mtCSI
ABAQEYNGiRfjhhx9aLTii9qBUVoMPPv+1UYJoZ22BqOf98VRgDyaIRERERGQydL6TWFVEVhR49
ejS5rUePHPHrhz5587DxkTUBoqMiIiIiKht6Jwk9unTB
z/99BNGjhzZaNuRI0fLuNuRI0fQq1evVgmGiIiIiIiorEsheoIzyMGOdA12rzmtaCSI
A/cT65QIflABoR7y0UTEbWy
m8Vyzesb99SYoJbTOUm8du0aRo0a1eS2UaNGIT8//GDImprJy4VYvWOX3H7To1W++B+Tvj7z
AC4OXFqAhFRc67ckN33DuK98m9W4MpNWRtHRERErUHnJLFr1644f/58k3v4f58k9suX74Me3v7hw6KqK
2o6tTYdSgbm/dfhlL1Z4EFEYCpY/pgQdhAWFvq9aguEVGHIggCjqbf0OmYs9nFbRQNERG1Jp0
/DU+ZMgUbNmxAp06d8OSTTT8LPgw1q9fj5kzFnEQPrbxSgQ37LiK7oFyFyrvZOl
OeZP9sOAPo4GioyIyDjUqtTI+r0M53KLkZ5bjBKZQqfj5TW1bRQNERG1Jp2TxHnz5iE7Oxvvvf
Mc/8N5772naBUHAhAkTEBkZ2aoaoBErWG3OvlSQNp7AXcqlVrtVbAXWtWVrtPbva4NWwegejaxcpAkRERtW+yKi
XO55UgPbcYF38rhUB9k4/xB92FtuadkGdhgmVqVj
O4uLikJ6eDrFYjjMDAQxfvhzdu3fDv35d7Dhcv35dhJkyYhPj6+Tc+FiyFzkiubk51qxZ
g4iICPz666+4c+MxOjt0whS5Ix2BrpcBLFjER6EwBP5+9jl2Hc1Cn1i6YEOTngpkTvCG1MDNQd
ERE7Y8gCLheXIX03GKcyy3GlesytFfLwTLnhZo0ZFV3XnpqvR0XNQ6eEJfz6DyAQnzoh7Vhv7oVeRiAYDgbQ7I/
JSIJFor9NbVlaGWbNmITAwEDt37oRCocHH36Il19+GXv37oVUKkVlZSVu3LiBTZs2wc/PT3O
spaXloz41IjJCej981a9fP/Tr1w8AcPv2bQRVFaGurg5GZVDk77gp+o6m58mZvzATe2Dsro337Iwn8uFGq1m4lF
eC64H8YNceMCz0REuPu8dtbvdzTTSIvLa5o9xkwsQj/3zigqq0ZpRfPTTnu72qKPq11rhGtyl
EolkpOTERUVhbFjxwIAEhMTMXr0aBw6dAgTJ07U6/w7/w4cOOrq7GBx98AKlUCgCIi4vD2LFjkZ
aWhqCgIGRnZ0MQBAwZGrdvy5E5FudE4E5Sq6qqN5778HX1xcPHDgKVL6Kurg4eHh5
ITk6Gq6trW8RK1GLFd6d6pe504SREZvAZK8eXQnWTGBFRO1EhbzCNNL8ScRRtzs7/Pr64vTp0
0hwDPR3h39cJA3o7wNrmCWmmmN2aH9emLuPzPzMxMVFVYcSIEZo2zs7/Pr64vTp0
42SxKCgIHz88ceaBLGh8vK7z91nZWXB2dmZCSIR6UXnJDE+Ph7jx4/Pqj+4PIi
Ii8M9//hPx8fFISEhocX9tMQf/4MGDWLduHa5dL+aXY+M1FVNV
of2Dp694Zr04ZgC42jf9TJSIydYIg4EaJXDONNO96OVqybKGLgzX8+zphUF9H9H9HXvDDOxdmF0
N2cbrAgfgq3fZTS5HEZvV1vMDu0PN2eb1joVk1NYeHfGy70X2bt27YqbN2822t/d3R3R3u7u5ab
Zs2bYJUKkVgYCAAIDs7G9bW1oiMjTs2fh4OCAsLAwzJw5E2KxzsXtNczN9T+2PWt4/UIkMt
3z7Aj4XrYOnZPEI0eOYPny5Xj66aeRkZGB69evevY+nSpQgODDoZKpcKqVat06q+CfPHkSUVF
RWL58OYKCgvDVV19hwYIF2LdvHw9PXU9XTIigiDgwMmr2PPLlUYffIKHuOPZ4L4wN+NAQUQd
h6pOjexrf04jvXdt2KaIRSJ49eiMQX2C4d4d4N/XCd0crJrs3Js9xsZBn9/MQBvrP8PPyqvuFgizkpjhj
ef80cfVjncQm1FdXQ0AjT73SKVSz/nss8wa9curFixAo6ZXiWAqGhoVi4cC
HOnDmD+Ph4lJeXY/HixXrFKu+emusIi8Uirdemp4dAd/L1qFzknjnzh306dMHAPDzzz/
D3Nxcc1exc+fOGb7Lebgb968GSEhIQgPDwcALFu2DGfPnsX27dvx9/xDJUvGe4WcIPbwhbHfPlvbsnJs
f5eBX7Nva7VbmGI1P1YVJFNSWV2LC3kl3Iv5JahWND1p3nr+dq+1lNLQVn+GzyPr46oL6OG
NjHEZ30q20SP1Hn11NNpn8ZiVfpirGtnS3CMgqFAlZW96++++LQgCPvrroI2zYsA
/HSyAX9o3dRgEpZ6HuKfASGuAhgX4lAcC8aQ1HyArsGoa7dWiLx8iD4dZCQFuXTLhDnjQ3VXK3ZtAIggA6DAHT
VBoyGHkZHei/bMgHWOUl0c3NDVlYWr4mSrMVtHzwemBGYLxdCl1ye5w/2Ecn3jklphfSUBGaAYVgX
XF0FSbPAY8HUp+oPmI4sr9ryD4MSavRp6crH9hjLU1O8wcZdIOFjMpxt7NEjfe8W7v6CZN9mL7gXxrdz0qb/Z2
dUpMm/F3sX3JTjEK5iw86Cu/aSzQdcrUbfWvuFQJO1UdGrvOaFC9q6dmiApTjYeY54k7PyDtJ2xUOOt5cHSi2F2O9d
bSS3RWJ3lgQVu5bJW2aZh1zLYhLJiD42Fb7ZFp43lKwdNRcZWpHhlnbc06MFjXyT3iRRdkKvUUjQSJUQRpSKj6R7WG
cXe0oJ0MrmKbJJmyqS7/k8GLePXqZMqn9lYhdLkGZA4e7s0/lL1TrOfQj1Kyw7TqlDsJmzcFWJ9M48/89ikpGQuVT
FwejJcu6Ru9/XNKEVK8QkxC/8Y26EEDUKTUSMRyB8NOhDDVbEYQmVr7NRDqrLyP0rrq97oY9c6Zml0fbH5/bjUSYD
htw12IQaNvO7mDMbsMcMkDmxqfRmK3VuQm8MlaCq7lWhHvm9mX3ULAWyQcTrBIYBfeSKX6yH0e4z/CxKKgHWoJJX6
qTnpYmt7lVBhCDOnWrgtx9gdl3qlYRZwbnmSeuIazfJvcWjr+2BrZkY5pc3hoZELZt6clU5n+mvvsFQxP8vrx/1iT
HPK7Bw3vM+5+W3dk0XeS8jhxehfbxf6t8RenwVSGcjFlSlnxg2jwKE9YSe5UjYRN7mOcH/L9+4rb6hQfxDwM4EHsIz
bJeKH0gHoTqVu2+pJ62ve3c5+aBb8UMM1EDNHeUBdXkybw/F9k09MMhzBeUzKRzLF7i5TvgkKCdpfWZsCV1kSUQRP1H
bJVyGYsDJzxn/Yr0tvoa2CBrSyckxT4oR63/DhkJKbImzDsVIWn9tcULp0pTnqXNiCsCg5pOxVfXt7ZGfi6IJfzFdt
nkSZKa8bOVvN8VOD3BhUXAiSZpKiSgzrsvk1ny8vFOthZLxpfFbuL1qS4xmKjaG8WzkHu3Nf9Ny/oPdQbM52T1jbdU
i+A9yOvd1H/TgO1N8JehJhdEhgK1cVsZ7kexq1cFXfH/vdgcvCoW5tzVQCG7+ys2qAHIg4+5ibTsBiHNZgxKjx/PMJ
GIBkl8erL1pGmMHvQ4e5CqFr0/0Vmx4J7k0ZoE4ecDk4f2RlkqzMc4D0e4/tYEkyQqXyEDmNMXF3lT10Ih0+PnBlay6/
dOsz03hqaoGRKbWxD3BPPchOOyWkHuZWSBZoEVJ4GIRe8JpUpEKbOeFE2F+akwZhr9Og7PcfGZ/1vXvYVd4TL1S2aw
ZrjpV3hpoqaxhMRIFoJHXGH3JBGGS6DgfJpGL7f1EhGbEJSHvPkqlKRHvaI9fSLmXYCNg6gLbvbYPO95xkr5XXhq1A
/rnSBbHSh7xAUQnGjJwMXy8b5rYa1vGKw6o1s3Z0OgEnBWK6yFqEFrtvRP72Qtrx1eAfPV67ZoqgjmQAU+Rs3Nm+ijh
i+aVlmlGq1G50eaHcI9RTre90BAz81fD8PPMM8CIgAWYlVDN3c0lUOWHgMbGm/ieHZkHGyoBV

fLnWtuHDh+PQoUO6nCYZkZslVVi/5wJulmj/x+XU2RILpg5ELxdbA0VGxoJVBcmYCYKAwlL53
buFOcXIaeE00m72Vpq7hX3dO3OmhQHVTzMtKipCz549Ne1FRUX3rRxfW1uLFStWICUlBUuXLs
WcOXO0tltYWMDCQjvZ9/LyglwuR3l5Oezt7fWKVaVS63Vce9fw34wgmO55dgR8L1uHzknijBk
z8MEHH2Dnzp3Iz8/HmjVrAACRkZE4fPgwVq5c2eK+WnsOvkwmg1wuh4uLS4v605Wpzmk25rnb
ZzKL8Mm3lxoVXBjQxwERUwbA1lpynyOJ7mJVQTJGqjo1cgrKNc8XFpVVN3uMSAT0c++ieb7Q1
ZFTsNoLHx8f2NjYIDU1VZMkymQyXL58WTMz6l5Lly7FoUOHkJCQ0GicUqvePLJJzFt2jRERE
Ro2i9cuAAnJye9E0Qi6jh0ThJfeOEFODg44NSpU4iMjERoaOjdjszN8fbbb+PZZ59tcV+tPQe
/Pulsqj9dpsE2xZTnNBvj3O06tYCdBzPw1Y85jbZNC+6Hv03oDzMxn4Gh5rGqIBmLqpoG00iv
lD6wmmg9K6kZBvZxxKC+ThjYxxE2VlzMvj2SSCQIDw9HfHw8HBwc4Obmhri4OLi4uCAkJAR1d
XUoLS2Fra0tLC0tsWfPHk11+WHDuH27T8ftajfZ/z48diyZQs8PDzg5+eHEydOYMuWLYiOjj
bgmRKRsdBrncSJEyc2+uCUmJiocz+tPQe//kObUqnU2r+5/lqC8/Dbjwq5Ehv2XsTF/FKtdku
JGeZN9kOAT1fIyk3zvaK7WvNCBqsKtg/GPKOhLd0sqcK5nGKczb6N7GvlULdgHmlXeysM7ueE
wf2c4dWzyyOdRsr3UX+LFi2CSqXCypUrUVNTg8DAQGduduhUSiQQFBQUIDg5GTEwMwsLCkJKSA
gCIjY1FbGysVj/1+yxZsgR2dnZISEhAYWEh3N3dER0djenTpxvi9IjIyOiVJLaW1p6DL5lLaW1p6D36VLF1
hbW6OoqEjrmKKiokZTUPVhqnOajWnfcwElMu0Kfa6O1lgYNQ1lgYNhCujp3adfzU/rCqYPt
gjDMa2kJdnRoZv5Xi1OVbOHXpJq7fbv6inVgEePdywDA/Fwzz7YYe3WwNVk2U76P+zMzMEBUV
haioqEbb3N3dkZWVpfk6OTm52f7Mzc0RERGhNd2UiKilDJoktvYcfJFIhCFDhuDUqVOYNm2ap
j01NRVDhw5tuxOhR+I/F27is++zUHtPEjjU2xmzQ/vDSmrQX2cyUqwq2D4Y24yG1iSvUeF8Xj
HO5dxdpuLeNV6bYikxw0BPRwzu54RBfZ20nr++c8dwvycd6X1kAkxEpsygn6rbYg7+rFmzMG/
ePPj6+mLMmDH4+uvCYhEwP+O9cSE4T25BhfpjVUF
2wdjmtHQGorK5DiXW4L03GJkX7uDOnXz00idOltqpqpF63zONtL38vDra+0hEZKoMfuultefgj
xo1CqtXr0ZSUhIeSExPRt29fbNy4ENZEZ6enoY4PXpIZRUKJO27jG/2ZjuSpeoPKO27gLzrMq12GysLzH/GD34eDgaKjE
wFqwrSo6BWC8i9/mc10nuX7GmKCEAfN7s/qpE6wc2pEy+IERHRI6F3kqhWq5GdnY2ioiFTI
EKpUKXbp00bmf1p6DDwBTpkzBlClTlTdI6F2pfsa3eQtO8iZFXahYh6udhiwdQBcOr8cMWIiABW
FaS2U61Q4WJ+Kc7lFOPClRJUVtc2e4xUYoYNg4Y1NcJj3k6wq4Tl/EhIqJHT68k8ZtvkFCQ
gKKioogFouxe/durFu3Du3Dh YWFkhISGhUAIJIF4Ig4PCvBfj3j7mNpmCNGuiKF8Z7wcLczEDRkS
liVUFqLbfvVN9d1D63GFm/t2waqaOdtME0UntYsCIoEREZmM5J4oEDB7Bs2TJMnjwZTzzxBF5
//XUAwFNPPYV33nkHSUlJeO211lo7TuogFLV12H4wEycv39JqNxqcQxOLMCPEC4/7dd0K2p1rCpI
+lKrBVy5IdMkhteLmy/UIgLQu7udJjF0d+Y0UiIial90ThI3btyI5557Dm+/DbWFSpqcBfhY
SgpKcG///1vJomkl6SUfJAyPaXbrJm4w2BAYLWQQ96Ip8S3Z4HLCZCvK7GoUlM/o/WE3MeOHf6A7aj7DDEjmtH6bc1hF/8XFPL7kIjXNo4egtw3vE0uWaEBkLpcbgwBj5tDsazn6NcjnNgBD8ZsbbYrmmr9rZPa5LHaSaLHiwIE7ToJNHPcVNK1kzG0nHN/OLnfO3/t+Y5MA8tgyS6qHntP0UJQBUvzeH9XsmtTOPYJU1bGXWXM+GiERp1B70R/sRFQ15WjEeU7cHmhaG6Dz6mmgEYSvmAKS/lCmrn4RfMEVAiOa6uZP6nljvD8Qh2wQAO0M8wQD01zdxiaekMcF3uHlzUZ4TVvf59+VQ89fghrzOfjDiXzK5yWaSfFFF/j4vpxiNAP20Pzk9AOW8dvgPKwHhGVzS1xGBtGmCJOp0cMmVPKc6iz2H7ZoD+UoA9oYsDMJGt4eQOz6cQcHaFaPlIykhzfwdQZZ2kEvXxa5BFRLY1EF/EBixbfWw9JLXjkIBZOugd+eOJS0Mt3YwOQSGrdwkyGhfUrGEZ4sx3UGUVdXDi6OW57lTYbHvNtTCbXo7wXd8JUl12UCUTK7GQwybhOeZZdwYvH8ZkBJ1ntx0JKnytPQiimldwY6LTRBo1F8WFxf5Jzy8ZX8eXuXuyyaWWPAYunyzJlZ0yU0cEi6yYhxyT1Hsbp7OQsaE0XoSrJdNsMzPPMxSkGWXn5e07cW8pfifwY1XdXmTs+0hL0Fp8bRBWzdT3uAQjPwJczaKWZFbfDMfBnjdpcPjHrnXb2ZqdxYBjLVeWwUWkPOafbL4rwO+zX6KMwAxhLm6Xtb8lYbS82f2mJcSJzLDRfNPgbfsUg08sEU7fTrPtdJHqB+Z7Mx7bAqIobFD0+nDo0u4R5aXVCZCBLs5ViPd1TL6Lu+SHKPqdXPbkLOkVmSHQv2HwgbM/0Ig4q3zfyYfsL8Gt9Ge9W9ve/zmFwJlo69mS0SzppDcYGnJ3Xm8xrwQVOB5Fw9Xz8iLSK0LfVeXwWlo6hyHHXX/mgZMi0Q8GZ1jAS5bvX2Gv54zlBxSYd/x6Lj2n6GfZ2UbRUHvY7+HmgN4hKnJXUrf+zpLQH0LRZgPgWYpyDK6fq4DzBH+P6X0sxbDfH7p3ZbN+bPfxLKyUtrKuaJ6dBx5kPHa+81dnexVDwz8gNGlZkTs5F3yS1Snn5WzdJoW4WTB5oqVyJDaqkWPV6j3jUNpbw+i3fTI0WOsQN0NCTswbWCPQHn0XDbfrTFoT01pRA/nH+1P3oMKNQIa7wDYZLZMBeQS7k/dQ8iFPdfW7jtyWZ7SlFdG+q+TdnytRRUHUG2dbhGtxvQ/7ou9tcDd2Ibj3xS5JXpJ1ccaSRaOL6YbPTzavLMg92z5btR/+VkrsyS+6BnXd0Yx+i+88gWYA0Il9WmpDdgmbZncycbC9R0X8gv/vL+DFUS5gAkLfMeBw7H6hMbMjG4+E7b+2zJNuW5cu5LGe3wGOt0mbYbqu8WSdfjmOh6V0aW3HRb3uVpAZ6XYmgDEI1k5H3SzuuPfOgMN9+VUWf9eD2gI7Q12N9eB2p5f52gvPZemXTd1+GPZ0TWM/7f/KwxRC4gBf+Ez6/w4v6ml3ZVS0qaqjHaRSv+w3YGHdIK5TcTKkHAXG1S1NRB7jh/XyMo3Mb/KOzE9FfL6m8ulbNcxOm17xGNWnsf4bGc81BajTmbdJNkBFP/+sAkrZ+0VDb7Q8YSnYsvpKmnEo2ng3Bpx4YFgAXyYH/vAgOHP3jTFLHRP2dbA57sdhUw6sBpkP3QnHoA6TGvZXfYpP5JJEfCUCj7hLsRvsP6kTRBhJtWE9TnvVVIgsgcXVYGcxT7F6iGjq6Fn7XxJ9+p3L+DTgS/0nmkLf2u1/mwhvRPrQmTAFRSsgBeuBg7Kz+z2kZMtz3KDR/4h1h2x4/zpFKlbNHHg+hbEkZ8R88UeWhEAvrXcD/JJNbR7qNAo1T4Ckt5Pz/CRxTPHTUXxwuSbX4j0gv2O/DqdMjg+C8qQG3xb96GkFk5yO6XyxfpD2v6Vp9kFx3AZP1YxI0+DTL0w/kTPYszSCAhEbCYL3AiCfzkQ7LwxmpqQsCuP0I8xmmFkb6HBH8gM92xPzd6+JqaYGXwLqPQUDRsWNLwCF+0B6o3qy1zk7PD7pRfVMNNd6r8ciGqTnGoI4Sra2YZ55Z2kdf6fSr8Z7aKPAnY8xk4uJgHbYmw6Q+DsmG6oSg1C4w2g3tA+EFYSB2BrdD6bfjkhzx1yQ05e/6CGi1wd0gCE/1i5FmdN49qObh0ztwi+XQn/pqNqFz0xhL18VGQoQyqNkMDAmDxbrd8S8sH/xLVlhNdg01oQ37h8T/mFWGQxoEX8b5Re7zIxY9qkm/e0m2+lCTZ9y8YNtF3H1xSbmmP4c8aKs2SRe5bQgxAefNCnInWjx5d+3mRP9SkeFsFNGcFOLSwkyvPADCDp5mWRE0mdMgzCpT85KVFI7Zt1W4wAZePg2Y3sZFD/Vc1pZ0Q0mXBWp2gEoW9pjpcPxMdbtSQP5NJc4WYN2Yv8nI/fOOlsBHr/BQSWNbbXR+YDnRZbBUWWTNNWrPcDbC/b0F6Gs49pHWXkqbmETpfTrpUm3OK0BfuZWIcLJgPyzJy9+GRbNVz3sSQlrE7sNuA/Y6c7zwcp0v35b9/Uc8SgPAyTu8vaEbvLpgz7EXX8GS6u9HUhXD9pDm0T1j8nncI5+g8YG19nz/lmBmTcAnD0Q8X5PLNDkf8ewNfjd5clE2T+Qz67Rm0RH5yrZDmvxzhkz3SXhQY5W+7u+U5nHIfgGflLgSUyewVW3n8FL9jFdzFOaQDC9xIH0bPKl5UGY0WtVljvx9sRvN/AYgBPBf2mTrsLvDnsTEbX/vQQ+cwQdS+nC1kWUyGMBf1x5ZMmDNb7QG3YwMdDmlDdD7E2+WF5n0zvDNojeuU9dj4T7GhfvfVkbfyLMhmhe2EmVRCP7lMdOJvvD0i3oUb5Pw6uwQ/bPfu6oXOBhPkYrvmvGh4vD7a8T8RXa8kFYS/XvXuGnzpeHdvdqBWLSpUaaKFB+pb8s7sWr8n8aNPRvoJfU6w8gqkXdmyIyVn8l4mS0Fd1nmAmA2XtB7TX7R1z82SOaVWwsU5iIpDcqWyWcLMOPdZUa1nRHw+HQ5VJTT2pY6GGM4P5e7jz9oM/ENNh2Z9HSBfpiz1SOCiEqT2RdCvJVV1l2AszhO6fzAvOgxXEpvDuJzCUnpJXuDHz4eXmX8QlhdW2k+J9MRvzKRJ0ALXPCLbR3MmS8Xzy+gBeaV8xxFeMKBE2jP/0z5ppn1y5VKcVPgBqTTyoCC1xUqgyZ13llgbF0W+0qL4RDjDbs8Xk8pJ2M0oHBbb19l29yHsyFrjoqTc9sKnpjxQ8j3Re0bzLfRhDVEJndqj9Ws7MXSIEEJwhqNv3DHFN0SOpCy44F9aCR6cqgHeVzW1vWW7UDJvRMPMupu6Scb4QPubVqj2dX4ZJFAuz/PdgA

ia1N4qYqrVgvbUyz/uvtUo/pxuWa28J8G7J9Grb2/JRTRjIgJgKTWDpcQclhIzWEnv/l1aXoP
Ce5ZUexBrVtFvEZ2TRF9fX2zZsgVBQUGQSCSadqVSieTkZPTv3x8AcOnSJbi6urZepGQUMq6W
YeM3F1FxzyLUvV3tsGDqAE7tIiKNllSjW5V8Ci2ZmGRuJoJPT3sM6uuEQX0d4dTZqnWDpRZxd
bJGiawGANDdqZOBoyF6sNaqiHk3sWuQ1DWTvDV8tu7eZFBZa3qJnbRBQndvgmclMf8j8bu7zU
r6R5vEDJZS7X2lFmZN3tnNu16O93f82uKYBnuxWGJL6Jwkvvnmm5g1axbGjRuHxx9/HI6Ojig
pKcHRo0dRWVmJLVu24MyZM1izZg0iIiLaImZqhwRBwPenruGrn/OgFrQ/0o31744ZT3rBwlxs
oOiIqL0RBAFbv8tothrdgxJEW2sLzTRSXw8HWEn1XtWJWsnzwf2wS8gBADwX3NfA0RDdX0vHI
LlChdh/nYWfhz0UtQ0KrjS4w3fvus+mQCox+zOJuyfJs5Jq//2gdqnErM1ncvTpbgcPF9sWzT
Tp7WqLPq52bRqPqdD5f9TBgwdjz5492LhxI44dO4bS0lK4uLhg9OjReOWVV9CzZ0+cOHECixY
twpw5c9oiZmpnapQqbDuQidOZRVrt5mYihD/ljTGDuhsoMiJqr67ckLV46mhDbs6dNNVI+7ja
QSzmNNL2xNWxE5Y862/oMIiapcsYVCGvxcnLRc3vaGBSCzPtO3D3JG/1UzWtGiZ9De/o/dH+K
BK71iQSiTBnYv8H3hUGAGupOWaH9jfq50wfJb0uu/bp0wexsbH33R4UFISgoCC9gyLjcatUjv
V7LuB6cZVWu72tFAvDBqI3r9YQURPO5hTrtL+vhz1emuADpy6cRkpED0/XMaitSMzFWolaUwl
do2ROeu8dvruvO/JFMzdnG6wIH4Kt32U0mfz3dr37jPujfL7U2OmVJFZUVODkyZOQy+UQhMaT
gaZMmfKwcZEROJdTjM0pl1Ct0J5m4dOzC155ZgDsOknucyQRdXS6Vpfr2sWKCSIRtZqHqXBpY
S7W3KXTSuakf/7d5FTNJp7DMxPzUZzW4uZsg7+/GIA31v9HU4jISmKGN57zRx9X465Uawg6J4
lHjx7Fa6+9hurqpqsIiUQiJokmTq0W8M3xfOz/72+Ntk0Y3hN/HduHgx4RPZCu1eVYjY6IWpO
uY8oTg7tj6hhPWErMYG7GzzjtlUgk0np/rC0tuCainnROEtesWYM+ffpgxYoV6NatG8RMBjqU
qppafPLtZVy4UqLVLrUww+yJVLrUww+yJ/RHo09VAkRGRMRnczwkHTl5t+f6sRkdErUjXMegvA11hY8WLV
dRx6JwkXrlyBUlJSQgICGiLeKgd+/1WBT7eewG379RotzeXezt8LCsIGc501ELcZqdERkSByDiB
5M59uA3bt3R2VlZVvEQu3YiUuFWL3j10YJon9fJ/z9xUAmiESkk/pqdNbNLFvBanRE1BY4BhE
9mM5J4vz58/Hxxx+joKCgVQJQq9VYu3tRo8ejUGDBDmH27Nm4erXrGm0Nm4erX52/9qtRpz5szBunXrGm0b
N24cvL29tf68+eabrRJvR6OqU2PX4xWxs3n8ZStWfC7yKAEwd3RsL/zoQ1pZcm4yIdFdfjc7Dx
bbJ7b1dbZtdxJqISF8cg4juT+dP9/v378etW7cQEhICBwcHWFpaam0XiUi/tLSkrCF1
98gZiYGHTr1g1xcXGYO3cuUlJSIJE0XR2zpqYG0dHROH78z9Pz9/bW2VVZW4saNG9i0aRP8/Pw
07ffGSc0rr1Rgw76LyC4o12rvZGmOuZP88Jino4EiIyJTwWp0RGRIHIOImqZzkuji4gIXF5dW
+eZKpRLJycmIiorC2LFjAQCJiYKYPXo0Dh06hIkTJzY6Ji0taitrYWdXeP54dnZ2RAEA
UOGDGlyO2kTBAGquj/vEMprapF3vRxqQcCGfRdxp1KptX+PrjZYEDYQXVmKnohaCavREZEhcQ
wiaknJDEmJqbVvnlmZiaqqqowYsQITZudnR18fX1x+vTpJpPEY8eOISQkBPPmzcPkyZMMbbc/
KyoKzszMTxBa4frsSW7/L0Fw5A4BqZR3e3/Frk/uP8OuGFyf4QGph9qhCJCIiIiKiR0zvh8mK
i4tRW1sLQRAA3H1GsLq6GmfOnMHzzz/foj4KBwsBAK6urlrtXbt2xc2bdN5s8ZvHixQ/sMzs7G
9bW1oiMjMTZs2fh4OCAsLAwzJw586GX6upVyLm8ztTIa1TN7msmFuH5kH4H4ICejBaR
dERERtQK1WY/369di9ezdkMhmGDh2KVatWoVevx9kOfn5OTg7i4OKSnp0MsMsFiMwMBDLly9H+7
dNfscPHgQ69atwVr1Vr1+Dh4YGoqGUx4YGoqCiMGTPmUZ0SERkxnZPZEzMxMvPHGG8j8y29s
DQCNnj2USqUoy9v6pBm5eTkoKyGhoVi1Ph4Ed9vdnD6tkbpL0/lPVJGewtb8PDrOBWmy7hHtfQ8gC
EHAu9vPtDhBfO+Vv2CAJ9coIyIiaiu61GgoKyvDrFmzEBgYiGDJYIRVsftrhOIzXDzD2Ehhk3Rh4vdKaze4KG2
IqleLkyZOIiorC8uXLERQUhK+++goLFizAvn374ODJBsBDSXOl10hArBb3oWgRVLSvcdXUr8PL4b6lR
CRCLBE088gV9++QW//PILPvvssxb3VVmRqlUahWWUSgUsLLS75m3bdu2QaFwMbvlrGb29v
VFVVYcOGDYiMjMTWvtpaaCJbA7tuLjbp2j0Xq5pXi6Qz+Y+4SuZJw8eu3GsvpDq1HS9wvYsZKXcm
42Gw4cPo7q6Gh988hGFjJyHaCkicAl4uHmWKjYgmHsoTw+KmxWxj3S0tFTVFzUDTgnczpPGbLiD
e77777aE+QiIyOzklien6l9mjnTpsHa2hrffPNMzyYgRkzZmDRokXYWsWMHAqIrwRX/TT
ToqIi9OzZU9NeVFQEHx8fLUMDAFhYWMDCwkrzvLC3K5K5bC3t9erXwBQNVgCwpidyZSZS
af/TGUXo1a3p8tBERET0cHSt0RAUFISBBx7QtD/xxBMQi8W4evUq+vfvr9X/9OnTcerUKUydqv
QkiMiK6J4lKpRK9e/cGPTp0wdZWVmFhYVi1ayM2crZxbl1SKJSMPly5ATRLCzqbtkM2sbAFP6z1LuQRY
50oVar8eSTT2LatGmIiIjCAwCAFOTk4PlSCaEnlNbZvuT0RERC2na40Gd3d3d3uLu7a7Vt2rQQ
JUqkUgYGBkMlkMvljarRP6jmQ0uZUn2GhhqWXBCJTPc8OwK+l61D5ySxe/xuRrSFkdO5vKRAH
Vy9UVlaioKAA7u7ukEgFIEB4ejvj4eDg4OMOzQ1xcXFwcBSEgI6urqUFpaC1tb2
xatcygWizF+/Hhs2bIFHh4e8PPzw4kTJ7BlyxER0freqomy9rSovmdHmJ/IiIiarmHrdHw2W
efYdeuXVixYgUcHR01SWdT/SkUCr3jNKX6DPcSi0Var031PDsCvpetQ+ck8amnnkJ8fDysrKw
wYcIE9OnTB4mJiZg3bx6Sk5PRo0cPnfpfbtGgRVCoVVq5ciZqaGgQBGmLr1q2QSCQoQKChAcHAw
YmJiEBYW1qL+lixZAjs7OyTdq7Ijo7G9OnTdT1kVkzW4nxMOnLza8v29WLSGiIior
ehbo0EQBHz0EUYsGED5s+fFuYDFr1Ge6lVgtar1mLwXh1pPeyLR
NgnZPEhQsX4urVq/j6886xYcAgsXLsSBAwdgZmaGNWvW6NNxNsfmKiEBUV1Wibu7u
71nTWe/3444+N2szNzRERExEaE13ZS09eluBw8XW/xWWNHsvr1dbdHHlWtOEhERtRV9ajTU1tZiyZ
IoQKi3MIo6KcjN4hUwqJZvt

xYoVSElJwdKlSzFnzhzNti5dusDa2hpFRdo1CIqKihpNQdWVqdRnuJcgaL821fPsCPhetg6dk
0SpVIq1a9eitvbuc2qjR4/G/v37cenSJfj5+WkNbtQ+iUQizJnYHzE70yBX3H8ZDGupOWaH9u
faiERERG1InxoNS5cuxaFDh5CQkNCosI1IJMKQIUNw6tQpTJs2TdOempqKoUOHtt2JEJHJ0Dl
JrNewgmjPnj2ZHBoZN2cbrAgfgq3fZTR5R7G3qy1mh/aHm7ONAaIjIiLqOHSt0bBnzx4cOHAA
S5cuxbBhw3D79m1NX/X7zJo1C/PmzYOvry/GjBmDr7/+GhkZGXj//fcNeKZEZCx0ThJramqwd
u1anDx5EhUVFVCrtW/hikQiHD58uNUCpLbj5myDv78YgDfDfW/wflVXefW7CSmOGN5/zRx9WOdx
CJiIgeEV1qNKSkpAC4u3Z1bGysVj/1+4waNQqrV69GUlISEhMT0bdvX2zcuBGenp6GOD0iMjI
6J4mrV6/Gv//9bwwZMgT9+vXTe3F6ah9EIhHMzf58D60tLeDZvbMBIyIiIup4dKnRkJyc3KI+
p0yZgilTpprRWiETUgeicJH7//feIjIzEggUL2iIeIiIiIiMiCdbwPW1tYiICCgLWIhIiI
iIiA9M5SRw9ejR+/vnnNgiFiIiIiIiIDK1F00337dunee3n54a1eiqKgIQ4cOhbW1daP9Of
+diIiIiIjIOLUoSVy+fHmjtu+++w7ffffdo3aRSMQkYiIiIHjlXJ2uUyGoAAN2dOhk4GuP
VoiTxyJEjbR0HERERERHRQ3k+uB92CTkAgOeC+xo4GuPVoiTRzc1N6+s7d+7LlzePzxxwEA
165dw08//YQpU6b6bAzs6u1YMkIiIiIiJqjqtjJyx51t/QYRg9nQvX5Obm4umnn8a7776rabt+/
Tri4uIQFhaGgoKCVg2QiIiIiIiHh2dk8TY2Fi4ubnhyy+/1LSNGDECR48ehZOTE+Li4lo1QC
IiIiIInp0dE4Sz507hwULFsDZ2Vmr3cHBAfPnz0dqamqrBUdERERERESPls5JokgkVZZP
blEolamtrHzooIiIiIiIiMgydk8Thw4cjKSkJpaWlWu2lpaXYuHEjhg8f3mrBERERERRER0aPV
ouqmDUVFReF///d/ERwcDH9/fzg4OKCsrAxnz56FVCrFmjVr2iJOIqI2o1arsX79euzevRsym
QxDhw7FqlWr0KtXrb3z8nJQVxcHNLT0yEWixEYGIjly5eje/fumn0OHjyIdevW4dq1a/Dw8E
BUVBTGjBnzqE6JiIiISG86MOns0aMHUlJS8Nxzz0Eul+PixYQyWR49tlnsW/fPvTu3bst4iQ
iajNJSUn44osv8N577+HLL7+ESCTC3C3LlzoVQqG+1bVlaGWbNmoVOnTti5cyc2b96Msryc
y1AoFACAkydPIqoqCjNmzmMC+ffswatQoLFiwAHl5eY/61IPylWjsWI
qJHTqlUIjk5GVFRURg7diwAIDExEaNHj8ahQ4cweJErf0PHz6M6upqfPDBB5BKpQCAuLg4jB
07FmlpaQgKCsLmzZZsREhKC8PBwAMCyZctw9uxZbN++++XWv5ICIiIqL2SOc7iUREpiQzMxNVVVU
YMWKEps3Ozg6g6+vr44ffp0o/2DgoLw8zZbN++XWv5ICIiIqL2SOc7iUREpiQzMxNVVVU
YMWKEps3Ozg6+vr44ffp0o/2DgoLw8ccfaxLEhsrLy6FWq5GWlqbVH3D3ee4zZ860/gkQERER
tTK97iQSEZmKwsJCAIC|rq6tWe9euXXHz5ks1G+7u6DmTZBKpQGMgDIRMJoNcNcLoeLi
0uL+tOFubnpXtcTibRfm/K5EhERtXdMEomoQ6uurgYrgYASCQSrXaV+Vry8vJmj
ixAo6Ojjpqks6n+6p9Z1IdYLIK9fSe9j2/vxGKR1mtVQ5L2zuBJoq5VBRseN3fuJuX7j+yM
yMlJrG6sKElFLWVpaArj7bGL9awBQKBSwsrK673GCIOCjjz7Chg0bMH/+fLz00ksAoJmGem
m+b6a45aLUAmk+t9fHunVgtar8vKml6Pl6i94IUMIjJlOieJK1aswLhx4zBy5EhYW1s/dAD1V
QVjYmLQrVs3xMXFYe7cuUhJSWl0Jb5eTU0NoqpOjcfz4cfj7+2ttq68quHz5cgQFBeGrr77
ULsG/fPnh6ej50vERkWuqnmRYVFaFnz56a56a9qKiIvj4+Dr+VAsVGIGUlBQsXboUc+bM0wZ
r0qULrK2tUVRUpHVRUpHVRUpHVMUVFRoymoulKp1A91fHsmCNqvTflciYiI2judH/rIzc3F4sWZ
Mweff/45rl+/rtc3Ho+rtc3r68qGBkZibFJiYm4desDesWDh061OQxaxWDh0Loke
8Oqgp6enli2bBn8/PywMH/4MwNk+MDGxsbpKamatpkMhkuXlpmDp1KtLT02FnZ9doe
8JCQlaCSIAiEQiDBkyKdOndJqTqT01NxdChQ1v/BIiIiIhamc5J4Y4dO5IdwvWa1CJVx8vxOZ
+/Xo8+eSTmDx5MhITE3Hu3LkW6Ve6rVUEAOHbsGEJCQrBv3z7y2tpqbWNNVQSLSlUQiQXh40OLj
43HkyBFkZmbi9ddfh4uLC0JCQlBXV4fbt2+jpqYGALBnzx4x4cOHAAr7/+OoYNG4bt29r/tTvM
2vWLHz33XXfYtmi0b8vLyEBsbi4yMDLz44ouGPFUiAIiKiFtHrmUR7e3tMnjwpjwjkydPhiAIOHXFD
766CNs2rQJn3zyCTIyMlrUj65VBRseL9+2vLasKAqZbbY9VBamjW7RoEVQFquvZAuFXqI
EBgZi69atkGkKCgoQHBwMGFhFoaUlBQ4AQGxsLGJjY7X6i9rn1KSkpCYmJkrOmfCY3VnLpJ
ffti48aNNPJORERERkGvJLGmpgZ4VkGmpgZpaWk4deoUTp06hQsXiUsHLkClUsHXz9vcT8PW1Wwqbgju1
9/DVBUETLvaHqsKUkdnZmaGqKgoREVFdrm7u6u6oRKwszdPhrW1PKClCmYMmVKa4VIRERE9M
jonCQ+99zFr6YPAwEDnDkTkTw4cPh7729vU596VtVtWbNmVtaDh4PqoxtdWMj3JU0+VkcIwNL2Q
QERERtR2dk8SsrCyo4VCryoVr4+vpi/PjxGcfGD5o8AAYdHzgrMzdv7k+VQUfpC2rCgKmW22PV
iKiezg+fnT59Gp9//f/jnGjRuHY8e4UXXkBgYCBefvlbN68GefX/pUFXwQVhUkIiIiI
iJ6ODrfSTQ3N8fLfQoUMdxohQLFy4ENXV1f/x5ZdfIi4uDgjrKRrAmfFaxpWFXRwcICbnZmxvi4u
K0qqqWlpbC1tZWazrqg8yaNQvz5s2Dr68vxowZg6+++w/6Bm6Jbcg84Dr68vxowZg6+//hoZGRl4//hoZGRl4//33dT1VIiIiKiDkevwjU
AUFxcjP/+9784ceIETpw4gcLCQnTv3h3cm8njx43xV5gxH7vR9ftacq2BKsKEhERERER6U/nJHH
cnNzIRaLMXjwjWYPztb3/D448/jn79+WrsFWTsgCjd7oSq/MCt5VBFj/1448/NtnOqoJEREREREERET60TlJHHEt60PaCCvmmOm3XLrZRQDgRcz+RSOKtlAEAuRyAXMEkN2NLpppkH0r5q+4Z4POmNCIfV
o2IiAiMGjUKdnZ2bREXEREREREREREREERGYDOSeJ///tfiBqBquvn6PiooKZ2x1kPDX3JpQ7MeAnGNZTS8sRtvCstqmeEWu2z7Ovq
XrsXv3bshkMgwdOhSrVq1Cr169mjYmoaldb3uTKD8/uiS4m9b98Gbt2Ukj3RRapsSIAMEPg4ds1V2Gkq8qR1R1j2mP1p7x7uCG+
mRecksba2Fp9++ilOnTqF2tpaCH+snyAIAqRyYp7IcFxJJM+9DEFVqIppTCwJpmcRMTq...

QATExMxOjRo3Ho0CFMnDix0TFpaWmIjo5GbW1tk4/+ZGdnQxAEDBkyhI8GEZHOdF4n8YcffsB
LL72Eb7/9Fi+88AIGDBiA3bt344cffoCbmxvUai7ETkRERNRSmZmZqKqqwogRIzRtdnZ28PX1
xenTp5s85tixYwgJCcG+ffuafMwnKysLzs7OTBCJSC8630ksLS3VXOXy9vbGl19+CQDo1q0b5
s2bh23btvFuIhEREVELFRYWAgBcXV212rt27YqbN282eczixYsf2Gd2djasra0RGRmJs2fPws
HBAWFhYZg5cybEYp3vEWiYm+t/bHvWsNyGSGS650nUUjoniba2tlAqlQAADw8P3Lx5E5WVlbC
xsdF8TUREREQtU11dDQCNnj2USqUoLy/Xq8+cnBxAxUVFQgNDQUCxcuxJkzZxAfH4/y8vJmE8z7
EYtFsLfvpNex7Z1YLNJ6barnSdRSOieJAQEB2LFjBwIDA+Hu7g4rKyscOnQIU6dOxdmzZ2FjY
9MWcRIRERGZpPpiMkqlUquwjEKhgJWVlV59btu2DQqFvO5zNvbG1VVVdiwYQMiIyP1upuoVg
uQyeR6xdPeqdWC1uuysioDRkPUMm15MUPnJHHBggUIDw/H/PnzsWPHDsyYMQNvvfUWduzYgay
sLDz//PNtESSRERGRSaqfZlpUVISePXXtq2ouKiuDj46NXnxYWFrCwsNBq8/LyglwuR3l5Oezt
7fXqV6UyzdoTgqD92lTPk6ildE4SfXx8cPDgQWRnZwMAlixZZAhsbG6SlpWHcuHGYN29eqwdJR
EREZKp8fHxgY2OD1NRUTZIok8lw+fJlhIeH69rCBTg5OemdIB
JRx6Fzkvj222/jmWeewciRIwEAIpEIr7zySqHRkRERNQRSCQShIeHIz4+Hg4ODnBzc0NcXBx
cXFwQEhKCuro6lJaWwtbWtkXrHIrFYowfPx5btmyBh4cH/Pz8cOLECWzZsgXR0dGP4IyIyNjp
nCTu378f48ePb4tYiIiIiDqkRYsWQaVSYeXKlaipqUFgYCC2bt0KiUSCgoICBAcHIyYmBmFhY
S3qb8mSJbCzs0NCQgIKCwvh7u6O6OhoTJ8+vY3PhIhMgc5J4sCBA/HLL78gKCioLeIhIiIi6n
DMzMwQFRWFqKioRtvc3d2RlZV132N//PHHJCJbB77//nv
07dsXjo6OWttFIhFWr17dagESEREREREHRo6Nzknjo0CF07doVAJCbm4c3Fyt7aKGq5ESERER
ERGRUdE5SWxqSgMRERERERGZBt1XUiUiIiKTxSSRiIiIiIiKTxSSRiIiIiINJgkEhERERERkQaTRCIi
iIiItJgkkhERERERREQaTBKJiIiIhIg0kiERERERERaRg8SVSr1Vi7di1Gjx6NQYMGYfbs2b
h69ep99y8rK8OK0SJUsQGBiIwMBA/P3vf4dcLtfaZ9y4cfD29tb6t2/bb68+abb7b1qRARERERERk9c0M
HkJSUhC+++AIxMTHo1q0bb4uLiMHfuXKSkpEAikTTTaf9GiRVAoFPj0008hk8kQQHR2Nd955Bx9+
+CEAoLKyEjdu3MCmTZvg5+eneZydnV2LnVkKRnRMREREREZGxMuidRKVSieTkZKZSieTkZERGRmLs2LHw8
mIibt26hUOHDjXa/Xa/+zZszh16hRiYmLg5+eHoKAgrvPvuu/j+++9RWlqq2dJHhO8Ur1o7tEnnvuiRT
ys+WNra/uoT4+IiIiMjoGDRJzMzRFVFUaMGaMG6Fps7Ozg6+HmenpRj4Rv6sE5hc3Eh2G29U25g5HbAX7GKCXOavEbx64Jq8nrw4NFkDLLlaBhu/gpBBHFEGZFErtHKqMvfRzLl8wMfA3xWlVNjVqPZVPXRoN5ABUjvZyHMaRCtZ3K
atmHDhkEkEuHXX38FAGRlZcHZ2Rl2dnZtfwJEREREQmxqDTTQsLCwEArq6uWu1du3bFzZs3
G+1/69atRvtKBJ06dJFs392djasra0RGRmJs2fPwsHBAWFhYZg5cybE4ofLic3NDf4IZ5sQi
bRfm+p5EhEREFR8wyaJFZXVwNAo2cPpVIpysvm9y/qecUpVIpFAoFACAnJwcVFRUIDQ3Fwo
ULcebMGcTHx6O8vByWy/WO1axWAR7+056H9+eicUirdemep5EREREQ8gyaJ9cVklEmE
ZhUIBKyurJvdXKpWN2hUKBaRSKQBg48aNqqqqgmNjAwA
ZhUIBKyurJvdXKpWN2hUKBaytrQEA27Ztg0KhgI2NDQDA29sbVVVV2LBhAyIjI/W+m6hWC5DJ
5M3vaITUakHrdVlZlQGjIWoeL2QQERERtR2DJon1U0eLior3+4eJkdZLvTXtm4Azlg1Ds2dPTXtRURF8fHyavFZU0dNTFQaM4Kjn8u/0KTmfQKhXFg0GwtJpJAJjlNzbBJoJYlhRxVlbZlRcVlqGtUekExJUXlQP...

4eHhOvenVqvx5JNPYtq0aYiIiNC0X7hwAU5OTnoniERkulr7YlWXLl1gbW2NoqIirfaioqJGU
1B1ZaoXcHihikgbJ1wTUYcmkUgQHh6O+Ph4HDlyBJmZmXj99dfh4uKCkJAQ1NXV4fbt25rpW8
0Ri8UYP348tmzZgoMHD+L333/Hl19+iS1btuj9PCIRmbaGF6vq1V+sCggI0Lk/kUiEIUOG4NS
pU1rtqampGDp06EPHS0Smj3cSCa5O1iiR3f0A3N2Jc/Cp41m0aBFUKhVWrlyJmpoaBAYGYuvW
rZBIJCgoKEBwcDBiYmIQFhbWov6WLFkCOzs7JCQkoLCwEO7u7oiOjsb06dPb+EyMF8ch6sgaX
qxycHCAm5sb4uLitC5WlZaWwtbWVms66oPMmjUL8+bNg6+vL8aMGYOvv/4aGRkZeP/999v9v4bI
wTxyAibSJBaHiDne6nrk6N0lLTnJ9+s6QKuw7nAABmPNkPro4cHKl9c3bueIvSm/IYBHAcIuP
T2uNQXV0d1qxZgz179mguVr311ltwd3dv9mLVuHHMXqVERGRmq179u3D0lJSSgsLETfvn0R
FRWFoKCgh4jRdMchjkFkjNry8xCTxBYy5YGRyNgwSSQiQ+M4RESG1pbjEJ9JJCIiIiIg0mi
UREREREKTBJJGIiIiIg0mCQSEREREGRBpNEIiIiIi0mCSSEREREEREBpcAqOFBEGAWs
0fFVF7YGbW8a5vcQwial84DhGRobXlOMQkkYiIiIiDQ63mUwIiIiIiui8miUREREREKT
BJJGIiIiIg0mCQSEREREGRBpNEIiIiIi0mCSSEREREEREBpMEomIiIiEiDSSIRERER
ERFpMEkkIiIiIiDSaJREREREEpMEkkYiIiIiDSYJBIREREEZEGk0QjJggC9u7di5KSk
vvuk5qaCm9vbxQUFDzCyKiet7c39uzZY+gwmvXTXTTz8hNzfX0GGQEeI41P5xHCJTx3Go/eM4ZH
yYJBqx06dPY/ny5aiurjZ0KHQfx48fR2hoqKHDeKDr16jlVdeeeB/rkT3w3Go/eM4RKaO41D
7x3HI+JgbOgDSnyAIhg6BmuHs7GzoEJrF3yN6GPz9af84DpGp4+9P+8dxyPjwTmI7l5OTg1df
fRXDhw/HgAEDEBISgu3btyM1NRUzZZ84EAAQHBzd7C//o0aOYHKSBgwYgIkTJ+Lnn3W/Wb1Kurq
8Onn36K8ePHY+DAgRg/fjz+/e9/a7anpqbC19cXJ0+eRGhoA+KGhoA/7yl7
9g2LBh+Mc//qH1D+ynn35CWFgYHnvsMYSEhOCf//wnlEpl6/6A2rmG0yuWL1+OqKgofPjhw
KCsKgQYPw6quv4vbt2w/sQy6X47333sOoUaMwePBg/O1vf8P58+c129PS0vC3v/0Njz32GB5/
/HG88847qKys1GwfN24cPvnkE0RGRmLw4MEYPnw4Vq9eDZVKChYgQHBwMAZs6ciXXr1gEA8
vLyMHfuXAwePBijRo3CkiVLtOJ84YUkOOJ84YUX8P/+3//DtGnTEBAQgH379rXWj4zaIY
PGjeOQEROKo3ZLL5cLIkSOFJUuWpWCLm5ucJvv/0mKCQkCF5eXnqNTGhgUlaPmxeu
FLy8v4amnnhJOjwpXLlyRXj+/HnhF198UYMGCZMmz/+4KGi+IKIgEw3EIwWCAT8PDZYk
YQUgtVVD0xjAEP1/5+4+J68l17SFbQ9pszF9xB5MFXlbmxNQjaVpJ/UyW6sRcYz1tLl0Cg/+/oflTQZtEpJ/8D8ZN0aAqHx2Nn8g3W
8EFGZtTpB9qEqeS84ZMV+x8cw+X6bKgtvz2Aw0dwWFQUYJFYhy8v8/+uH9GtCUdpA36bRmOrHmkCjJRtyIaeAKQZW/aKYNQyfcfBfUEoYkPHwdyWIYs8xYfX
2u/Lft4SVd6kgtS4/Lg/SDfHbwRVDTVYHXv7aZRyUHC9dodtPHNvZp+9Ls1i7s2c3/3kWjSGu1RtyBSa9d3k4G0Aw8L1N7vGjLpcY1gLbiFrQ72N77vdM0
GpfWnNPpj7kKx/zoSzcTywN0xNy+7xmf+GsDtqF+TbSCx39Svc8LLS5jZHfnRQ7JmS7h1Oc4x/9A/PvSlERlpZR5DcG97P8UcmDw8AQ2k1nhR/+/F0tIhlwLvOL/5lSPoIXiD/V6Qy
t6dnX3mhJsVbK+ac4C7bRm17eFvM0TsJaI9OvUfRjywL/nDmCnYnKsQFT42qXZXlZmkN+bXPhwgM7wIKUYj/Vc8DpB4FK9Ss2+5Io7SKXKqW3vPb4mHsvlWfXT

```
oi4X4TcYmIiIiIiKjD4XRTIiIiIiIi0mCSSERERERERBpMEomIiIiIiEiDSSIRERERERFpMEk
kIiIiIiIiDSaJREREREREpMEkkYiIiIiIiDSYJBIREREREZEGk0QiIiIiIiLSYJJIREREREE
GkwSiYiIiIiISINJIhEREREREWn8fygGGuzYsZ9CAAAAAElFTkSuQmCC",
      "text/plain": [
       "<Figure size 912.222x600 with 6 Axes>"
      ]
     },
     "metadata": {},
     "output_type": "display_data"
    }
   ],
   "source": [
    "df_ci = pd.DataFrame.from_dict(res_dict, orient='index')\n",
    "df_ci.to_csv('ci_change_score.csv')\n",
    "\n",
    "g = sns.FacetGrid(df_ci, col='task name',  col_wrap=3, sharey=False,
margin_titles=True)\n",
    "g = g.map(sns.pointplot,'program type','value', dodge=True)\n",
    "g.set_axis_labels('', 'raw change score')\n",
    "g.add_legend()"
   ]
  }
 ],
 "metadata": {
  "kernelspec": {
   "display_name": "base",
   "language": "python",
   "name": "python3"
  },
  "language_info": {
   "codemirror_mode": {
    "name": "ipython",
    "version": 3
   },
   "file_extension": ".py",
   "mimetype": "text/x-python",
   "name": "python",
   "nbconvert_exporter": "python",
   "pygments_lexer": "ipython3",
   "version": "3.9.7"
  }
 },
 "nbformat": 4,
 "nbformat_minor": 2
}
```
